# Changes in reflectance of rice seedlings during planthopper feeding as detected by digital camera: Potential applications for high-throughput phenotyping

**DOI:** 10.1371/journal.pone.0238173

**Published:** 2020-08-27

**Authors:** Finbarr G. Horgan, Artzai Jauregui, Ainara Peñalver Cruz, Eduardo Crisol Martínez, Carmencita C. Bernal

**Affiliations:** 1 EcoLaVerna Integral Restoration Ecology, Bridestown, Kildinan, Co. Cork, Ireland; 2 Environment and Sustainable Resource Management, University College Dublin, Belfield, Dublin, Ireland; 3 Unidad de Diseño, Departamento de Branding, Dirección de Comunicaciones Corporativas, Universidad de Talca, Talca, Chile; 4 International Rice Research Institute, Metro Manila, Philippines; 5 Laboratorio de Control Biológico, Instituto de Ciencias Biológicas, Universidad de Talca, Talca, Chile; 6 Department of Agroecology, COEXPHAL (Association of Vegetable and Fruit Growers of Almeria), La Mojonera, Almeria, Spain; Zhejiang University, CHINA

## Abstract

Damage to grasses and cereals by phloem-feeding herbivores is manifest as nutrient and chlorophyll loss, desiccation, and a gradual decline in host vigour. Chlorophyll loss in particular leads to a succession of colour changes before eventual host death. Depending on the attacking herbivore species, colour changes can be difficult to detect with the human eye. This study used digital images to examine colour changes of rice seedlings during feeding by the brown planthopper, *Nilaparvata lugens* (Stål) and whitebacked planthopper, *Sogatella furcifera* (Horváth). Values for red (580 nm), green (540 nm) and blue (550 nm) reflectance for 39 rice varieties during seedling seed-box tests were derived from images captured with a digital camera. Red and blue reflectance gradually increased as herbivore damage progressed until final plant death. Red reflectance was greater from plants attacked by the brown planthopper than plants attacked by the whitebacked planthopper, which had proportionately more green and blue reflectance, indicating distinct impacts by the two planthoppers on their hosts. Analysis of digital images was used to discriminate variety responses to the two planthoppers. Ordination methods based on red-green-blue reflectance and vegetation indices such as the Green Leaf Index (GLI) that included blue reflectance were more successful than two-colour indices or indices based on hue, saturation and brightness in discriminating between damage responses among varieties. We make recommendations to advance seed-box screening methods for cereal resistance to phloem feeders and demonstrate how images from digital cameras can be used to improve the quality of data captured during high-throughput phenotyping.

## Introduction

Crop breeding relies on the identification of useful traits during phenotyping studies and the eventual elimination from breeding programs of plants with relatively undesirable traits [1,2]. Host plant resistance is a valuable trait to reduce the damage and yield losses from insects and diseases to crops [3–5]. Breeding for crop resistance to herbivores has relied heavily on laboratory-based phenotyping to eliminate susceptible plants, to identify donor varieties, and to test breeding lines at successive stages of varietal development [3]. Host resistance and tolerance to herbivore and disease damage are often complex traits governed by several genes. The probability of isolating highly resistant or tolerant plants increases proportionately with the size of phenotyped populations. For example, during exploratory screening for resistance to insect pests in rice, normally less than 1% of plant materials has some resistance, indicating that resistance genes are frequently rare [6–8]. Phenotyping for cereal resistance to herbivores, including aphids and other sucking pests, is mainly conducted using seedlings grown under greenhouse conditions which speeds-up the tests and allows a greater throughput of plant materials [3,9]. In rice, 83% of published studies apply the Standard Seedling Seed-box Test (SSST) or the Modified Seedling Seed-box Test (MSST) [9] during phenotyping for planthopper and leafhopper resistance and for gene discovery (from 503 papers: Horgan, unpublished). Widawski et al. [10] have indicated that routine screening of rice varieties using bulk seedling screening has resulted in lower incidences of pest infestation in farmers’ fields in China. Furthermore, Horgan et al. [11] have indicated that attention to routine screening increased varietal resistance against the green leafhopper, *Nephotettix virescens*, in the Philippines, despite an apparent lack of deliberate introgression with leafhopper resistant donor varieties during varietal development.

Bulk seedling tests such as the SSST and MSST rely on visual recognition of damage levels (usually rated from 0 = undamaged to 9 = seedling death) by trained technicians during evaluation. Such methods are time-consuming and expensive, and evaluations can become subjective and tedious; however, they still remain among the least expensive methods for evaluating resistance and seem unlikely to be replaced by more rigorous phenotyping methods (e.g., based on herbivore fitness) any time soon [10,11]. Nevertheless, advances in sensors, digital imagery, data capture and data sharing, as well as robotics could be used to improve the efficiency of phenotyping from such traditional screening methods [1,12]. To our knowledge, there have been no previous empirical studies to assess the possibilities of using digital image analysis during high-throughput phenotyping for host plant resistance in bulk seedling tests [13]. However, automated and digital devices might be expected to reduce screening costs and increase throughput during the early stages of varietal development, where large numbers of plants must be quickly evaluated and where bulk seedling tests have become a standard practice.

A large number of recent studies have examined the potential for images captured with commercially available digital cameras to assess crop health and to support precision agriculture [14–16]. Several authors have proposed methods that can be applied to crop management by linking images through specialized or adapted software to give simple and informative read-outs upon which researchers, breeders or farmers can base their management actions [13,17–20]. Digital cameras can be used by technicians at ground level and are increasingly attached to drones for precision agriculture and forestry [15,21]. For example, digital cameras have been applied to assess plant colour for aesthetics in horticulture [19,22], or to assess nitrogen content in field crops to support decisions around applying fertilizers [18,23–26]. In breeding programs, crop biomass, plant height and form, canopy cover, and plant responses to climate can all be estimated using digital cameras [27–30]. Despite the progress, digital images are still not widely used in breeding for insect resistance or in assessing damage from insects to field crops [13,31]. Digitized images have been used in entomology and plant-pathology research, for example to estimate disease lesions or leaf-areas lost to leaf-chewing insects [16,32,33]. But damage to plants from phloem-feeding insects is more visually complex than damage from leaf chewers because it is manifest as colour changes over the entire plant and often lacks necrotic lesions or obvious losses to plant tissues and biomass [3].

Yang et al. [34] identified spectral characteristics (signatures) associated with planthopper and leaffolder damage to rice using spectroradiometry with potential applications for remote sensing. However, these authors highlighted the more useful signatures for grading damage to late stage crops as outside the range of visible light (i.e., ≈ 1400 nm; visible light = 380–740 nm). These results have been corroborated by several authors using similar methods [35–38]. Damage from phloem-feeders can be quantified using Soil-Plant Analyses Development (SPAD) meters [39] because damaged plants often turn yellow as they lose chlorophyll during the progression of insect attack [40,41]. However, SPAD meters are impractical for assessing chlorophyll from insect-damaged cereal seedlings because the leaves are often too narrow and tend to shrink and curl as damage progresses. A number of previous studies have shown that estimates of chlorophyll content derived from SPAD meters are often highly correlated with estimates based on calibrated digital images from commercially available digital cameras, allowing cameras to be used during assessments of plant health and nutrient requirements [29,42,43].

In the present study, we propose the development of a system whereby colour digital images could be used as a component of high-throughput phenotyping for insect resistance in rice (and potentially in other crops). We adapted a popular bulk seedling screening method (the SSST) for the efficient capture of digital images and assessed the accuracy with which images detect damage from brown planthopper, *Nilaparvata lugens* (Stål), and whitebacked planthopper, *Sogatella furcifera* (Horváth) feeding. The brown planthopper is regarded as one of the most damaging pests of rice in Asia and most rice-breeding programs in the region include some component of screening for planthopper resistance using the SSST [10,11]. The whitebacked planthopper is an emerging pest in Asia, largely due to the widespread adoption of hybrid rice varieties with cytoplasmic male-sterile (CMS) lineages. Many rice-breeding programs in northern India and China routinely screen for rice reactions to the whitebacked planthopper using the SSST [44]. We used rice varieties with known responses (susceptible, resistant, or tolerant) to the two species of planthopper based on several recent publications [11,45–47] and therefore against which the efficiency of methods to identify resistance could be examined. We conducted replicated SSSTs with the planthoppers and analyzed images of all rice varieties during the progression of each test to identify useful signatures for evaluating plant condition in response to planthopper infestations. To develop a standardized protocol, we further assessed the impact of screening conditions and camera settings on image quality and utility. To our knowledge, this is the first study to examine the possibility of using digital cameras as part of routine screening for resistance against common cereal pests. Based on our results we propose a model for phenotyping using digital images together with other, available technologies, to improve the information gathered from bulk tests such as the SSST or MSST.

## Materials and methods

### Herbivore species

The brown planthopper occurs throughout South and East Asia and in Australia. It is a common rice field insect that normally causes little damage where populations are kept in check by natural enemies; however, the planthopper can attain damaging population densities in fields with high applications of nitrogenous fertilizers and resurgence insecticides [48,49]. The brown planthopper feeds on phloem from the base of the rice plant and causes ‘hopperburn’ (patches of dead rice plants) during outbreaks. Rice plants killed by the planthopper often appear reddish-brown in colour and become desiccated [49].

The whitebacked planthopper is more widely distributed in Asia than the brown planhopper and has become an increasingly prominent pest of rice in recent years, often dominating planthopper communities in rice fields. Hybrid rice varieties with wild abortive CMS lineages are particularly susceptible to the planthopper. Because of the increasing areas of land dedicated to hybrid rice, whitebacked planthopper increasingly causes extensive losses to rice production, particularly in China and Northern Vietnam [44]. Plants damaged by the whitebacked planthopper become dehydrated, often turning a gray or straw colour.

A colony of brown planthopper and a colony of whitebacked planthopper have each been maintained at the International Rice Research Institute (IRRI) in the Philippines since 2009. Both colonies were initiated with > 500 wild collected individuals from Laguna Province, Philippines. The planthoppers were maintained in wire mesh cages of 120 × 60 × 60 cm (H × W × L) under greenhouse conditions (temperature: 25–37°C; natural light ca. 12D: 12N), and were continuously reared on the susceptible variety TN1 (ca 30 days after sowing). Feeding plants were changed every 3 to 5 days. Previous screening studies have indicated that the Laguna brown planthopper population is virulent against the *Bph1*, *bph2*, *Bph18*, *BPH25*, and *BPH26* genes for resistance and the Laguna whitebacked planthopper population is virulent against the *Wbph1* and *Wbph2* genes for resistance [11,45].

### Plant materials

We used 39 rice varieties in our experiments, including TN1 which was used as a susceptible control and natal host (i.e., the plant host on which test colonies were maintained). The 39 varieties have been extensively studied in recent years as possible sources of resistance against planthoppers, such that reactions to both planthopper species are relatively well documented [11,45]. The materials included donor varieties with the following genes for resistance to the brown planthopper: *Bph1* (Mudgo), *bph2* (ASD7), *Bph3(t)* (Rathu Heenati), *Bph4* (Babawee), *Bph5* (ARC10550), *Bph6* (Swarnalata), *Bph7* (Jia Nong 66); *Bph8* (Chinsaba), *Bph9* (Balamawee, Pokkali), *Bph10* (IR65482-4-136-2-2), *Bph18* (IR65482-7-216-1-2-B), *Bph 20* and *Bph21* (IR71033), *BPH25* and *BPH26* (ADR52), *Bph32* (Rathu Heenati, PTB33 and IR62), and *Qbph3* and *Qbph9* (Yagyaw). A number of varieties that tentatively possess either the *Bph1*, *bph2*, *Bph3(t)/Bph32* or *bph4* loci were also included (i.e., *Bph1*: IR24, IR64; *bph2*: IR40; *Bph3(t)* or *Bph32*: IR56, IR60, IR62, IR70, IR72, IR74; *bph4*: IR66). We also included varieties with the following genes for resistance to the whitebacked planthopper: *Wbph1* (N22), *Wbph2* (ARC10239), *Wbph4* (ARC6650), *Wbph5* (N’Diang Marie), *Wbph6* (Da Hua Gu), and *WbphN* and *WbphO* (MO1). The *japonica* variety Asiminori also has resistance to whitebacked planthopper through an ovicidal response, likely associated with an *Ovc* gene [50]. We included Utri Rajapan and Triveni as two varieties with noted tolerance to the brown planthopper [51,52] and IR22 as a further susceptible control. Further details of the varieties have been presented by Horgan et al. [11,45].

Seed was acquired from IRRI. Traditional varieties were acquired through the International Network for Genetic Evaluation of Rice (INGER). The IR varieties were acquired through the Plant Breeding, Genetics and Biotechnology (PBGB) Division of IRRI.

### Image capture and processing

Digital images were captured using a Nikon D90 (12.3 megapixel) digital camera (Nikon Corp., Tokyo, Japan). The images were collected in raw-format with colour depth of 68.7 billion colours (12 bit) and image size of 4288 × 2848 pixels (≈ 9.50 megabytes per image). Camera settings included a shutter speed of 1/200 s, aperture of f22, and a focal length of 24 mm. The camera was calibrated for exposure using the ColorChecker grayscale card (Gretag Macbeth), white-balanced using a ColorChecker whitebalance card (Gretag Macbeth) and colour-verified using the ColorChecker SG (Gretag Macbeth) before each series of data captures. The colour profile was obtained using ColorChecker Passport (X-Rite, Grand Rapids, MI, USA). Images were captured through a trapezoidal frame placed over individual patches (S1 Fig) of rice seedlings. After downloading the images to a personal computer, the corresponding colour profile was applied to each digital image using the Camera Raw 6.0 plug-in of Adobe Photoshop CS5 (Adobe Systems, CA, USA). Each image was opened in Photoshop, the shape of the leaves were selected with the Colour Range command (i.e., image segregation) and means for red (580 nm), green (540 nm) and blue (550 nm) reflectance were recorded from the displayed statistics in the histogram panel. The histogram maps the number of pixels at each colour intensity level (0 to 255). We also recorded displayed luminosity and chroma for each image.

### Greenhouse experiments

A series of experiments was conducted in a greenhouse to 1) assess optimal sowing and data capture conditions for the application of digital image analyses and 2) assess digital images for light and colour parameters that best indicate damage from brown and whitebacked planthoppers.

#### Image stability for digital image analyses

The SSST is normally conducted by planting test varieties as narrow rows into trays of soil. Rows are closely positioned to ensure that planthoppers can easily access and move between test varieties [3]. This sowing configuration is not suitable for the purpose of digital image analyses because of difficulties in ensuring that images capture information only from the test variety of interest. Therefore to adapt the SSST for digital images, we planted rice seeds for each variety in square patches with clear spaces (bare soil) between each patch (see below). We assessed how the size of these patches (5 cm^2^, 8 cm^2^, 10 cm^2^, 12 cm^2^ or 15 cm^2^), the configuration of seedlings within patches (sown to rows or broadcast) and the density of seedlings (1 cm^-2^ or 2 cm^-2^) affected images. We also assessed whether colour parameters of foliage changed significantly during development by staggering sowing such that seedlings of 8 and 14 days old were available at the same time under each treatment. Furthermore, we assessed optimal conditions for image capture by assessing two background colours (black and blue) and for images taken with and without a flash (i-TTL flash control that commands advanced wireless lighting). Backgrounds were based on the inside colours of trapezoidal frames (with base dimensions according to the size of seedling patches indicated above and a height of 25 cm: S1 Fig). Frames were placed over the seedlings and images captured from inside the frame. Seeds of TN1 were sown in patches to open trays of soil (110 × 60 × 10 cm [L × W × H]) on a greenhouse bench. Patches of different sizes, sowing configurations, seedling densities and ages were randomly interspersed on each soil tray. The set-up was replicated five times requiring 200 patches (40 × 5) and generating 800 images (160 × 5).

#### Images of plant reactions to planthoppers

Seed of the 39 test varieties were germinated directly in shallow (5 cm deep) metal trays with moistened paddy soil. Trays (described above) were divided into 45 squares of 5 × 5 cm (L × W) each separated by 2 cm by pressing a wooden frame onto the soil to leave an imprint. Seed of the test varieties were broadcast onto the squares at a density of 2 cm^-2^, with one variety per square. Seed of the susceptible control TN1 were sown to squares at each corner (4 patches) and at the centre (3 patches) of the trays (7 patches per tray). Two varieties (Da Hua Gu and Jia Nong 66) did not develop during some tests leaving one or two empty squares in each tray. Except for TN1, varieties were randomly assigned to squares with positions carefully recorded on trial maps. There were four trays for each of three experimental replicates (three replicates = 12 trays in total) corresponding to a brown planthopper-infested SSST and corresponding control, non-infested tray, and a whitebacked planthopper-infested SSST and corresponding control, non-infested tray. Two non-infested controls were used because of potential differences in the time for susceptible controls to die from either brown or whitebacked planthopper damage; however tests with both planthoppers were completed at the same time and data from the two controls were combined.

Seven days after sowing, the seedlings were thinned to 25 per square and infested with eight second instar nymphs of the test planthopper per seedling. After infestation, photographs of the squares were taken through a blue trapezoidal frame every day until the seedlings of TN1 were killed. Camera settings included a shutter speed of 1/200s, an aperture of f22, and a focal length of 24 mm. Images were standardized using a flash (i-TTL flash control). During the last day of each test, when the TN1 plants had died, experienced entomology technicians rated each variety from 0 to 9 using the Standard Evaluation System that regards scores of 0 to 3 as ‘resistant’, 4 to 6 as ‘moderately resistant’, and 7 to 9 as ‘susceptible’ (details of SSST protocols and the damage rating system are provided with S1 Table). Following evaluations, the numbers of dead plants in each square were recorded. All plants (living and dead) were also collected using scissors to cut foliage above the soil, and placed in individual paper bags. The plant materials were dried in a forced draught oven at 60°C and weighed.

### Data processing and analyses

Because the duration of replicated SSSTs varied from 6 to 10 days, the time of experiments was standardized by representing sampling days as a proportion of the overall time to complete each replicate. Days of evaluation were converted to nearest 0.1 interval proportions. The final evaluation for each replicate was therefore taken at proportional time 1. This facilitated comparisons of colour changes for the two planthopper species over time and facilitated comparisons between replicates. However, to evaluate relative damage, only data from the final days of each replicate, when TN1 had died, were included in analyses.

We converted our values for mean red, mean green and mean blue reflectance to hue, saturation and brightness according to Karcher and Richardson [19]. Further details about the interpretations of red-green-blue reflectance and hue-saturation-brightness can be found in Mendoza et al. [22]. We calculated brightness ratios (i.e., normalized red (r), normalized green (g), and normalized blue (b)) according to formulas presented by Yadev et al. [26]. We investigated a range of indices (Table 1) that have been developed to describe the colour of vegetation based on mean red-green-blue reflectance or hue-saturation-brightness values. Indices included the ‘Green, Red Difference’ (also referred to as ‘Green Minus Red’—GMR), the ‘Greenness Index’ (also referred to as ‘Green Divided by Red’—GDR), the ‘Normalized Green Red Difference Index’ (NGRDI [53,54]), the Variable Atmospherically Resistant Index (VARI [17,55]), the ‘Green Leaf Index’ (GLI [56]), the ‘Triangular Greenness Index’ (TGI [53]), and the ‘Dark Green Colour Index’ (DGCI [19]). Details of each index and their calculation are presented in Table 1. Information about the indices are available from original publications and have been reviewed by Hunt et al. [53] and Sanceechan et al. [21].

**Table 1:** Light and colour reflectance with derived indices used to evaluate planthopper damage to rice seedlings in the Standard Seedling Seed-box Test (SSST). Correlation coefficients (df = 37 rice varieties) and associated levels of significance for relations between each parameter or index and corresponding damage scores from the SSST or estimated seedling weight loss during the SSST are also indicated.

Colours and indices	Formula	Brown planthopper damage score^1^	Brown planthopper seedling weight loss^1^	Whitebacked planthopper damage score^1^	Whitebacked planthopper seedling weight loss^1^
Red reflectance (R)		0.699***	0.287	0.102	-0.103
Green reflectance (G)		-0.480***	-0.738***	-0.442***	-0.496***
Blue reflectance (B)		0.885***	0.611***	0.672***	0.396*
Chroma	max(R,G,B)-min(R,G,B)	-0.931***	-0.838***	-0.774***	-0.633***
r	R/(R+G+B)	0.414**	0.250	0.137	0.044
g	G/(R+G+B)	-0.954***	-0.792***	-0.863***	-0.635***
b	B/(R+G+B)	0.909***	0.781***	0.754***	-0.553***
Brightness (V)	max(R,G,B)	-0.284ns	-0.631***	-0.403**	-0.470***
Hue (H)	If max(R,G,B) = R, 60{(G-B)/[max(R,G,B)–min(R,G,B)]}	-0.710***	-0.519***	-0.550***	-0.444**
	If max(R,G,B) = G, 60(2+{B-R)/max(R,G,B)–min(R,G,B)]})				
	If max(R,G,B) = B, 60(4+{R-G)/max(R,G,B)–min(R,G,B)]})^2^				
Saturation (S)	Chroma/max(R,G,B)	-0.940***	-0.802***	-0.811***	-0.609***
GMR	G-R	-0.917***	-0.723***	-0.818***	-0.623***
GDR	G/R	-0.917***	-0.742***	-0.787***	-0.563***
NGRDI	(G-R)/(G+R)	-0.920***	-0.723***	-0.809***	-0.570***
VARI	(G-R)/(G+(R-B)	-0.891***	-0.692***	-0.772***	-0.549***
GLI	2×(G-R-B)/(2×G+R+B)	-0.962***	-0.785***	-0.857***	-0.625***
TGI	-0.5×190×(R-G)-(120)×(R-B)	-0.954***	-0.718***	-0.830***	-0.663***
DGCI	[(H-60)/60+(1-S = (1-V)]/3	0.908***	0.862***	0.740***	0.614***
Damage score^3^			0.773***		0.679***

^1^: *** = P ≤ 0.005, ** = P ≤ 0.01.

^2^: Red-green-blue levels (scale = 0–255) converted to proportions by dividing by 255.

^3^: Based on results from SSSTs.

We conducted repeated measures general linear models (GLMs) on red-green-blue and hue-saturation-brightness values for non-infested, brown planthopper-infested and whitebacked planthopper-infested TN1 plants to assess colour changes as the susceptible variety became progressively more damaged. We also used repeated measures GLMs to investigate how one (i.e., NGRDI, GLI and DGCI) of each type of index (i.e., based on red and green reflectance, based on red, green and blue reflectance, or based on hue-saturation-brightness) functioned in distinguishing a brown planthopper-resistant plant (PTB33) and a whitebacked planthopper-resistant plant (N’Diang Marie), or a plant with resistance to both planthopper species (Balamawee). Definitions of these four plants as resistant or susceptible to each planthopper species were based on knowledge of the varieties gained from previous studies by the research team and using the same insect colonies [11].

We also used permutational analysis of variance (PERMANOVA) to test whether red-green-blue reflectance values alone could meaningfully differentiate between the four rice varieties (TN1, PTB33, N’Diang Marie and Balamawee) on the basis of their reactions to planthoppers. PERMANOVA is a non-parametric method for multivariate analysis of variance [57]. PERMANOVA generates pseudo-F values (analogous to Fisher’s F-ratios) directly from resemblance matrices, and P-values are then obtained using permutations. The factors included in the PERMANOVA model were: ‘treatment’, considered as a fixed factor with three levels (brown planthopper-infested, whitebacked planthopper-infested, and control); ‘time’, considered as a fixed factor with 5 levels (6^th^ to 10^th^ time periods) and ‘variety’, considered as a random factor nested in ‘treatment’ with 4 levels (one for each of the 4 selected varieties). Additionally, two PERMANOVA models were run to test differences between brown planthopper-infested and control plants, and between whitebacked planthopper-infested and control plants. PERMANOVA pair-wise tests were conducted to analyse differences between levels of statistically significant factors and interaction terms. Canonical Analysis of Principal coordinates (CAP), a constrained ordination method [58], was used to visualize the differences shown in the PERMANOVA analyses.

We conducted Pearson correlations of derived indices using values for all varieties. Because of poor germination in two of the varieties, the number of varieties in the different tests varies from 37 to 39. We also conducted Spearman correlations to examine relations between derived indices and SSST results, and Pearson correlations to examine relations between indices and plant weight loss during the experiments. Because the Green Leaf Index (GLI) was clearly associated with changes in plant condition, we further examined whether this index could be used to potentially measure the ‘tolerance’ (i.e., comparative condition change; see discussion) of plants to planthopper herbivory. Because the SSST is a choice experiment and herbivore pressures on individual varieties were unequal, we examined condition change as ΔGLI = GLI_infested_-GLI_control_ for each variety and proportional condition change relative to TN1 as |ΔGLI| = GLI_infested-control_test/GLI_infested-control_TN1. Values for GLI, ΔGLI, and |ΔGLI|, as well as SSST damage scores and estimated plant weight reductions were examined using univariate GLMs. Post-hoc tests were conducted using Duncan’s many-to-one comparisons or Tukey’s pair-wise comparisons (as indicated with results). Residuals were plotted following all parametric analyses and were found to be normal and homogeneous.

## Results

### Colour changes of infested and control TN1

**Fig 1:**
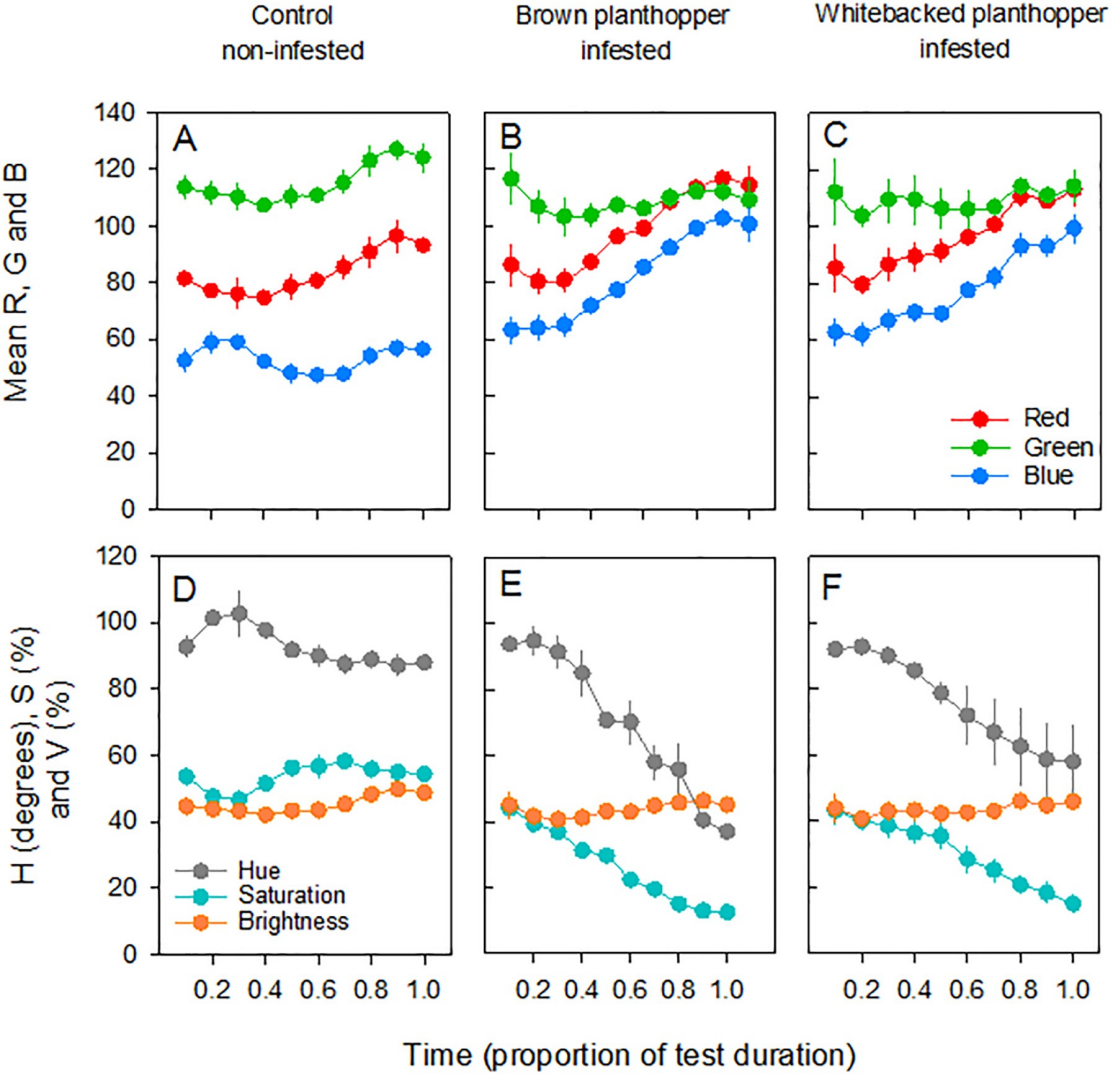
Mean red (R), green (G) and blue reflectance (B) based on digital images of (A) non-infested, control TN1 rice plants with (B) brown planthopper-infested and (C) whitebacked planthopper-infested TN1 plants for comparisons. Corresponding hue (H), saturation (S) and brightness (V) values are indicated for the same control (D), brown planthopper-infested (E), and whitebacked planthopper-infested (F) plants. Means are from three Standard Seedling Seed-box Tests (N = 3), each with seven sub-replicates for TN1. Standard errors are indicated.

Mean green reflectance (G) values varied over the course of the tests (being generally lower at mid-time points: F_9,54_ = 2.818, P = 0.009) but did not differ between treatments (F_2,6_ = 1.064, P = 0.402). Values for mean red reflectance (R) (F_9,54_ = 21.283, P < 0.001) and mean blue reflectance (B) (F_9,54_ = 30.821, P < 0.001) both increased over the course of the tests and were higher (R: F_2,6_ = 20.872, P = 0.002; B: F_2,6_ = 72.023, P < 0.001) for infested plants (Fig 1A–1C). There was a significant (time*treatment) interaction for blue reflectance (F_18,54_ = 9.268, P < 0.001) because of similar values at the start of the tests, but higher values for infested plants compared to controls at the end of the tests (Fig 1A–1C).

Brightness (V) remained relatively stable over the course of the experiment (albeit with a detectable increase during later stages of the tests: F_9,54_ = 3.471, P = 0.002) and was not affected by treatment (F_2,6_ = 0.617, P = 0.571). Colours became increasingly less-saturated (S) as the tests progressed (F_9,54_ = 142.024, P < 0.001) with lower values for infested plants (F_2,6_ = 50.506, P < 0.001) and a significant (time*treatment) interaction (F_18,54_ = 18.456, P < 0.001) because of similar purity of colours across treatments at the beginning of each test (Fig 1D–1F). Hue (H) similarly declined over the course of the tests (F_9,54_ = 33.038, P < 0.001) with a significant (time*treatment) interaction (F_18,54_ = 4.744, P < 0.001) because of similar colours at the beginning of the experiments regardless of treatment. There was a significant treatment effect on hue (F_2,6_ = 8.229, P = 0.019), but hue did not distinguish whitebacked planthopper-infested plants from controls (based on Tukey’s post-hoc tests: Fig 1D–1F).

Of the derived measures and indices, all, except normalized red (r), were capable of distinguishing infested from control plants over the course of testing (S2 Fig and S2 Table). Index values generally indicated the same patterns in colour progression including a greening effect of control plants between periods 2 and 5, a crossing of curves for brown planthopper-infested and whitebacked planthopper-infested plants at time period 2 due to relatively low mean green reflectance on brown planthopper-infested plants at time period 1; and a gradual separation of curves for brown and whitebacked planthopper-infested plants as the tests progressed (i.e., faster rate of increase in red versus green for brown planthopper-infested plants). Therefore, indices derived from mean red and green reflectance were sufficient to distinguish treatments. However, among the indices that included blue reflectance (i.e., GLI, VARI, TRI) or were based on hue (i.e., DGCI), there was a notable separation of control plants and infested plants as the tests progressed in time—largely due to the convergence of mean blue and mean red in infested plants, but a divergence in controls (see Fig 1A–1C). The indices that incorporated mean blue could, therefore, better discriminate infested and control plants than indices based only on green and red.

### Identification of resistance across test varieties

Index values across rice varieties were generally highly correlated (S3 Fig). Index bi-plots based on screening with brown and whitebacked planthoppers indicated three distinct groupings as 1) indices mainly based on red and green reflectance (i.e., GMR, GDR, NGRDI) or with a relatively low weighting for blue reflectance (i.e., VARI); 2) indices with a significant weighting for blue reflectance (i.e., GLI, TRI); and 3) the DGCI that was based on values for hue, saturation, and brightness. GLI and TRI were more closely correlated with DGCI than were the remaining indices (S3 Fig).

**Fig 2:**
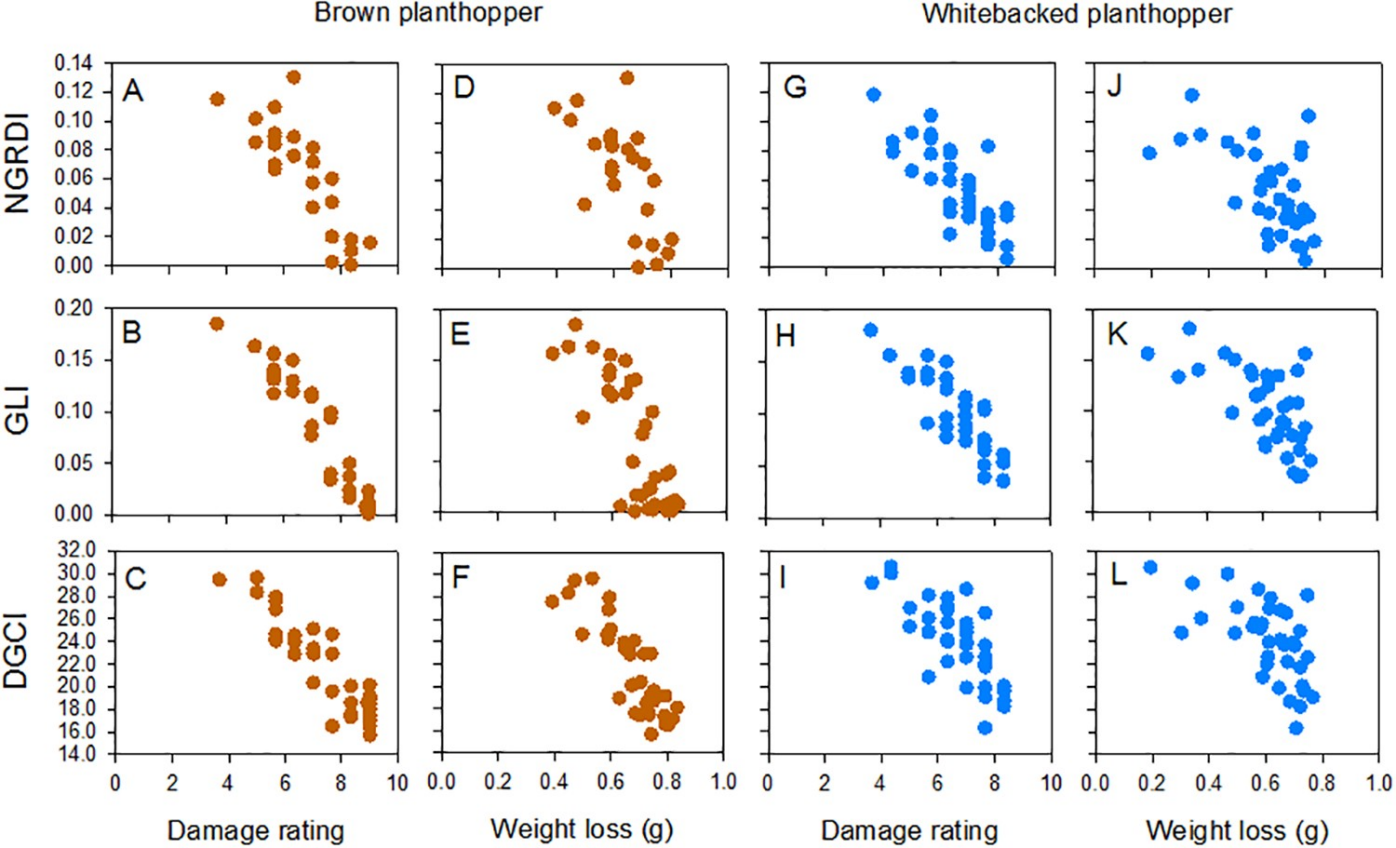
Correlations between colour indices and damage ratings or estimated weight-losses for 37 rice varieties after Standard Seedling Seed-box Tests with (A-F) the brown planthopper and (G-L) the whitebacked planthopper. NGRDI, GLI and DGCI were better correlated with damage ratings for the brown planthopper (A, B and C, respectively) than for the whitebacked planthopper (G, H and I, respectively). NGRDI, GLI and DGCI were better correlated with damage ratings (A-C, G-I) than with corresponding estimates of plant weight loss (D-F, J-L). Correlation coefficients are indicated in Table 1. Values for damage ratings and weight losses for each variety are presented in S3 and S4 Tables. Note that DGCI values are inversed in the figure.

Red-green-blue reflectance, hue-saturation-brightness, and index values were generally highly correlated with SSST damage scores as rated by technicians, and with estimates of plant weight loss from the tests (Table 1). Mean red and normalized red (r), mean blue, and brightness were the exceptions. Correlation coefficients were also generally lower for hue and mean green than for derived indices (Table 1). Red-green-blue reflectance, hue-saturation-brightness, and index values were generally better correlated with SSST results for the brown planthopper than for the whitebacked planthopper and better correlated with SSST results that with plant weight loss estimates for both planthopper species (Table 1, Fig 2). The highest correlation coefficients for SSSTs were normalized green (g), GLI and TRI, and for weight loss estimates they were chroma, DGCI (for brown planthopper) and TGI (for whitebacked planthopper) (Table 1).

**Fig 3:**
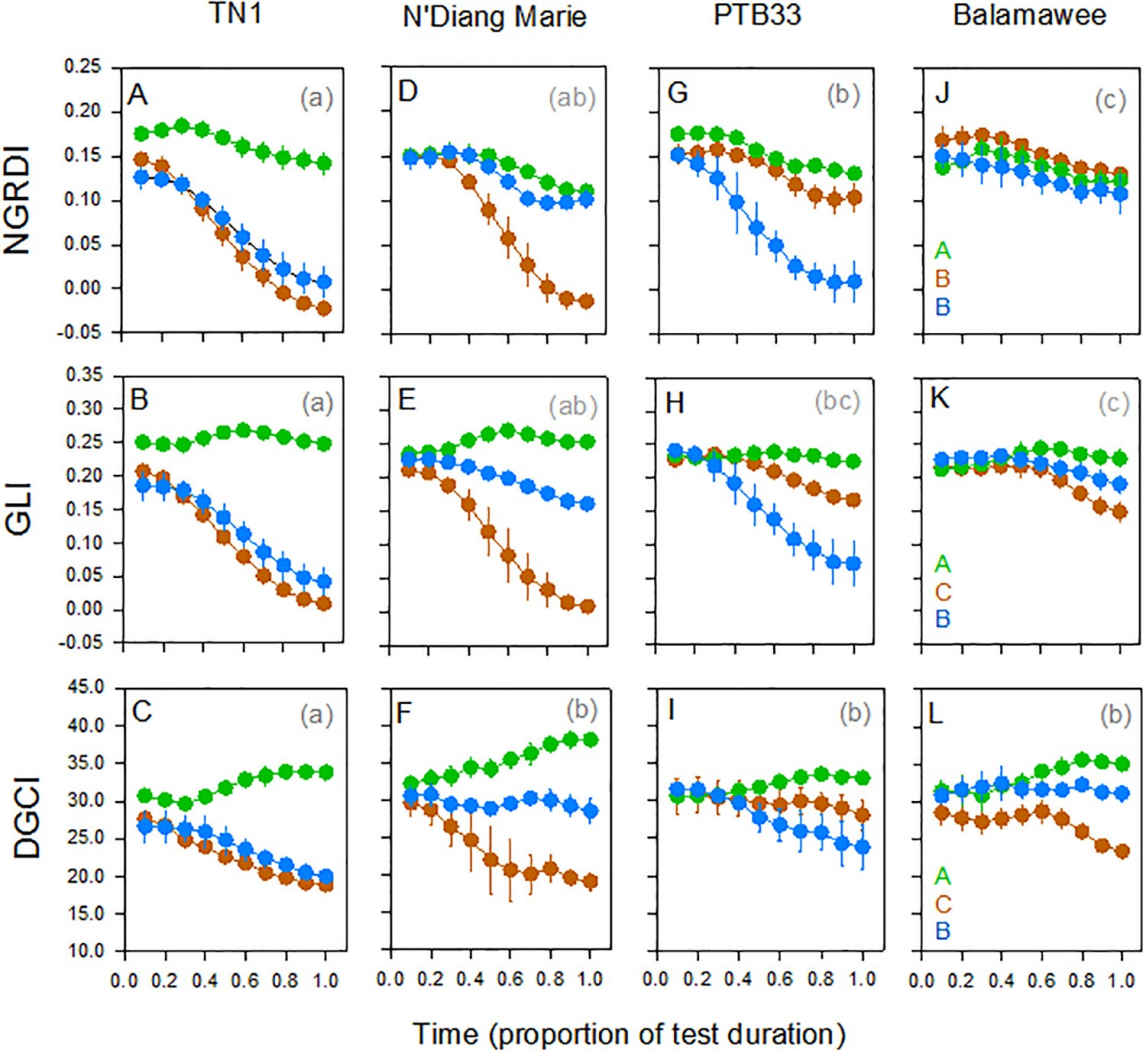
Changes in index values for control (green lines and symbols), brown planthopper-infested (brown lines and symbols) and whitebacked planthopper-infested (blue lines and symbols) seedlings of (A-C) TN1, (D-F) N’Diang Marie, (G-I) PTB33, and (J-L) Balamawee during Standard Seedling Seed-box Tests. Three indices are included as derived from mean red and green reflectance (NGRDI: A,D,G,J), mean red, green and blue reflectance (GLI: B,E,H,K) and hue-saturation-brightness (DGCI: C,F,I,L). Lower case letters indicate homogenous variety groups, uppercase letters indicate homogenous treatment groups (colours correspond with treatment symbols). Standard errors are indicated (N = 3).

Indices (NGRDI, GLI and DGCI) detected temporal changes in the conditions of a variety resistant to the brown planthopper (PTB33), a variety resistant to the whitebacked planthopper (N’Diang Marie), a variety resistant to both planthoppers (Balamawee) and a susceptible variety (TN1). All two and three-way interactions between time, treatment and variety were also significant (except for DGCI). They also indicated treatment, variety and between-subject interaction effects (Table 2). All three indices distinguished control plants from infested plants, but NGDRI did not distinguish brown planthopper-infested and whitebacked planthopper-infested plants (i.e., differences in levels and form of damage). GLI and DGCI distinguished resistant from susceptible plants, but only GLI indicated relative resistance strength (i.e., brown planthopper-resistant, whitebacked planthopper-resistant, or resistant to both planthoppers) (Table 2, Fig 3).

**Table 2:** Results of repeated measures GLMs for three vegetation-colour indices based on Standard Seedling Seed-box Test results for four rice varieties (see Fig 3).

Sources of variation	DF	NGRDI^1^	GLI^1^	DGCI^1^
*Within subjects*				
Time	9	87.382***	80.838***	7.188***
Time*treatment	18	6.793***	26.014***	11.004***
Time*variety	27	3.799***	4.968***	1.24ns
Time*variety*treatment	54	3.159***	4.504***	1.659**
Error	216			
*Between subjects*				
Treatment	2	23.206***	67.228***	32.833***
Variety	3	7.865***	12.244***	6.427***
Treatment*variety	6	9.259***	14.188***	3.712**
Error	24			

*** = P ≤ 0.005,

** = P ≤ 0.01.

**Fig 4:**
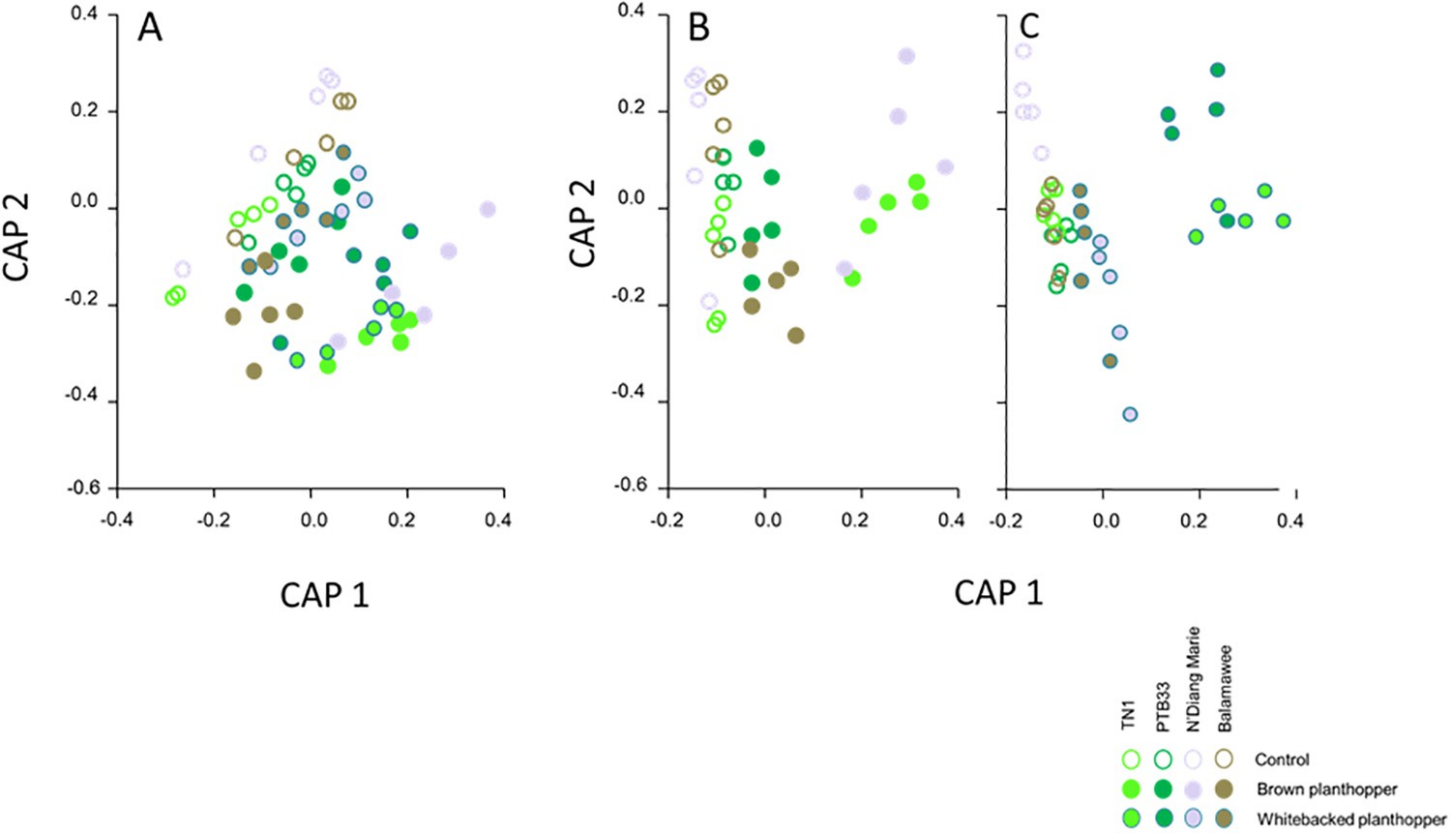
Bi-plots after Canonical Analysis of Principal coordinates (CAP) indicating clustering of four varieties and treatments with analysis (A) combined for both planthopper species, or with (B) brown planthopper alone and (C) whitebacked planthopper alone. Note that controls and ‘resistant’ varieties have values of CAP1 < 0.1 and CAP2 > -0.3 on each graph.

Red, green, and blue reflectance were relatively stable during the first five time periods with differences between treatments and varieties becoming apparent after period 6 (S5 and S6 Tables). PERMANOVA analyses of PTB33, N’Diang Marie, Balamawee and TN1 indicated significant effects of all factors on reflectance levels, with ‘treatment’ having, comparatively, the lowest effect (‘treatment’: Pseudo-F_2,59_ = 3.299, P = 0.033; ‘time’: Pseudo-F_4,59_ = 27.396, P = 0.001, and; ‘variety’: Pseudo-F_9,59_ = 20.747, P = 0.001). Pair-wise comparisons detected significant differences between brown planthopper-infested and control plants (t = 2.255, P = 0.028) and between whitebacked planthopper-infested and control plants (t = 2.228, P = 0.029), but not between brown- and whitebacked planthopper-infested plants (t = 0.643, P = 0.680: see CAP bi-plots in Fig 4). Also, pair-wise comparisons across rice varieties showed significant differences between all pairs (all P < 0.05: Fig 4). Pair-wise comparisons across time showed that all time periods were significantly different from each other (all P < 0.05) except the 9^th^ period, which did not differ from the 8^th^ (t = 0.723, P = 0.497) or 10^th^ period (t = 0.655, P = 0.556).

### Damage assessment and comparative condition change

Green Leaf Index (GLI) values highlighted 13 varieties as significantly less damaged by the brown planthopper than the susceptible control TN1. This included four more varieties than were highlighted by the SSST and included Balamawee that was apparently resistant to both the brown and whitebacked planthopper according to a variety of indices, as well as Swarnalata that received an average damage score of 7.0 in the SSSTs (S3 Table and S4 Fig). GLI values highlighted four varieties as less damaged by the whitebacked planthopper than TN1; these included N’Diang Marie that was not statistically different from TN1 according to the SSSTs. Two varieties, IR24 and Rathu Heenati, were highlighted in the SSSTs as more resistant than TN1, but showed no significant differences from TN1 according to analysis of GLI values (S4 Table and S6 Fig).

**Fig 5:**
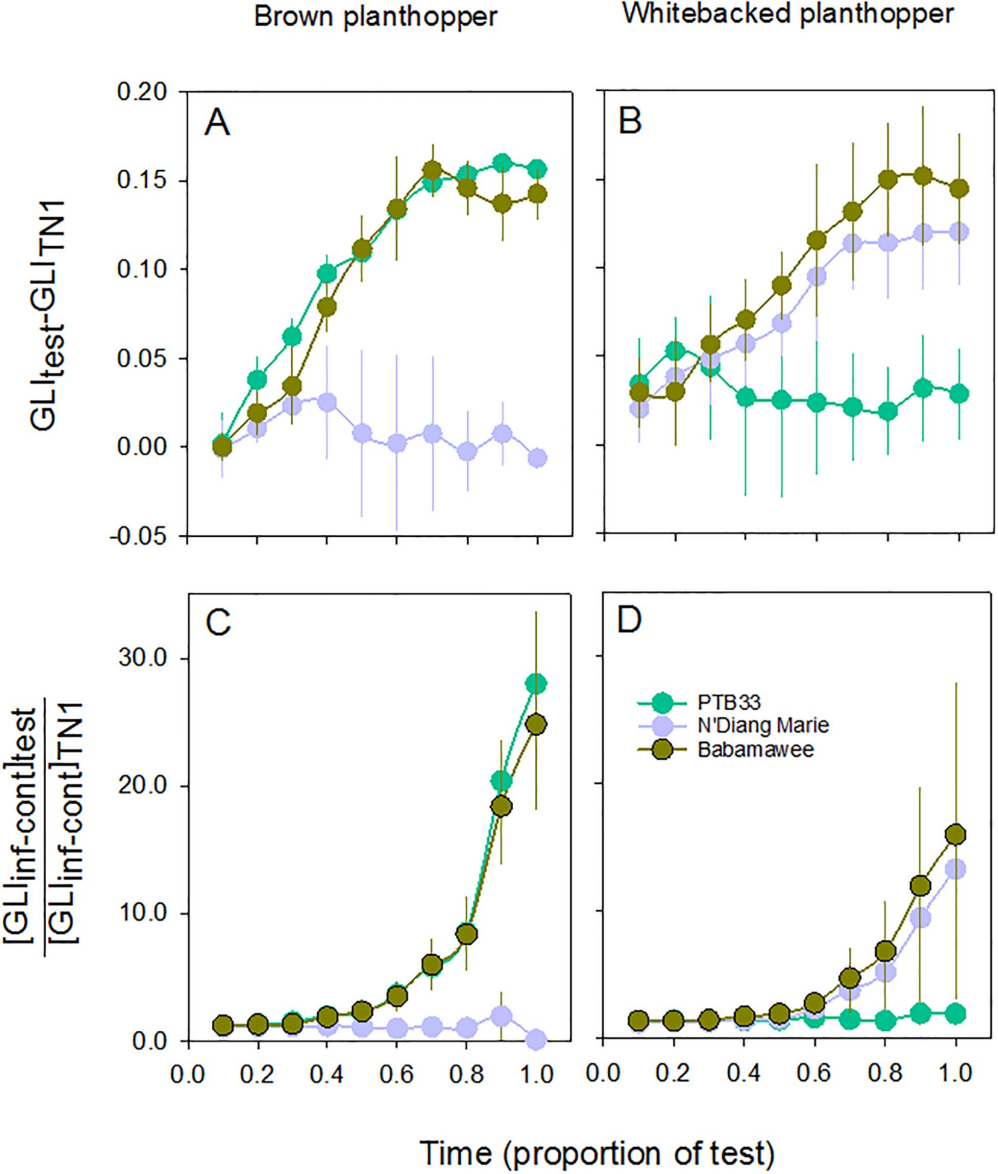
Comparisons of (A,B) relative damage and (C,D) changes in condition of PTB33 (resistant to brown planthopper), N’Diang Marie (resistant to whitebacked planthopper) and Balamawee (resistant to both planthoppers) after infestation with (A,C) brown or (B,D) whitebacked planthopper in Standard Seedling Seed-box Tests. Results and analyses for 37 varieties are included in S3 and S4 Tables and S4–S7 Figs. Standard errors are indicated (N = 3).

Seven varieties (i.e., Rathu Heenati, MOI, PTB33, IR62, IR65482-4-136-2-2, IR74 and Balamawee) were highlighted as maintaining better condition than TN1 during tests (i.e., **ΔGLI**); all of these varieties, except Balamawee, were also highlighted after analyses of damage scores from the SSSTs (S3 Table). The least damaged plants were between 23 and 30 times better than TN1 (i.e., **|ΔGLI|**) in maintaining condition during the tests (S3 Table and S5 Fig; Fig 5). No varieties were highlighted in the analysis of condition change during tests with the whitebacked planthopper. This relatively low resolution was due to large variability around GLI values based on SSSTs with this planthopper species (S4 Table and S7 Fig; Fig 5). Differences in condition change measured as seedling weight losses were generally not statistical significant for either planthopper species (S3 and S4 Tables).

### Image stability

**Fig 6:**
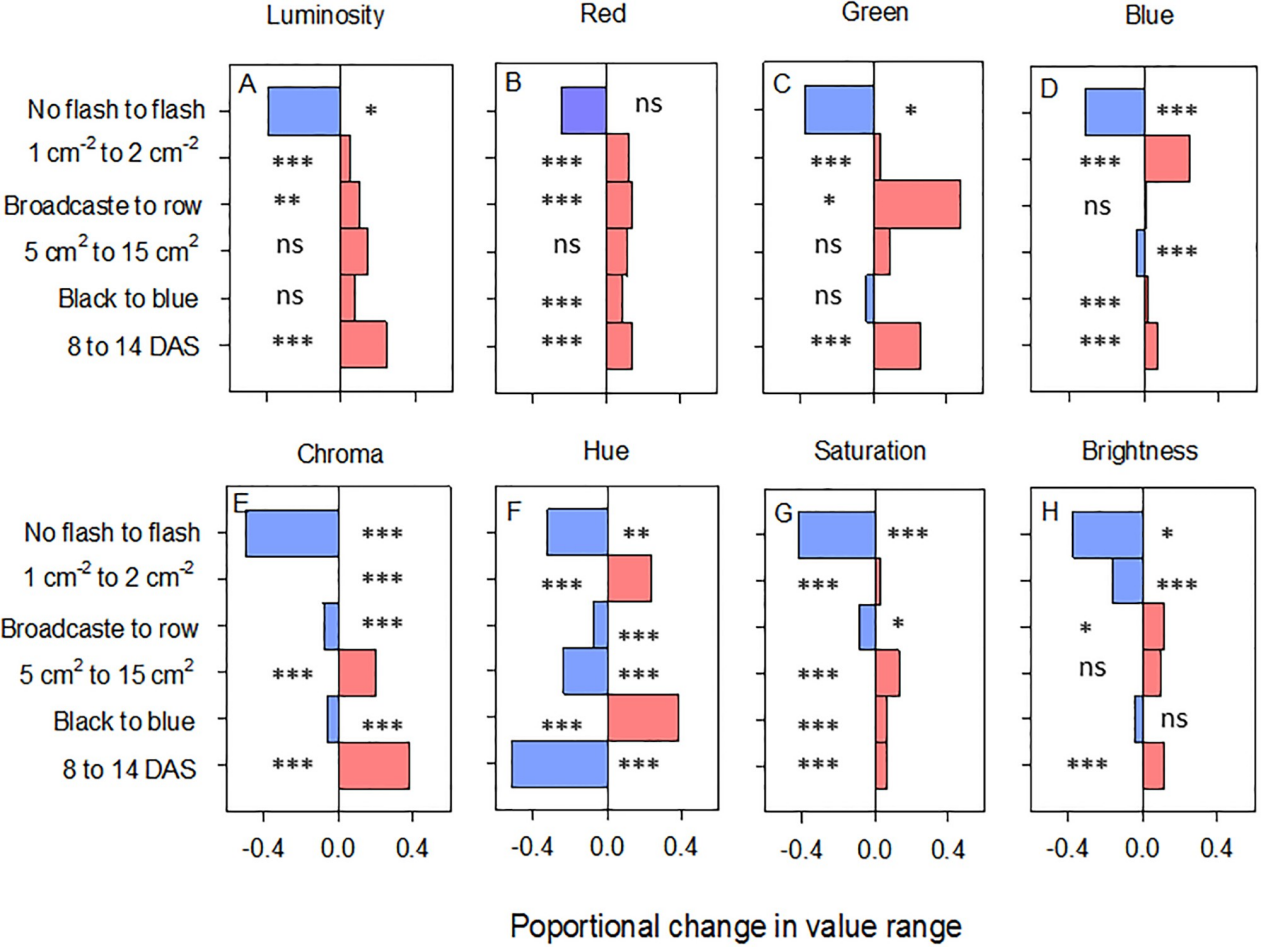
Changes in value ranges of (A) luminosity, (B) mean red reflectance, (C) mean green reflectance, (D) mean blue reflectance, (E) chroma, (F) hue, (G) saturation and (H) brightness as related to changes in conditions during screening (age of plants, size of patch, density and sowing regime) and during data capture (i.e., use of flash, colour of trapezoidal frame). Further details of the effects are presented in S8 Fig and S7 and S8 Tables. Levels of significance based on GLM analyses are indicated as *** = P ≤ 0.005, ** = P ≤ 0.001, * = P ≤ 0.05, and ns = P > 0.5 (N = 5).

Growing conditions, frame colour and the use of a flash had varying effects on luminosity, red-green-blue reflectance, chroma, and hue-saturation-brightness (Fig 6; S8 Fig and S7 Table). Chroma, saturation and hue were most sensitive to plant growth conditions (i.e., planting density, distribution, patch size and plant age, whereas mean blue reflectance was most sensitive to the use of a flash and the colour of the frame (Fig 6). The effects of plant-rearing conditions were reduced by using a flash; however, the flash also reduced the magnitude of colour differences due to plant age—although patterns across samples were maintained for blue reflectance and derived GLI values (S7 Fig). The flash therefore reduced variability between images taken under different conditions while the use of the GLI neutralized the effects of frame colour and further highlighted changes in blue reflectance.

## Discussion

Changes in red, green and blue reflectance as detected by digital camera in the present study can be used to assess damage to rice seedlings from two species of planthopper. Discrimination of rice reactions to planthopper feeding were best achieved using indices that incorporated blue reflectance, or through ordination methods based on combined mean values for red, green and blue reflectance. Yang et al. [34] used spectroradiometry to identify signatures (spectral characteristics) from mature potted rice plants (i.e., early grain-filling phase) damaged by the brown planthopper. Strong correlations between reflectance at 1450 nm and grades of hopperburn indicated that gross changes in canopy colouration (mainly due to a loss of chlorophyll) and plant structures (including due to dehydration) could be discriminated on the basis of changes to near-infrared reflectance. The authors suggested that the alteration of internal plant tissues due to planthopper feeding resulted in a substantial reduction in reflectance at about 745 nm, which is generally highly stable in undamaged rice plants [59]. Increased reflectance in the shortwave infrared zone (1300–2400 nm) also suggested that the plants had become water stressed due to planthopper feeding [34]. Without specialized filters, digital cameras cannot assess near-infrared reflectance. However, Yang et al. [34] also detected significant correlations between reflectance at 426 nm and planthopper damage—indicating the sensitivity of near blue reflectance to planthopper damage in older rice plants.

Although changes in reflectance as a response to planthopper damage are small at shorter wavelengths compared to longer wavelengths [34,38], variability in the reflectance of visible light was sufficient to determine grades of damage to rice planthoppers in the present study. By following the progression of damage to TN1 plants over time, and by comparing images across 36 other rice varieties, we demonstrated the limitations of using single reflectance measures (e.g., red reflectance, green reflectance, hue, etc.) and indices based on two colours only (usually red and green reflectance) during discrimination of damage levels. We found that hue was insufficient to distinguish between TN1 damage from the brown and whitebacked planthoppers due to the large variability between replicates. Similarly the DGCI which is based on hue-saturation-brightness (Table 1), although it separated control from damaged plants, was insufficient to distinguish between successive levels of damage from the brown and whitebacked planthoppers. Normalized green (g) and blue (b) were useful to distinguish treatments, as were a range of indices based on mean red and green reflectance. However, by including blue reflectance, index values for control and infested plants tended to better diverge over the course of the tests. This was due to increasing proportions of mean red relative to green reflectance as control plants aged. Although blue reflectance also increases with plant age, the proportional change in blue relative to green reflectance was smaller (see Fig 1). In contrast, as damage progressed over time, mean red and blue reflectance increased by similar amounts. Therefore, by applying three-colour indices, noise from background changes in plant colour due to aging was effectively reduced.

Among the indices that we considered, VARI, GLI and TRI all included mean blue reflectance; however, with VARI, the difference between red and blue reflectance is used only as a denominator, with greater weights given to red and green (Table 1). VARI was originally developed to control for blue reflectance and to compensate for atmospheric effects in remote sensing images under high saturation [17]. However, because it included blue reflectance, VARI was found to be more sensitive to vegetative fractions than two-colour indices such as NGRDI [17]. TRI and GLI were similarly developed to incorporate blue reflectance as an improvement over two-colour indices for determining the chlorophyll content of crops at both leaf and canopy scales [53,56]. In general, TRI behaved similarly to GLI in our analyses. We found that VARI approximates NGRDI during the early stages of planthopper damage, but more closely aligns with GLI during later stages as blue reflectance becomes more prominent. Although this resulted in a greater magnitude of change compared to either NGRDI or GLI during the progression of damage, VARI was more sensitive to colour changes as plants aged, creating greater variability in the control plants than was observed using the other indices and thereby reducing the discriminatory power of tests. We recommend GLI over TRI for phenotyping seedling responses to planthoppers only because it is more easily comprehended and easier to calculate, otherwise, both indices were equality effective for evaluating SSST results. Importantly, analyses of GLI values gave a higher resolution for damage estimates compared to damage scores from the SSSTs and indicated four more varieties with relatively low damage to the brown planthopper (each with demonstrated resistance according to more detailed fitness bioassays [11]). Similarly, analysis of GLI values indicated N’Diang Marie as significantly less damaged by the whitebacked planthopper, but this variety was not identified as resistant according to SSST damage scores. However, Rathu Heenati with resistance to whitebacked planthopper at later growth stages [11], was indicated as resistant based on SSST damage scores, but not GLI values; meanwhile, IR24, which is susceptible to planthoppers [11], was falsely indicated as ‘resistant’ based on SSST damage scores.

Hunt et al. [53] demonstrated that TRI (and presumably related indices) performs better when the leaf area index is >2. Damage to rice crops from planthoppers, particularly at early crop stages (when damage from the whitebacked planthopper is most intense [50,60]), reduces the leaf area index and exposes background soils, which limits the effectiveness of digital images in determining damage. The remote detection of damage from planthoppers during early crop stages—stages at which damage is more prevalent—is therefore limited even when using TRI and GLI. The analysis of individual leaves is also limited because digital cameras, spectroradiometers and other sensors have detector lenses that require larger fields of view [34] than the width of a damaged rice seedling. Our screening tests overcame these limitations by using high densities of seed in square patches that provided a relatively homogenous stand for evaluation without soil exposure. Although increasing seedling densities also reduced GLI values as the plants tended to elongate and age under higher densities, the effects did not impact evaluations because the patches of different varieties were standardized through the test protocol.

Researchers generally use the same evaluation systems for the brown and whitebacked planthoppers during phenotyping [3]; however our results indicate that rice plant responses to the two species and the manifestation of damage from each planthopper are slightly different. Whereas feeding by the brown planthopper rapidly depletes plant resources to cause eventual desiccation of the plant (referred to as hopperburn), plants infested with the whitebacked planthopper can maintain a blue-green colour until the plant dies or is no longer suitable for insect feeding. In our study, the visual aspects of whitebacked planthopper damage during SSSTs were more variable than for damage from the brown planthopper; for example, variability in hue between replicates of the SSSTs with the whitebacked planthopper was relatively large and analysis of variance was less successful in identifying resistant varieties. Furthermore, when evaluated using the Standard Evaluation System, some varieties that die due to whitebacked planthopper infestation remain blue-green for several days, obscuring plant death and delaying assignation of a damage score of 9). In effect, SSSTs with the whitebacked planthopper often end when TN1 scores are between 8 and 9. The pathology of the whitebacked planthopper therefore includes changes to rice plant colour and form that are not easily detected by human evaluators, and standard cut-off points (i.e., 3 = resistance, 5 = moderate resistance, etc.) do not transmit easily to tests with this planthopper species, particularly when conducting comparative screening against the brown planthopper or other phloem-feeders (i.e., leafhoppers, *Nephotettix* spp.). Several researchers have already indicated that SSSTs have a limited capacity to identify rice resistance to the whitebacked planthopper compared to, for example, field screening [61,62]. This has led some researchers to adopt an SSST for whitebacked planthopper that uses older seedlings where relative ranges of damage may become more apparent [63]. Our results indicate that some of the issues of SSSTs relate to the imperceptibility to evaluators of minor colour changes to plants during whitebacked planthopper attack. A higher resolution from digital images meant that potentially important varieties were rated highly when red, green and blue reflectance was analyzed. Our results therefore indicate that some of the short-comings of the SSST for evaluating host responses to the whitebacked planthopper can be overcome by recording digital images.

Apart from the possible imperceptions of subtle colour changes, evaluations based on the visual appraisal of damage by technicians are also subject to evaluator biases. Bock et al. [16] refer to this as rater (= evaluator) reliability defined as ‘the extent to which the same measurement of individuals obtained under different conditions yield the same results’. During assessments, two aspects of reliability affect damage comparisons. These are intra-rater reliability (affecting the repeatability of tests) and inter-rater reliability (affecting the reproducibility of tests)[16]. Because planthoppers are migratory insects that travel 1000s of kilometres during spring migrations, research institutes across Asia tend to collaborate and share results during the development of resistant rice varieties [45]. Therefore it is important that the results of screening tests should be reproducible and meaningful in the context of different evaluators, different institutes, and different planthopper pests. Based on our results, we suggest that digital image analysis will be more reliable than rater evaluations and can provide better estimates to compare damage between planthopper species. Furthermore, depending on the index that is used, even slight planthopper damage can be detected using digital images at the early stages of the SSSTs. For example, index values were generally different between control and infested plants even on the first day after infestation in our study, and between brown planthopper-infested and whitebacked planthopper-infested plants toward the end of the tests (e.g., Balamawee on the final day of evaluation, using GLI—but not NGRDI). Our PERMANOVA analyses indicated that plants could be differentiated on the basis of combined red, green and blue reflectance by day 6 and before TN1 plants had actually died. Such high resolution may be particularly useful for longer duration screening tests including MSSTs and for field evaluations [3,9]. Thus, this paper presents a novel approach for using multivariate statistics of combined red, green and blue values to evaluate resistance to planthoppers. Previous studies have used similar methods for environmental assessments, such as evaluating phytotoxicity to grassland plants [64] or the impact of nitrogen enrichment on Mediterranean maquis vegetation [65].

**Fig 7:**
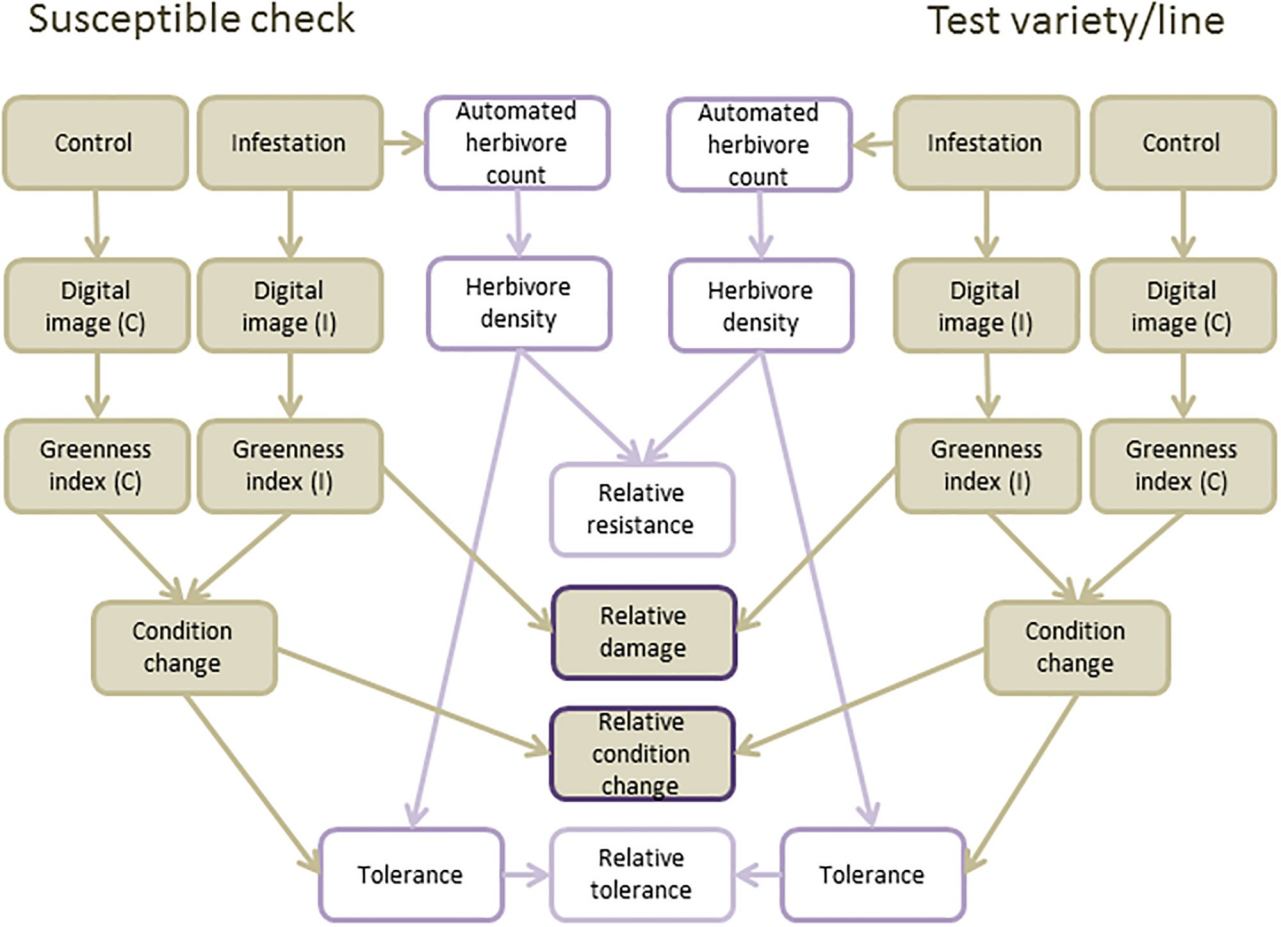
Methods for improving the quality of data captured from bulk phenotyping tests using sensors (including digital cameras) and automation. Brown rectangles indicate components of phenotyping that were addressed in the present study to give results as ‘relative damage’ and ‘relative condition change’ (brown squares with purple outline: based on comparisons between test varieties/lines and the susceptible check/control). Open rectangles indicate how quantification of herbivore fitness during no-choice bioassays using available sensors could transform phenotyping to provide estimates of ‘relative resistance’ and ‘relative tolerance’.

The SSST and MSST, whether evaluated by human eye or digital camera, will only give information on relative damage to test varieties where insects can choose feeding plants. However, plant-herbivore interactions are classified into a number of categories that include resistance and tolerance (Fig 7). Resistance is the ability of a plant to defend against an insect thereby reducing the insect’s fitness and limiting damage. Tolerance is the ability of a plant to compensate for damage [5]. Both tolerance and resistance are relative measures and are often negatively correlated, particularly in sap-sucking insects such as planthoppers [66]. Therefore, it not possible to distinguish resistance from tolerance based on damage scores from the SSST or other choice feeding tests. Researchers will often conduct further tests on selected varieties and breeding lines to clarify the nature of plant-herbivore interactions [46], but in many cases, varieties are selected based entirely on SSST results and carried forward in breeding programs without further investigation. By assessing the greenness of seedlings, SSST results can be presented as a continuous, quantitative variable that better represents damage as a relative measure.

Tolerance is measured by comparing plants receiving at least two levels of quantified herbivore pressure (one of which can be zero) [46,67,68]. Estimates of tolerance also require that resulting changes to plant condition are quantified. Plant condition can also be assessed using dry weights, tiller numbers, or yields [40,68,69]. Several researchers have measured tolerance in terms of changes to photosynthesis (measured using CO_2_ absorption) or chlorophyll content [40,41]. Greenness is closely related to chlorophyll content [29,40–42] and can therefore be used to determine relative levels of tolerance across test varieties in a manner analogous to our calculations of relative condition change (i.e., |ΔGLI|). Our study used non-infested controls to compare scales of plant damage, but because insects could freely move between plants, we did not determine comparative levels of tolerance—but rather, indicate relative changes in plant condition. To assess relative levels of resistance and tolerance, researchers could adjust bulk tests such as the SSST to control herbivore pressures and incorporate non-damaged controls. For example, a range of sensors have been developed to count planthoppers or otherwise estimate herbivore densities [70–73] or to measure plant physiological responses to herbivore feeding [74,75]. Indeed simply weighing planthoppers at the end of a test could substantially increase knowledge gained from bulk tests [76]. To further improve tests, insects could be confined to test plants—thereby turning a choice test to a non-choice test. This might not be recommended where antixenotic resistance is desired (i.e., resistance against ovipositing planthoppers), but will improve the characterization of rice-herbivore interactions where researchers aim to increase antibiosis resistance (as when using the SSST). By automating screening tests using robotics and sensors as depicted in Fig 7, and by applying analyses of digital images from commercially available cameras or specialized spectroradiometers, phenotyping could be substantially improved.

## Supporting information

S1 Fig Diagram of inverted trapezoidal frame. (DOCX)

S2 Fig Comparisons of raw values and derived indices representing control, non-infested TN1 seedlings, BPH-infested TN1 seedlings and WBPH-infested TN1 seedlings. (DOCX)

S3 Fig Correlation matrix indicating correlations between a range of greenness indices for 38 rice varieties exposed to brown planthopper or whitebacked planthopper. (DOCX)

S4 Fig Results for damage relative to TN1 from three runs of the adapted-SSST for phenotyping of rice for resistance to the brown planthopper, *Nilaparvata lugens*. (DOCX)

S5 Fig Results for condition change relative to TN1 from three runs of the adapted-SSST for phenotyping of rice for resistance to the brown planthopper, *Nilaparvata lugens*. (DOCX)

S6 Fig Results for damage relative to TN1 from three runs of the adapted-SSST for phenotyping of rice for resistance to the whitebacked planthopper, *Sogatela furcifera*. (DOCX)

S7 Fig Results for condition change relative to TN1 from three runs of the adapted-SSST for phenotyping of rice for resistance to the whitebacked planthopper, *Sogatela furcifera*. (DOCX)

S8 Fig Mean values (± SEM) for luminosity, mean R, mean G, mean B, and derived GLI from digital images of TN1 seedlings reared under different test conditions. (DOCX)

S1 Table Damage scores for brown and whitebacked planthoppers in standard seed-box screening tests according to the Standard Evaluation System for rice. (DOCX)

S2 Table Results of repeated measures GLM colour and index responses to control, BPH-infested and WBPH-infested TN1 plants in seed-box tests. (DOCX)

S3 Table Results of SSSTs for 37 rice varieties exposed to the brown planthopper. (DOCX)

S4 Table Results of SSSTs for 37 rice varieties exposed to the whitebacked planthopper. (DOCX)

S5 Table Results of permutational MANOVA. (DOCX)

S6 Table Pairwise tests from permutational MANOVA. (DOCX)

S7 Table Results of univariate GLM for effects of test and light conditions on colour space parameters. (DOCX)

S8 Table Results of univariate GLM for effects of test conditions on plant numbers and weight in SSST tests. (DOCX)

S9 Table Reflectance data from standard seedling seed-box tests. (DOCX)

S10 Table Damage scores and plant weights from standard seedling seed-box tests. (DOCX)

S11 Table Data from condition experiments. (DOCX)

## References

[1] Araus JL, Cairns JE (2014) Field high-throughput phenotyping: the new crop breeding frontier. Trends in Plant Science 19: 52–61. 10.1016/j.tplants.2013.09.008 24139902

[2] Yang W, Duan L, Chen G, Xiong L, Liu Q (2013) Plant phenomics and high-throughput phenotyping: accelerating rice functional genomics using multidisciplinary technologies. Current Opinion in Plant Biology 16: 180–187. 10.1016/j.pbi.2013.03.005 23578473

[3] Heinrichs E (1985) Genetic evaluation for insect resistance in rice. International Rice Research Institute, Manila, Philippines.

[4] Horgan F (2017) Integrated pest management for sustainable rice cultivation: a holistic approach In: Sasaki T ed. Achieving Sustainable Cultivation of Rice—Cultivation, Pest and Disease Management. Burleigh-Dodds, Oxford, UK, pp 309–342.

[5] Horgan FG (2012) Diversity and defence: plant–herbivore interactions at multiple scales and trophic levels In: Gurr GM, Wratten SD, Snyder WE eds. Biodiversity and Insect Pests: Key Issues for Sustainable Management. Wiley-Blackwell, Oxford, UK, pp 241–258.

[6] Romena A, Heinrichs E (1989) Wild rices *Oryza* spp. as sources of resistance to rice insects. Journal of Plant Protection in the Tropics 6: 213–221.

[7] Heinrichs EA, Camanag E, Romena A (1985) Evaluation of rice cultivars for resistance to *Cnaphalocrocis medinalis* Guenée (Lepidoptera: Pyralidae). Journal of Economic Entomology 78: 274–278.

[8] Romena AM, Rapusas HR, Heinrichs EA (1986) Evaluation of rice accessions for resistance to the whitebacked planthopper *Sogatella furcifera* (Horváth) (Homoptera: Delphacidae). Crop Protection 5: 334–340.

[9] Velusamy R, Heinrichs EA, Medrano FG (1986) Greenhouse techniques to identify field resistance to the brown planthopper, *Nilaparvata lugens* (Stål) (Homoptera: Delphacidae), in rice cultivars. Crop Protection 5: 328–333.

[10] Widawsky D, Rozelle S, Jin S, Huang J (1998) Pesticide productivity, host-plant resistance and productivity in China. Agricultural Economics 19: 203–217.

[11] Horgan FG, Srinivasan TS, Bentur JS, Kumar R, Bhanu KV, et al (2017) Geographic and research center origins of rice resistance to Asian planthoppers and leafhoppers: implications for rice breeding and gene deployment. Agronomy 7: 62. 10.3390/agronomy7040062 PMC7371011 32704393

[12] Fahlgren N, Gehan MA, Baxter I (2015) Lights, camera, action: high-throughput plant phenotyping is ready for a close-up. Current Opinion in Plant Biology 24: 93–99. 10.1016/j.pbi.2015.02.006 25733069

[13] Goggin FL, Lorence A, Topp CN (2015) Applying high-throughput phenotyping to plant–insect interactions: picturing more resistant crops. Current Opinion in Insect Science 9: 69–76. 10.1016/j.cois.2015.03.002 32846711

[14] Sakamoto T, Gitelson AA, Nguy-Robertson AL, Arkebauer TJ, Wardlow BD, et al (2012) An alternative method using digital cameras for continuous monitoring of crop status. Agricultural and Forest Meteorology 154–155: 113–126.

[15] Saberioon MM, Amin MSM, Gholizadeh A, Ezri MH (2014) A review of optical methods for assessing nitrogen contents during rice growth. Applied Engineering in Agriculture 30: 657–669.

[16] Bock C, Poole G, Parker P, Gottwald T (2010) Plant disease severity estimated visually, by digital photography and image analysis, and by hyperspectral imaging. Critical Reviews in Plant Sciences 29: 59–107.

[17] Gitelson AA, Kaufman YJ, Stark R, Rundquist D (2002) Novel algorithms for remote estimation of vegetation fraction. Remote Sensing of Environment 80: 76–87.

[18] Sahurkar S, Chilke B (2017) Assessment of chlorophyll and nitrogen contents of leaves using image processing technique. International Research Journal of Engineering and Technology 4: 2243–2247.

[19] Karcher DE, Richardson MD (2003) Quantifying turfgrass color using digital image analysis. Crop Science 43: 943–951.

[20] Karcher DE, Richardson MD (2005) Batch analysis of digital images to evaluate turfgrass characteristics. Crop Science 45: 1536–1539.

[21] Sanseechan P, Saengprachathanarug K, Posom J, Wongpichet S, Chea C, et al (2019) Use of vegetation indices in monitoring sugarcane white leaf disease symptoms in sugarcane field using multispectral UAV aerial imagery. IOP Conference Series: Earth and Environmental Science 301: 012025.

[22] Mendoza F, Dejmek P, Aguilera JM (2006) Calibrated color measurements of agricultural foods using image analysis. Postharvest Biology and Technology 41: 285–295.

[23] Confalonieri R, Paleari L, Movedi E, Pagani V, Orlando F, et al (2015) Improving in vivo plant nitrogen content estimates from digital images: trueness and precision of a new approach as compared to other methods and commercial devices. Biosystems Engineering 135: 21–30.

[24] Lee K-J, Lee B-W (2013) Estimation of rice growth and nitrogen nutrition status using color digital camera image analysis. European Journal of Agronomy 48: 57–65.

[25] Li Y, Chen D, Walker CN, Angus JF (2010) Estimating the nitrogen status of crops using a digital camera. Field Crops Research 118: 221–227.

[26] Yadav SP, Ibaraki Y, Dutta Gupta S (2010) Estimation of the chlorophyll content of micropropagated potato plants using RGB based image analysis. Plant Cell, Tissue and Organ Culture 100: 183–188.

[27] Jannoura R, Brinkmann K, Uteau D, Bruns C, Joergensen RG (2015) Monitoring of crop biomass using true colour aerial photographs taken from a remote controlled hexacopter. Biosystems Engineering 129: 341–351.

[28] Rebetzke G, Jimenez-Berni J, Fischer R, Deery D, Smith D (2019) High-throughput phenotyping to enhance the use of crop genetic resources. Plant Science 282: 40–48. 10.1016/j.plantsci.2018.06.017 31003610

[29] Nijland W, de Jong R, de Jong SM, Wulder MA, Bater CW, et al (2014) Monitoring plant condition and phenology using infrared sensitive consumer grade digital cameras. Agricultural and Forest Meteorology 184: 98–106.

[30] Lee K-J, Lee B-W (2011) Estimating canopy cover from color digital camera image of rice field. Journal of Crop Science and Biotechnology 14: 151–155.

[31] Clément A, Verfaille T, Lormel C, Jaloux B (2015) A new colour vision system to quantify automatically foliar discolouration caused by insect pests feeding on leaf cells. Biosystems Engineering 133: 128–140.

[32] Hargrove WW, Crossley DA (1988) Video digitizer for the rapid measurement of leaf area lost to herbivorous insects. Annals of the Entomological Society of America 81: 593–598.

[33] Stone C, Coops NC (2004) Assessment and monitoring of damage from insects in Australian eucalypt forests and commercial plantations. Australian Journal of Entomology 43: 283–292.

[34] Yang C-M, Cheng C-H, Chen R-K (2007) Changes in spectral characteristics of rice canopy infested with brown planthopper and leaffolder. Crop Science 47: 329–335.

[35] Wu X, Cheng Q (2010) Study on the spectral characteristics of the damaged rice under brown planthopper, Nilaparvata lugens. Proceedings of the SPIE 7857, Multispectral, Hyperspectral, and Ultraspectral Remote Sensing Technology, Techniques, and Applications III, Incheon, South Korea, 785716, 10.1117/12.869427.

[36] Prabhakar M, Prasad Y, Desai S, Thirupathi M (2013) Spectral and spatial properties of rice brown plant hopper and groundnut late leaf spot disease infestation under field conditions. Journal of Agrometeorology 15: 57–62.

[37] Zhou Z, Zang Y, Luo X, Wang P (2011) Color-based corner detection algorithm for rice plant-hopper infestation area on rice stem using the RGB color space. American Society of Agricultural and Biological Engineers, Louisville, Kentucky, USA, 1111374. 10.13031/2013.37803)

[38] Prasannakumar N, Chander S, Sahoo R (2013) Spectral signatures of rice crop damaged by brown planthopper under field and glass house conditions. Current Biotica 7: 124–133.

[39] Huang J-R, Sun J-Y, Liao H-J, Liu X-D (2015) Detection of brown planthopper infestation based on SPAD and spectral data from rice under different rates of nitrogen fertilizer. Precision Agriculture 16: 148–163.

[40] Rubia-Sanchez E, Suzuki Y, Miyamoto K, Watanabe T (1999) The potential for compensation of the effects of the brown planthopper *Nilaparvata lugens* Stål (Homoptera: Delphacidae) feeding on rice. Crop Protection 18: 39–45.

[41] Watanabe T, Kitagawa H (2000) Photosynthesis and translocation of assimilates in rice plants following phloem feeding by the planthopper *Nilaparvata lugens* (Homoptera: Delphacidae). Journal of Economic Entomology 93: 1192–1198. 10.1603/0022-0493-93.4.1192 10985030

[42] Pagola M, Ortiz R, Irigoyen I, Bustince H, Barrenechea E, et al (2009) New method to assess barley nitrogen nutrition status based on image colour analysis: comparison with SPAD-502. Computers and Electronics in Agriculture 65: 213–218.

[43] Widjaja Putra BT, Soni P (2018) Enhanced broadband greenness in assessing chlorophyll a and b, carotenoid, and nitrogen in *Robusta* coffee plantations using a digital camera. Precision Agriculture 19: 238–256.

[44] Horgan FG, Crisol E (2013) Hybrid rice and insect herbivores in Asia. Entomologia Experimentalis et Applicata 148: 1–19.

[45] Horgan FG, Ramal AF, Bentur JS, Kumar R, Bhanu KV, et al (2015) Virulence of brown planthopper (*Nilaparvata lugens*) populations from South and South East Asia against resistant rice varieties. Crop Protection 78: 222–231.

[46] Qiu Y, Guo J, Jing S, Tang M, Zhu L, et al (2011) Identification of antibiosis and tolerance in rice varieties carrying brown planthopper resistance genes. Entomologia Experimentalis et Applicata 141: 224–231.

[47] Bhanu KV, Lakshmi VJ, Katti G, Reddy AV (2014) Antibiosis and tolerance mechanisms of resistance in rice varieties carrying brown planthopper resistance genes. Asian Journal of Biological and Life Sciences 3: 108–113.

[48] Bottrell DG, Schoenly KG (2012) Resurrecting the ghost of green revolutions past: the brown planthopper as a recurring threat to high-yielding rice production in tropical Asia. Journal of Asia-Pacific Entomology 15: 122–140.

[49] Backus EA, Serrano MS, Ranger CM (2005) Mechanisms of hopperburn: an overview of insect taxonomy, behavior, and physiology. Annual Review of Entomology 50: 125–151. 10.1146/annurev.ento.49.061802.123310 15471532

[50] Horgan FG, Srinivasan TS, Naik BS, Ramal AF, Bernal CC, et al (2016) Effects of nitrogen on egg-laying inhibition and ovicidal response in planthopper-resistant rice varieties. Crop Protection 89: 223–230. 10.1016/j.cropro.2016.07.033 27812236 PMC5026402

[51] Panda N, Heinrichs EA (1983) Levels of tolerance and antibiosis in rice varieties having moderate resistance to the brown planthopper, *Nilaparvata lugens* (Stål) (Hemiptera: Delphacidae). Environmental Entomology 12: 1204–1214.

[52] Ho DT, Heinrichs EA, Medrano F (1982) Tolerance of the rice variety Triveni to the brown planthopper, *Nilaparvata lugens*. Environmental Entomology 11: 598–602.

[53] Hunt ER, Daughtry C, Eitel JU, Long DS (2011) Remote sensing leaf chlorophyll content using a visible band index. Agronomy Journal 103: 1090–1099.

[54] Tucker CJ (1978) Red and photographic infrared linear combinations for monitoring vegetation. North Atlantic Space Administration, Goodard Flight Center, Greenbelt, MD, USA.

[55] Carlson TN, Ripley DA (1997) On the relation between NDVI, fractional vegetation cover, and leaf area index. Remote Sensing of Environment 62: 241–252.

[56] Louhaichi M, Borman MM, Johnson DE (2001) Spatially located platform and aerial photography for documentation of grazing impacts on wheat. Geocarto International 16: 65–70.

[57] Anderson MJ (2001) A new method for non-parametric multivariate analysis of variance. Austral Ecology 26: 32–46.

[58] Anderson MJ, Willis TJ (2003) Canonical analysis of principal coordinates: a useful method of constrained ordination for ecology. Ecology 82: 511–525.

[59] Boyer M, Miller J, Belanger M, Hare E, Wu J (1988) Senescence and spectral reflectance in leaves of northern pin oak (*Quercus palustris* Muenchh.). Remote Sensing of Environment 25: 71–87.

[60] Cook AG, Perfect TJ (1985) The influence of immigration on population development of *Nilaparvata lugens* and *Sogatella furcifera* and its interaction with immigration by predators. Crop Protection 4: 423–433.

[61] Sogawa K, Liu G, Shen J (2003) A review on the hyper-susceptibility of Chinese hybrid rice to insect pests. Chinese Journal of Rice Science 17: 23–30.

[62] Sogawa K, Qian Q, Da-li Z, Jiang H, Long-jun Z (2005) Differential expression of whitebacked planthopper resistance in the japonica/indica double haploid rice population under field evaluation and seedbox screening test. Rice Science 12: 63–67.

[63] Yun S, Lee G-H, Kim K-M (2016) Optimum screening time for improved WBPH-associated QTL analysis in rice. International Journal of Agriculture and Biology 18: 844–850.

[64] Silva ER, Lazarotto DC, Schwambach J, Overbeck GE, Soares GLG (2017) Phytotoxic effects of extract and essential oil of *Eucalyptus saligna* (Myrtaceae) leaf litter on grassland species. Australian Journal of Botany 65: 172–182.

[65] Dias T, Crous CJ, Liberati D, Munzi S, Gouveia C, et al (2017) Alleviating nitrogen limitation in Mediterranean maquis vegetation leads to ecological degradation. Land Degradation and Development 28: 2482–2492.

[66] Leimu R, Koricheva J (2006) A meta-analysis of tradeoffs between plant tolerance and resistance to herbivores: combining the evidence from ecological and agricultural studies. Oikos 112: 1–9.

[67] Wise MJ, Carr DE (2008) On quantifying tolerance of herbivory for comparative analyses Evolution 62: 2429–2434. 10.1111/j.1558-5646.2008.00458.x 18637836

[68] Horgan FG, Crisol-Martínez E, Almazan MLP, Romena A, Ramal AF, et al (2016) Susceptibility and tolerance in hybrid and pure-line rice varieties to herbivore attack: biomass partitioning and resource-based compensation in response to damage. Annals of Applied Biology 169: 200–213.

[69] Rubia E, Shepard B, Yambao E, Ingram K, Arida G, et al (1989) Stem borer damage and grain yield of flooded rice. Journal of Plant Protection in the Tropics 6: 205–211.

[70] Yao Q, Xian D-x, Liu Q-j, Yang B-j, Diao G-q, et al (2014) Automated counting of rice planthoppers in paddy felds based on image processing. Journal of Integrative Agriculture 13: 1736–1745.

[71] Yao Q, Chen G-t, Wang Z, Zhang C, Yang B-j, et al (2017) Automated detection and identification of white-backed planthoppers in paddy fields using image processing. Journal of Integrative Agriculture 16: 1547–1557.

[72] Liu T, Chen W, Wu W, Sun C, Guo W, et al (2016) Detection of aphids in wheat fields using a computer vision technique. Biosystems Engineering 141: 82–93.

[73] Mongkolchart N, Ketcham M. (2014) The measurement of brown planthopper by image processing. International Conference on Advanced Computiotional Technologies and Creative Media. Pattaya, Thailand, pp 102–105.

[74] Xu S, Zhou Z, Lu H, Luo X, Lan Y, et al (2014) Estimation of the age and amount of brown rice plant hoppers based on bionic electronic nose use. Sensors 14: 18114–18130. 10.3390/s141018114 25268913 PMC4239905

[75] Zhou B, Wang J (2011) Discrimination of different types of damage of rice plants by electronic nose. Biosystems Engineering 109: 250–257.

[76] Horgan FG, Peñalver Cruz A, Bernal CC, Ramal AF, Almazan MLP, et al (2018) Resistance and tolerance to the brown planthopper, *Nilaparvata lugens* (Stål), in rice infested at different growth stages across a gradient of nitrogen applications. Field Crops Research 217: 53–65. 10.1016/j.fcr.2017.12.008 29503500 PMC5777095

