# Supplementary material for: Changes in reflectance of rice seedlings during planthopper feeding as detected by digital camera: Potential applications for high-throughput phenotyping
Source: PLoS One. 2020 Aug 27;15(8):e0238173. doi: 10.1371/journal.pone.0238173 (PMC7451558; doi:10.1371/journal.pone.0238173)
Supplement: S1 Fig — (DOCX) [file pone.0238173.s001.docx]

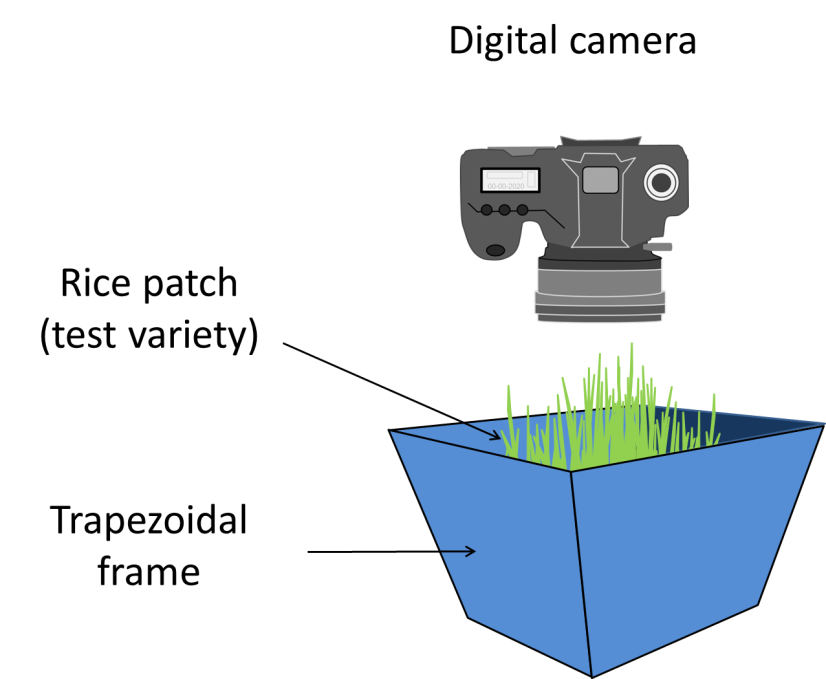


**Fig S1. Diagram of inverted trapezoidal frame** (with blue inside surface) placed over a rice patch during the adapted-Standard Seedling Seed-box Test during image capture using a digital camera.
