# Supplementary material for: Changes in reflectance of rice seedlings during planthopper feeding as detected by digital camera: Potential applications for high-throughput phenotyping
Source: PLoS One. 2020 Aug 27;15(8):e0238173. doi: 10.1371/journal.pone.0238173 (PMC7451558; doi:10.1371/journal.pone.0238173)
Supplement: S2 Fig — (DOCX) [file pone.0238173.s002.docx]

**Fig S2. Comparisons of raw values and derived indices representing control, non-infested TN1 seedlings (green lines and symbols), brown planthopper-infested TN1 seedlings (brown lines and symbols) and whitebacked planthopper-infested TN1 seedlings (blue lines and symbols).** Indicators are as follows: A, normalized red (r), B, normalized green (g), C, normalized blue (b), D, Hue, E, Saturation, F, GMR, G, GDR, H, NGRDI, I, VARI, J, GLI, K, TGI, and L, DGCI. Mean red, green and blue reflectance are indicated in Figure 1 and NGRDI, GLI and DGCI (inverse) appear again in Figure 2. Standard errors are indicated. Results for repeated measures GLMs are indicated as time (T), treatment (H), and time*treatment interaction (T*H). *** = P ≤ 0.001, * = P ≤ 0.05, ns = P > 0.05 (N = 3). F-values are presented in Table S2.
