# Supplementary material for: Changes in reflectance of rice seedlings during planthopper feeding as detected by digital camera: Potential applications for high-throughput phenotyping
Source: PLoS One. 2020 Aug 27;15(8):e0238173. doi: 10.1371/journal.pone.0238173 (PMC7451558; doi:10.1371/journal.pone.0238173)
Supplement: S3 Fig — (DOCX) [file pone.0238173.s003.docx]

**Figure S3: Correlation matrix indicating correlations between a range of greenness indices for 38 rice varieties exposed to brown planthopper (brown symbols) or whitebacked planthopper (blue symbols).** Numbers are Pearson Correlation Coefficients (all P-values ≤ 0.005).
