# Supplementary material for: Changes in reflectance of rice seedlings during planthopper feeding as detected by digital camera: Potential applications for high-throughput phenotyping
Source: PLoS One. 2020 Aug 27;15(8):e0238173. doi: 10.1371/journal.pone.0238173 (PMC7451558; doi:10.1371/journal.pone.0238173)
Supplement: S4 Fig — (DOCX) [file pone.0238173.s004.docx]

**Fig S4. Results for damage relative to TN1 from three runs of the Standard Seedlin Seed-box test for phenotyping of rice for resistance to the brown planthopper.** Graphs indicate the relative values of GLI_test_-GLI_TN1_ for 36 rice varieties (A-AJ) using the Laguna-planthopper population. Varieties are ordered according to rank resistance from the highest resistance (A) to the least resistance (AJ).
