# Supplementary material for: Changes in reflectance of rice seedlings during planthopper feeding as detected by digital camera: Potential applications for high-throughput phenotyping
Source: PLoS One. 2020 Aug 27;15(8):e0238173. doi: 10.1371/journal.pone.0238173 (PMC7451558; doi:10.1371/journal.pone.0238173)
Supplement: S5 Fig — (DOCX) [file pone.0238173.s005.docx]

**Fig S5. Results for condition change relative to TN1 from three runs of the Standard Seedling Seed-box Test for phenotyping of rice for resistance to the brown planthopper**. Graphs indicate the relative proportional changes in GLI between infested and control plants for each test variety (A-AJ) relative to the susceptible control variety TN1. Varieties are ordered according to rank resistance from the highest resistance (A) to the least resistance (AJ).
