# Supplementary material for: Changes in reflectance of rice seedlings during planthopper feeding as detected by digital camera: Potential applications for high-throughput phenotyping
Source: PLoS One. 2020 Aug 27;15(8):e0238173. doi: 10.1371/journal.pone.0238173 (PMC7451558; doi:10.1371/journal.pone.0238173)
Supplement: S8 Fig — (DOCX) [file pone.0238173.s008.docx]

**Fig S8. Mean values (± SEM) for A, luminosity, B, mean red reflectance, C, mean green reflectance, D, mean blue reflectance, and E, derived GLI from digital images of TN1 seedlings reared under different test conditions.** Graphs are divided between images taken without a flash or with a flash (as indicated). Plants are ordered according to age (8 days or 14 days) and planting density (1 cm^-2^ or 2 cm^-2^) as indicated. Images were captured using black (grey symbols) or blue (blue symbols) trapezoidal funnels. For further details see Table S7 and Table S8
