# Supplementary material for: Changes in reflectance of rice seedlings during planthopper feeding as detected by digital camera: Potential applications for high-throughput phenotyping
Source: PLoS One. 2020 Aug 27;15(8):e0238173. doi: 10.1371/journal.pone.0238173 (PMC7451558; doi:10.1371/journal.pone.0238173)
Supplement: S1 Table — (DOCX) [file pone.0238173.s009.docx]

**Table S1 Damage scores for brown and whitebacked planthoppers in Standard Seedbox Screening Tests (SSSTs)^1^ according to the Standard Evaluation System for rice**

| Damage score | Damage |
| --- | --- |
| 0 | No damage |
| 1 | Very slight damage |
| 3 | First and 2^nd^ leaves of most plants partially yellowing |
| 5 | Pronounced yellowing and stunting or about 10 to 25% of the plants wilting or dead and remaining plants severely stunted or dying |
| 7 | More than half of the plants dead, stunted or both; remaining plants severely stunted or wilted |
| 9 | All plants dead |

1: The SSST is a rapid screening test to evaluate interactions between rice seedlings and insect herbivores. In the test, large trays (≥ 60 × 40 × 40 cm, length × width × height) and with 3 cm (deep) of paddy soil, are planted with test varieties and a susceptible control (often TN1). About 30-40 seed of each variety are sown directly to the trays in lines. After 7 days, seedlings in each line are thinned to 25 per line. The lines are then infested with 1^st^ or 2^nd^ instar planthopper nymphs at a rate of 8 per seedling. The nymphs are allowed to feed and develop until the susceptible control variety dies, i.e., damage score for control = 9. At that time, the test is completed and all test varieties are evaluated receiving a damage score. Researchers can vary aspects of the test, but tests must include rice at the seedling stage and nymphs at a density of ca 8/seedling.
