# Supplementary material for: Changes in reflectance of rice seedlings during planthopper feeding as detected by digital camera: Potential applications for high-throughput phenotyping
Source: PLoS One. 2020 Aug 27;15(8):e0238173. doi: 10.1371/journal.pone.0238173 (PMC7451558; doi:10.1371/journal.pone.0238173)
Supplement: S2 Table — (DOCX) [file pone.0238173.s010.docx]

**Table S2 Results of repeated measures GLM for colour and index responses to control, brown planthopper-infested and whitebacked planthopper-infested TN1 plants in Standard Seedling Seed-box Tests**

| Index | Sources of variation | |  |
| --- | --- | --- | --- |
|  | Within subject effects | | Between subject effects |
|  | Time | Time*treatment | Treatment |
| Red reflectance | 21.283*** | 1.124 | 20.872*** |
| Green reflectance | 2.818** | 0.684 | 1.064 |
| Blue reflectance | 30.821*** | 9.268*** | 72.023*** |
| Normalized r | 15.793*** | 0.653 | 2.207 |
| Normalized g | 69.868*** | 23.462*** | 51.441*** |
| Normalized b | 10.925*** | 9.879*** | 51.056*** |
| Hue | 33.038*** | 4.744*** | 8.229* |
| Saturation | 35.224*** | 18.456*** | 50.506*** |
| Brightness | 3.471** | 0.397 | 0.617 |
| GMR | 76.760*** | 15.995*** | 20.612*** |
| DGDR | 66.421*** | 7.073*** | 38.205*** |
| NGRDI | 72.597*** | 9.139*** | 31.739*** |
| VARI | 57.524*** | 6.091*** | 24.525*** |
| GLI | 91.247*** | 27.493*** | 47.401*** |
| TGI | 34.577*** | 17.975*** | 24.816*** |
| DGCI | 7.330*** | 7.412*** | 27.467*** |
| DF | 9 | 18 | 2 |
| Error DF | 54 | 54 | 6 |
