# Supplementary material for: Changes in reflectance of rice seedlings during planthopper feeding as detected by digital camera: Potential applications for high-throughput phenotyping
Source: PLoS One. 2020 Aug 27;15(8):e0238173. doi: 10.1371/journal.pone.0238173 (PMC7451558; doi:10.1371/journal.pone.0238173)
Supplement: S3 Table — (DOCX) [file pone.0238173.s011.docx]

**Table S3. Results of Standard Seedling Seed-box Tests for 37 rice varieties exposed to the brown planthopper** (numbers are means ± SEM)

| Variety | GLI^1^ | GLI infested - GLI control^1^ | GLI (infested/control)Test/(infested/control)TN1^2^ | SSST^1^ | Seedling weight loss^1^ |
| --- | --- | --- | --- | --- | --- |
| Rathu Heenati | 0.185±0.010*** | 0.037±0.026*** | 30.275±4.044^e^ | 3.667±0.667*** | 0.567±0.121 |
| MOI | 0.163±0.015*** | 0.064±0.008*** | 27.609±7.766^de^ | 5.000±0.000*** | 0.420±0.044 |
| PTB33 | 0.164±0.005*** | 0.055±0.017*** | 28.005±5.693^de^ | 5.000±1.155*** | 0.427±0.158 |
| ADR52 | 0.118±0.063* | 0.114±0.072 | 15.282±7.169^abcde^ | 5.667±1.764* | 0.673±0.103 |
| IR56 | 0.135±0.041* | 0.115±0.046 | 17.546±2.926^abcde^ | 5.667±0.667* | 0.537±0.110 |
| IR62 | 0.157±0.025** | 0.077±0.024** | 23.730±2.437^bcde^ | 5.667±0.667* | 0.273±0.002 |
| IR65482-4-136-2-2 | 0.156±0.046*** | 0.099±0.036* | 23.203±9.215^abcde^ | 5.667±0.667* | 0.707±0.220 |
| IR71033 | 0.131±0.043* | 0.119±0.027 | 18.999±6.815^abcde^ | 5.667±0.667* | 0.491±0.094 |
| IR74 | 0.141±0.017* | 0.092±0.016* | 22.495±4.824^abcde^ | 5.667±0.667* | 0.486±0.036 |
| Babawee | 0.120±0.028* | 0.115±0.038 | 17.498±2.586^abcde^ | 6.333±0.667 | 0.567±0.108 |
| Balamawee | 0.150±0.013** | 0.081±0.004** | 24.803±6.749^cde^ | 6.333±0.667 | 0.645±0.126 |
| IR64 | 0.130±0.024* | 0.128±0.053 | 20.137±6.094^abcde^ | 6.333±0.667 | 0.654±0.031 |
| IR24 | 0.114±0.045 | 0.133±0.040 | 14.861±2.311^abcde^ | 7.000±1.155 | 0.533±0.033 |
| Pokkali | 0.077±0.039 | 0.143±0.033 | 9.838±3.776^abcde^ | 7.000±1.155 | 1.240±0.220* |
| Swarnalata | 0.118±0.025* | 0.119±0.026 | 18.049±4.373^abcde^ | 7.000±0.000 | 0.490±0.221 |
| Triveni | 0.086±0.053 | 0.196±0.059 | 8.347±4.973^abcde^ | 7.000±1.155 | 0.574±0.064 |
| ARC6650 | 0.094±0.049 | 0.122±0.059 | 12.826±6.011^abcde^ | 7.667±0.667 | 0.436±0.081 |
| IR60 | 0.040±0.009 | 0.215±0.012 | 5.490±0.907^abcd^ | 7.667±0.667 | 0.801±0.154 |
| IR70 | 0.034±0.037 | 0.208±0.036 | 3.384±3.762^abc^ | 7.667±0.667 | 0.633±0.110 |
| IR72 | 0.099±0.052 | 0.155±0.055 | 10.977±5.169^abcde^ | 7.667±0.667 | 0.764±0.246 |
| ARC10550 | 0.017±0.008 | 0.221±0.003 | 2.182±0.528^abc^ | 8.333±0.667 | 1.024±0.220 |
| ASD7 | 0.016±0.012 | 0.211±0.009 | 2.088±1.717^abc^ | 8.333±0.667 | 0.882±0.301 |
| Da Hua Gu | 0.037±0.031 | 0.184±0.049 | 5.607±3.974^abcd^ | 8.333±0.667 | 0.329±0.133 |
| IR65482-7-216-1-2-B | 0.024±0.014 | 0.215±0.016 | 3.802±1.905^abc^ | 8.333±0.667 | 0.809±0.156 |
| IR66 | 0.050±0.036 | 0.176±0.043 | 7.653±5.253^abcde^ | 8.333±0.667 | 0.429±0.070 |
| TN1 | 0.007±0.002 | 0.241±0.010 |  | 8.905±0.095 | 0.601±0.153 |
| ARC10239 | 0.003±0.002 | 0.217±0.016 | 0.375±0.411^ab^ | 9.000±0.000 | 0.763±0.098 |
| Asiminori | 0.023±0.021 | 0.222±0.012 | 2.127±1.962^abc^ | 9.000±0.000 | 0.562±0.098 |
| Chinsaba | 0.003±0.002 | 0.220±0.023 | 0.427±0.379^ab^ | 9.000±0.000 | 0.701±0.064 |
| IR22 | 0.012±0.007 | 0.222±0.020 | 1.508±0.708^abc^ | 9.000±0.000 | 0.688±0.072 |
| IR40 | 0.003±0.007 | 0.256±0.030 | 0.416±0.988^ab^ | 9.000±0.000 | 0.570±0.032 |
| Mudgo | 0.006±0.001 | 0.241±0.008 | 0.922±0.194^ab^ | 9.000±0.000 | 0.820±0.145 |
| N22 | 0.008±0.012 | 0.249±0.027 | 0.959±1.736^ab^ | 9.000±0.000 | 0.634±0.138 |
| N'Diang Marie | 0.001±0.001 | 0.255±0.016 | 0.094±0.202^a^ | 9.000±0.000 | 1.028±0.399 |
| T65 | 0.001±0.001 | 0.239±0.009 | 0.194±0.135^a^ | 9.000±0.000 | 0.640±0.140 |
| Utri Rajapan | 0.008±0.005 | 0.270±0.011 | 1.093±0.622^ab^ | 9.000±0.000 | 1.017±0.307 |
| Yagyaw | 0.001±0.001 | 0.260±0.008 | 0.090±0.180^a^ | 9.000±0.000 | 0.548±0.122 |
| F-variety^3^ 36/74 | 4.924*** | 4.358*** | 5.629*** | 5.158*** | 1.696* |

1: *** P ≤ 0.005, ** = P ≤ 0.01, * = P ≤ 0.05 (Duncan’s many-to-one comparisons)

2: Lowercase letters indicate homogenous groups (Tukey pairwise comparisons)

3: Nominator DF = 36, denominator DF = 74
