# Supplementary material for: Changes in reflectance of rice seedlings during planthopper feeding as detected by digital camera: Potential applications for high-throughput phenotyping
Source: PLoS One. 2020 Aug 27;15(8):e0238173. doi: 10.1371/journal.pone.0238173 (PMC7451558; doi:10.1371/journal.pone.0238173)
Supplement: S4 Table — (DOCX) [file pone.0238173.s012.docx]

**Table S4: Results of Standard Seedling Seed-box Tests for 37 rice varieties exposed to the whitebacked planthopper** (numbers are means ± SEM)

| Variety | GLI^1^ | GLI infested - GLI control^1^ | GLI (infested-control)Test/(infested-control)TN1^2^ | SSST^1^ | Seedling weight loss^1^ |
| --- | --- | --- | --- | --- | --- |
| Balamawee | 0.181±0.016* | 0.050±0.002 | 15.959±11.953 | 3.667±0.667*** | 0.340±0.059 |
| MOI | 0.156±0.010* | 0.071±0.017 | 12.367±8.521 | 4.333±1.333** | 0.152±0.284 |
| Pokkali | 0.157±0.011* | 0.063±0.012 | 15.041±11.303 | 4.333±0.667** | 0.816±0.042 |
| IR24 | 0.140±0.026 | 0.107±0.010 | 12.807±10.121 | 5.000±0.000* | 0.493±0.028 |
| Rathu Heenati | 0.135±0.007 | 0.088±0.009 | 11.882±8.635 | 5.000±1.155* | 0.737±0.188 |
| IR62 | 0.091±0.014 | 0.143±0.025 | 5.809±3.449 | 5.667±0.667 | 0.409±0.060 |
| IR66 | 0.134±0.043 | 0.092±0.048 | 7.176±3.092 | 5.667±0.667 | 0.192±0.077 |
| IR71033 | 0.140±0.029 | 0.111±0.017 | 13.459±10.945 | 5.667±0.667 | 0.518±0.082 |
| N22 | 0.140±0.036 | 0.117±0.046 | 7.194±3.496 | 5.667±1.764 | 0.282±0.042 |
| N'Diang Marie | 0.156±0.015* | 0.100±0.011 | 13.270±10.217 | 5.667±1.333 | 0.649±0.354 |
| ADR52 | 0.150±0.035 | 0.081±0.035 | 15.661±12.938 | 6.333±0.667 | 0.567±0.224 |
| Babawee | 0.087±0.023 | 0.148±0.021 | 5.590±3.256 | 6.333±0.667 | 0.653±0.142 |
| IR64 | 0.135±0.026 | 0.123±0.026 | 12.533±10.243 | 6.333±0.667 | 0.550±0.115 |
| IR65482-4-136-2-2 | 0.078±0.036 | 0.177±0.044 | 2.490±0.498 | 6.333±0.667 | 0.776±0.057 |
| IR72 | 0.134±0.006 | 0.120±0.009 | 10.025±7.095 | 6.333±0.667 | 0.672±0.177 |
| IR74 | 0.097±0.011 | 0.136±0.025 | 7.256±4.820 | 6.333±0.667 | 0.501±0.028 |
| Mudgo | 0.124±0.055 | 0.123±0.062 | 3.691±1.045 | 6.333±1.333 | 0.803±0.140 |
| ARC6650 | 0.098±0.021 | 0.118±0.010 | 9.383±6.917 | 7.000±0.000 | 0.429±0.112 |
| Chinsaba | 0.114±0.008 | 0.109±0.015 | 9.734±6.884 | 7.000±0.000 | 0.553±0.044 |
| IR56 | 0.117±0.027 | 0.133±0.021 | 8.283±5.455 | 7.000±0.000 | 0.527±0.100 |
| IR65482-7-216-1-2-B | 0.108±0.031 | 0.131±0.028 | 7.751±4.890 | 7.000±0.000 | 0.769±0.186 |
| IR70 | 0.090±0.049 | 0.152±0.054 | 2.569±1.250 | 7.000±1.155 | 0.561±0.107 |
| Swarnalata | 0.074±0.049 | 0.163±0.055 | 2.460±1.407 | 7.000±1.155 | 0.488±0.204 |
| Triveni | 0.083±0.052 | 0.199±0.056 | 2.280±1.199 | 7.000±1.155 | 0.598±0.145 |
| Yagyaw | 0.116±0.020 | 0.144±0.014 | 10.759±8.676 | 7.000±0.000 | 0.473±0.096 |
| ARC10239 | 0.069±0.029 | 0.151±0.035 | 3.056±0.965 | 7.667±0.667 | 0.621±0.103 |
| Asiminori | 0.108±0.060 | 0.137±0.055 | 2.424±1.580 | 7.667±0.667 | 0.551±0.146 |
| Da Hua Gu | 0.051±0.026 | 0.170±0.037 | 1.540±0.924 | 7.667±0.667 | 0.319±0.120 |
| IR22 | 0.076±0.033 | 0.157±0.050 | 2.333±0.341 | 7.667±0.667 | 0.589±0.052 |
| IR40 | 0.039±0.012 | 0.220±0.030 | 1.751±0.701 | 7.667±0.667 | 0.543±0.041 |
| IR60 | 0.073±0.019 | 0.182±0.017 | 6.975±5.886 | 7.667±0.667 | 0.729±0.153 |
| PTB33 | 0.065±0.033 | 0.154±0.038 | 1.919±0.834 | 7.667±0.667 | 0.580±0.363 |
| Utri Rajapan | 0.104±0.052 | 0.174±0.063 | 2.488±1.262 | 7.667±0.667 | 0.861±0.251 |
| ARC10550 | 0.053±0.008 | 0.185±0.013 | 5.496±4.440 | 8.333±0.667 | 1.023±0.248 |
| ASD7 | 0.035±0.014 | 0.192±0.009 | 1.319±0.302 | 8.333±0.667 | 0.907±0.441 |
| T65 | 0.061±0.033 | 0.179±0.042 | 1.299±0.641 | 8.333±0.667 | 0.587±0.087 |
| TN1 | 0.036±0.018 | 0.212±0.027 |  | 8.333±0.415 | 0.591±0.156 |
| F-value^3^ | 1.587* | 1.503 | 0.564 | 2.218*** | 1.309 |

1: *** P ≤ 0.005, ** = P ≤ 0.01, * = P ≤ 0.05 (Duncan’s many-to-one comparisons)

2: Lowercase letters indicate homogenous groups (Tukey pairwise comparisons)

3: Nominator DF = 36, denominator DF = 74
