# Supplementary material for: Changes in reflectance of rice seedlings during planthopper feeding as detected by digital camera: Potential applications for high-throughput phenotyping
Source: PLoS One. 2020 Aug 27;15(8):e0238173. doi: 10.1371/journal.pone.0238173 (PMC7451558; doi:10.1371/journal.pone.0238173)
Supplement: S5 Table — (DOCX) [file pone.0238173.s013.docx]

**Table S5: Results of permutational MANOVA** (see Fig. 4)

| Source of variation | DF | SS^1^ | MS | Pseudo-F | Unique P (perms) | Perms^2^ |
| --- | --- | --- | --- | --- | --- | --- |
| Treatment | 2 | 61.145 | 30.572 | 2.762 | 0.045 | 4723 |
| Time | 9 | 120.540 | 13.394 | 25.341 | 0.0001 | 9932 |
| Variety | 9 | 99.623 | 11.069 | 20.943 | 0.0001 | 9907 |
| Treatment*time | 18 | 32.879 | 1.826 | 3.456 | 0.0001 | 9865 |
| Residuals | 81 | 42.811 | 0.529 |  |  |  |
| Total | 119 | 357 |  |  |  |  |

1: Type III SS

2: 9999 permutations
