# Supplementary material for: Changes in reflectance of rice seedlings during planthopper feeding as detected by digital camera: Potential applications for high-throughput phenotyping
Source: PLoS One. 2020 Aug 27;15(8):e0238173. doi: 10.1371/journal.pone.0238173 (PMC7451558; doi:10.1371/journal.pone.0238173)
Supplement: S6 Table — (DOCX) [file pone.0238173.s014.docx]

**Table S6: Pairwise tests from permutational MANOVA** (see Fig. 4)

| Groups^1^ | t-statistic | P (perm) | P (MC) |
| --- | --- | --- | --- |
| *Time period = 1* |  |  |  |
| Brown planthopper, control | 0.756 | 0.597 | 0.529 |
| Brown planthopper, whitebacked planthopper | 0.681 | 0.498 | 0.588 |
| Control, whitebacked planthopper | 0.375 | 0.889 | 0.841 |
| *Time period = 2* |  |  |  |
| Brown planthopper, control | 2.166 | 0.060 | 0.067 |
| Brown planthopper, whitebacked planthopper | 1.042 | 0.419 | 0.367 |
| Control, whitebacked planthopper | 2.156 | 0.084 | 0.058 |
| *Time period = 3* |  |  |  |
| Brown planthopper, control | 1.294 | 0.322 | 0.245 |
| Brown planthopper, whitebacked planthopper | 1.931 | 0.088 | 0.079 |
| Control, whitebacked planthopper | 0.593 | 0.668 | 0.690 |
| *Time period = 4* |  |  |  |
| Brown planthopper, control | 1.519 | 0.157 | 0.123 |
| Brown planthopper, whitebacked planthopper | 1.370 | 0.253 | 0.203 |
| Control, whitebacked planthopper | 1.035 | 0.416 | 0.376 |
| *Time period = 5* |  |  |  |
| Brown planthopper, control | 1.752 | 0.052 | 0.061 |
| Brown planthopper, whitebacked planthopper | 0.705 | 0.645 | 0.623 |
| Control, whitebacked planthopper | 1.664 | 0.06 | 0.101 |
| *Time period = 6* |  |  |  |
| Brown planthopper, control | 2.77 | 0.025 | 0.018 |
| Brown planthopper, whitebacked planthopper | 0.997 | 0.391 | 0.389 |
| Control, whitebacked planthopper | 2.368 | 0.031 | 0.033 |
| Time period = 7 |  |  |  |
| Brown planthopper, control | 2.159 | 0.029 | 0.043 |
| Brown planthopper, whitebacked planthopper | 0.616 | 0.734 | 0.666 |
| Control, whitebacked planthopper | 1.864 | 0.032 | 0.063 |
| *Time period = 8* |  |  |  |
| Brown planthopper, control | 2.226 | 0.032 | 0.022 |
| Brown planthopper, whitebacked planthopper | 0.599 | 0.719 | 0.702 |
| Control, whitebacked planthopper | 2.062 | 0.017 | 0.041 |
| *Time period = 9* |  |  |  |
| Brown planthopper, control | 2.164 | 0.029 | 0.035 |
| Brown planthopper, whitebacked planthopper | 0.684 | 0.738 | 0.640 |
| Control, whitebacked planthopper | 2.326 | 0.054 | 0.042 |
| *Time period = 10* |  |  |  |
| Brown planthopper, control | 2.870 | 0.024 | 0.010 |
| Brown planthopper, whitebacked planthopper | 0.742 | 0.631 | 0.566 |
| Control, whitebacked planthopper | 2.734 | 0.03 | 0.015 |

1: Nominator DF = 1, denominator = 6
