# Supplementary material for: Changes in reflectance of rice seedlings during planthopper feeding as detected by digital camera: Potential applications for high-throughput phenotyping
Source: PLoS One. 2020 Aug 27;15(8):e0238173. doi: 10.1371/journal.pone.0238173 (PMC7451558; doi:10.1371/journal.pone.0238173)
Supplement: S7 Table — (DOCX) [file pone.0238173.s015.docx]

**Table S7: Results of univariate GLM for effects of test and light conditions on colour space parameters**

| Sources of variation | Luminosity^1^ | Mean R^1^ | Mean G^1^ | Mean B^1^ | Chroma^1^ | Hue^1^ | Saturation^1^ | Brightness^1^ |
| --- | --- | --- | --- | --- | --- | --- | --- | --- |
| Plant age (T) | 530.201*** | 625.978*** | 459.591*** | 154.447*** | 1056.97*** | 692.386*** | 867.750*** | 460.36*** |
| Box color (B) | 1.435 | 26.014*** | 0.213 | 113.126*** | 55.296*** | 245.105*** | 170.125*** | 0.250 |
| Flash (F) | 3.939* | 0.750 | 5.894* | 319.236*** | 88.951*** | 7.455** | 263.773*** | 5.999* |
| Size (S) | 0.504 | 1.362 | 1.240 | 9.157*** | 9.821*** | 6.586*** | 15.725*** | 1.234 |
| Density (D) | 48.091*** | 22.365*** | 34.556*** | 269.002*** | 17.362*** | 69.829*** | 212.106*** | 34.789*** |
| Distribution (R) | 6.201** | 10.156*** | 5.815* | 0.155 | 8.374*** | 16.237*** | 5.969* | 5.519* |
| T*B | 1.638 | 2.347 | 1.915 | 3.407 | 0.011 | 27.580*** | 0.058 | 2.029 |
| T*F | 219.000*** | 349.451*** | 351.277*** | 25.751*** | 598.953*** | 165.080*** | 321.467*** | 354.166*** |
| T*S | 2.615 | 2.077 | 1.142 | 10.233*** | 1.495 | 1.540 | 5.427*** | 1.104 |
| T*D | 0.293 | 0.057 | 0.074 | 1.836 | 1.982 | 13.522*** | 0.215 | 0.076 |
| T*R | 0.034 | 0.901 | 0.571 | 0.044 | 0.886 | 6.581** | 2.001 | 0.490 |
| B*F | 111.577*** | 78.176*** | 127.050*** | 161.202*** | 19.057*** | 9.708** | 27.092*** | 126.205*** |
| B*S | 1.136 | 1.706 | 1.313 | 1.757 | 0.333 | 2.933*** | 0.667 | 1.328 |
| B*D | 0.31 | 3.197 | 0.81 | 4.424* | 4.904* | 20.376*** | 3.807* | 0.660 |
| B*R | 0.49 | 1.69 | 0.891 | 0.032 | 1.498 | 6.468** | 0.893 | 0.926 |
| F*S | 3.507** | 1.353 | 2.256 | 0.316 | 3.847*** | 0.523 | 1.367 | 2.271 |
| F*D | 0.639 | 5.457* | 5.132* | 32.184*** | 39.208*** | 30.062*** | 71.362*** | 5.003* |
| F*R | 0.247 | 0.979 | 0.012 | 1.057 | 0.552 | 5.499** | 1.445 | 0.004 |
| S*D | 1.310 | 1.606 | 1.576 | 1.110 | 2.182 | 1.839 | 2.012 | 1.492 |
| S*R | 0.741 | 0.286 | 0.662 | 1.008 | 0.17 | 0.238 | 0.586 | 0.633 |
| D*R | 0.393 | 1.965 | 1.472 | 0.088 | 1.826 | 2.242 | 0.770 | 1.409 |
| T*B*F | 13.342*** | 23.204*** | 21.083*** | 5.495** | 14.009*** | 0.466 | 0.770 | 20.576*** |
| T*B*S | 1.709 | 2.164 | 2.363 | 1.351 | 2.443* | 2.234 | 2.165 | 2.339* |
| T*B*D | 0.84 | 1.634 | 0.925 | 0.143 | 0.362 | 7.040** | 0.043 | 0.832 |
| T*B*R | 0.101 | 0.026 | 0.096 | 0.236 | 0.076 | 3.34 | 0.167 | 0.098 |
| T*F*S | 2.633 | 1.237 | 1.847 | 1.126 | 2.608* | 0.866 | 0.87 | 1.844 |
| T*F*D | 0.597 | 0.002 | 0.456 | 0.001 | 1.082 | 14.764*** | 1.047 | 0.507 |
| T*F*R | 0.065 | 0.011 | 0.032 | 0.457 | 0.617 | 0.560 | 0.399 | 0.026 |
| T*S*D | 3.271** | 2.198 | 2.824* | 3.222** | 2.331 | 1.988 | 3.129** | 2.761* |
| T*S*R | 0.576 | 0.582 | 0.481 | 0.802 | 0.191 | 0.482 | 0.490 | 0.539 |
| T*D*R | 0.001 | 0.034 | 0.039 | 1.372 | 1.234 | 0.116 | 1.611 | 0.083 |
| B*F*S | 1.641 | 2.058 | 1.822 | 0.517 | 1.405 | 1.205 | 0.788 | 1.887 |
| B*F*D | 0.601 | 0.310 | 1.28 | 3.471 | 0.076 | 3.628 | 0.004 | 1.155 |
| B*F*R | 0.033 | 0.104 | 0.024 | 0.001 | 0.018 | 0.982 | 0.009 | 0.002 |
| B*S*D | 0.212 | 0.425 | 0.205 | 0.165 | 0.523 | 0.862 | 0.414 | 0.185 |
| B*S*R | 0.387 | 0.518 | 0.687 | 1.181 | 0.194 | 0.95 | 0.798 | 0.709 |
| B*D*R | 0.455 | 0.591 | 0.644 | 0.379 | 0.362 | 0.053 | 0.001 | 0.6 |
| F*S*D | 1.069 | 0.287 | 0.504 | 1.686 | 0.073 | 0.955 | 1.704 | 0.51 |
| F*S*R | 1.176 | 0.690 | 1.031 | 3.156** | 0.99 | 2.665 | 3.507** | 1.115 |
| F*D*R | 0.091 | 0.577 | 0.172 | 0.034 | 0.174 | 1.44 | 0.007 | 0.16 |
| S*D*R | 1.372 | 0.917 | 1.323 | 1.712 | 1.198 | 0.562 | 1.201 | 1.35 |
| T*B*F*S | 0.150 | 0.550 | 0.374 | 0.386 | 1.023 | 1.209 | 1.221 | 0.371 |
| T*B*F*D | 1.502 | 2.903 | 2.574 | 0.067 | 3.255 | 0.001 | 2.001 | 2.721 |
| T*B*F*R | 0.486 | 1.431 | 0.759 | 0.053 | 0.511 | 1.659 | 0.195 | 0.810 |
| T*B*S*D | 0.017 | 0.103 | 0.086 | 0.418 | 0.137 | 0.155 | 0.256 | 0.092 |
| T*B*S*R | 0.313 | 0.155 | 0.178 | 0.368 | 0.066 | 0.327 | 0.24 | 0.19 |
| T*B*D*R | 0.351 | 0.201 | 0.247 | 0.395 | 0.001 | 0.095 | 0.069 | 0.33 |
| T*F*S*D | 0.418 | 0.371 | 0.392 | 0.749 | 0.800 | 1.369 | 1.905 | 0.388 |
| T*F*S*R | 0.158 | 0.269 | 0.209 | 0.255 | 0.102 | 0.439 | 0.201 | 0.278 |
| T*F*D*R | 0.318 | 0.015 | 0.006 | 0.197 | 0.101 | 0.031 | 0.102 | 0.01 |
| T*S*D*R | 2.409* | 2.891** | 2.650* | 1.626 | 3.055* | 0.677 | 1.315 | 2.654 |
| B*F*S*D | 0.171 | 0.281 | 0.198 | 0.086 | 0.322 | 0.148 | 0.173 | 0.227 |
| B*F*S*R | 0.116 | 0.241 | 0.091 | 0.079 | 0.091 | 0.54 | 0.108 | 0.102 |
| B*F*D*R | 0.057 | 0.357 | 0.468 | 0.111 | 0.807 | 0.353 | 0.639 | 0.507 |
| B*S*D*R | 0.124 | 0.237 | 0.168 | 0.961 | 0.460 | 0.718 | 0.876 | 0.150 |
| F*S*D*R | 1.575 | 1.901 | 2.645* | 0.387 | 2.868 | 0.501 | 0.750 | 2.697* |
| T*B*F*S*D | 0.036 | 0.197 | 0.240 | 0.252 | 0.828 | 0.447 | 0.640 | 0.289 |
| T*B*F*S*R | 0.214 | 0.178 | 0.250 | 0.190 | 0.110 | 0.066 | 0.097 | 0.210 |
| T*B*F*D*R | 0.015 | 0.003 | 0.002 | 0.273 | 0.163 | 0.027 | 0.248 | 0.001 |
| T*B*S*D*R | 0.461 | 0.478 | 0.583 | 0.726 | 0.158 | 0.371 | 0.277 | 0.664 |
| T*F*S*D*R | 3.650** | 4.388*** | 4.026 | 1.221 | 3.192 | 0.620 | 0.289 | 4.048*** |
| B*F*S*D*R | 0.165 | 0.223 | 0.246 | 0.563 | 0.109 | 0.238 | 0.228 | 0.264 |
| T*B*F*S*D*R | 0.140 | 0.161 | 0.185 | 0.065 | 0.426 | 0.197 | 0.239 | 0.153 |

1: *** = P < 0.005, ** = P < 0.01, * = P < 0.05
