# Supplementary material for: Changes in reflectance of rice seedlings during planthopper feeding as detected by digital camera: Potential applications for high-throughput phenotyping
Source: PLoS One. 2020 Aug 27;15(8):e0238173. doi: 10.1371/journal.pone.0238173 (PMC7451558; doi:10.1371/journal.pone.0238173)
Supplement: S8 Table — (DOCX) [file pone.0238173.s016.docx]

**Table S8: Results of univariate GLM for effects of test conditions on plant numbers and weight in Standard Seedling Seed-box Tests**

| Sources of variation | Number of plants | Average weight |
| --- | --- | --- |
| Plant age (T) | 0.955 | 3828.975*** |
| Box size (S) | 512.388*** | 16.911*** |
| Density (D) | 1647.894*** | 288.420*** |
| Distribution (R) | 0.498 | 5.183* |
| T*S | 4.605*** | 9.235*** |
| T*D | 12.577*** | 130.006*** |
| T*R | 1.568 | 2.203 |
| S*D | 189.444*** | 8.340*** |
| S*R | 0.107 | 1.489 |
| D*R | 4.187* | 0.982 |
| T*S*D | 0.487 | 4.810*** |
| T*S*R | 0.224 | 1.074 |
| T*D*R | 0.016 | 1.734 |
| S*D*R | 1.163 | 2.767* |
| T*S*D*R | 0.701 | 2.285 |

1: *** = P < 0.005, ** = P < 0.01, * = P < 0.05
