# Supplementary material for: Changes in reflectance of rice seedlings during planthopper feeding as detected by digital camera: Potential applications for high-throughput phenotyping
Source: PLoS One. 2020 Aug 27;15(8):e0238173. doi: 10.1371/journal.pone.0238173 (PMC7451558; doi:10.1371/journal.pone.0238173)
Supplement: S9 Table — (DOCX) [file pone.0238173.s017.docx]

**Table S9. Reflectance data from Standard Seedling Seed-box Tests** (SSSTs) (BPH = brown planthopper; WBPH = whitebacked planthopper; mean R = mean red reflectance, mean G = mean green reflectance, mean B = mean blue reflectance)

| Replication | Treatment | Variety | Day | Mean R | Mean G | Mean B |
| --- | --- | --- | --- | --- | --- | --- |
| 1 | BPH | ADR52 | 1 | 98.89 | 135.67 | 64.54 |
| 1 | BPH | ADR52 | 2 | 65.48 | 98.32 | 60.54 |
| 1 | BPH | ADR52 | 3 | 73.47 | 108.57 | 63.54 |
| 1 | BPH | ADR52 | 4 | 84.95 | 115.91 | 65.83 |
| 1 | BPH | ADR52 | 5 | 95.95 | 116.47 | 54.31 |
| 1 | BPH | ADR52 | 6 | 89.97 | 117.48 | 70.66 |
| 1 | BPH | ADR52 | 7 | 110.07 | 141.52 | 82.88 |
| 1 | BPH | ADR52 | 8 | 98.16 | 128.43 | 87.65 |
| 1 | BPH | ADR52 | 9 | 96.56 | 120.70 | 86.99 |
| 1 | BPH | ADR52 | 10 | 90.08 | 110.30 | 81.04 |
| 1 | CONTROL | ADR52 | 1 | 106.46 | 135.61 | 60.44 |
| 1 | CONTROL | ADR52 | 2 | 99.49 | 137.96 | 78.34 |
| 1 | CONTROL | ADR52 | 3 | 107.80 | 142.43 | 65.01 |
| 1 | CONTROL | ADR52 | 4 | 86.98 | 120.06 | 66.70 |
| 1 | CONTROL | ADR52 | 5 | 101.47 | 132.32 | 70.13 |
| 1 | CONTROL | ADR52 | 6 | 93.14 | 112.73 | 58.50 |
| 1 | CONTROL | ADR52 | 7 | 112.75 | 136.13 | 69.01 |
| 1 | CONTROL | ADR52 | 8 | 109.25 | 137.47 | 79.65 |
| 1 | CONTROL | ADR52 | 9 | 120.67 | 139.74 | 76.74 |
| 1 | CONTROL | ADR52 | 10 | 113.98 | 128.15 | 68.80 |
| 1 | WBPH | ADR52 | 1 | 100.94 | 134.85 | 60.47 |
| 1 | WBPH | ADR52 | 2 | 71.22 | 103.22 | 54.94 |
| 1 | WBPH | ADR52 | 3 | 104.59 | 132.86 | 65.18 |
| 1 | WBPH | ADR52 | 4 | 87.94 | 116.98 | 65.31 |
| 1 | WBPH | ADR52 | 5 | 87.77 | 114.57 | 60.06 |
| 1 | WBPH | ADR52 | 6 | 102.22 | 117.07 | 66.77 |
| 1 | WBPH | ADR52 | 7 | 91.56 | 109.40 | 67.59 |
| 1 | WBPH | ADR52 | 8 | 83.37 | 105.47 | 77.89 |
| 1 | WBPH | ADR52 | 9 | 95.26 | 112.44 | 76.32 |
| 1 | WBPH | ADR52 | 10 | 84.50 | 102.39 | 77.34 |
| 2 | BPH | ADR52 | 1 | 74.20 | 100.18 | 55.61 |
| 2 | BPH | ADR52 | 2 | 73.96 | 103.98 | 57.60 |
| 2 | BPH | ADR52 | 3 | 73.72 | 107.78 | 59.59 |
| 2 | BPH | ADR52 | 4 | 69.00 | 99.70 | 63.24 |
| 2 | BPH | ADR52 | 5 | 64.28 | 91.62 | 66.88 |
| 2 | BPH | ADR52 | 6 | 78.61 | 97.79 | 81.83 |
| 2 | BPH | ADR52 | 7 | 95.84 | 108.13 | 97.78 |
| 2 | BPH | ADR52 | 8 | 113.06 | 118.46 | 113.73 |
| 2 | BPH | ADR52 | 9 | 116.64 | 118.11 | 113.40 |
| 2 | BPH | ADR52 | 10 | 120.22 | 117.76 | 113.06 |
| 2 | CONTROL | ADR52 | 1 | 77.03 | 100.35 | 40.72 |
| 2 | CONTROL | ADR52 | 2 | 70.28 | 101.27 | 53.97 |
| 2 | CONTROL | ADR52 | 3 | 63.53 | 102.18 | 67.22 |
| 2 | CONTROL | ADR52 | 4 | 71.44 | 106.22 | 54.35 |
| 2 | CONTROL | ADR52 | 5 | 79.34 | 110.26 | 41.47 |
| 2 | CONTROL | ADR52 | 6 | 97.76 | 121.39 | 38.20 |
| 2 | CONTROL | ADR52 | 7 | 91.68 | 120.05 | 44.05 |
| 2 | CONTROL | ADR52 | 8 | 85.60 | 118.71 | 49.90 |
| 2 | CONTROL | ADR52 | 9 | 100.08 | 128.93 | 48.89 |
| 2 | CONTROL | ADR52 | 10 | 114.55 | 139.15 | 47.88 |
| 2 | WBPH | ADR52 | 1 | 81.50 | 115.66 | 58.61 |
| 2 | WBPH | ADR52 | 2 | 80.97 | 113.70 | 55.37 |
| 2 | WBPH | ADR52 | 3 | 80.43 | 111.73 | 52.12 |
| 2 | WBPH | ADR52 | 4 | 79.88 | 111.83 | 52.90 |
| 2 | WBPH | ADR52 | 5 | 79.32 | 111.93 | 53.68 |
| 2 | WBPH | ADR52 | 6 | 79.71 | 108.35 | 46.25 |
| 2 | WBPH | ADR52 | 7 | 89.97 | 118.56 | 53.54 |
| 2 | WBPH | ADR52 | 8 | 100.23 | 128.76 | 60.82 |
| 2 | WBPH | ADR52 | 9 | 97.85 | 124.89 | 60.01 |
| 2 | WBPH | ADR52 | 10 | 95.46 | 121.02 | 59.19 |
| 3 | BPH | ADR52 | 1 | 96.70 | 126.85 | 55.21 |
| 3 | BPH | ADR52 | 2 | 95.95 | 128.86 | 57.26 |
| 3 | BPH | ADR52 | 3 | 95.19 | 130.87 | 59.31 |
| 3 | BPH | ADR52 | 4 | 92.91 | 127.26 | 56.82 |
| 3 | BPH | ADR52 | 5 | 90.62 | 123.65 | 54.33 |
| 3 | BPH | ADR52 | 6 | 84.32 | 114.63 | 47.15 |
| 3 | BPH | ADR52 | 7 | 88.21 | 118.96 | 53.29 |
| 3 | BPH | ADR52 | 8 | 92.10 | 123.28 | 59.42 |
| 3 | BPH | ADR52 | 9 | 107.91 | 138.41 | 60.93 |
| 3 | BPH | ADR52 | 10 | 109.49 | 139.17 | 67.78 |
| 3 | CONTROL | ADR52 | 1 | 92.26 | 126.78 | 58.39 |
| 3 | CONTROL | ADR52 | 2 | 92.56 | 127.29 | 58.22 |
| 3 | CONTROL | ADR52 | 3 | 92.85 | 127.80 | 58.05 |
| 3 | CONTROL | ADR52 | 4 | 80.40 | 112.66 | 48.27 |
| 3 | CONTROL | ADR52 | 5 | 67.95 | 97.52 | 38.48 |
| 3 | CONTROL | ADR52 | 6 | 83.82 | 115.68 | 48.22 |
| 3 | CONTROL | ADR52 | 7 | 87.93 | 121.36 | 48.41 |
| 3 | CONTROL | ADR52 | 8 | 92.03 | 127.04 | 48.59 |
| 3 | CONTROL | ADR52 | 9 | 88.99 | 118.96 | 38.63 |
| 3 | CONTROL | ADR52 | 10 | 99.60 | 133.61 | 55.76 |
| 3 | WBPH | ADR52 | 1 | 86.38 | 122.03 | 65.80 |
| 3 | WBPH | ADR52 | 2 | 84.59 | 114.64 | 58.92 |
| 3 | WBPH | ADR52 | 3 | 82.80 | 107.25 | 52.04 |
| 3 | WBPH | ADR52 | 4 | 81.25 | 104.98 | 49.40 |
| 3 | WBPH | ADR52 | 5 | 79.69 | 102.71 | 46.75 |
| 3 | WBPH | ADR52 | 6 | 100.36 | 118.64 | 54.69 |
| 3 | WBPH | ADR52 | 7 | 109.98 | 125.26 | 62.86 |
| 3 | WBPH | ADR52 | 8 | 119.60 | 131.88 | 71.03 |
| 3 | WBPH | ADR52 | 9 | 95.29 | 103.11 | 66.35 |
| 3 | WBPH | ADR52 | 10 | 126.20 | 133.10 | 85.59 |
| 1 | BPH | ARC10239 | 1 | 114.48 | 146.25 | 66.28 |
| 1 | BPH | ARC10239 | 2 | 99.28 | 128.00 | 69.14 |
| 1 | BPH | ARC10239 | 3 | 95.37 | 118.58 | 58.98 |
| 1 | BPH | ARC10239 | 4 | 94.91 | 124.65 | 75.91 |
| 1 | BPH | ARC10239 | 5 | 97.94 | 118.79 | 57.83 |
| 1 | BPH | ARC10239 | 6 | 104.48 | 131.80 | 83.49 |
| 1 | BPH | ARC10239 | 7 | 100.13 | 123.83 | 112.25 |
| 1 | BPH | ARC10239 | 8 | 106.43 | 111.08 | 109.63 |
| 1 | BPH | ARC10239 | 9 | 101.62 | 102.27 | 102.63 |
| 1 | BPH | ARC10239 | 10 | 103.02 | 106.77 | 107.75 |
| 1 | CONTROL | ARC10239 | 1 | 104.47 | 140.53 | 66.34 |
| 1 | CONTROL | ARC10239 | 2 | 102.95 | 136.11 | 70.38 |
| 1 | CONTROL | ARC10239 | 3 | 100.84 | 134.35 | 68.00 |
| 1 | CONTROL | ARC10239 | 4 | 98.63 | 132.49 | 73.79 |
| 1 | CONTROL | ARC10239 | 5 | 104.20 | 133.50 | 79.93 |
| 1 | CONTROL | ARC10239 | 6 | 98.43 | 118.29 | 64.75 |
| 1 | CONTROL | ARC10239 | 7 | 96.76 | 118.40 | 56.79 |
| 1 | CONTROL | ARC10239 | 8 | 132.33 | 153.47 | 75.56 |
| 1 | CONTROL | ARC10239 | 9 | 131.43 | 155.75 | 82.22 |
| 1 | CONTROL | ARC10239 | 10 | 114.63 | 134.58 | 67.37 |
| 1 | WBPH | ARC10239 | 1 | 104.93 | 137.11 | 65.79 |
| 1 | WBPH | ARC10239 | 2 | 87.41 | 118.15 | 58.45 |
| 1 | WBPH | ARC10239 | 3 | 117.62 | 138.88 | 67.62 |
| 1 | WBPH | ARC10239 | 4 | 129.23 | 143.66 | 83.18 |
| 1 | WBPH | ARC10239 | 5 | 126.07 | 136.51 | 87.21 |
| 1 | WBPH | ARC10239 | 6 | 118.44 | 131.82 | 94.54 |
| 1 | WBPH | ARC10239 | 7 | 112.83 | 122.14 | 90.80 |
| 1 | WBPH | ARC10239 | 8 | 123.91 | 133.43 | 109.63 |
| 1 | WBPH | ARC10239 | 9 | 110.04 | 118.41 | 97.20 |
| 1 | WBPH | ARC10239 | 10 | 111.76 | 120.01 | 99.12 |
| 2 | BPH | ARC10239 | 1 | 85.34 | 99.48 | 80.58 |
| 2 | BPH | ARC10239 | 2 | 90.50 | 97.64 | 84.12 |
| 2 | BPH | ARC10239 | 3 | 95.65 | 95.80 | 87.66 |
| 2 | BPH | ARC10239 | 4 | 96.68 | 95.45 | 89.40 |
| 2 | BPH | ARC10239 | 5 | 97.71 | 95.10 | 91.14 |
| 2 | BPH | ARC10239 | 6 | 109.64 | 103.37 | 98.08 |
| 2 | BPH | ARC10239 | 7 | 123.49 | 116.21 | 110.16 |
| 2 | BPH | ARC10239 | 8 | 137.33 | 129.05 | 122.23 |
| 2 | BPH | ARC10239 | 9 | 127.68 | 121.00 | 115.39 |
| 2 | BPH | ARC10239 | 10 | 118.03 | 112.95 | 108.54 |
| 2 | CONTROL | ARC10239 | 1 | 101.22 | 134.42 | 57.29 |
| 2 | CONTROL | ARC10239 | 2 | 90.37 | 127.28 | 67.23 |
| 2 | CONTROL | ARC10239 | 3 | 79.52 | 120.14 | 77.16 |
| 2 | CONTROL | ARC10239 | 4 | 83.99 | 120.80 | 64.12 |
| 2 | CONTROL | ARC10239 | 5 | 88.45 | 121.46 | 51.07 |
| 2 | CONTROL | ARC10239 | 6 | 79.78 | 111.56 | 44.63 |
| 2 | CONTROL | ARC10239 | 7 | 87.87 | 120.22 | 46.78 |
| 2 | CONTROL | ARC10239 | 8 | 95.95 | 128.87 | 48.92 |
| 2 | CONTROL | ARC10239 | 9 | 98.17 | 128.39 | 52.64 |
| 2 | CONTROL | ARC10239 | 10 | 100.38 | 127.91 | 56.36 |
| 2 | WBPH | ARC10239 | 1 | 91.60 | 125.17 | 65.61 |
| 2 | WBPH | ARC10239 | 2 | 90.34 | 118.12 | 59.97 |
| 2 | WBPH | ARC10239 | 3 | 89.08 | 111.06 | 54.32 |
| 2 | WBPH | ARC10239 | 4 | 88.07 | 108.06 | 61.89 |
| 2 | WBPH | ARC10239 | 5 | 87.05 | 105.05 | 69.45 |
| 2 | WBPH | ARC10239 | 6 | 94.99 | 101.21 | 80.29 |
| 2 | WBPH | ARC10239 | 7 | 109.75 | 114.33 | 97.76 |
| 2 | WBPH | ARC10239 | 8 | 124.50 | 127.45 | 115.23 |
| 2 | WBPH | ARC10239 | 9 | 111.94 | 112.47 | 101.49 |
| 2 | WBPH | ARC10239 | 10 | 99.38 | 97.48 | 87.75 |
| 3 | BPH | ARC10239 | 1 | 91.84 | 127.25 | 75.81 |
| 3 | BPH | ARC10239 | 2 | 93.42 | 123.20 | 71.95 |
| 3 | BPH | ARC10239 | 3 | 95.00 | 119.14 | 68.08 |
| 3 | BPH | ARC10239 | 4 | 95.52 | 115.63 | 77.92 |
| 3 | BPH | ARC10239 | 5 | 96.04 | 112.12 | 87.75 |
| 3 | BPH | ARC10239 | 6 | 98.96 | 108.13 | 97.85 |
| 3 | BPH | ARC10239 | 7 | 113.81 | 116.84 | 108.52 |
| 3 | BPH | ARC10239 | 8 | 128.65 | 125.54 | 119.19 |
| 3 | BPH | ARC10239 | 9 | 115.29 | 112.11 | 107.12 |
| 3 | BPH | ARC10239 | 10 | 115.64 | 115.22 | 113.09 |
| 3 | CONTROL | ARC10239 | 1 | 106.59 | 140.22 | 57.45 |
| 3 | CONTROL | ARC10239 | 2 | 103.98 | 138.80 | 57.66 |
| 3 | CONTROL | ARC10239 | 3 | 101.37 | 137.38 | 57.87 |
| 3 | CONTROL | ARC10239 | 4 | 110.75 | 141.87 | 55.75 |
| 3 | CONTROL | ARC10239 | 5 | 120.13 | 146.35 | 53.63 |
| 3 | CONTROL | ARC10239 | 6 | 97.08 | 129.33 | 54.46 |
| 3 | CONTROL | ARC10239 | 7 | 108.92 | 141.36 | 61.44 |
| 3 | CONTROL | ARC10239 | 8 | 120.75 | 153.39 | 68.42 |
| 3 | CONTROL | ARC10239 | 9 | 123.20 | 155.36 | 62.88 |
| 3 | CONTROL | ARC10239 | 10 | 139.70 | 166.68 | 70.74 |
| 3 | WBPH | ARC10239 | 1 | 107.73 | 136.16 | 58.70 |
| 3 | WBPH | ARC10239 | 2 | 99.29 | 129.87 | 58.07 |
| 3 | WBPH | ARC10239 | 3 | 90.84 | 123.58 | 57.44 |
| 3 | WBPH | ARC10239 | 4 | 96.00 | 124.01 | 54.40 |
| 3 | WBPH | ARC10239 | 5 | 101.16 | 124.44 | 51.35 |
| 3 | WBPH | ARC10239 | 6 | 95.69 | 112.59 | 50.38 |
| 3 | WBPH | ARC10239 | 7 | 111.49 | 124.91 | 63.41 |
| 3 | WBPH | ARC10239 | 8 | 127.28 | 137.22 | 76.43 |
| 3 | WBPH | ARC10239 | 9 | 105.85 | 118.69 | 76.58 |
| 3 | WBPH | ARC10239 | 10 | 128.53 | 140.66 | 92.27 |
| 1 | BPH | ARC10550 | 1 | 111.56 | 141.38 | 66.97 |
| 1 | BPH | ARC10550 | 2 | 84.81 | 118.16 | 54.82 |
| 1 | BPH | ARC10550 | 3 | 96.29 | 118.85 | 51.86 |
| 1 | BPH | ARC10550 | 4 | 105.83 | 134.99 | 51.15 |
| 1 | BPH | ARC10550 | 5 | 91.07 | 121.37 | 53.75 |
| 1 | BPH | ARC10550 | 6 | 110.00 | 136.86 | 73.84 |
| 1 | BPH | ARC10550 | 7 | 106.25 | 131.99 | 89.26 |
| 1 | BPH | ARC10550 | 8 | 121.35 | 136.21 | 120.40 |
| 1 | BPH | ARC10550 | 9 | 129.78 | 129.96 | 112.75 |
| 1 | BPH | ARC10550 | 10 | 118.93 | 118.73 | 113.34 |
| 1 | CONTROL | ARC10550 | 1 | 92.17 | 130.33 | 61.92 |
| 1 | CONTROL | ARC10550 | 2 | 100.53 | 128.72 | 59.54 |
| 1 | CONTROL | ARC10550 | 3 | 113.99 | 148.85 | 60.20 |
| 1 | CONTROL | ARC10550 | 4 | 85.42 | 106.02 | 43.84 |
| 1 | CONTROL | ARC10550 | 5 | 117.69 | 150.49 | 62.94 |
| 1 | CONTROL | ARC10550 | 6 | 85.81 | 113.35 | 48.57 |
| 1 | CONTROL | ARC10550 | 7 | 89.55 | 109.35 | 39.41 |
| 1 | CONTROL | ARC10550 | 8 | 106.96 | 135.61 | 62.98 |
| 1 | CONTROL | ARC10550 | 9 | 119.78 | 148.58 | 65.33 |
| 1 | CONTROL | ARC10550 | 10 | 98.91 | 123.42 | 52.91 |
| 1 | WBPH | ARC10550 | 1 | 100.45 | 135.05 | 65.96 |
| 1 | WBPH | ARC10550 | 2 | 75.35 | 107.16 | 56.93 |
| 1 | WBPH | ARC10550 | 3 | 110.15 | 140.02 | 62.03 |
| 1 | WBPH | ARC10550 | 4 | 80.33 | 111.22 | 61.33 |
| 1 | WBPH | ARC10550 | 5 | 88.91 | 118.37 | 66.74 |
| 1 | WBPH | ARC10550 | 6 | 94.02 | 118.63 | 74.94 |
| 1 | WBPH | ARC10550 | 7 | 97.96 | 119.87 | 77.27 |
| 1 | WBPH | ARC10550 | 8 | 105.29 | 122.97 | 95.68 |
| 1 | WBPH | ARC10550 | 9 | 101.82 | 107.03 | 82.50 |
| 1 | WBPH | ARC10550 | 10 | 99.77 | 106.83 | 97.83 |
| 2 | BPH | ARC10550 | 1 | 81.53 | 107.24 | 52.39 |
| 2 | BPH | ARC10550 | 2 | 77.31 | 102.22 | 55.13 |
| 2 | BPH | ARC10550 | 3 | 73.09 | 97.19 | 57.86 |
| 2 | BPH | ARC10550 | 4 | 78.45 | 101.75 | 66.54 |
| 2 | BPH | ARC10550 | 5 | 83.80 | 106.31 | 75.21 |
| 2 | BPH | ARC10550 | 6 | 91.49 | 100.15 | 81.76 |
| 2 | BPH | ARC10550 | 7 | 107.36 | 112.25 | 99.11 |
| 2 | BPH | ARC10550 | 8 | 123.22 | 124.35 | 116.46 |
| 2 | BPH | ARC10550 | 9 | 120.91 | 119.92 | 112.75 |
| 2 | BPH | ARC10550 | 10 | 118.59 | 115.49 | 109.04 |
| 2 | CONTROL | ARC10550 | 1 | 85.77 | 111.22 | 43.46 |
| 2 | CONTROL | ARC10550 | 2 | 81.03 | 112.87 | 53.09 |
| 2 | CONTROL | ARC10550 | 3 | 76.29 | 114.51 | 62.71 |
| 2 | CONTROL | ARC10550 | 4 | 79.44 | 115.16 | 52.97 |
| 2 | CONTROL | ARC10550 | 5 | 82.58 | 115.81 | 43.22 |
| 2 | CONTROL | ARC10550 | 6 | 102.53 | 136.95 | 53.77 |
| 2 | CONTROL | ARC10550 | 7 | 115.41 | 149.69 | 62.81 |
| 2 | CONTROL | ARC10550 | 8 | 128.29 | 162.42 | 71.85 |
| 2 | CONTROL | ARC10550 | 9 | 124.61 | 152.09 | 65.15 |
| 2 | CONTROL | ARC10550 | 10 | 120.93 | 141.76 | 58.44 |
| 2 | WBPH | ARC10550 | 1 | 86.73 | 114.77 | 49.28 |
| 2 | WBPH | ARC10550 | 2 | 75.28 | 105.35 | 48.87 |
| 2 | WBPH | ARC10550 | 3 | 63.83 | 95.92 | 48.45 |
| 2 | WBPH | ARC10550 | 4 | 73.66 | 102.99 | 46.68 |
| 2 | WBPH | ARC10550 | 5 | 83.49 | 110.06 | 44.90 |
| 2 | WBPH | ARC10550 | 6 | 82.39 | 109.60 | 64.19 |
| 2 | WBPH | ARC10550 | 7 | 94.15 | 116.02 | 78.57 |
| 2 | WBPH | ARC10550 | 8 | 105.91 | 122.43 | 92.94 |
| 2 | WBPH | ARC10550 | 9 | 105.36 | 117.96 | 93.67 |
| 2 | WBPH | ARC10550 | 10 | 104.80 | 113.48 | 94.39 |
| 3 | BPH | ARC10550 | 1 | 101.21 | 136.18 | 64.13 |
| 3 | BPH | ARC10550 | 2 | 100.88 | 136.05 | 63.52 |
| 3 | BPH | ARC10550 | 3 | 100.54 | 135.92 | 62.91 |
| 3 | BPH | ARC10550 | 4 | 94.54 | 126.96 | 58.18 |
| 3 | BPH | ARC10550 | 5 | 88.54 | 117.99 | 53.44 |
| 3 | BPH | ARC10550 | 6 | 85.04 | 112.98 | 51.19 |
| 3 | BPH | ARC10550 | 7 | 92.28 | 119.99 | 63.95 |
| 3 | BPH | ARC10550 | 8 | 99.51 | 126.99 | 76.71 |
| 3 | BPH | ARC10550 | 9 | 118.31 | 121.81 | 102.46 |
| 3 | BPH | ARC10550 | 10 | 120.26 | 124.22 | 112.61 |
| 3 | CONTROL | ARC10550 | 1 | 89.84 | 124.14 | 56.29 |
| 3 | CONTROL | ARC10550 | 2 | 95.45 | 129.98 | 55.66 |
| 3 | CONTROL | ARC10550 | 3 | 101.05 | 135.82 | 55.02 |
| 3 | CONTROL | ARC10550 | 4 | 97.93 | 131.51 | 53.77 |
| 3 | CONTROL | ARC10550 | 5 | 94.81 | 127.19 | 52.51 |
| 3 | CONTROL | ARC10550 | 6 | 83.13 | 113.04 | 44.71 |
| 3 | CONTROL | ARC10550 | 7 | 98.41 | 129.62 | 51.36 |
| 3 | CONTROL | ARC10550 | 8 | 113.69 | 146.20 | 58.00 |
| 3 | CONTROL | ARC10550 | 9 | 100.72 | 129.96 | 53.25 |
| 3 | CONTROL | ARC10550 | 10 | 128.77 | 159.91 | 62.46 |
| 3 | WBPH | ARC10550 | 1 | 91.94 | 128.06 | 67.55 |
| 3 | WBPH | ARC10550 | 2 | 83.86 | 115.59 | 61.01 |
| 3 | WBPH | ARC10550 | 3 | 75.78 | 103.12 | 54.47 |
| 3 | WBPH | ARC10550 | 4 | 79.38 | 105.50 | 61.68 |
| 3 | WBPH | ARC10550 | 5 | 82.97 | 107.87 | 68.88 |
| 3 | WBPH | ARC10550 | 6 | 101.12 | 118.25 | 77.76 |
| 3 | WBPH | ARC10550 | 7 | 102.37 | 116.19 | 83.00 |
| 3 | WBPH | ARC10550 | 8 | 103.62 | 114.13 | 88.23 |
| 3 | WBPH | ARC10550 | 9 | 105.41 | 114.28 | 92.07 |
| 3 | WBPH | ARC10550 | 10 | 118.66 | 126.26 | 107.28 |
| 1 | BPH | ARC11367 | 1 | 106.59 | 143.32 | 84.35 |
| 1 | BPH | ARC11367 | 2 | 75.07 | 108.44 | 67.29 |
| 1 | BPH | ARC11367 | 3 | 85.29 | 113.06 | 63.77 |
| 1 | BPH | ARC11367 | 4 | 65.15 | 102.12 | 75.87 |
| 1 | BPH | ARC11367 | 5 | 85.52 | 103.69 | 68.07 |
| 1 | BPH | ARC11367 | 6 | 94.47 | 106.43 | 74.18 |
| 1 | BPH | ARC11367 | 7 | 111.64 | 118.17 | 92.08 |
| 1 | BPH | ARC11367 | 8 | 94.81 | 98.95 | 86.55 |
| 1 | BPH | ARC11367 | 9 | 107.26 | 102.88 | 97.17 |
| 1 | BPH | ARC11367 | 10 | 100.00 | 98.45 | 97.99 |
| 1 | CONTROL | ARC11367 | 1 | 102.12 | 138.42 | 69.07 |
| 1 | CONTROL | ARC11367 | 2 | 100.24 | 129.94 | 77.91 |
| 1 | CONTROL | ARC11367 | 3 | 106.82 | 143.19 | 76.16 |
| 1 | CONTROL | ARC11367 | 4 | 79.00 | 114.67 | 70.56 |
| 1 | CONTROL | ARC11367 | 5 | 108.23 | 141.59 | 77.61 |
| 1 | CONTROL | ARC11367 | 6 | 86.50 | 121.01 | 74.86 |
| 1 | CONTROL | ARC11367 | 7 | 101.26 | 127.89 | 78.74 |
| 1 | CONTROL | ARC11367 | 8 | 110.82 | 148.01 | 88.80 |
| 1 | CONTROL | ARC11367 | 9 | 126.03 | 150.71 | 85.70 |
| 1 | CONTROL | ARC11367 | 10 | 103.59 | 137.50 | 89.58 |
| 1 | WBPH | ARC11367 | 1 | 106.39 | 139.00 | 85.20 |
| 1 | WBPH | ARC11367 | 2 | 85.07 | 115.84 | 73.78 |
| 1 | WBPH | ARC11367 | 3 | 103.39 | 134.51 | 82.10 |
| 1 | WBPH | ARC11367 | 4 | 112.04 | 142.19 | 90.33 |
| 1 | WBPH | ARC11367 | 5 | 97.83 | 111.60 | 63.18 |
| 1 | WBPH | ARC11367 | 6 | 106.65 | 129.25 | 95.22 |
| 1 | WBPH | ARC11367 | 7 | 104.28 | 117.18 | 71.61 |
| 1 | WBPH | ARC11367 | 8 | 111.02 | 125.61 | 84.03 |
| 1 | WBPH | ARC11367 | 9 | 95.75 | 104.66 | 68.83 |
| 1 | WBPH | ARC11367 | 10 | 83.35 | 99.23 | 80.21 |
| 1 | BPH | ARC6650 | 1 | 110.37 | 149.45 | 72.73 |
| 1 | BPH | ARC6650 | 2 | 95.10 | 119.32 | 57.65 |
| 1 | BPH | ARC6650 | 3 | 127.92 | 150.22 | 61.36 |
| 1 | BPH | ARC6650 | 4 | 105.92 | 138.88 | 68.56 |
| 1 | BPH | ARC6650 | 5 | 116.44 | 137.40 | 55.05 |
| 1 | BPH | ARC6650 | 6 | 112.47 | 136.68 | 74.38 |
| 1 | BPH | ARC6650 | 7 | 108.77 | 130.49 | 67.14 |
| 1 | BPH | ARC6650 | 8 | 110.63 | 132.89 | 91.84 |
| 1 | BPH | ARC6650 | 9 | 91.24 | 106.95 | 70.22 |
| 1 | BPH | ARC6650 | 10 | 118.21 | 130.28 | 92.40 |
| 1 | CONTROL | ARC6650 | 1 | 114.95 | 148.36 | 64.38 |
| 1 | CONTROL | ARC6650 | 2 | 112.57 | 145.58 | 67.47 |
| 1 | CONTROL | ARC6650 | 3 | 118.09 | 151.79 | 72.60 |
| 1 | CONTROL | ARC6650 | 4 | 101.07 | 130.62 | 66.03 |
| 1 | CONTROL | ARC6650 | 5 | 126.68 | 150.24 | 59.93 |
| 1 | CONTROL | ARC6650 | 6 | 115.87 | 137.56 | 68.39 |
| 1 | CONTROL | ARC6650 | 7 | 119.30 | 139.61 | 55.83 |
| 1 | CONTROL | ARC6650 | 8 | 130.17 | 148.17 | 66.54 |
| 1 | CONTROL | ARC6650 | 9 | 137.75 | 154.06 | 67.78 |
| 1 | CONTROL | ARC6650 | 10 | 122.95 | 146.42 | 79.38 |
| 1 | WBPH | ARC6650 | 1 | 113.48 | 151.37 | 77.54 |
| 1 | WBPH | ARC6650 | 2 | 92.92 | 121.82 | 66.90 |
| 1 | WBPH | ARC6650 | 3 | 119.20 | 150.97 | 81.62 |
| 1 | WBPH | ARC6650 | 4 | 110.27 | 128.16 | 71.71 |
| 1 | WBPH | ARC6650 | 5 | 112.00 | 121.29 | 67.42 |
| 1 | WBPH | ARC6650 | 6 | 106.60 | 118.65 | 77.24 |
| 1 | WBPH | ARC6650 | 7 | 127.08 | 136.21 | 97.23 |
| 1 | WBPH | ARC6650 | 8 | 125.49 | 125.29 | 92.94 |
| 1 | WBPH | ARC6650 | 9 | 114.69 | 119.12 | 103.78 |
| 1 | WBPH | ARC6650 | 10 | 110.38 | 117.72 | 100.02 |
| 2 | BPH | ARC6650 | 1 | 83.85 | 117.73 | 67.37 |
| 2 | BPH | ARC6650 | 2 | 82.16 | 111.61 | 56.79 |
| 2 | BPH | ARC6650 | 3 | 80.47 | 105.48 | 46.20 |
| 2 | BPH | ARC6650 | 4 | 73.86 | 97.45 | 53.34 |
| 2 | BPH | ARC6650 | 5 | 67.24 | 89.41 | 60.47 |
| 2 | BPH | ARC6650 | 6 | 80.13 | 85.55 | 77.83 |
| 2 | BPH | ARC6650 | 7 | 108.96 | 110.08 | 102.78 |
| 2 | BPH | ARC6650 | 8 | 137.79 | 134.61 | 127.73 |
| 2 | BPH | ARC6650 | 9 | 123.32 | 121.48 | 117.05 |
| 2 | BPH | ARC6650 | 10 | 108.85 | 108.35 | 106.36 |
| 2 | CONTROL | ARC6650 | 1 | 92.18 | 117.71 | 45.03 |
| 2 | CONTROL | ARC6650 | 2 | 79.31 | 112.72 | 61.05 |
| 2 | CONTROL | ARC6650 | 3 | 66.43 | 107.72 | 77.06 |
| 2 | CONTROL | ARC6650 | 4 | 77.86 | 110.68 | 57.56 |
| 2 | CONTROL | ARC6650 | 5 | 89.29 | 113.63 | 38.05 |
| 2 | CONTROL | ARC6650 | 6 | 91.25 | 123.21 | 49.22 |
| 2 | CONTROL | ARC6650 | 7 | 96.51 | 129.18 | 54.21 |
| 2 | CONTROL | ARC6650 | 8 | 101.76 | 135.15 | 59.20 |
| 2 | CONTROL | ARC6650 | 9 | 100.07 | 134.10 | 61.42 |
| 2 | CONTROL | ARC6650 | 10 | 98.38 | 133.04 | 63.63 |
| 2 | WBPH | ARC6650 | 1 | 91.69 | 120.83 | 54.83 |
| 2 | WBPH | ARC6650 | 2 | 89.03 | 120.29 | 58.77 |
| 2 | WBPH | ARC6650 | 3 | 86.37 | 119.74 | 62.71 |
| 2 | WBPH | ARC6650 | 4 | 89.75 | 119.77 | 59.00 |
| 2 | WBPH | ARC6650 | 5 | 93.13 | 119.79 | 55.29 |
| 2 | WBPH | ARC6650 | 6 | 91.21 | 115.51 | 55.96 |
| 2 | WBPH | ARC6650 | 7 | 101.67 | 124.79 | 67.84 |
| 2 | WBPH | ARC6650 | 8 | 112.13 | 134.07 | 79.72 |
| 2 | WBPH | ARC6650 | 9 | 106.80 | 125.00 | 81.45 |
| 2 | WBPH | ARC6650 | 10 | 101.46 | 115.92 | 83.17 |
| 3 | BPH | ARC6650 | 1 | 114.90 | 143.36 | 64.76 |
| 3 | BPH | ARC6650 | 2 | 114.20 | 143.58 | 68.19 |
| 3 | BPH | ARC6650 | 3 | 113.50 | 143.80 | 71.61 |
| 3 | BPH | ARC6650 | 4 | 112.31 | 142.95 | 68.38 |
| 3 | BPH | ARC6650 | 5 | 111.12 | 142.10 | 65.14 |
| 3 | BPH | ARC6650 | 6 | 99.76 | 129.46 | 56.62 |
| 3 | BPH | ARC6650 | 7 | 104.65 | 135.15 | 59.08 |
| 3 | BPH | ARC6650 | 8 | 109.53 | 140.84 | 61.54 |
| 3 | BPH | ARC6650 | 9 | 116.91 | 137.98 | 73.78 |
| 3 | BPH | ARC6650 | 10 | 129.29 | 153.49 | 87.43 |
| 3 | CONTROL | ARC6650 | 1 | 80.99 | 114.71 | 58.72 |
| 3 | CONTROL | ARC6650 | 2 | 89.79 | 125.79 | 63.84 |
| 3 | CONTROL | ARC6650 | 3 | 98.58 | 136.87 | 68.95 |
| 3 | CONTROL | ARC6650 | 4 | 92.74 | 127.96 | 60.95 |
| 3 | CONTROL | ARC6650 | 5 | 86.89 | 119.04 | 52.94 |
| 3 | CONTROL | ARC6650 | 6 | 79.16 | 111.48 | 53.15 |
| 3 | CONTROL | ARC6650 | 7 | 92.51 | 124.29 | 58.16 |
| 3 | CONTROL | ARC6650 | 8 | 105.86 | 137.10 | 63.17 |
| 3 | CONTROL | ARC6650 | 9 | 102.48 | 130.86 | 64.05 |
| 3 | CONTROL | ARC6650 | 10 | 113.32 | 146.64 | 73.03 |
| 3 | WBPH | ARC6650 | 1 | 95.18 | 129.06 | 59.69 |
| 3 | WBPH | ARC6650 | 2 | 93.66 | 127.50 | 57.96 |
| 3 | WBPH | ARC6650 | 3 | 92.14 | 125.93 | 56.23 |
| 3 | WBPH | ARC6650 | 4 | 90.42 | 121.32 | 57.99 |
| 3 | WBPH | ARC6650 | 5 | 88.70 | 116.70 | 59.75 |
| 3 | WBPH | ARC6650 | 6 | 94.61 | 118.31 | 58.06 |
| 3 | WBPH | ARC6650 | 7 | 101.73 | 122.45 | 64.11 |
| 3 | WBPH | ARC6650 | 8 | 108.85 | 126.59 | 70.16 |
| 3 | WBPH | ARC6650 | 9 | 121.03 | 137.32 | 79.92 |
| 3 | WBPH | ARC6650 | 10 | 136.69 | 146.80 | 91.71 |
| 1 | BPH | ASD7 | 1 | 108.57 | 146.41 | 74.19 |
| 1 | BPH | ASD7 | 2 | 77.96 | 111.53 | 64.75 |
| 1 | BPH | ASD7 | 3 | 107.78 | 134.79 | 62.74 |
| 1 | BPH | ASD7 | 4 | 94.36 | 117.61 | 47.11 |
| 1 | BPH | ASD7 | 5 | 101.27 | 133.16 | 70.48 |
| 1 | BPH | ASD7 | 6 | 101.42 | 133.81 | 83.78 |
| 1 | BPH | ASD7 | 7 | 97.31 | 123.04 | 74.60 |
| 1 | BPH | ASD7 | 8 | 89.62 | 114.85 | 85.67 |
| 1 | BPH | ASD7 | 9 | 94.67 | 110.12 | 77.02 |
| 1 | BPH | ASD7 | 10 | 110.30 | 113.40 | 99.00 |
| 1 | CONTROL | ASD7 | 1 | 106.29 | 130.92 | 53.58 |
| 1 | CONTROL | ASD7 | 2 | 106.57 | 132.44 | 65.89 |
| 1 | CONTROL | ASD7 | 3 | 106.48 | 141.84 | 61.91 |
| 1 | CONTROL | ASD7 | 4 | 91.96 | 115.93 | 44.55 |
| 1 | CONTROL | ASD7 | 5 | 92.24 | 123.77 | 51.19 |
| 1 | CONTROL | ASD7 | 6 | 87.93 | 112.20 | 51.63 |
| 1 | CONTROL | ASD7 | 7 | 110.10 | 132.25 | 48.98 |
| 1 | CONTROL | ASD7 | 8 | 117.66 | 141.47 | 51.82 |
| 1 | CONTROL | ASD7 | 9 | 117.33 | 140.73 | 60.37 |
| 1 | CONTROL | ASD7 | 10 | 92.92 | 117.44 | 53.11 |
| 1 | WBPH | ASD7 | 1 | 115.82 | 143.19 | 75.45 |
| 1 | WBPH | ASD7 | 2 | 82.96 | 105.49 | 54.98 |
| 1 | WBPH | ASD7 | 3 | 104.71 | 127.24 | 73.01 |
| 1 | WBPH | ASD7 | 4 | 103.87 | 124.22 | 78.48 |
| 1 | WBPH | ASD7 | 5 | 112.62 | 120.64 | 79.54 |
| 1 | WBPH | ASD7 | 6 | 117.00 | 122.63 | 94.47 |
| 1 | WBPH | ASD7 | 7 | 97.54 | 107.00 | 88.15 |
| 1 | WBPH | ASD7 | 8 | 111.36 | 112.93 | 84.80 |
| 1 | WBPH | ASD7 | 9 | 86.84 | 94.30 | 78.75 |
| 1 | WBPH | ASD7 | 10 | 100.06 | 110.46 | 98.37 |
| 2 | BPH | ASD7 | 1 | 59.08 | 77.14 | 53.59 |
| 2 | BPH | ASD7 | 2 | 61.79 | 75.51 | 54.63 |
| 2 | BPH | ASD7 | 3 | 64.50 | 73.87 | 55.67 |
| 2 | BPH | ASD7 | 4 | 68.55 | 74.14 | 58.78 |
| 2 | BPH | ASD7 | 5 | 72.60 | 74.41 | 61.88 |
| 2 | BPH | ASD7 | 6 | 72.89 | 70.69 | 57.42 |
| 2 | BPH | ASD7 | 7 | 86.79 | 83.92 | 74.31 |
| 2 | BPH | ASD7 | 8 | 100.69 | 97.15 | 91.20 |
| 2 | BPH | ASD7 | 9 | 99.19 | 95.08 | 89.92 |
| 2 | BPH | ASD7 | 10 | 97.68 | 93.00 | 88.63 |
| 2 | CONTROL | ASD7 | 1 | 81.24 | 108.73 | 42.14 |
| 2 | CONTROL | ASD7 | 2 | 81.05 | 109.80 | 47.45 |
| 2 | CONTROL | ASD7 | 3 | 80.85 | 110.86 | 52.76 |
| 2 | CONTROL | ASD7 | 4 | 81.87 | 113.18 | 51.23 |
| 2 | CONTROL | ASD7 | 5 | 82.89 | 115.50 | 49.69 |
| 2 | CONTROL | ASD7 | 6 | 92.43 | 117.20 | 48.54 |
| 2 | CONTROL | ASD7 | 7 | 103.90 | 132.06 | 54.98 |
| 2 | CONTROL | ASD7 | 8 | 115.36 | 146.92 | 61.42 |
| 2 | CONTROL | ASD7 | 9 | 107.51 | 133.73 | 58.49 |
| 2 | CONTROL | ASD7 | 10 | 99.65 | 120.54 | 55.55 |
| 2 | WBPH | ASD7 | 1 | 81.76 | 108.19 | 53.10 |
| 2 | WBPH | ASD7 | 2 | 75.66 | 103.33 | 54.68 |
| 2 | WBPH | ASD7 | 3 | 69.55 | 98.46 | 56.26 |
| 2 | WBPH | ASD7 | 4 | 69.35 | 95.68 | 56.63 |
| 2 | WBPH | ASD7 | 5 | 69.14 | 92.89 | 56.99 |
| 2 | WBPH | ASD7 | 6 | 81.55 | 92.04 | 68.69 |
| 2 | WBPH | ASD7 | 7 | 89.28 | 95.75 | 79.35 |
| 2 | WBPH | ASD7 | 8 | 97.01 | 99.46 | 90.01 |
| 2 | WBPH | ASD7 | 9 | 101.46 | 100.58 | 92.14 |
| 2 | WBPH | ASD7 | 10 | 105.91 | 101.70 | 94.26 |
| 3 | BPH | ASD7 | 1 | 88.42 | 124.06 | 58.24 |
| 3 | BPH | ASD7 | 2 | 82.30 | 117.49 | 59.99 |
| 3 | BPH | ASD7 | 3 | 76.17 | 110.92 | 61.74 |
| 3 | BPH | ASD7 | 4 | 79.83 | 112.63 | 57.80 |
| 3 | BPH | ASD7 | 5 | 83.49 | 114.34 | 53.85 |
| 3 | BPH | ASD7 | 6 | 66.09 | 92.78 | 56.56 |
| 3 | BPH | ASD7 | 7 | 81.27 | 101.33 | 72.95 |
| 3 | BPH | ASD7 | 8 | 96.45 | 109.88 | 89.33 |
| 3 | BPH | ASD7 | 9 | 124.59 | 128.93 | 110.25 |
| 3 | BPH | ASD7 | 10 | 137.42 | 137.79 | 132.87 |
| 3 | CONTROL | ASD7 | 1 | 98.18 | 134.06 | 61.06 |
| 3 | CONTROL | ASD7 | 2 | 97.18 | 133.77 | 66.46 |
| 3 | CONTROL | ASD7 | 3 | 96.17 | 133.47 | 71.85 |
| 3 | CONTROL | ASD7 | 4 | 94.79 | 128.55 | 62.67 |
| 3 | CONTROL | ASD7 | 5 | 93.41 | 123.63 | 53.48 |
| 3 | CONTROL | ASD7 | 6 | 94.09 | 124.44 | 53.80 |
| 3 | CONTROL | ASD7 | 7 | 107.22 | 134.46 | 61.55 |
| 3 | CONTROL | ASD7 | 8 | 120.34 | 144.48 | 69.30 |
| 3 | CONTROL | ASD7 | 9 | 115.64 | 149.64 | 75.30 |
| 3 | CONTROL | ASD7 | 10 | 126.01 | 162.36 | 76.15 |
| 3 | WBPH | ASD7 | 1 | 93.09 | 129.27 | 64.85 |
| 3 | WBPH | ASD7 | 2 | 88.66 | 117.28 | 64.23 |
| 3 | WBPH | ASD7 | 3 | 84.23 | 105.29 | 63.60 |
| 3 | WBPH | ASD7 | 4 | 81.27 | 98.63 | 66.38 |
| 3 | WBPH | ASD7 | 5 | 78.30 | 91.97 | 69.16 |
| 3 | WBPH | ASD7 | 6 | 98.66 | 107.63 | 75.13 |
| 3 | WBPH | ASD7 | 7 | 100.00 | 107.63 | 81.43 |
| 3 | WBPH | ASD7 | 8 | 101.34 | 107.62 | 87.73 |
| 3 | WBPH | ASD7 | 9 | 94.69 | 96.31 | 80.70 |
| 3 | WBPH | ASD7 | 10 | 124.12 | 127.73 | 109.90 |
| 1 | BPH | Asiminori | 1 | 59.53 | 92.77 | 57.70 |
| 1 | BPH | Asiminori | 2 | 63.74 | 98.81 | 67.67 |
| 1 | BPH | Asiminori | 3 | 81.38 | 117.76 | 73.59 |
| 1 | BPH | Asiminori | 4 | 76.27 | 111.23 | 70.73 |
| 1 | BPH | Asiminori | 5 | 77.38 | 107.24 | 57.93 |
| 1 | BPH | Asiminori | 6 | 64.12 | 86.90 | 67.61 |
| 1 | BPH | Asiminori | 7 | 88.26 | 106.11 | 93.84 |
| 1 | BPH | Asiminori | 8 | 118.74 | 127.04 | 122.19 |
| 1 | BPH | Asiminori | 9 | 127.41 | 121.50 | 112.86 |
| 1 | BPH | Asiminori | 10 | 118.50 | 118.15 | 115.07 |
| 1 | CONTROL | Asiminori | 1 | 72.54 | 109.17 | 58.45 |
| 1 | CONTROL | Asiminori | 2 | 78.40 | 108.00 | 70.48 |
| 1 | CONTROL | Asiminori | 3 | 77.51 | 113.39 | 59.69 |
| 1 | CONTROL | Asiminori | 4 | 69.00 | 102.11 | 57.56 |
| 1 | CONTROL | Asiminori | 5 | 68.11 | 93.78 | 45.43 |
| 1 | CONTROL | Asiminori | 6 | 61.33 | 92.50 | 52.79 |
| 1 | CONTROL | Asiminori | 7 | 68.17 | 93.32 | 47.29 |
| 1 | CONTROL | Asiminori | 8 | 73.94 | 106.92 | 57.87 |
| 1 | CONTROL | Asiminori | 9 | 87.32 | 113.36 | 69.15 |
| 1 | CONTROL | Asiminori | 10 | 64.30 | 95.98 | 58.40 |
| 1 | WBPH | Asiminori | 1 | 82.68 | 111.98 | 57.80 |
| 1 | WBPH | Asiminori | 2 | 74.79 | 100.81 | 55.63 |
| 1 | WBPH | Asiminori | 3 | 82.25 | 112.81 | 67.97 |
| 1 | WBPH | Asiminori | 4 | 85.58 | 115.79 | 71.27 |
| 1 | WBPH | Asiminori | 5 | 89.49 | 112.30 | 66.89 |
| 1 | WBPH | Asiminori | 6 | 84.01 | 109.66 | 80.38 |
| 1 | WBPH | Asiminori | 7 | 85.86 | 109.42 | 79.21 |
| 1 | WBPH | Asiminori | 8 | 99.88 | 124.21 | 93.70 |
| 1 | WBPH | Asiminori | 9 | 83.15 | 98.61 | 69.50 |
| 1 | WBPH | Asiminori | 10 | 70.27 | 88.42 | 69.45 |
| 2 | BPH | Asiminori | 1 | 66.06 | 95.60 | 66.14 |
| 2 | BPH | Asiminori | 2 | 62.01 | 83.68 | 63.56 |
| 2 | BPH | Asiminori | 3 | 57.95 | 71.76 | 60.98 |
| 2 | BPH | Asiminori | 4 | 84.12 | 91.99 | 81.14 |
| 2 | BPH | Asiminori | 5 | 110.29 | 112.22 | 101.30 |
| 2 | BPH | Asiminori | 6 | 108.79 | 104.96 | 100.52 |
| 2 | BPH | Asiminori | 7 | 117.32 | 112.79 | 108.01 |
| 2 | BPH | Asiminori | 8 | 125.85 | 120.62 | 115.50 |
| 2 | BPH | Asiminori | 9 | 114.73 | 111.29 | 108.29 |
| 2 | BPH | Asiminori | 10 | 103.60 | 101.96 | 101.08 |
| 2 | CONTROL | Asiminori | 1 | 79.63 | 109.04 | 55.41 |
| 2 | CONTROL | Asiminori | 2 | 75.57 | 105.55 | 58.29 |
| 2 | CONTROL | Asiminori | 3 | 71.51 | 102.05 | 61.17 |
| 2 | CONTROL | Asiminori | 4 | 72.38 | 105.12 | 59.91 |
| 2 | CONTROL | Asiminori | 5 | 73.25 | 108.18 | 58.64 |
| 2 | CONTROL | Asiminori | 6 | 88.65 | 116.53 | 55.53 |
| 2 | CONTROL | Asiminori | 7 | 94.66 | 122.72 | 61.03 |
| 2 | CONTROL | Asiminori | 8 | 100.66 | 128.91 | 66.53 |
| 2 | CONTROL | Asiminori | 9 | 91.98 | 118.23 | 56.86 |
| 2 | CONTROL | Asiminori | 10 | 83.29 | 107.55 | 47.19 |
| 2 | WBPH | Asiminori | 1 | 61.48 | 89.92 | 61.21 |
| 2 | WBPH | Asiminori | 2 | 61.93 | 85.81 | 62.57 |
| 2 | WBPH | Asiminori | 3 | 62.38 | 81.69 | 63.92 |
| 2 | WBPH | Asiminori | 4 | 77.03 | 91.11 | 76.98 |
| 2 | WBPH | Asiminori | 5 | 91.68 | 100.53 | 90.04 |
| 2 | WBPH | Asiminori | 6 | 97.61 | 97.58 | 95.44 |
| 2 | WBPH | Asiminori | 7 | 100.23 | 101.02 | 99.96 |
| 2 | WBPH | Asiminori | 8 | 102.85 | 104.45 | 104.47 |
| 2 | WBPH | Asiminori | 9 | 110.21 | 109.18 | 107.31 |
| 2 | WBPH | Asiminori | 10 | 117.57 | 113.90 | 110.14 |
| 3 | BPH | Asiminori | 1 | 66.10 | 99.33 | 66.91 |
| 3 | BPH | Asiminori | 2 | 64.55 | 94.41 | 62.96 |
| 3 | BPH | Asiminori | 3 | 62.99 | 89.49 | 59.01 |
| 3 | BPH | Asiminori | 4 | 66.76 | 93.61 | 66.72 |
| 3 | BPH | Asiminori | 5 | 70.52 | 97.72 | 74.43 |
| 3 | BPH | Asiminori | 6 | 70.35 | 91.98 | 72.64 |
| 3 | BPH | Asiminori | 7 | 78.24 | 97.92 | 80.51 |
| 3 | BPH | Asiminori | 8 | 86.13 | 103.85 | 88.38 |
| 3 | BPH | Asiminori | 9 | 94.35 | 107.63 | 95.54 |
| 3 | BPH | Asiminori | 10 | 129.65 | 145.55 | 126.00 |
| 3 | CONTROL | Asiminori | 1 | 91.59 | 129.46 | 57.42 |
| 3 | CONTROL | Asiminori | 2 | 80.95 | 117.48 | 54.50 |
| 3 | CONTROL | Asiminori | 3 | 70.31 | 105.50 | 51.58 |
| 3 | CONTROL | Asiminori | 4 | 72.12 | 106.36 | 49.52 |
| 3 | CONTROL | Asiminori | 5 | 73.92 | 107.22 | 47.46 |
| 3 | CONTROL | Asiminori | 6 | 70.90 | 103.58 | 46.68 |
| 3 | CONTROL | Asiminori | 7 | 75.97 | 109.88 | 49.90 |
| 3 | CONTROL | Asiminori | 8 | 81.03 | 116.17 | 53.12 |
| 3 | CONTROL | Asiminori | 9 | 95.98 | 126.18 | 47.23 |
| 3 | CONTROL | Asiminori | 10 | 94.75 | 133.82 | 58.85 |
| 3 | WBPH | Asiminori | 1 | 60.67 | 94.43 | 58.89 |
| 3 | WBPH | Asiminori | 2 | 66.24 | 96.97 | 57.87 |
| 3 | WBPH | Asiminori | 3 | 71.81 | 99.51 | 56.85 |
| 3 | WBPH | Asiminori | 4 | 68.63 | 97.76 | 57.91 |
| 3 | WBPH | Asiminori | 5 | 65.44 | 96.00 | 58.96 |
| 3 | WBPH | Asiminori | 6 | 69.04 | 97.06 | 55.83 |
| 3 | WBPH | Asiminori | 7 | 68.72 | 96.49 | 57.19 |
| 3 | WBPH | Asiminori | 8 | 68.40 | 95.92 | 58.54 |
| 3 | WBPH | Asiminori | 9 | 87.87 | 116.37 | 60.67 |
| 3 | WBPH | Asiminori | 10 | 97.66 | 131.96 | 76.14 |
| 1 | BPH | Babawee | 1 | 122.65 | 154.33 | 72.87 |
| 1 | BPH | Babawee | 2 | 84.58 | 120.31 | 65.34 |
| 1 | BPH | Babawee | 3 | 131.92 | 162.89 | 78.18 |
| 1 | BPH | Babawee | 4 | 99.26 | 133.00 | 67.74 |
| 1 | BPH | Babawee | 5 | 91.41 | 118.37 | 61.44 |
| 1 | BPH | Babawee | 6 | 109.96 | 136.27 | 74.27 |
| 1 | BPH | Babawee | 7 | 93.02 | 121.96 | 70.42 |
| 1 | BPH | Babawee | 8 | 120.65 | 143.54 | 76.51 |
| 1 | BPH | Babawee | 9 | 109.07 | 130.57 | 80.00 |
| 1 | BPH | Babawee | 10 | 116.02 | 139.05 | 95.11 |
| 1 | CONTROL | Babawee | 1 | 98.40 | 135.89 | 66.24 |
| 1 | CONTROL | Babawee | 2 | 93.30 | 131.19 | 72.69 |
| 1 | CONTROL | Babawee | 3 | 99.24 | 134.31 | 68.76 |
| 1 | CONTROL | Babawee | 4 | 85.65 | 118.33 | 64.44 |
| 1 | CONTROL | Babawee | 5 | 94.16 | 129.32 | 72.38 |
| 1 | CONTROL | Babawee | 6 | 82.43 | 114.41 | 65.34 |
| 1 | CONTROL | Babawee | 7 | 103.18 | 133.98 | 66.55 |
| 1 | CONTROL | Babawee | 8 | 96.72 | 126.22 | 66.77 |
| 1 | CONTROL | Babawee | 9 | 111.44 | 142.35 | 72.60 |
| 1 | CONTROL | Babawee | 10 | 109.24 | 137.12 | 77.02 |
| 1 | WBPH | Babawee | 1 | 113.82 | 149.98 | 78.89 |
| 1 | WBPH | Babawee | 2 | 94.99 | 122.95 | 71.87 |
| 1 | WBPH | Babawee | 3 | 112.44 | 130.44 | 67.19 |
| 1 | WBPH | Babawee | 4 | 107.84 | 132.52 | 76.17 |
| 1 | WBPH | Babawee | 5 | 129.51 | 136.86 | 80.51 |
| 1 | WBPH | Babawee | 6 | 112.79 | 121.34 | 88.26 |
| 1 | WBPH | Babawee | 7 | 90.55 | 101.69 | 83.62 |
| 1 | WBPH | Babawee | 8 | 119.99 | 130.54 | 110.51 |
| 1 | WBPH | Babawee | 9 | 101.08 | 112.09 | 94.02 |
| 1 | WBPH | Babawee | 10 | 95.32 | 107.12 | 91.59 |
| 2 | BPH | Babawee | 1 | 83.21 | 109.13 | 46.73 |
| 2 | BPH | Babawee | 2 | 78.09 | 103.78 | 47.43 |
| 2 | BPH | Babawee | 3 | 72.96 | 98.43 | 48.12 |
| 2 | BPH | Babawee | 4 | 73.37 | 97.72 | 49.19 |
| 2 | BPH | Babawee | 5 | 73.78 | 97.00 | 50.26 |
| 2 | BPH | Babawee | 6 | 79.01 | 101.62 | 64.66 |
| 2 | BPH | Babawee | 7 | 86.62 | 102.70 | 74.49 |
| 2 | BPH | Babawee | 8 | 94.23 | 103.78 | 84.32 |
| 2 | BPH | Babawee | 9 | 93.89 | 105.76 | 89.96 |
| 2 | BPH | Babawee | 10 | 93.55 | 107.74 | 95.59 |
| 2 | CONTROL | Babawee | 1 | 95.54 | 123.57 | 47.40 |
| 2 | CONTROL | Babawee | 2 | 90.47 | 124.91 | 64.20 |
| 2 | CONTROL | Babawee | 3 | 85.40 | 126.24 | 81.00 |
| 2 | CONTROL | Babawee | 4 | 84.06 | 116.93 | 61.34 |
| 2 | CONTROL | Babawee | 5 | 82.71 | 107.62 | 41.68 |
| 2 | CONTROL | Babawee | 6 | 88.67 | 121.51 | 50.13 |
| 2 | CONTROL | Babawee | 7 | 94.09 | 127.13 | 50.90 |
| 2 | CONTROL | Babawee | 8 | 99.50 | 132.74 | 51.67 |
| 2 | CONTROL | Babawee | 9 | 106.12 | 140.44 | 58.45 |
| 2 | CONTROL | Babawee | 10 | 112.73 | 148.14 | 65.22 |
| 2 | WBPH | Babawee | 1 | 66.96 | 95.25 | 54.26 |
| 2 | WBPH | Babawee | 2 | 62.95 | 90.33 | 54.06 |
| 2 | WBPH | Babawee | 3 | 58.93 | 85.41 | 53.85 |
| 2 | WBPH | Babawee | 4 | 61.60 | 87.49 | 54.03 |
| 2 | WBPH | Babawee | 5 | 64.26 | 89.56 | 54.21 |
| 2 | WBPH | Babawee | 6 | 70.10 | 88.50 | 59.19 |
| 2 | WBPH | Babawee | 7 | 77.68 | 90.51 | 64.02 |
| 2 | WBPH | Babawee | 8 | 85.25 | 92.52 | 68.85 |
| 2 | WBPH | Babawee | 9 | 86.15 | 91.91 | 71.84 |
| 2 | WBPH | Babawee | 10 | 87.05 | 91.30 | 74.82 |
| 3 | BPH | Babawee | 1 | 92.39 | 127.86 | 52.36 |
| 3 | BPH | Babawee | 2 | 98.62 | 129.51 | 54.06 |
| 3 | BPH | Babawee | 3 | 104.84 | 131.15 | 55.76 |
| 3 | BPH | Babawee | 4 | 96.37 | 123.19 | 53.90 |
| 3 | BPH | Babawee | 5 | 87.90 | 115.23 | 52.04 |
| 3 | BPH | Babawee | 6 | 89.94 | 115.48 | 53.50 |
| 3 | BPH | Babawee | 7 | 85.16 | 110.63 | 56.49 |
| 3 | BPH | Babawee | 8 | 80.38 | 105.77 | 59.48 |
| 3 | BPH | Babawee | 9 | 85.30 | 110.22 | 65.27 |
| 3 | BPH | Babawee | 10 | 108.64 | 134.59 | 87.41 |
| 3 | CONTROL | Babawee | 1 | 109.08 | 137.32 | 52.17 |
| 3 | CONTROL | Babawee | 2 | 114.82 | 141.98 | 53.24 |
| 3 | CONTROL | Babawee | 3 | 120.56 | 146.64 | 54.30 |
| 3 | CONTROL | Babawee | 4 | 110.47 | 139.69 | 52.23 |
| 3 | CONTROL | Babawee | 5 | 100.38 | 132.73 | 50.15 |
| 3 | CONTROL | Babawee | 6 | 91.91 | 124.52 | 44.79 |
| 3 | CONTROL | Babawee | 7 | 96.60 | 129.84 | 50.94 |
| 3 | CONTROL | Babawee | 8 | 101.29 | 135.16 | 57.08 |
| 3 | CONTROL | Babawee | 9 | 109.81 | 142.80 | 42.86 |
| 3 | CONTROL | Babawee | 10 | 129.79 | 167.39 | 64.79 |
| 3 | WBPH | Babawee | 1 | 101.67 | 131.64 | 57.27 |
| 3 | WBPH | Babawee | 2 | 96.29 | 127.51 | 56.48 |
| 3 | WBPH | Babawee | 3 | 90.90 | 123.37 | 55.69 |
| 3 | WBPH | Babawee | 4 | 89.52 | 121.05 | 54.54 |
| 3 | WBPH | Babawee | 5 | 88.14 | 118.72 | 53.39 |
| 3 | WBPH | Babawee | 6 | 105.35 | 131.99 | 64.12 |
| 3 | WBPH | Babawee | 7 | 111.49 | 133.00 | 66.48 |
| 3 | WBPH | Babawee | 8 | 117.63 | 134.01 | 68.84 |
| 3 | WBPH | Babawee | 9 | 122.97 | 138.41 | 77.40 |
| 3 | WBPH | Babawee | 10 | 135.95 | 149.64 | 92.68 |
| 1 | BPH | Balamawee | 1 | 87.95 | 122.64 | 71.70 |
| 1 | BPH | Balamawee | 2 | 74.67 | 111.05 | 73.30 |
| 1 | BPH | Balamawee | 3 | 73.82 | 105.90 | 71.24 |
| 1 | BPH | Balamawee | 4 | 85.62 | 119.37 | 76.81 |
| 1 | BPH | Balamawee | 5 | 81.93 | 114.04 | 72.10 |
| 1 | BPH | Balamawee | 6 | 83.31 | 115.22 | 82.02 |
| 1 | BPH | Balamawee | 7 | 111.26 | 141.05 | 76.32 |
| 1 | BPH | Balamawee | 8 | 99.19 | 134.56 | 99.05 |
| 1 | BPH | Balamawee | 9 | 91.56 | 117.59 | 83.66 |
| 1 | BPH | Balamawee | 10 | 91.10 | 118.42 | 87.77 |
| 1 | CONTROL | Balamawee | 1 | 124.96 | 156.05 | 82.20 |
| 1 | CONTROL | Balamawee | 2 | 106.04 | 145.06 | 89.43 |
| 1 | CONTROL | Balamawee | 3 | 112.34 | 145.21 | 68.53 |
| 1 | CONTROL | Balamawee | 4 | 84.46 | 116.16 | 69.93 |
| 1 | CONTROL | Balamawee | 5 | 121.14 | 144.70 | 70.01 |
| 1 | CONTROL | Balamawee | 6 | 85.30 | 114.46 | 62.19 |
| 1 | CONTROL | Balamawee | 7 | 99.76 | 130.17 | 63.42 |
| 1 | CONTROL | Balamawee | 8 | 105.78 | 136.59 | 67.95 |
| 1 | CONTROL | Balamawee | 9 | 123.91 | 144.87 | 68.13 |
| 1 | CONTROL | Balamawee | 10 | 106.42 | 135.62 | 67.48 |
| 1 | WBPH | Balamawee | 1 | 105.16 | 139.98 | 68.00 |
| 1 | WBPH | Balamawee | 2 | 98.46 | 123.20 | 68.01 |
| 1 | WBPH | Balamawee | 3 | 130.11 | 158.28 | 70.62 |
| 1 | WBPH | Balamawee | 4 | 121.88 | 149.88 | 69.72 |
| 1 | WBPH | Balamawee | 5 | 102.62 | 125.53 | 52.95 |
| 1 | WBPH | Balamawee | 6 | 102.19 | 125.96 | 70.26 |
| 1 | WBPH | Balamawee | 7 | 112.30 | 130.25 | 66.76 |
| 1 | WBPH | Balamawee | 8 | 100.36 | 128.87 | 78.12 |
| 1 | WBPH | Balamawee | 9 | 119.96 | 136.29 | 74.03 |
| 1 | WBPH | Balamawee | 10 | 95.86 | 121.02 | 74.79 |
| 2 | BPH | Balamawee | 1 | 68.42 | 100.85 | 56.11 |
| 2 | BPH | Balamawee | 2 | 66.52 | 97.60 | 55.95 |
| 2 | BPH | Balamawee | 3 | 64.61 | 94.35 | 55.78 |
| 2 | BPH | Balamawee | 4 | 70.30 | 100.46 | 54.13 |
| 2 | BPH | Balamawee | 5 | 75.98 | 106.56 | 52.48 |
| 2 | BPH | Balamawee | 6 | 78.61 | 106.81 | 54.27 |
| 2 | BPH | Balamawee | 7 | 76.53 | 104.38 | 59.04 |
| 2 | BPH | Balamawee | 8 | 74.44 | 101.95 | 63.81 |
| 2 | BPH | Balamawee | 9 | 74.40 | 100.18 | 63.80 |
| 2 | BPH | Balamawee | 10 | 74.35 | 98.40 | 63.78 |
| 2 | CONTROL | Balamawee | 1 | 89.10 | 113.44 | 55.66 |
| 2 | CONTROL | Balamawee | 2 | 81.55 | 112.70 | 64.82 |
| 2 | CONTROL | Balamawee | 3 | 73.99 | 111.96 | 73.97 |
| 2 | CONTROL | Balamawee | 4 | 82.08 | 119.09 | 66.28 |
| 2 | CONTROL | Balamawee | 5 | 90.16 | 126.21 | 58.59 |
| 2 | CONTROL | Balamawee | 6 | 84.35 | 117.47 | 51.97 |
| 2 | CONTROL | Balamawee | 7 | 100.92 | 131.07 | 57.36 |
| 2 | CONTROL | Balamawee | 8 | 117.49 | 144.66 | 62.74 |
| 2 | CONTROL | Balamawee | 9 | 103.57 | 133.12 | 57.29 |
| 2 | CONTROL | Balamawee | 10 | 89.65 | 121.58 | 51.83 |
| 2 | WBPH | Balamawee | 1 | 81.04 | 110.68 | 58.71 |
| 2 | WBPH | Balamawee | 2 | 74.53 | 105.72 | 55.56 |
| 2 | WBPH | Balamawee | 3 | 68.02 | 100.75 | 52.41 |
| 2 | WBPH | Balamawee | 4 | 76.75 | 108.68 | 55.85 |
| 2 | WBPH | Balamawee | 5 | 85.48 | 116.61 | 59.29 |
| 2 | WBPH | Balamawee | 6 | 83.67 | 107.21 | 48.84 |
| 2 | WBPH | Balamawee | 7 | 91.59 | 117.04 | 55.54 |
| 2 | WBPH | Balamawee | 8 | 99.50 | 126.86 | 62.23 |
| 2 | WBPH | Balamawee | 9 | 92.52 | 122.67 | 65.32 |
| 2 | WBPH | Balamawee | 10 | 85.54 | 118.48 | 68.41 |
| 3 | BPH | Balamawee | 1 | 105.58 | 135.61 | 71.75 |
| 3 | BPH | Balamawee | 2 | 98.37 | 131.76 | 72.03 |
| 3 | BPH | Balamawee | 3 | 91.15 | 127.90 | 72.31 |
| 3 | BPH | Balamawee | 4 | 86.31 | 120.56 | 66.14 |
| 3 | BPH | Balamawee | 5 | 81.47 | 113.22 | 59.97 |
| 3 | BPH | Balamawee | 6 | 85.87 | 117.65 | 61.08 |
| 3 | BPH | Balamawee | 7 | 85.91 | 113.96 | 64.27 |
| 3 | BPH | Balamawee | 8 | 85.94 | 110.27 | 67.46 |
| 3 | BPH | Balamawee | 9 | 82.28 | 105.17 | 85.14 |
| 3 | BPH | Balamawee | 10 | 103.45 | 132.07 | 97.80 |
| 3 | CONTROL | Balamawee | 1 | 92.21 | 122.08 | 64.65 |
| 3 | CONTROL | Balamawee | 2 | 98.27 | 131.20 | 67.22 |
| 3 | CONTROL | Balamawee | 3 | 104.33 | 140.32 | 69.79 |
| 3 | CONTROL | Balamawee | 4 | 99.87 | 135.13 | 64.77 |
| 3 | CONTROL | Balamawee | 5 | 95.41 | 129.93 | 59.74 |
| 3 | CONTROL | Balamawee | 6 | 98.99 | 130.19 | 52.61 |
| 3 | CONTROL | Balamawee | 7 | 105.55 | 139.11 | 59.07 |
| 3 | CONTROL | Balamawee | 8 | 112.10 | 148.02 | 65.53 |
| 3 | CONTROL | Balamawee | 9 | 114.80 | 149.09 | 72.03 |
| 3 | CONTROL | Balamawee | 10 | 120.04 | 156.74 | 84.97 |
| 3 | WBPH | Balamawee | 1 | 82.82 | 118.31 | 63.07 |
| 3 | WBPH | Balamawee | 2 | 89.85 | 121.11 | 60.60 |
| 3 | WBPH | Balamawee | 3 | 96.87 | 123.91 | 58.13 |
| 3 | WBPH | Balamawee | 4 | 87.82 | 116.31 | 57.79 |
| 3 | WBPH | Balamawee | 5 | 78.76 | 108.71 | 57.45 |
| 3 | WBPH | Balamawee | 6 | 87.42 | 117.55 | 59.53 |
| 3 | WBPH | Balamawee | 7 | 92.79 | 121.47 | 60.03 |
| 3 | WBPH | Balamawee | 8 | 98.15 | 125.39 | 60.53 |
| 3 | WBPH | Balamawee | 9 | 120.34 | 144.45 | 67.46 |
| 3 | WBPH | Balamawee | 10 | 121.78 | 141.90 | 84.79 |
| 1 | BPH | Chinsaba | 1 | 110.39 | 143.10 | 81.35 |
| 1 | BPH | Chinsaba | 2 | 90.57 | 118.51 | 71.22 |
| 1 | BPH | Chinsaba | 3 | 97.69 | 124.80 | 94.38 |
| 1 | BPH | Chinsaba | 4 | 97.69 | 108.73 | 102.09 |
| 1 | BPH | Chinsaba | 5 | 124.03 | 115.67 | 103.37 |
| 1 | BPH | Chinsaba | 6 | 114.71 | 113.49 | 109.30 |
| 1 | BPH | Chinsaba | 7 | 122.26 | 119.02 | 115.83 |
| 1 | BPH | Chinsaba | 8 | 105.41 | 100.83 | 95.43 |
| 1 | BPH | Chinsaba | 9 | 109.64 | 105.13 | 101.03 |
| 1 | BPH | Chinsaba | 10 | 95.79 | 90.36 | 82.68 |
| 1 | CONTROL | Chinsaba | 1 | 87.38 | 120.99 | 62.51 |
| 1 | CONTROL | Chinsaba | 2 | 85.55 | 118.82 | 76.09 |
| 1 | CONTROL | Chinsaba | 3 | 103.35 | 137.30 | 76.37 |
| 1 | CONTROL | Chinsaba | 4 | 93.01 | 118.97 | 69.32 |
| 1 | CONTROL | Chinsaba | 5 | 87.55 | 118.41 | 66.79 |
| 1 | CONTROL | Chinsaba | 6 | 78.79 | 109.79 | 69.92 |
| 1 | CONTROL | Chinsaba | 7 | 102.56 | 134.79 | 78.29 |
| 1 | CONTROL | Chinsaba | 8 | 93.18 | 127.18 | 79.99 |
| 1 | CONTROL | Chinsaba | 9 | 124.44 | 158.24 | 106.78 |
| 1 | CONTROL | Chinsaba | 10 | 99.33 | 131.38 | 78.39 |
| 1 | WBPH | Chinsaba | 1 | 126.20 | 153.99 | 73.89 |
| 1 | WBPH | Chinsaba | 2 | 108.06 | 132.25 | 74.79 |
| 1 | WBPH | Chinsaba | 3 | 129.43 | 149.98 | 84.25 |
| 1 | WBPH | Chinsaba | 4 | 119.92 | 147.61 | 81.10 |
| 1 | WBPH | Chinsaba | 5 | 121.01 | 145.32 | 77.60 |
| 1 | WBPH | Chinsaba | 6 | 125.75 | 146.71 | 76.97 |
| 1 | WBPH | Chinsaba | 7 | 137.42 | 156.67 | 86.72 |
| 1 | WBPH | Chinsaba | 8 | 130.64 | 149.05 | 99.70 |
| 1 | WBPH | Chinsaba | 9 | 123.19 | 133.03 | 80.01 |
| 1 | WBPH | Chinsaba | 10 | 130.85 | 141.09 | 100.71 |
| 2 | BPH | Chinsaba | 1 | 92.29 | 123.38 | 69.67 |
| 2 | BPH | Chinsaba | 2 | 87.86 | 115.48 | 66.76 |
| 2 | BPH | Chinsaba | 3 | 83.43 | 107.58 | 63.85 |
| 2 | BPH | Chinsaba | 4 | 83.48 | 98.65 | 70.19 |
| 2 | BPH | Chinsaba | 5 | 83.52 | 89.71 | 76.53 |
| 2 | BPH | Chinsaba | 6 | 111.15 | 107.13 | 101.46 |
| 2 | BPH | Chinsaba | 7 | 120.75 | 115.80 | 110.72 |
| 2 | BPH | Chinsaba | 8 | 130.34 | 124.46 | 119.97 |
| 2 | BPH | Chinsaba | 9 | 118.86 | 113.25 | 108.59 |
| 2 | BPH | Chinsaba | 10 | 107.38 | 102.04 | 97.21 |
| 2 | CONTROL | Chinsaba | 1 | 98.23 | 125.34 | 55.72 |
| 2 | CONTROL | Chinsaba | 2 | 89.36 | 120.49 | 58.01 |
| 2 | CONTROL | Chinsaba | 3 | 80.49 | 115.63 | 60.29 |
| 2 | CONTROL | Chinsaba | 4 | 94.27 | 130.00 | 57.98 |
| 2 | CONTROL | Chinsaba | 5 | 108.05 | 144.36 | 55.67 |
| 2 | CONTROL | Chinsaba | 6 | 103.59 | 138.95 | 59.00 |
| 2 | CONTROL | Chinsaba | 7 | 106.35 | 142.56 | 65.37 |
| 2 | CONTROL | Chinsaba | 8 | 109.10 | 146.16 | 71.73 |
| 2 | CONTROL | Chinsaba | 9 | 104.61 | 139.75 | 63.83 |
| 2 | CONTROL | Chinsaba | 10 | 100.11 | 133.34 | 55.93 |
| 2 | WBPH | Chinsaba | 1 | 86.57 | 112.15 | 50.03 |
| 2 | WBPH | Chinsaba | 2 | 88.33 | 114.02 | 52.19 |
| 2 | WBPH | Chinsaba | 3 | 90.09 | 115.89 | 54.34 |
| 2 | WBPH | Chinsaba | 4 | 94.01 | 118.89 | 53.82 |
| 2 | WBPH | Chinsaba | 5 | 97.93 | 121.88 | 53.29 |
| 2 | WBPH | Chinsaba | 6 | 87.49 | 102.75 | 51.82 |
| 2 | WBPH | Chinsaba | 7 | 99.50 | 115.87 | 62.41 |
| 2 | WBPH | Chinsaba | 8 | 111.50 | 128.99 | 72.99 |
| 2 | WBPH | Chinsaba | 9 | 111.36 | 125.59 | 76.17 |
| 2 | WBPH | Chinsaba | 10 | 111.21 | 122.19 | 79.34 |
| 3 | BPH | Chinsaba | 1 | 130.79 | 160.62 | 69.73 |
| 3 | BPH | Chinsaba | 2 | 118.00 | 150.59 | 65.49 |
| 3 | BPH | Chinsaba | 3 | 105.20 | 140.55 | 61.25 |
| 3 | BPH | Chinsaba | 4 | 99.41 | 131.09 | 60.63 |
| 3 | BPH | Chinsaba | 5 | 93.61 | 121.63 | 60.00 |
| 3 | BPH | Chinsaba | 6 | 108.40 | 129.63 | 62.32 |
| 3 | BPH | Chinsaba | 7 | 108.05 | 129.24 | 66.75 |
| 3 | BPH | Chinsaba | 8 | 107.70 | 128.84 | 71.17 |
| 3 | BPH | Chinsaba | 9 | 100.08 | 100.96 | 89.30 |
| 3 | BPH | Chinsaba | 10 | 148.30 | 142.46 | 134.05 |
| 3 | CONTROL | Chinsaba | 1 | 103.87 | 139.66 | 57.56 |
| 3 | CONTROL | Chinsaba | 2 | 117.38 | 154.30 | 68.74 |
| 3 | CONTROL | Chinsaba | 3 | 130.89 | 168.93 | 79.92 |
| 3 | CONTROL | Chinsaba | 4 | 122.61 | 152.95 | 67.26 |
| 3 | CONTROL | Chinsaba | 5 | 114.32 | 136.96 | 54.60 |
| 3 | CONTROL | Chinsaba | 6 | 100.75 | 133.39 | 54.86 |
| 3 | CONTROL | Chinsaba | 7 | 109.08 | 141.89 | 57.64 |
| 3 | CONTROL | Chinsaba | 8 | 117.41 | 150.38 | 60.41 |
| 3 | CONTROL | Chinsaba | 9 | 139.99 | 163.70 | 63.40 |
| 3 | CONTROL | Chinsaba | 10 | 150.81 | 180.61 | 82.96 |
| 3 | WBPH | Chinsaba | 1 | 101.31 | 136.64 | 68.11 |
| 3 | WBPH | Chinsaba | 2 | 97.94 | 131.89 | 67.26 |
| 3 | WBPH | Chinsaba | 3 | 94.56 | 127.14 | 66.40 |
| 3 | WBPH | Chinsaba | 4 | 88.68 | 118.44 | 61.46 |
| 3 | WBPH | Chinsaba | 5 | 82.79 | 109.74 | 56.52 |
| 3 | WBPH | Chinsaba | 6 | 106.31 | 132.02 | 65.70 |
| 3 | WBPH | Chinsaba | 7 | 109.39 | 133.94 | 67.32 |
| 3 | WBPH | Chinsaba | 8 | 112.47 | 135.85 | 68.94 |
| 3 | WBPH | Chinsaba | 9 | 102.34 | 117.68 | 69.01 |
| 3 | WBPH | Chinsaba | 10 | 146.00 | 157.57 | 101.11 |
| 1 | BPH | DaHuaGu | 1 | 99.20 | 137.94 | 80.88 |
| 1 | BPH | DaHuaGu | 2 | 60.07 | 91.56 | 60.07 |
| 1 | BPH | DaHuaGu | 3 | 72.40 | 106.81 | 81.91 |
| 1 | BPH | DaHuaGu | 4 | 73.03 | 94.76 | 74.82 |
| 1 | BPH | DaHuaGu | 5 | 93.15 | 103.78 | 82.47 |
| 1 | BPH | DaHuaGu | 6 | 94.17 | 101.60 | 79.97 |
| 1 | BPH | DaHuaGu | 7 | 114.82 | 105.54 | 96.02 |
| 1 | BPH | DaHuaGu | 8 | 104.20 | 98.97 | 92.36 |
| 1 | BPH | DaHuaGu | 9 | 93.81 | 84.67 | 74.57 |
| 1 | BPH | DaHuaGu | 10 | 103.48 | 99.89 | 97.57 |
| 1 | CONTROL | DaHuaGu | 1 | 91.65 | 122.16 | 59.86 |
| 1 | CONTROL | DaHuaGu | 2 | 76.92 | 114.29 | 67.61 |
| 1 | CONTROL | DaHuaGu | 3 | 84.16 | 121.76 | 66.37 |
| 1 | CONTROL | DaHuaGu | 4 | 67.39 | 103.13 | 60.22 |
| 1 | CONTROL | DaHuaGu | 5 | 82.11 | 119.94 | 65.49 |
| 1 | CONTROL | DaHuaGu | 6 | 69.76 | 103.41 | 59.55 |
| 1 | CONTROL | DaHuaGu | 7 | 70.97 | 104.78 | 60.46 |
| 1 | CONTROL | DaHuaGu | 8 | 102.48 | 137.12 | 81.07 |
| 1 | CONTROL | DaHuaGu | 9 | 83.11 | 118.27 | 71.09 |
| 1 | CONTROL | DaHuaGu | 10 | 109.06 | 134.87 | 52.86 |
| 1 | WBPH | DaHuaGu | 1 | 99.07 | 128.14 | 74.20 |
| 1 | WBPH | DaHuaGu | 2 | 90.43 | 109.57 | 64.68 |
| 1 | WBPH | DaHuaGu | 3 | 112.13 | 132.88 | 71.56 |
| 1 | WBPH | DaHuaGu | 4 | 110.46 | 134.19 | 88.53 |
| 1 | WBPH | DaHuaGu | 5 | 92.02 | 104.91 | 76.94 |
| 1 | WBPH | DaHuaGu | 6 | 110.86 | 125.39 | 92.02 |
| 1 | WBPH | DaHuaGu | 7 | 105.85 | 112.53 | 90.90 |
| 1 | WBPH | DaHuaGu | 8 | 115.68 | 129.38 | 93.81 |
| 1 | WBPH | DaHuaGu | 9 | 87.57 | 93.11 | 79.13 |
| 1 | WBPH | DaHuaGu | 10 | 87.08 | 95.73 | 81.85 |
| 2 | BPH | DaHuaGu | 1 | 58.56 | 84.71 | 58.02 |
| 2 | BPH | DaHuaGu | 2 | 57.01 | 79.44 | 56.82 |
| 2 | BPH | DaHuaGu | 3 | 55.46 | 74.17 | 55.61 |
| 2 | BPH | DaHuaGu | 4 | 66.72 | 80.71 | 65.25 |
| 2 | BPH | DaHuaGu | 5 | 77.98 | 87.24 | 74.89 |
| 2 | BPH | DaHuaGu | 6 | 97.16 | 101.27 | 88.53 |
| 2 | BPH | DaHuaGu | 7 | 102.03 | 99.81 | 89.39 |
| 2 | BPH | DaHuaGu | 8 | 106.89 | 98.34 | 90.25 |
| 2 | BPH | DaHuaGu | 9 | 97.39 | 92.63 | 85.35 |
| 2 | BPH | DaHuaGu | 10 | 87.89 | 86.91 | 80.44 |
| 2 | CONTROL | DaHuaGu | 1 | 74.26 | 108.86 | 60.29 |
| 2 | CONTROL | DaHuaGu | 2 | 76.44 | 113.92 | 72.96 |
| 2 | CONTROL | DaHuaGu | 3 | 78.61 | 118.97 | 85.63 |
| 2 | CONTROL | DaHuaGu | 4 | 89.76 | 122.37 | 71.73 |
| 2 | CONTROL | DaHuaGu | 5 | 100.90 | 125.77 | 57.82 |
| 2 | CONTROL | DaHuaGu | 6 | 83.87 | 112.70 | 59.38 |
| 2 | CONTROL | DaHuaGu | 7 | 91.02 | 119.16 | 60.01 |
| 2 | CONTROL | DaHuaGu | 8 | 98.16 | 125.62 | 60.64 |
| 2 | CONTROL | DaHuaGu | 9 | 96.15 | 127.61 | 65.29 |
| 2 | CONTROL | DaHuaGu | 10 | 94.14 | 129.60 | 69.93 |
| 2 | WBPH | DaHuaGu | 1 | 71.38 | 101.77 | 65.67 |
| 2 | WBPH | DaHuaGu | 2 | 66.53 | 93.42 | 59.06 |
| 2 | WBPH | DaHuaGu | 3 | 61.67 | 85.06 | 52.44 |
| 2 | WBPH | DaHuaGu | 4 | 67.93 | 87.54 | 67.70 |
| 2 | WBPH | DaHuaGu | 5 | 74.19 | 90.02 | 82.95 |
| 2 | WBPH | DaHuaGu | 6 | 85.69 | 87.25 | 81.42 |
| 2 | WBPH | DaHuaGu | 7 | 94.30 | 94.41 | 90.56 |
| 2 | WBPH | DaHuaGu | 8 | 102.90 | 101.56 | 99.69 |
| 2 | WBPH | DaHuaGu | 9 | 103.20 | 102.33 | 100.83 |
| 2 | WBPH | DaHuaGu | 10 | 103.50 | 103.10 | 101.96 |
| 3 | BPH | DaHuaGu | 1 | 94.87 | 124.05 | 67.10 |
| 3 | BPH | DaHuaGu | 2 | 95.89 | 126.97 | 69.38 |
| 3 | BPH | DaHuaGu | 3 | 96.91 | 129.89 | 71.66 |
| 3 | BPH | DaHuaGu | 4 | 97.78 | 129.38 | 76.26 |
| 3 | BPH | DaHuaGu | 5 | 98.64 | 128.86 | 80.86 |
| 3 | BPH | DaHuaGu | 6 | 89.30 | 106.82 | 67.85 |
| 3 | BPH | DaHuaGu | 7 | 97.17 | 115.75 | 77.84 |
| 3 | BPH | DaHuaGu | 8 | 105.03 | 124.67 | 87.83 |
| 3 | BPH | DaHuaGu | 9 | 98.59 | 111.82 | 80.20 |
| 3 | BPH | DaHuaGu | 10 | 106.91 | 119.13 | 88.64 |
| 3 | CONTROL | DaHuaGu | 1 | 82.43 | 117.75 | 65.22 |
| 3 | CONTROL | DaHuaGu | 2 | 94.94 | 133.99 | 74.01 |
| 3 | CONTROL | DaHuaGu | 3 | 107.44 | 150.22 | 82.80 |
| 3 | CONTROL | DaHuaGu | 4 | 95.51 | 133.82 | 72.32 |
| 3 | CONTROL | DaHuaGu | 5 | 83.58 | 117.42 | 61.84 |
| 3 | CONTROL | DaHuaGu | 6 | 79.82 | 114.32 | 62.80 |
| 3 | CONTROL | DaHuaGu | 7 | 85.32 | 120.34 | 67.08 |
| 3 | CONTROL | DaHuaGu | 8 | 90.82 | 126.36 | 71.36 |
| 3 | CONTROL | DaHuaGu | 9 | 86.37 | 120.41 | 71.28 |
| 3 | CONTROL | DaHuaGu | 10 | 118.75 | 157.14 | 96.30 |
| 3 | WBPH | DaHuaGu | 1 | 76.09 | 106.24 | 62.79 |
| 3 | WBPH | DaHuaGu | 2 | 77.93 | 110.59 | 65.64 |
| 3 | WBPH | DaHuaGu | 3 | 79.76 | 114.93 | 68.49 |
| 3 | WBPH | DaHuaGu | 4 | 79.94 | 111.78 | 67.79 |
| 3 | WBPH | DaHuaGu | 5 | 80.12 | 108.62 | 67.09 |
| 3 | WBPH | DaHuaGu | 6 | 77.38 | 102.56 | 64.94 |
| 3 | WBPH | DaHuaGu | 7 | 93.45 | 113.37 | 67.50 |
| 3 | WBPH | DaHuaGu | 8 | 109.52 | 124.17 | 70.06 |
| 3 | WBPH | DaHuaGu | 9 | 93.02 | 113.75 | 70.00 |
| 3 | WBPH | DaHuaGu | 10 | 133.74 | 136.70 | 95.10 |
| 1 | BPH | IR22 | 1 | 96.55 | 131.66 | 78.43 |
| 1 | BPH | IR22 | 2 | 80.06 | 97.62 | 66.52 |
| 1 | BPH | IR22 | 3 | 87.04 | 100.14 | 70.62 |
| 1 | BPH | IR22 | 4 | 100.89 | 101.53 | 94.50 |
| 1 | BPH | IR22 | 5 | 114.54 | 110.48 | 106.57 |
| 1 | BPH | IR22 | 6 | 99.59 | 95.77 | 89.97 |
| 1 | BPH | IR22 | 7 | 101.15 | 97.40 | 92.67 |
| 1 | BPH | IR22 | 8 | 116.08 | 112.08 | 107.41 |
| 1 | BPH | IR22 | 9 | 113.56 | 106.29 | 99.78 |
| 1 | BPH | IR22 | 10 | 80.32 | 77.21 | 73.88 |
| 1 | CONTROL | IR22 | 1 | 79.25 | 105.90 | 50.96 |
| 1 | CONTROL | IR22 | 2 | 70.06 | 106.01 | 61.04 |
| 1 | CONTROL | IR22 | 3 | 78.80 | 112.76 | 56.80 |
| 1 | CONTROL | IR22 | 4 | 68.26 | 100.96 | 57.41 |
| 1 | CONTROL | IR22 | 5 | 89.31 | 125.81 | 67.51 |
| 1 | CONTROL | IR22 | 6 | 68.87 | 100.29 | 55.66 |
| 1 | CONTROL | IR22 | 7 | 67.01 | 96.56 | 51.78 |
| 1 | CONTROL | IR22 | 8 | 88.11 | 116.83 | 67.57 |
| 1 | CONTROL | IR22 | 9 | 113.58 | 137.25 | 66.50 |
| 1 | CONTROL | IR22 | 10 | 82.41 | 112.14 | 72.44 |
| 1 | WBPH | IR22 | 1 | 108.86 | 144.93 | 73.58 |
| 1 | WBPH | IR22 | 2 | 90.91 | 116.05 | 55.20 |
| 1 | WBPH | IR22 | 3 | 124.11 | 147.85 | 71.93 |
| 1 | WBPH | IR22 | 4 | 118.02 | 138.08 | 71.98 |
| 1 | WBPH | IR22 | 5 | 118.14 | 136.17 | 64.90 |
| 1 | WBPH | IR22 | 6 | 105.81 | 127.84 | 67.16 |
| 1 | WBPH | IR22 | 7 | 112.55 | 128.84 | 64.17 |
| 1 | WBPH | IR22 | 8 | 113.87 | 130.55 | 85.08 |
| 1 | WBPH | IR22 | 9 | 111.95 | 123.45 | 75.34 |
| 1 | WBPH | IR22 | 10 | 113.23 | 130.60 | 96.89 |
| 2 | BPH | IR22 | 1 | 55.63 | 77.29 | 40.81 |
| 2 | BPH | IR22 | 2 | 57.71 | 79.03 | 43.93 |
| 2 | BPH | IR22 | 3 | 59.78 | 80.77 | 47.05 |
| 2 | BPH | IR22 | 4 | 62.63 | 83.35 | 53.36 |
| 2 | BPH | IR22 | 5 | 65.48 | 85.93 | 59.66 |
| 2 | BPH | IR22 | 6 | 90.56 | 102.06 | 82.74 |
| 2 | BPH | IR22 | 7 | 98.72 | 104.44 | 87.63 |
| 2 | BPH | IR22 | 8 | 106.88 | 106.82 | 92.52 |
| 2 | BPH | IR22 | 9 | 108.61 | 106.22 | 94.87 |
| 2 | BPH | IR22 | 10 | 110.34 | 105.61 | 97.21 |
| 2 | CONTROL | IR22 | 1 | 78.48 | 104.83 | 48.68 |
| 2 | CONTROL | IR22 | 2 | 77.85 | 104.29 | 52.07 |
| 2 | CONTROL | IR22 | 3 | 77.22 | 103.74 | 55.45 |
| 2 | CONTROL | IR22 | 4 | 76.48 | 107.46 | 53.61 |
| 2 | CONTROL | IR22 | 5 | 75.73 | 111.18 | 51.77 |
| 2 | CONTROL | IR22 | 6 | 72.24 | 104.22 | 45.55 |
| 2 | CONTROL | IR22 | 7 | 83.33 | 117.57 | 50.94 |
| 2 | CONTROL | IR22 | 8 | 94.41 | 130.91 | 56.32 |
| 2 | CONTROL | IR22 | 9 | 94.59 | 125.72 | 51.73 |
| 2 | CONTROL | IR22 | 10 | 94.76 | 120.53 | 47.13 |
| 2 | WBPH | IR22 | 1 | 67.89 | 96.86 | 51.77 |
| 2 | WBPH | IR22 | 2 | 67.68 | 94.12 | 47.48 |
| 2 | WBPH | IR22 | 3 | 67.47 | 91.37 | 43.19 |
| 2 | WBPH | IR22 | 4 | 69.25 | 90.03 | 45.62 |
| 2 | WBPH | IR22 | 5 | 71.03 | 88.69 | 48.05 |
| 2 | WBPH | IR22 | 6 | 91.18 | 103.12 | 65.43 |
| 2 | WBPH | IR22 | 7 | 105.62 | 111.20 | 87.68 |
| 2 | WBPH | IR22 | 8 | 120.05 | 119.28 | 109.92 |
| 2 | WBPH | IR22 | 9 | 115.29 | 110.82 | 99.97 |
| 2 | WBPH | IR22 | 10 | 110.52 | 102.35 | 90.02 |
| 3 | BPH | IR22 | 1 | 71.21 | 99.03 | 67.83 |
| 3 | BPH | IR22 | 2 | 76.43 | 100.66 | 75.47 |
| 3 | BPH | IR22 | 3 | 81.64 | 102.29 | 83.11 |
| 3 | BPH | IR22 | 4 | 96.80 | 111.00 | 93.34 |
| 3 | BPH | IR22 | 5 | 111.96 | 119.70 | 103.57 |
| 3 | BPH | IR22 | 6 | 108.07 | 110.70 | 98.78 |
| 3 | BPH | IR22 | 7 | 115.85 | 119.34 | 105.64 |
| 3 | BPH | IR22 | 8 | 123.62 | 127.98 | 112.49 |
| 3 | BPH | IR22 | 9 | 117.25 | 115.48 | 109.22 |
| 3 | BPH | IR22 | 10 | 140.82 | 143.47 | 131.94 |
| 3 | CONTROL | IR22 | 1 | 69.24 | 102.23 | 53.42 |
| 3 | CONTROL | IR22 | 2 | 73.87 | 109.22 | 57.67 |
| 3 | CONTROL | IR22 | 3 | 78.49 | 116.21 | 61.91 |
| 3 | CONTROL | IR22 | 4 | 70.28 | 105.06 | 53.22 |
| 3 | CONTROL | IR22 | 5 | 62.07 | 93.91 | 44.52 |
| 3 | CONTROL | IR22 | 6 | 62.75 | 91.74 | 41.46 |
| 3 | CONTROL | IR22 | 7 | 74.26 | 103.30 | 44.68 |
| 3 | CONTROL | IR22 | 8 | 85.77 | 114.85 | 47.89 |
| 3 | CONTROL | IR22 | 9 | 75.41 | 107.26 | 46.03 |
| 3 | CONTROL | IR22 | 10 | 97.82 | 130.29 | 55.93 |
| 3 | WBPH | IR22 | 1 | 80.84 | 114.92 | 66.38 |
| 3 | WBPH | IR22 | 2 | 75.25 | 107.21 | 61.99 |
| 3 | WBPH | IR22 | 3 | 69.66 | 99.50 | 57.59 |
| 3 | WBPH | IR22 | 4 | 76.72 | 105.06 | 61.46 |
| 3 | WBPH | IR22 | 5 | 83.77 | 110.61 | 65.32 |
| 3 | WBPH | IR22 | 6 | 82.22 | 102.80 | 62.52 |
| 3 | WBPH | IR22 | 7 | 88.59 | 107.83 | 67.13 |
| 3 | WBPH | IR22 | 8 | 94.96 | 112.85 | 71.73 |
| 3 | WBPH | IR22 | 9 | 103.56 | 122.72 | 85.13 |
| 3 | WBPH | IR22 | 10 | 118.93 | 134.15 | 96.55 |
| 1 | BPH | IR24 | 1 | 79.48 | 115.51 | 67.62 |
| 1 | BPH | IR24 | 2 | 64.01 | 101.12 | 76.00 |
| 1 | BPH | IR24 | 3 | 84.43 | 120.62 | 73.74 |
| 1 | BPH | IR24 | 4 | 72.22 | 100.00 | 53.17 |
| 1 | BPH | IR24 | 5 | 86.19 | 111.23 | 63.29 |
| 1 | BPH | IR24 | 6 | 86.80 | 111.01 | 71.53 |
| 1 | BPH | IR24 | 7 | 83.83 | 106.15 | 65.65 |
| 1 | BPH | IR24 | 8 | 87.65 | 112.15 | 88.52 |
| 1 | BPH | IR24 | 9 | 93.37 | 105.38 | 76.31 |
| 1 | BPH | IR24 | 10 | 107.60 | 109.86 | 84.33 |
| 1 | CONTROL | IR24 | 1 | 64.97 | 97.41 | 56.34 |
| 1 | CONTROL | IR24 | 2 | 61.12 | 97.02 | 69.66 |
| 1 | CONTROL | IR24 | 3 | 64.47 | 96.70 | 60.90 |
| 1 | CONTROL | IR24 | 4 | 65.93 | 97.75 | 60.66 |
| 1 | CONTROL | IR24 | 5 | 81.76 | 115.29 | 66.29 |
| 1 | CONTROL | IR24 | 6 | 64.70 | 93.78 | 59.44 |
| 1 | CONTROL | IR24 | 7 | 66.23 | 95.74 | 58.51 |
| 1 | CONTROL | IR24 | 8 | 95.48 | 122.54 | 70.95 |
| 1 | CONTROL | IR24 | 9 | 108.32 | 134.53 | 86.70 |
| 1 | CONTROL | IR24 | 10 | 79.89 | 110.24 | 68.12 |
| 1 | WBPH | IR24 | 1 | 88.30 | 118.42 | 59.72 |
| 1 | WBPH | IR24 | 2 | 74.14 | 103.90 | 56.97 |
| 1 | WBPH | IR24 | 3 | 79.40 | 106.26 | 55.72 |
| 1 | WBPH | IR24 | 4 | 107.19 | 138.69 | 75.72 |
| 1 | WBPH | IR24 | 5 | 95.75 | 118.15 | 60.24 |
| 1 | WBPH | IR24 | 6 | 91.38 | 118.26 | 76.90 |
| 1 | WBPH | IR24 | 7 | 101.98 | 116.62 | 64.16 |
| 1 | WBPH | IR24 | 8 | 115.86 | 130.92 | 79.34 |
| 1 | WBPH | IR24 | 9 | 103.39 | 118.78 | 82.22 |
| 1 | WBPH | IR24 | 10 | 103.40 | 117.46 | 91.55 |
| 2 | BPH | IR24 | 1 | 64.18 | 89.15 | 44.92 |
| 2 | BPH | IR24 | 2 | 65.02 | 89.57 | 45.64 |
| 2 | BPH | IR24 | 3 | 65.86 | 89.99 | 46.35 |
| 2 | BPH | IR24 | 4 | 64.60 | 86.95 | 44.87 |
| 2 | BPH | IR24 | 5 | 63.34 | 83.91 | 43.39 |
| 2 | BPH | IR24 | 6 | 78.82 | 98.41 | 49.44 |
| 2 | BPH | IR24 | 7 | 86.49 | 102.34 | 61.21 |
| 2 | BPH | IR24 | 8 | 94.15 | 106.27 | 72.98 |
| 2 | BPH | IR24 | 9 | 99.03 | 109.75 | 82.79 |
| 2 | BPH | IR24 | 10 | 103.90 | 113.23 | 92.60 |
| 2 | CONTROL | IR24 | 1 | 63.82 | 90.03 | 45.28 |
| 2 | CONTROL | IR24 | 2 | 60.32 | 92.00 | 54.31 |
| 2 | CONTROL | IR24 | 3 | 56.82 | 93.97 | 63.33 |
| 2 | CONTROL | IR24 | 4 | 64.63 | 100.70 | 58.28 |
| 2 | CONTROL | IR24 | 5 | 72.43 | 107.42 | 53.22 |
| 2 | CONTROL | IR24 | 6 | 64.41 | 98.20 | 50.07 |
| 2 | CONTROL | IR24 | 7 | 74.70 | 109.83 | 52.77 |
| 2 | CONTROL | IR24 | 8 | 84.99 | 121.45 | 55.46 |
| 2 | CONTROL | IR24 | 9 | 81.87 | 116.79 | 52.24 |
| 2 | CONTROL | IR24 | 10 | 78.75 | 112.12 | 49.01 |
| 2 | WBPH | IR24 | 1 | 77.11 | 105.88 | 47.98 |
| 2 | WBPH | IR24 | 2 | 70.94 | 97.26 | 45.37 |
| 2 | WBPH | IR24 | 3 | 64.77 | 88.63 | 42.75 |
| 2 | WBPH | IR24 | 4 | 68.14 | 96.92 | 49.40 |
| 2 | WBPH | IR24 | 5 | 71.51 | 105.21 | 56.05 |
| 2 | WBPH | IR24 | 6 | 76.40 | 103.62 | 47.92 |
| 2 | WBPH | IR24 | 7 | 80.39 | 107.19 | 53.32 |
| 2 | WBPH | IR24 | 8 | 84.38 | 110.75 | 58.72 |
| 2 | WBPH | IR24 | 9 | 81.62 | 106.40 | 60.48 |
| 2 | WBPH | IR24 | 10 | 78.85 | 102.05 | 62.24 |
| 3 | BPH | IR24 | 1 | 71.91 | 105.49 | 59.90 |
| 3 | BPH | IR24 | 2 | 73.73 | 107.88 | 57.03 |
| 3 | BPH | IR24 | 3 | 75.54 | 110.27 | 54.16 |
| 3 | BPH | IR24 | 4 | 75.75 | 108.64 | 52.45 |
| 3 | BPH | IR24 | 5 | 75.96 | 107.01 | 50.74 |
| 3 | BPH | IR24 | 6 | 78.15 | 109.62 | 57.91 |
| 3 | BPH | IR24 | 7 | 81.71 | 108.95 | 52.88 |
| 3 | BPH | IR24 | 8 | 85.27 | 108.27 | 47.84 |
| 3 | BPH | IR24 | 9 | 92.15 | 121.67 | 65.32 |
| 3 | BPH | IR24 | 10 | 115.21 | 146.19 | 77.79 |
| 3 | CONTROL | IR24 | 1 | 69.94 | 99.66 | 52.90 |
| 3 | CONTROL | IR24 | 2 | 64.33 | 95.73 | 50.85 |
| 3 | CONTROL | IR24 | 3 | 58.71 | 91.79 | 48.80 |
| 3 | CONTROL | IR24 | 4 | 61.76 | 93.85 | 46.90 |
| 3 | CONTROL | IR24 | 5 | 64.80 | 95.90 | 44.99 |
| 3 | CONTROL | IR24 | 6 | 62.23 | 93.34 | 46.96 |
| 3 | CONTROL | IR24 | 7 | 69.78 | 101.42 | 47.57 |
| 3 | CONTROL | IR24 | 8 | 77.33 | 109.50 | 48.18 |
| 3 | CONTROL | IR24 | 9 | 88.90 | 116.84 | 47.52 |
| 3 | CONTROL | IR24 | 10 | 92.97 | 129.37 | 55.40 |
| 3 | WBPH | IR24 | 1 | 66.11 | 97.53 | 57.08 |
| 3 | WBPH | IR24 | 2 | 64.80 | 96.00 | 56.14 |
| 3 | WBPH | IR24 | 3 | 63.49 | 94.46 | 55.19 |
| 3 | WBPH | IR24 | 4 | 66.95 | 96.44 | 55.31 |
| 3 | WBPH | IR24 | 5 | 70.41 | 98.42 | 55.42 |
| 3 | WBPH | IR24 | 6 | 73.81 | 97.98 | 52.67 |
| 3 | WBPH | IR24 | 7 | 79.77 | 105.47 | 57.68 |
| 3 | WBPH | IR24 | 8 | 85.73 | 112.95 | 62.68 |
| 3 | WBPH | IR24 | 9 | 89.61 | 111.68 | 69.02 |
| 3 | WBPH | IR24 | 10 | 114.90 | 135.90 | 88.25 |
| 1 | BPH | IR40 | 1 | 105.05 | 143.24 | 81.39 |
| 1 | BPH | IR40 | 2 | 64.52 | 95.19 | 61.25 |
| 1 | BPH | IR40 | 3 | 87.62 | 118.67 | 55.24 |
| 1 | BPH | IR40 | 4 | 86.85 | 114.01 | 72.01 |
| 1 | BPH | IR40 | 5 | 96.88 | 119.41 | 86.25 |
| 1 | BPH | IR40 | 6 | 105.86 | 114.26 | 83.52 |
| 1 | BPH | IR40 | 7 | 92.23 | 102.44 | 88.68 |
| 1 | BPH | IR40 | 8 | 101.11 | 103.66 | 99.28 |
| 1 | BPH | IR40 | 9 | 97.94 | 93.27 | 90.42 |
| 1 | BPH | IR40 | 10 | 120.18 | 117.70 | 107.68 |
| 1 | CONTROL | IR40 | 1 | 83.12 | 120.15 | 60.91 |
| 1 | CONTROL | IR40 | 2 | 76.48 | 114.26 | 65.41 |
| 1 | CONTROL | IR40 | 3 | 86.31 | 121.74 | 62.02 |
| 1 | CONTROL | IR40 | 4 | 66.95 | 97.81 | 52.61 |
| 1 | CONTROL | IR40 | 5 | 77.92 | 110.37 | 57.31 |
| 1 | CONTROL | IR40 | 6 | 65.25 | 95.39 | 54.27 |
| 1 | CONTROL | IR40 | 7 | 73.24 | 104.39 | 55.38 |
| 1 | CONTROL | IR40 | 8 | 85.57 | 118.14 | 63.38 |
| 1 | CONTROL | IR40 | 9 | 113.96 | 148.74 | 86.40 |
| 1 | CONTROL | IR40 | 10 | 85.72 | 116.24 | 64.34 |
| 1 | WBPH | IR40 | 1 | 92.29 | 124.69 | 64.76 |
| 1 | WBPH | IR40 | 2 | 75.72 | 105.82 | 60.58 |
| 1 | WBPH | IR40 | 3 | 84.48 | 110.62 | 65.79 |
| 1 | WBPH | IR40 | 4 | 110.02 | 124.80 | 78.41 |
| 1 | WBPH | IR40 | 5 | 110.07 | 123.98 | 89.94 |
| 1 | WBPH | IR40 | 6 | 107.08 | 120.19 | 89.40 |
| 1 | WBPH | IR40 | 7 | 90.90 | 94.22 | 73.92 |
| 1 | WBPH | IR40 | 8 | 99.11 | 108.82 | 89.02 |
| 1 | WBPH | IR40 | 9 | 102.75 | 108.45 | 84.35 |
| 1 | WBPH | IR40 | 10 | 76.68 | 81.14 | 73.03 |
| 2 | BPH | IR40 | 1 | 68.41 | 88.92 | 61.25 |
| 2 | BPH | IR40 | 2 | 69.91 | 84.86 | 62.23 |
| 2 | BPH | IR40 | 3 | 71.41 | 80.80 | 63.21 |
| 2 | BPH | IR40 | 4 | 84.35 | 89.85 | 74.93 |
| 2 | BPH | IR40 | 5 | 97.29 | 98.89 | 86.65 |
| 2 | BPH | IR40 | 6 | 97.66 | 92.82 | 88.50 |
| 2 | BPH | IR40 | 7 | 110.24 | 105.16 | 98.51 |
| 2 | BPH | IR40 | 8 | 122.82 | 117.49 | 108.51 |
| 2 | BPH | IR40 | 9 | 126.32 | 119.39 | 111.42 |
| 2 | BPH | IR40 | 10 | 129.82 | 121.29 | 114.33 |
| 2 | CONTROL | IR40 | 1 | 70.26 | 104.64 | 49.51 |
| 2 | CONTROL | IR40 | 2 | 67.54 | 104.82 | 60.63 |
| 2 | CONTROL | IR40 | 3 | 64.82 | 104.99 | 71.74 |
| 2 | CONTROL | IR40 | 4 | 67.38 | 103.58 | 57.64 |
| 2 | CONTROL | IR40 | 5 | 69.94 | 102.17 | 43.53 |
| 2 | CONTROL | IR40 | 6 | 72.49 | 104.75 | 41.55 |
| 2 | CONTROL | IR40 | 7 | 87.19 | 122.67 | 49.60 |
| 2 | CONTROL | IR40 | 8 | 101.89 | 140.59 | 57.65 |
| 2 | CONTROL | IR40 | 9 | 92.51 | 128.25 | 50.40 |
| 2 | CONTROL | IR40 | 10 | 83.12 | 115.90 | 43.15 |
| 2 | WBPH | IR40 | 1 | 75.98 | 100.46 | 43.25 |
| 2 | WBPH | IR40 | 2 | 75.86 | 101.12 | 45.12 |
| 2 | WBPH | IR40 | 3 | 75.73 | 101.78 | 46.99 |
| 2 | WBPH | IR40 | 5 | 71.10 | 89.94 | 45.95 |
| 2 | WBPH | IR40 | 6 | 73.79 | 85.60 | 67.61 |
| 2 | WBPH | IR40 | 7 | 79.95 | 89.31 | 64.75 |
| 2 | WBPH | IR40 | 8 | 88.79 | 88.67 | 83.54 |
| 2 | WBPH | IR40 | 9 | 90.43 | 89.25 | 82.38 |
| 2 | WBPH | IR40 | 10 | 92.06 | 89.83 | 81.21 |
| 3 | BPH | IR40 | 1 | 101.63 | 130.57 | 59.67 |
| 3 | BPH | IR40 | 2 | 96.29 | 126.16 | 54.18 |
| 3 | BPH | IR40 | 3 | 90.95 | 121.75 | 48.69 |
| 3 | BPH | IR40 | 4 | 83.71 | 110.15 | 52.94 |
| 3 | BPH | IR40 | 5 | 76.46 | 98.54 | 57.19 |
| 3 | BPH | IR40 | 6 | 77.40 | 93.57 | 71.61 |
| 3 | BPH | IR40 | 7 | 93.02 | 100.66 | 83.06 |
| 3 | BPH | IR40 | 8 | 108.64 | 107.75 | 94.50 |
| 3 | BPH | IR40 | 9 | 119.09 | 108.16 | 101.14 |
| 3 | BPH | IR40 | 10 | 112.70 | 104.62 | 98.84 |
| 3 | CONTROL | IR40 | 1 | 79.72 | 112.72 | 54.05 |
| 3 | CONTROL | IR40 | 2 | 77.49 | 111.68 | 51.77 |
| 3 | CONTROL | IR40 | 3 | 75.26 | 110.63 | 49.48 |
| 3 | CONTROL | IR40 | 4 | 82.30 | 117.17 | 44.92 |
| 3 | CONTROL | IR40 | 5 | 89.34 | 123.70 | 40.35 |
| 3 | CONTROL | IR40 | 6 | 70.66 | 103.31 | 43.35 |
| 3 | CONTROL | IR40 | 7 | 75.34 | 107.85 | 45.17 |
| 3 | CONTROL | IR40 | 8 | 80.02 | 112.39 | 46.99 |
| 3 | CONTROL | IR40 | 9 | 86.69 | 121.53 | 48.81 |
| 3 | CONTROL | IR40 | 10 | 104.82 | 142.34 | 60.14 |
| 3 | WBPH | IR40 | 1 | 79.09 | 114.03 | 64.10 |
| 3 | WBPH | IR40 | 2 | 78.59 | 111.57 | 62.11 |
| 3 | WBPH | IR40 | 3 | 78.08 | 109.11 | 60.11 |
| 3 | WBPH | IR40 | 4 | 78.04 | 106.30 | 60.14 |
| 3 | WBPH | IR40 | 5 | 78.00 | 103.49 | 60.17 |
| 3 | WBPH | IR40 | 6 | 72.15 | 90.08 | 54.19 |
| 3 | WBPH | IR40 | 7 | 82.26 | 97.57 | 65.26 |
| 3 | WBPH | IR40 | 8 | 92.36 | 105.06 | 76.32 |
| 3 | WBPH | IR40 | 9 | 93.50 | 105.11 | 76.72 |
| 3 | WBPH | IR40 | 10 | 114.65 | 121.83 | 101.98 |
| 1 | BPH | IR56 | 1 | 95.13 | 130.21 | 70.49 |
| 1 | BPH | IR56 | 2 | 69.18 | 99.95 | 64.81 |
| 1 | BPH | IR56 | 3 | 68.99 | 101.40 | 54.30 |
| 1 | BPH | IR56 | 4 | 80.26 | 113.57 | 59.10 |
| 1 | BPH | IR56 | 5 | 74.26 | 100.36 | 55.36 |
| 1 | BPH | IR56 | 6 | 88.87 | 122.40 | 72.44 |
| 1 | BPH | IR56 | 7 | 94.39 | 122.93 | 58.27 |
| 1 | BPH | IR56 | 8 | 94.73 | 127.33 | 80.56 |
| 1 | BPH | IR56 | 9 | 108.98 | 131.73 | 72.85 |
| 1 | BPH | IR56 | 10 | 88.80 | 114.66 | 77.43 |
| 1 | CONTROL | IR56 | 1 | 80.57 | 117.59 | 58.70 |
| 1 | CONTROL | IR56 | 2 | 79.08 | 114.28 | 62.83 |
| 1 | CONTROL | IR56 | 3 | 84.76 | 112.94 | 56.43 |
| 1 | CONTROL | IR56 | 4 | 85.59 | 115.06 | 51.51 |
| 1 | CONTROL | IR56 | 5 | 81.68 | 111.27 | 45.35 |
| 1 | CONTROL | IR56 | 6 | 73.43 | 105.86 | 59.16 |
| 1 | CONTROL | IR56 | 7 | 77.55 | 110.70 | 59.43 |
| 1 | CONTROL | IR56 | 8 | 87.49 | 122.42 | 66.12 |
| 1 | CONTROL | IR56 | 9 | 93.59 | 127.84 | 65.96 |
| 1 | CONTROL | IR56 | 10 | 76.99 | 106.39 | 57.08 |
| 1 | WBPH | IR56 | 1 | 95.30 | 129.57 | 70.79 |
| 1 | WBPH | IR56 | 2 | 84.90 | 108.28 | 57.38 |
| 1 | WBPH | IR56 | 3 | 98.53 | 122.90 | 61.43 |
| 1 | WBPH | IR56 | 4 | 108.30 | 133.79 | 85.60 |
| 1 | WBPH | IR56 | 5 | 115.29 | 126.76 | 77.71 |
| 1 | WBPH | IR56 | 6 | 103.82 | 116.13 | 84.62 |
| 1 | WBPH | IR56 | 7 | 100.20 | 114.45 | 92.22 |
| 1 | WBPH | IR56 | 8 | 117.36 | 124.10 | 91.38 |
| 1 | WBPH | IR56 | 9 | 90.95 | 100.91 | 80.63 |
| 1 | WBPH | IR56 | 10 | 91.37 | 103.45 | 84.86 |
| 2 | BPH | IR56 | 1 | 71.35 | 100.90 | 58.44 |
| 2 | BPH | IR56 | 2 | 71.73 | 97.08 | 55.05 |
| 2 | BPH | IR56 | 3 | 72.10 | 93.26 | 51.66 |
| 2 | BPH | IR56 | 4 | 73.45 | 93.93 | 60.03 |
| 2 | BPH | IR56 | 5 | 74.80 | 94.59 | 68.40 |
| 2 | BPH | IR56 | 6 | 84.63 | 96.41 | 73.60 |
| 2 | BPH | IR56 | 7 | 95.42 | 106.76 | 85.15 |
| 2 | BPH | IR56 | 8 | 106.21 | 117.10 | 96.69 |
| 2 | BPH | IR56 | 9 | 91.70 | 100.15 | 84.24 |
| 2 | BPH | IR56 | 10 | 77.18 | 83.19 | 71.79 |
| 2 | CONTROL | IR56 | 1 | 75.97 | 100.99 | 47.18 |
| 2 | CONTROL | IR56 | 2 | 72.51 | 104.99 | 60.81 |
| 2 | CONTROL | IR56 | 3 | 69.04 | 108.98 | 74.43 |
| 2 | CONTROL | IR56 | 4 | 83.21 | 119.44 | 65.13 |
| 2 | CONTROL | IR56 | 5 | 97.38 | 129.90 | 55.82 |
| 2 | CONTROL | IR56 | 6 | 72.40 | 105.50 | 47.20 |
| 2 | CONTROL | IR56 | 7 | 78.41 | 112.57 | 51.23 |
| 2 | CONTROL | IR56 | 8 | 84.42 | 119.64 | 55.25 |
| 2 | CONTROL | IR56 | 9 | 88.42 | 120.67 | 52.77 |
| 2 | CONTROL | IR56 | 10 | 92.41 | 121.69 | 50.29 |
| 2 | WBPH | IR56 | 1 | 71.58 | 101.77 | 56.26 |
| 2 | WBPH | IR56 | 2 | 70.28 | 99.66 | 54.87 |
| 2 | WBPH | IR56 | 3 | 68.98 | 97.54 | 53.47 |
| 2 | WBPH | IR56 | 4 | 75.93 | 100.72 | 53.35 |
| 2 | WBPH | IR56 | 5 | 76.13 | 99.66 | 59.71 |
| 2 | WBPH | IR56 | 6 | 74.41 | 91.88 | 53.06 |
| 2 | WBPH | IR56 | 7 | 90.40 | 103.49 | 73.54 |
| 2 | WBPH | IR56 | 8 | 107.00 | 121.37 | 79.47 |
| 2 | WBPH | IR56 | 9 | 109.62 | 119.25 | 79.44 |
| 2 | WBPH | IR56 | 10 | 112.24 | 117.12 | 79.40 |
| 3 | BPH | IR56 | 1 | 89.80 | 125.88 | 63.66 |
| 3 | BPH | IR56 | 2 | 80.56 | 115.12 | 59.65 |
| 3 | BPH | IR56 | 3 | 71.32 | 104.35 | 55.63 |
| 3 | BPH | IR56 | 4 | 76.67 | 109.41 | 55.81 |
| 3 | BPH | IR56 | 5 | 82.01 | 114.47 | 55.98 |
| 3 | BPH | IR56 | 6 | 79.91 | 111.18 | 52.40 |
| 3 | BPH | IR56 | 7 | 85.53 | 113.91 | 53.90 |
| 3 | BPH | IR56 | 8 | 91.14 | 116.64 | 55.39 |
| 3 | BPH | IR56 | 9 | 82.19 | 110.02 | 62.12 |
| 3 | BPH | IR56 | 10 | 117.60 | 146.58 | 81.72 |
| 3 | CONTROL | IR56 | 1 | 85.63 | 120.63 | 50.77 |
| 3 | CONTROL | IR56 | 2 | 86.09 | 121.73 | 51.88 |
| 3 | CONTROL | IR56 | 3 | 86.54 | 122.83 | 52.99 |
| 3 | CONTROL | IR56 | 4 | 91.95 | 128.11 | 52.23 |
| 3 | CONTROL | IR56 | 5 | 97.35 | 133.39 | 51.47 |
| 3 | CONTROL | IR56 | 6 | 92.34 | 124.02 | 43.95 |
| 3 | CONTROL | IR56 | 7 | 99.35 | 129.87 | 48.70 |
| 3 | CONTROL | IR56 | 8 | 106.35 | 135.72 | 53.44 |
| 3 | CONTROL | IR56 | 9 | 85.76 | 117.57 | 48.96 |
| 3 | CONTROL | IR56 | 10 | 96.64 | 132.90 | 59.09 |
| 3 | WBPH | IR56 | 1 | 77.77 | 113.34 | 55.55 |
| 3 | WBPH | IR56 | 2 | 80.23 | 115.62 | 55.01 |
| 3 | WBPH | IR56 | 3 | 82.69 | 117.90 | 54.46 |
| 3 | WBPH | IR56 | 4 | 81.03 | 113.65 | 55.49 |
| 3 | WBPH | IR56 | 5 | 79.36 | 109.39 | 56.51 |
| 3 | WBPH | IR56 | 6 | 84.67 | 112.23 | 56.13 |
| 3 | WBPH | IR56 | 7 | 89.20 | 114.92 | 58.34 |
| 3 | WBPH | IR56 | 8 | 93.72 | 117.60 | 60.54 |
| 3 | WBPH | IR56 | 9 | 105.76 | 127.31 | 72.11 |
| 3 | WBPH | IR56 | 10 | 116.77 | 136.15 | 76.42 |
| 1 | BPH | IR60 | 1 | 87.29 | 124.30 | 67.09 |
| 1 | BPH | IR60 | 2 | 83.34 | 111.98 | 62.60 |
| 1 | BPH | IR60 | 3 | 78.73 | 107.31 | 58.96 |
| 1 | BPH | IR60 | 4 | 83.97 | 102.52 | 61.25 |
| 1 | BPH | IR60 | 5 | 82.28 | 104.03 | 66.55 |
| 1 | BPH | IR60 | 6 | 95.81 | 113.84 | 69.48 |
| 1 | BPH | IR60 | 7 | 97.29 | 103.01 | 74.80 |
| 1 | BPH | IR60 | 8 | 98.68 | 105.42 | 95.32 |
| 1 | BPH | IR60 | 9 | 80.20 | 86.91 | 77.30 |
| 1 | BPH | IR60 | 10 | 80.89 | 82.77 | 75.08 |
| 1 | CONTROL | IR60 | 1 | 76.81 | 113.49 | 50.34 |
| 1 | CONTROL | IR60 | 2 | 73.86 | 112.87 | 65.36 |
| 1 | CONTROL | IR60 | 3 | 92.09 | 126.85 | 51.43 |
| 1 | CONTROL | IR60 | 4 | 69.64 | 104.69 | 48.76 |
| 1 | CONTROL | IR60 | 5 | 63.89 | 95.32 | 46.21 |
| 1 | CONTROL | IR60 | 6 | 66.92 | 99.91 | 50.18 |
| 1 | CONTROL | IR60 | 7 | 69.86 | 101.91 | 47.94 |
| 1 | CONTROL | IR60 | 8 | 75.79 | 108.57 | 49.26 |
| 1 | CONTROL | IR60 | 9 | 86.33 | 120.81 | 55.09 |
| 1 | CONTROL | IR60 | 10 | 80.33 | 110.10 | 51.95 |
| 1 | WBPH | IR60 | 1 | 96.12 | 130.03 | 67.02 |
| 1 | WBPH | IR60 | 2 | 78.56 | 104.30 | 52.44 |
| 1 | WBPH | IR60 | 3 | 84.78 | 111.00 | 58.56 |
| 1 | WBPH | IR60 | 4 | 105.12 | 130.41 | 73.76 |
| 1 | WBPH | IR60 | 5 | 98.62 | 117.62 | 73.51 |
| 1 | WBPH | IR60 | 6 | 96.81 | 121.33 | 82.14 |
| 1 | WBPH | IR60 | 7 | 91.94 | 107.75 | 67.61 |
| 1 | WBPH | IR60 | 8 | 112.36 | 129.47 | 90.66 |
| 1 | WBPH | IR60 | 9 | 87.69 | 99.18 | 75.93 |
| 1 | WBPH | IR60 | 10 | 84.83 | 94.13 | 74.35 |
| 2 | BPH | IR60 | 1 | 56.97 | 83.15 | 48.47 |
| 2 | BPH | IR60 | 2 | 59.57 | 82.37 | 52.55 |
| 2 | BPH | IR60 | 3 | 62.17 | 81.59 | 56.63 |
| 2 | BPH | IR60 | 4 | 70.30 | 86.98 | 62.55 |
| 2 | BPH | IR60 | 5 | 78.42 | 92.36 | 68.47 |
| 2 | BPH | IR60 | 6 | 81.58 | 91.01 | 72.95 |
| 2 | BPH | IR60 | 7 | 87.89 | 94.91 | 81.18 |
| 2 | BPH | IR60 | 8 | 94.20 | 98.81 | 89.41 |
| 2 | BPH | IR60 | 9 | 95.19 | 98.42 | 88.64 |
| 2 | BPH | IR60 | 10 | 96.18 | 98.02 | 87.87 |
| 2 | CONTROL | IR60 | 1 | 69.88 | 102.03 | 40.06 |
| 2 | CONTROL | IR60 | 2 | 69.29 | 99.13 | 46.06 |
| 2 | CONTROL | IR60 | 3 | 68.70 | 96.22 | 52.05 |
| 2 | CONTROL | IR60 | 4 | 75.54 | 107.08 | 50.82 |
| 2 | CONTROL | IR60 | 5 | 82.37 | 117.94 | 49.59 |
| 2 | CONTROL | IR60 | 6 | 73.45 | 96.20 | 29.59 |
| 2 | CONTROL | IR60 | 7 | 88.31 | 112.74 | 39.37 |
| 2 | CONTROL | IR60 | 8 | 103.17 | 129.27 | 49.15 |
| 2 | CONTROL | IR60 | 9 | 94.25 | 120.44 | 46.86 |
| 2 | CONTROL | IR60 | 10 | 85.33 | 111.61 | 44.56 |
| 2 | WBPH | IR60 | 1 | 62.03 | 94.82 | 57.01 |
| 2 | WBPH | IR60 | 2 | 59.94 | 91.86 | 54.47 |
| 2 | WBPH | IR60 | 3 | 57.85 | 88.90 | 51.92 |
| 2 | WBPH | IR60 | 4 | 74.07 | 101.65 | 53.99 |
| 2 | WBPH | IR60 | 5 | 79.16 | 105.76 | 54.50 |
| 2 | WBPH | IR60 | 6 | 67.61 | 94.12 | 54.88 |
| 2 | WBPH | IR60 | 7 | 81.52 | 101.00 | 63.50 |
| 2 | WBPH | IR60 | 8 | 88.62 | 110.12 | 73.94 |
| 2 | WBPH | IR60 | 9 | 86.13 | 102.64 | 73.01 |
| 2 | WBPH | IR60 | 10 | 83.64 | 95.15 | 72.07 |
| 3 | BPH | IR60 | 1 | 72.24 | 105.92 | 63.00 |
| 3 | BPH | IR60 | 2 | 73.66 | 101.79 | 59.38 |
| 3 | BPH | IR60 | 3 | 75.07 | 97.66 | 55.75 |
| 3 | BPH | IR60 | 4 | 72.04 | 95.11 | 58.44 |
| 3 | BPH | IR60 | 5 | 69.00 | 92.56 | 61.13 |
| 3 | BPH | IR60 | 6 | 74.37 | 95.48 | 68.73 |
| 3 | BPH | IR60 | 7 | 83.62 | 100.89 | 71.60 |
| 3 | BPH | IR60 | 8 | 92.87 | 106.29 | 74.47 |
| 3 | BPH | IR60 | 9 | 96.57 | 109.18 | 96.80 |
| 3 | BPH | IR60 | 10 | 119.00 | 128.68 | 109.85 |
| 3 | CONTROL | IR60 | 1 | 83.15 | 119.29 | 56.62 |
| 3 | CONTROL | IR60 | 2 | 84.20 | 120.73 | 52.13 |
| 3 | CONTROL | IR60 | 3 | 85.24 | 122.16 | 47.63 |
| 3 | CONTROL | IR60 | 4 | 82.28 | 117.15 | 44.06 |
| 3 | CONTROL | IR60 | 5 | 79.31 | 112.14 | 40.49 |
| 3 | CONTROL | IR60 | 6 | 76.12 | 105.63 | 35.90 |
| 3 | CONTROL | IR60 | 7 | 75.22 | 105.82 | 39.27 |
| 3 | CONTROL | IR60 | 8 | 74.31 | 106.00 | 42.63 |
| 3 | CONTROL | IR60 | 9 | 86.90 | 117.22 | 38.60 |
| 3 | CONTROL | IR60 | 10 | 105.85 | 141.86 | 63.97 |
| 3 | WBPH | IR60 | 1 | 86.53 | 122.52 | 59.37 |
| 3 | WBPH | IR60 | 2 | 83.53 | 114.88 | 54.86 |
| 3 | WBPH | IR60 | 3 | 80.53 | 107.23 | 50.35 |
| 3 | WBPH | IR60 | 4 | 76.55 | 100.18 | 53.87 |
| 3 | WBPH | IR60 | 5 | 72.57 | 93.13 | 57.39 |
| 3 | WBPH | IR60 | 6 | 89.93 | 103.26 | 71.97 |
| 3 | WBPH | IR60 | 7 | 98.76 | 107.83 | 82.80 |
| 3 | WBPH | IR60 | 8 | 107.59 | 112.39 | 93.63 |
| 3 | WBPH | IR60 | 9 | 119.64 | 118.38 | 100.56 |
| 3 | WBPH | IR60 | 10 | 123.75 | 120.88 | 101.01 |
| 1 | BPH | IR62 | 1 | 86.99 | 116.40 | 52.04 |
| 1 | BPH | IR62 | 2 | 94.50 | 133.98 | 78.04 |
| 1 | BPH | IR62 | 3 | 101.20 | 136.82 | 71.53 |
| 1 | BPH | IR62 | 4 | 78.52 | 110.24 | 63.08 |
| 1 | BPH | IR62 | 5 | 104.08 | 126.86 | 57.67 |
| 1 | BPH | IR62 | 6 | 104.14 | 128.76 | 73.47 |
| 1 | BPH | IR62 | 7 | 120.98 | 142.52 | 80.99 |
| 1 | BPH | IR62 | 8 | 110.64 | 136.42 | 88.75 |
| 1 | BPH | IR62 | 9 | 131.60 | 149.13 | 91.40 |
| 1 | BPH | IR62 | 10 | 104.91 | 127.54 | 86.33 |
| 1 | CONTROL | IR62 | 1 | 76.25 | 112.38 | 58.32 |
| 1 | CONTROL | IR62 | 2 | 80.79 | 116.14 | 67.35 |
| 1 | CONTROL | IR62 | 3 | 89.96 | 118.43 | 56.90 |
| 1 | CONTROL | IR62 | 4 | 78.29 | 105.05 | 53.63 |
| 1 | CONTROL | IR62 | 5 | 76.67 | 107.87 | 57.78 |
| 1 | CONTROL | IR62 | 6 | 70.84 | 96.11 | 53.01 |
| 1 | CONTROL | IR62 | 7 | 72.54 | 96.65 | 50.91 |
| 1 | CONTROL | IR62 | 8 | 87.18 | 113.85 | 62.06 |
| 1 | CONTROL | IR62 | 9 | 94.01 | 124.31 | 67.82 |
| 1 | CONTROL | IR62 | 10 | 94.19 | 115.92 | 59.09 |
| 1 | WBPH | IR62 | 1 | 89.35 | 122.82 | 71.05 |
| 1 | WBPH | IR62 | 2 | 67.21 | 98.25 | 62.44 |
| 1 | WBPH | IR62 | 3 | 79.46 | 110.80 | 69.97 |
| 1 | WBPH | IR62 | 4 | 75.34 | 102.86 | 66.75 |
| 1 | WBPH | IR62 | 5 | 81.01 | 98.99 | 66.08 |
| 1 | WBPH | IR62 | 6 | 83.97 | 102.18 | 71.45 |
| 1 | WBPH | IR62 | 7 | 76.00 | 94.45 | 69.27 |
| 1 | WBPH | IR62 | 8 | 95.49 | 110.35 | 75.26 |
| 1 | WBPH | IR62 | 9 | 91.51 | 108.88 | 72.83 |
| 1 | WBPH | IR62 | 10 | 97.93 | 114.65 | 87.80 |
| 2 | BPH | IR62 | 1 | 71.70 | 105.56 | 56.15 |
| 2 | BPH | IR62 | 2 | 69.30 | 101.63 | 54.52 |
| 2 | BPH | IR62 | 3 | 66.90 | 97.70 | 52.89 |
| 2 | BPH | IR62 | 4 | 64.00 | 95.03 | 52.59 |
| 2 | BPH | IR62 | 5 | 61.09 | 92.36 | 52.29 |
| 2 | BPH | IR62 | 6 | 81.51 | 111.83 | 65.86 |
| 2 | BPH | IR62 | 7 | 81.93 | 108.85 | 72.44 |
| 2 | BPH | IR62 | 8 | 82.35 | 105.86 | 79.02 |
| 2 | BPH | IR62 | 9 | 82.40 | 105.28 | 80.08 |
| 2 | BPH | IR62 | 10 | 82.45 | 104.70 | 81.13 |
| 2 | CONTROL | IR62 | 1 | 72.17 | 100.61 | 51.91 |
| 2 | CONTROL | IR62 | 2 | 73.19 | 104.48 | 59.22 |
| 2 | CONTROL | IR62 | 3 | 74.21 | 108.34 | 66.52 |
| 2 | CONTROL | IR62 | 4 | 66.98 | 100.23 | 58.08 |
| 2 | CONTROL | IR62 | 5 | 59.74 | 92.12 | 49.64 |
| 2 | CONTROL | IR62 | 6 | 69.70 | 102.87 | 49.76 |
| 2 | CONTROL | IR62 | 7 | 81.23 | 111.75 | 51.98 |
| 2 | CONTROL | IR62 | 8 | 92.76 | 120.63 | 54.19 |
| 2 | CONTROL | IR62 | 9 | 76.02 | 105.83 | 52.33 |
| 2 | CONTROL | IR62 | 10 | 59.28 | 91.02 | 50.46 |
| 2 | WBPH | IR62 | 1 | 65.58 | 94.69 | 59.01 |
| 2 | WBPH | IR62 | 2 | 66.91 | 95.42 | 56.36 |
| 2 | WBPH | IR62 | 3 | 68.23 | 96.15 | 53.71 |
| 2 | WBPH | IR62 | 4 | 61.85 | 89.57 | 51.04 |
| 2 | WBPH | IR62 | 5 | 65.84 | 90.24 | 50.15 |
| 2 | WBPH | IR62 | 6 | 80.17 | 103.02 | 57.47 |
| 2 | WBPH | IR62 | 7 | 81.58 | 103.77 | 67.27 |
| 2 | WBPH | IR62 | 8 | 95.55 | 113.42 | 79.66 |
| 2 | WBPH | IR62 | 9 | 85.22 | 97.45 | 74.16 |
| 2 | WBPH | IR62 | 10 | 74.88 | 81.47 | 68.65 |
| 3 | BPH | IR62 | 1 | 100.44 | 132.81 | 63.41 |
| 3 | BPH | IR62 | 2 | 90.92 | 124.38 | 59.00 |
| 3 | BPH | IR62 | 3 | 81.40 | 115.95 | 54.59 |
| 3 | BPH | IR62 | 4 | 88.07 | 118.70 | 54.09 |
| 3 | BPH | IR62 | 5 | 94.74 | 121.44 | 53.59 |
| 3 | BPH | IR62 | 6 | 80.76 | 112.68 | 48.63 |
| 3 | BPH | IR62 | 7 | 83.32 | 112.83 | 50.82 |
| 3 | BPH | IR62 | 8 | 85.87 | 112.97 | 53.00 |
| 3 | BPH | IR62 | 9 | 91.08 | 114.84 | 56.67 |
| 3 | BPH | IR62 | 10 | 127.18 | 159.57 | 83.62 |
| 3 | CONTROL | IR62 | 1 | 74.57 | 109.01 | 54.75 |
| 3 | CONTROL | IR62 | 2 | 78.20 | 113.10 | 54.34 |
| 3 | CONTROL | IR62 | 3 | 81.83 | 117.19 | 53.93 |
| 3 | CONTROL | IR62 | 4 | 81.04 | 115.63 | 52.37 |
| 3 | CONTROL | IR62 | 5 | 80.24 | 114.07 | 50.81 |
| 3 | CONTROL | IR62 | 6 | 74.73 | 106.23 | 47.74 |
| 3 | CONTROL | IR62 | 7 | 84.41 | 118.08 | 50.88 |
| 3 | CONTROL | IR62 | 8 | 94.09 | 129.93 | 54.02 |
| 3 | CONTROL | IR62 | 9 | 90.19 | 125.02 | 55.07 |
| 3 | CONTROL | IR62 | 10 | 91.69 | 125.83 | 59.59 |
| 3 | WBPH | IR62 | 1 | 75.76 | 110.01 | 58.30 |
| 3 | WBPH | IR62 | 2 | 83.78 | 116.90 | 57.50 |
| 3 | WBPH | IR62 | 3 | 91.80 | 123.78 | 56.69 |
| 3 | WBPH | IR62 | 4 | 84.82 | 113.10 | 55.23 |
| 3 | WBPH | IR62 | 5 | 77.84 | 102.42 | 53.76 |
| 3 | WBPH | IR62 | 6 | 91.43 | 113.66 | 58.00 |
| 3 | WBPH | IR62 | 7 | 100.77 | 120.90 | 66.25 |
| 3 | WBPH | IR62 | 8 | 110.11 | 128.13 | 74.50 |
| 3 | WBPH | IR62 | 9 | 94.17 | 109.41 | 66.81 |
| 3 | WBPH | IR62 | 10 | 119.23 | 134.50 | 98.65 |
| 1 | BPH | IR64 | 1 | 88.10 | 126.88 | 65.10 |
| 1 | BPH | IR64 | 2 | 67.43 | 103.68 | 63.65 |
| 1 | BPH | IR64 | 3 | 72.30 | 106.30 | 63.95 |
| 1 | BPH | IR64 | 4 | 70.29 | 101.40 | 56.81 |
| 1 | BPH | IR64 | 5 | 84.10 | 108.96 | 58.16 |
| 1 | BPH | IR64 | 6 | 77.12 | 103.13 | 67.10 |
| 1 | BPH | IR64 | 7 | 98.52 | 118.63 | 61.77 |
| 1 | BPH | IR64 | 8 | 92.00 | 116.29 | 77.77 |
| 1 | BPH | IR64 | 9 | 114.18 | 133.06 | 82.68 |
| 1 | BPH | IR64 | 10 | 80.31 | 97.81 | 56.98 |
| 1 | CONTROL | IR64 | 1 | 87.89 | 117.06 | 50.37 |
| 1 | CONTROL | IR64 | 2 | 88.66 | 123.04 | 64.53 |
| 1 | CONTROL | IR64 | 3 | 86.50 | 120.49 | 53.58 |
| 1 | CONTROL | IR64 | 4 | 86.26 | 111.31 | 49.75 |
| 1 | CONTROL | IR64 | 5 | 106.69 | 139.11 | 62.86 |
| 1 | CONTROL | IR64 | 6 | 84.28 | 111.54 | 56.91 |
| 1 | CONTROL | IR64 | 7 | 99.01 | 120.72 | 54.29 |
| 1 | CONTROL | IR64 | 8 | 104.11 | 127.85 | 63.02 |
| 1 | CONTROL | IR64 | 9 | 112.72 | 138.58 | 69.35 |
| 1 | CONTROL | IR64 | 10 | 92.57 | 112.19 | 57.30 |
| 1 | WBPH | IR64 | 1 | 99.31 | 126.48 | 59.36 |
| 1 | WBPH | IR64 | 2 | 83.73 | 110.30 | 55.79 |
| 1 | WBPH | IR64 | 3 | 124.09 | 152.07 | 76.94 |
| 1 | WBPH | IR64 | 4 | 118.40 | 137.74 | 71.68 |
| 1 | WBPH | IR64 | 5 | 118.35 | 138.46 | 78.83 |
| 1 | WBPH | IR64 | 6 | 117.86 | 136.81 | 72.70 |
| 1 | WBPH | IR64 | 7 | 118.04 | 136.11 | 77.32 |
| 1 | WBPH | IR64 | 8 | 102.27 | 113.80 | 71.44 |
| 1 | WBPH | IR64 | 9 | 96.26 | 113.46 | 82.69 |
| 1 | WBPH | IR64 | 10 | 103.10 | 120.53 | 95.35 |
| 2 | BPH | IR64 | 1 | 59.08 | 89.18 | 51.28 |
| 2 | BPH | IR64 | 2 | 62.03 | 89.08 | 49.64 |
| 2 | BPH | IR64 | 3 | 64.97 | 88.98 | 48.00 |
| 2 | BPH | IR64 | 4 | 65.10 | 90.48 | 49.34 |
| 2 | BPH | IR64 | 5 | 65.22 | 91.97 | 50.68 |
| 2 | BPH | IR64 | 6 | 80.68 | 108.14 | 64.68 |
| 2 | BPH | IR64 | 7 | 77.88 | 102.14 | 64.44 |
| 2 | BPH | IR64 | 8 | 75.08 | 96.13 | 64.19 |
| 2 | BPH | IR64 | 9 | 83.85 | 103.34 | 72.91 |
| 2 | BPH | IR64 | 10 | 92.62 | 110.55 | 81.63 |
| 2 | CONTROL | IR64 | 1 | 79.58 | 107.46 | 44.02 |
| 2 | CONTROL | IR64 | 2 | 71.96 | 106.56 | 59.78 |
| 2 | CONTROL | IR64 | 3 | 64.34 | 105.65 | 75.53 |
| 2 | CONTROL | IR64 | 4 | 71.03 | 109.18 | 63.46 |
| 2 | CONTROL | IR64 | 5 | 77.72 | 112.70 | 51.38 |
| 2 | CONTROL | IR64 | 6 | 80.17 | 114.04 | 48.64 |
| 2 | CONTROL | IR64 | 7 | 82.46 | 116.49 | 51.45 |
| 2 | CONTROL | IR64 | 8 | 84.74 | 118.93 | 54.26 |
| 2 | CONTROL | IR64 | 9 | 94.00 | 127.01 | 51.73 |
| 2 | CONTROL | IR64 | 10 | 103.25 | 135.08 | 49.19 |
| 2 | WBPH | IR64 | 1 | 62.51 | 93.41 | 46.12 |
| 2 | WBPH | IR64 | 2 | 59.52 | 90.26 | 45.52 |
| 2 | WBPH | IR64 | 3 | 56.53 | 87.11 | 44.91 |
| 2 | WBPH | IR64 | 4 | 65.91 | 95.35 | 52.36 |
| 2 | WBPH | IR64 | 5 | 63.58 | 94.55 | 51.01 |
| 2 | WBPH | IR64 | 6 | 73.61 | 100.65 | 47.07 |
| 2 | WBPH | IR64 | 7 | 90.54 | 113.72 | 57.14 |
| 2 | WBPH | IR64 | 8 | 100.90 | 124.41 | 56.81 |
| 2 | WBPH | IR64 | 9 | 89.59 | 112.04 | 57.75 |
| 2 | WBPH | IR64 | 10 | 78.28 | 99.67 | 58.68 |
| 3 | BPH | IR64 | 1 | 70.55 | 99.97 | 48.87 |
| 3 | BPH | IR64 | 2 | 68.78 | 100.31 | 49.89 |
| 3 | BPH | IR64 | 3 | 67.00 | 100.65 | 50.90 |
| 3 | BPH | IR64 | 4 | 68.43 | 101.07 | 50.64 |
| 3 | BPH | IR64 | 5 | 69.85 | 101.48 | 50.38 |
| 3 | BPH | IR64 | 6 | 72.47 | 100.78 | 53.53 |
| 3 | BPH | IR64 | 7 | 72.59 | 101.07 | 56.64 |
| 3 | BPH | IR64 | 8 | 72.70 | 101.35 | 59.74 |
| 3 | BPH | IR64 | 9 | 75.88 | 95.98 | 69.96 |
| 3 | BPH | IR64 | 10 | 85.46 | 92.84 | 67.68 |
| 3 | CONTROL | IR64 | 1 | 83.75 | 118.06 | 57.59 |
| 3 | CONTROL | IR64 | 2 | 76.54 | 111.05 | 52.94 |
| 3 | CONTROL | IR64 | 3 | 69.32 | 104.03 | 48.29 |
| 3 | CONTROL | IR64 | 4 | 71.89 | 104.78 | 45.09 |
| 3 | CONTROL | IR64 | 5 | 74.45 | 105.53 | 41.88 |
| 3 | CONTROL | IR64 | 6 | 80.12 | 107.25 | 34.95 |
| 3 | CONTROL | IR64 | 7 | 85.64 | 115.88 | 41.05 |
| 3 | CONTROL | IR64 | 8 | 91.15 | 124.50 | 47.15 |
| 3 | CONTROL | IR64 | 9 | 113.87 | 139.48 | 43.36 |
| 3 | CONTROL | IR64 | 10 | 109.85 | 146.52 | 49.36 |
| 3 | WBPH | IR64 | 1 | 84.31 | 119.59 | 62.38 |
| 3 | WBPH | IR64 | 2 | 79.47 | 111.41 | 56.76 |
| 3 | WBPH | IR64 | 3 | 74.62 | 103.23 | 51.14 |
| 3 | WBPH | IR64 | 4 | 82.93 | 109.61 | 52.77 |
| 3 | WBPH | IR64 | 5 | 91.24 | 115.98 | 54.40 |
| 3 | WBPH | IR64 | 6 | 89.29 | 107.57 | 52.26 |
| 3 | WBPH | IR64 | 7 | 99.94 | 118.32 | 63.07 |
| 3 | WBPH | IR64 | 8 | 110.59 | 129.07 | 73.87 |
| 3 | WBPH | IR64 | 9 | 114.22 | 125.95 | 83.50 |
| 3 | WBPH | IR64 | 10 | 127.27 | 136.52 | 86.46 |
| 1 | BPH | IR65482-4-136-2-2 | 1 | 95.32 | 134.62 | 63.97 |
| 1 | BPH | IR65482-4-136-2-2 | 2 | 70.09 | 103.51 | 61.08 |
| 1 | BPH | IR65482-4-136-2-2 | 3 | 88.17 | 123.35 | 65.06 |
| 1 | BPH | IR65482-4-136-2-2 | 4 | 89.71 | 118.99 | 62.18 |
| 1 | BPH | IR65482-4-136-2-2 | 5 | 88.26 | 109.15 | 56.48 |
| 1 | BPH | IR65482-4-136-2-2 | 6 | 97.07 | 116.16 | 65.69 |
| 1 | BPH | IR65482-4-136-2-2 | 7 | 93.11 | 115.24 | 69.73 |
| 1 | BPH | IR65482-4-136-2-2 | 8 | 111.27 | 126.99 | 90.56 |
| 1 | BPH | IR65482-4-136-2-2 | 9 | 110.73 | 125.55 | 94.45 |
| 1 | BPH | IR65482-4-136-2-2 | 10 | 92.83 | 96.20 | 75.69 |
| 1 | CONTROL | IR65482-4-136-2-2 | 1 | 92.97 | 131.15 | 60.67 |
| 1 | CONTROL | IR65482-4-136-2-2 | 2 | 99.27 | 142.52 | 85.15 |
| 1 | CONTROL | IR65482-4-136-2-2 | 3 | 89.84 | 125.21 | 57.17 |
| 1 | CONTROL | IR65482-4-136-2-2 | 4 | 76.46 | 109.93 | 54.05 |
| 1 | CONTROL | IR65482-4-136-2-2 | 5 | 102.07 | 135.95 | 53.70 |
| 1 | CONTROL | IR65482-4-136-2-2 | 6 | 79.60 | 110.62 | 54.82 |
| 1 | CONTROL | IR65482-4-136-2-2 | 7 | 95.37 | 126.56 | 53.57 |
| 1 | CONTROL | IR65482-4-136-2-2 | 8 | 98.58 | 129.83 | 58.58 |
| 1 | CONTROL | IR65482-4-136-2-2 | 9 | 131.41 | 155.34 | 63.88 |
| 1 | CONTROL | IR65482-4-136-2-2 | 10 | 93.00 | 121.50 | 57.42 |
| 1 | WBPH | IR65482-4-136-2-2 | 1 | 99.75 | 131.59 | 61.48 |
| 1 | WBPH | IR65482-4-136-2-2 | 2 | 77.82 | 106.63 | 54.86 |
| 1 | WBPH | IR65482-4-136-2-2 | 3 | 97.67 | 130.95 | 64.59 |
| 1 | WBPH | IR65482-4-136-2-2 | 4 | 96.97 | 120.11 | 57.22 |
| 1 | WBPH | IR65482-4-136-2-2 | 5 | 106.30 | 130.45 | 64.50 |
| 1 | WBPH | IR65482-4-136-2-2 | 6 | 96.18 | 120.70 | 71.19 |
| 1 | WBPH | IR65482-4-136-2-2 | 7 | 101.99 | 117.57 | 62.97 |
| 1 | WBPH | IR65482-4-136-2-2 | 8 | 117.41 | 133.55 | 79.75 |
| 1 | WBPH | IR65482-4-136-2-2 | 9 | 116.15 | 130.29 | 74.22 |
| 1 | WBPH | IR65482-4-136-2-2 | 10 | 113.94 | 128.33 | 78.36 |
| 2 | BPH | IR65482-4-136-2-2 | 1 | 86.73 | 115.82 | 49.12 |
| 2 | BPH | IR65482-4-136-2-2 | 2 | 78.19 | 109.09 | 48.98 |
| 2 | BPH | IR65482-4-136-2-2 | 3 | 69.64 | 102.36 | 48.84 |
| 2 | BPH | IR65482-4-136-2-2 | 4 | 78.88 | 111.79 | 51.85 |
| 2 | BPH | IR65482-4-136-2-2 | 5 | 88.11 | 121.22 | 54.85 |
| 2 | BPH | IR65482-4-136-2-2 | 6 | 101.12 | 125.55 | 54.52 |
| 2 | BPH | IR65482-4-136-2-2 | 7 | 104.41 | 133.32 | 65.66 |
| 2 | BPH | IR65482-4-136-2-2 | 8 | 107.70 | 141.09 | 76.80 |
| 2 | BPH | IR65482-4-136-2-2 | 9 | 101.34 | 133.03 | 77.29 |
| 2 | BPH | IR65482-4-136-2-2 | 10 | 94.97 | 124.96 | 77.78 |
| 2 | CONTROL | IR65482-4-136-2-2 | 1 | 61.08 | 93.30 | 45.59 |
| 2 | CONTROL | IR65482-4-136-2-2 | 2 | 56.78 | 90.57 | 50.11 |
| 2 | CONTROL | IR65482-4-136-2-2 | 3 | 52.48 | 87.83 | 54.63 |
| 2 | CONTROL | IR65482-4-136-2-2 | 4 | 62.60 | 96.59 | 49.03 |
| 2 | CONTROL | IR65482-4-136-2-2 | 5 | 72.72 | 105.35 | 43.43 |
| 2 | CONTROL | IR65482-4-136-2-2 | 6 | 76.10 | 108.93 | 46.12 |
| 2 | CONTROL | IR65482-4-136-2-2 | 7 | 77.74 | 110.52 | 46.79 |
| 2 | CONTROL | IR65482-4-136-2-2 | 8 | 79.38 | 112.11 | 47.46 |
| 2 | CONTROL | IR65482-4-136-2-2 | 9 | 92.35 | 121.49 | 47.98 |
| 2 | CONTROL | IR65482-4-136-2-2 | 10 | 105.32 | 130.87 | 48.49 |
| 2 | WBPH | IR65482-4-136-2-2 | 1 | 87.25 | 110.75 | 48.39 |
| 2 | WBPH | IR65482-4-136-2-2 | 2 | 80.59 | 105.99 | 49.31 |
| 2 | WBPH | IR65482-4-136-2-2 | 3 | 73.93 | 101.22 | 50.22 |
| 2 | WBPH | IR65482-4-136-2-2 | 4 | 72.02 | 100.74 | 53.81 |
| 2 | WBPH | IR65482-4-136-2-2 | 5 | 87.51 | 114.37 | 62.70 |
| 2 | WBPH | IR65482-4-136-2-2 | 6 | 84.35 | 100.83 | 68.37 |
| 2 | WBPH | IR65482-4-136-2-2 | 7 | 90.27 | 106.09 | 72.57 |
| 2 | WBPH | IR65482-4-136-2-2 | 8 | 106.92 | 111.52 | 98.07 |
| 2 | WBPH | IR65482-4-136-2-2 | 9 | 109.58 | 111.18 | 99.78 |
| 2 | WBPH | IR65482-4-136-2-2 | 10 | 112.23 | 110.83 | 101.49 |
| 3 | BPH | IR65482-4-136-2-2 | 1 | 89.19 | 124.04 | 59.60 |
| 3 | BPH | IR65482-4-136-2-2 | 2 | 89.34 | 124.46 | 55.50 |
| 3 | BPH | IR65482-4-136-2-2 | 3 | 89.48 | 124.88 | 51.39 |
| 3 | BPH | IR65482-4-136-2-2 | 4 | 87.75 | 119.92 | 45.87 |
| 3 | BPH | IR65482-4-136-2-2 | 5 | 86.01 | 114.95 | 40.34 |
| 3 | BPH | IR65482-4-136-2-2 | 6 | 89.04 | 117.71 | 46.04 |
| 3 | BPH | IR65482-4-136-2-2 | 7 | 96.32 | 125.08 | 45.13 |
| 3 | BPH | IR65482-4-136-2-2 | 8 | 103.59 | 132.45 | 44.21 |
| 3 | BPH | IR65482-4-136-2-2 | 9 | 100.30 | 129.91 | 61.60 |
| 3 | BPH | IR65482-4-136-2-2 | 10 | 123.82 | 150.57 | 69.49 |
| 3 | CONTROL | IR65482-4-136-2-2 | 1 | 85.68 | 118.94 | 55.73 |
| 3 | CONTROL | IR65482-4-136-2-2 | 2 | 83.57 | 117.73 | 54.03 |
| 3 | CONTROL | IR65482-4-136-2-2 | 3 | 81.45 | 116.52 | 52.33 |
| 3 | CONTROL | IR65482-4-136-2-2 | 4 | 80.48 | 114.08 | 49.49 |
| 3 | CONTROL | IR65482-4-136-2-2 | 5 | 79.51 | 111.64 | 46.65 |
| 3 | CONTROL | IR65482-4-136-2-2 | 6 | 85.50 | 117.82 | 47.92 |
| 3 | CONTROL | IR65482-4-136-2-2 | 7 | 90.62 | 121.15 | 49.14 |
| 3 | CONTROL | IR65482-4-136-2-2 | 8 | 95.73 | 124.48 | 50.35 |
| 3 | CONTROL | IR65482-4-136-2-2 | 9 | 86.73 | 115.51 | 50.48 |
| 3 | CONTROL | IR65482-4-136-2-2 | 10 | 105.03 | 140.96 | 57.58 |
| 3 | WBPH | IR65482-4-136-2-2 | 1 | 82.56 | 115.02 | 61.77 |
| 3 | WBPH | IR65482-4-136-2-2 | 2 | 82.99 | 114.93 | 58.90 |
| 3 | WBPH | IR65482-4-136-2-2 | 3 | 83.42 | 114.83 | 56.03 |
| 3 | WBPH | IR65482-4-136-2-2 | 4 | 78.99 | 107.34 | 54.39 |
| 3 | WBPH | IR65482-4-136-2-2 | 5 | 74.56 | 99.84 | 52.74 |
| 3 | WBPH | IR65482-4-136-2-2 | 6 | 85.80 | 107.50 | 56.28 |
| 3 | WBPH | IR65482-4-136-2-2 | 7 | 92.46 | 111.74 | 66.76 |
| 3 | WBPH | IR65482-4-136-2-2 | 8 | 99.12 | 115.97 | 77.23 |
| 3 | WBPH | IR65482-4-136-2-2 | 9 | 104.43 | 110.17 | 83.43 |
| 3 | WBPH | IR65482-4-136-2-2 | 10 | 140.77 | 145.01 | 110.29 |
| 1 | BPH | IR65482-7-216-1-2-B | 1 | 85.75 | 120.19 | 64.53 |
| 1 | BPH | IR65482-7-216-1-2-B | 2 | 99.15 | 134.44 | 81.66 |
| 1 | BPH | IR65482-7-216-1-2-B | 3 | 87.88 | 120.20 | 65.80 |
| 1 | BPH | IR65482-7-216-1-2-B | 4 | 87.27 | 113.62 | 60.93 |
| 1 | BPH | IR65482-7-216-1-2-B | 5 | 99.94 | 126.24 | 61.57 |
| 1 | BPH | IR65482-7-216-1-2-B | 6 | 94.18 | 121.51 | 73.90 |
| 1 | BPH | IR65482-7-216-1-2-B | 7 | 92.97 | 105.62 | 61.31 |
| 1 | BPH | IR65482-7-216-1-2-B | 8 | 113.42 | 123.18 | 84.08 |
| 1 | BPH | IR65482-7-216-1-2-B | 9 | 98.43 | 106.99 | 85.37 |
| 1 | BPH | IR65482-7-216-1-2-B | 10 | 106.59 | 110.53 | 93.17 |
| 1 | CONTROL | IR65482-7-216-1-2-B | 1 | 72.30 | 105.23 | 52.04 |
| 1 | CONTROL | IR65482-7-216-1-2-B | 2 | 76.98 | 111.36 | 64.33 |
| 1 | CONTROL | IR65482-7-216-1-2-B | 3 | 94.22 | 124.97 | 48.82 |
| 1 | CONTROL | IR65482-7-216-1-2-B | 4 | 76.26 | 104.83 | 41.62 |
| 1 | CONTROL | IR65482-7-216-1-2-B | 5 | 82.92 | 115.47 | 52.95 |
| 1 | CONTROL | IR65482-7-216-1-2-B | 6 | 82.70 | 114.68 | 54.59 |
| 1 | CONTROL | IR65482-7-216-1-2-B | 7 | 85.11 | 118.74 | 57.43 |
| 1 | CONTROL | IR65482-7-216-1-2-B | 8 | 94.57 | 127.83 | 64.14 |
| 1 | CONTROL | IR65482-7-216-1-2-B | 9 | 99.97 | 126.19 | 57.31 |
| 1 | CONTROL | IR65482-7-216-1-2-B | 10 | 103.11 | 123.79 | 50.35 |
| 1 | WBPH | IR65482-7-216-1-2-B | 1 | 96.91 | 124.82 | 60.15 |
| 1 | WBPH | IR65482-7-216-1-2-B | 2 | 101.95 | 123.25 | 60.41 |
| 1 | WBPH | IR65482-7-216-1-2-B | 3 | 114.29 | 136.70 | 65.65 |
| 1 | WBPH | IR65482-7-216-1-2-B | 4 | 118.87 | 147.67 | 75.06 |
| 1 | WBPH | IR65482-7-216-1-2-B | 5 | 121.74 | 140.91 | 82.01 |
| 1 | WBPH | IR65482-7-216-1-2-B | 6 | 128.36 | 134.75 | 79.74 |
| 1 | WBPH | IR65482-7-216-1-2-B | 7 | 134.18 | 140.79 | 82.26 |
| 1 | WBPH | IR65482-7-216-1-2-B | 8 | 140.67 | 148.37 | 99.75 |
| 1 | WBPH | IR65482-7-216-1-2-B | 9 | 124.87 | 132.43 | 104.93 |
| 1 | WBPH | IR65482-7-216-1-2-B | 10 | 108.67 | 119.03 | 97.77 |
| 2 | BPH | IR65482-7-216-1-2-B | 1 | 78.94 | 103.48 | 49.72 |
| 2 | BPH | IR65482-7-216-1-2-B | 2 | 74.29 | 101.87 | 53.79 |
| 2 | BPH | IR65482-7-216-1-2-B | 3 | 69.63 | 100.26 | 57.86 |
| 2 | BPH | IR65482-7-216-1-2-B | 4 | 74.54 | 104.46 | 57.69 |
| 2 | BPH | IR65482-7-216-1-2-B | 5 | 79.44 | 108.65 | 57.52 |
| 2 | BPH | IR65482-7-216-1-2-B | 6 | 90.48 | 112.41 | 74.31 |
| 2 | BPH | IR65482-7-216-1-2-B | 7 | 102.08 | 117.35 | 90.95 |
| 2 | BPH | IR65482-7-216-1-2-B | 8 | 113.68 | 122.28 | 107.59 |
| 2 | BPH | IR65482-7-216-1-2-B | 9 | 106.77 | 111.45 | 100.98 |
| 2 | BPH | IR65482-7-216-1-2-B | 10 | 99.85 | 100.61 | 94.37 |
| 2 | CONTROL | IR65482-7-216-1-2-B | 1 | 80.83 | 114.07 | 57.03 |
| 2 | CONTROL | IR65482-7-216-1-2-B | 2 | 88.30 | 123.51 | 64.81 |
| 2 | CONTROL | IR65482-7-216-1-2-B | 3 | 95.77 | 132.94 | 72.59 |
| 2 | CONTROL | IR65482-7-216-1-2-B | 4 | 111.62 | 142.95 | 70.38 |
| 2 | CONTROL | IR65482-7-216-1-2-B | 5 | 127.47 | 152.95 | 68.17 |
| 2 | CONTROL | IR65482-7-216-1-2-B | 6 | 69.06 | 101.13 | 53.14 |
| 2 | CONTROL | IR65482-7-216-1-2-B | 7 | 73.12 | 105.02 | 52.38 |
| 2 | CONTROL | IR65482-7-216-1-2-B | 8 | 77.17 | 108.90 | 51.61 |
| 2 | CONTROL | IR65482-7-216-1-2-B | 9 | 89.66 | 118.70 | 54.07 |
| 2 | CONTROL | IR65482-7-216-1-2-B | 10 | 102.14 | 128.50 | 56.52 |
| 2 | WBPH | IR65482-7-216-1-2-B | 1 | 82.17 | 106.43 | 47.47 |
| 2 | WBPH | IR65482-7-216-1-2-B | 2 | 84.17 | 109.56 | 46.18 |
| 2 | WBPH | IR65482-7-216-1-2-B | 3 | 86.16 | 112.68 | 44.88 |
| 2 | WBPH | IR65482-7-216-1-2-B | 4 | 79.96 | 105.59 | 50.17 |
| 2 | WBPH | IR65482-7-216-1-2-B | 5 | 85.99 | 109.95 | 50.12 |
| 2 | WBPH | IR65482-7-216-1-2-B | 6 | 85.94 | 99.89 | 52.31 |
| 2 | WBPH | IR65482-7-216-1-2-B | 7 | 82.47 | 98.64 | 68.07 |
| 2 | WBPH | IR65482-7-216-1-2-B | 8 | 80.59 | 96.45 | 67.76 |
| 2 | WBPH | IR65482-7-216-1-2-B | 9 | 85.39 | 98.66 | 74.30 |
| 2 | WBPH | IR65482-7-216-1-2-B | 10 | 90.19 | 100.87 | 80.83 |
| 3 | BPH | IR65482-7-216-1-2-B | 1 | 79.82 | 105.76 | 54.34 |
| 3 | BPH | IR65482-7-216-1-2-B | 2 | 85.83 | 113.66 | 55.83 |
| 3 | BPH | IR65482-7-216-1-2-B | 3 | 91.84 | 121.55 | 57.31 |
| 3 | BPH | IR65482-7-216-1-2-B | 4 | 83.92 | 111.41 | 61.73 |
| 3 | BPH | IR65482-7-216-1-2-B | 5 | 76.00 | 101.26 | 66.15 |
| 3 | BPH | IR65482-7-216-1-2-B | 6 | 86.50 | 105.21 | 74.23 |
| 3 | BPH | IR65482-7-216-1-2-B | 7 | 99.06 | 111.04 | 87.43 |
| 3 | BPH | IR65482-7-216-1-2-B | 8 | 111.62 | 116.87 | 100.62 |
| 3 | BPH | IR65482-7-216-1-2-B | 9 | 144.44 | 133.68 | 122.83 |
| 3 | BPH | IR65482-7-216-1-2-B | 10 | 137.34 | 128.12 | 117.50 |
| 3 | CONTROL | IR65482-7-216-1-2-B | 1 | 90.62 | 121.08 | 52.16 |
| 3 | CONTROL | IR65482-7-216-1-2-B | 2 | 92.31 | 120.66 | 50.28 |
| 3 | CONTROL | IR65482-7-216-1-2-B | 3 | 94.00 | 120.23 | 48.40 |
| 3 | CONTROL | IR65482-7-216-1-2-B | 4 | 93.63 | 122.81 | 49.63 |
| 3 | CONTROL | IR65482-7-216-1-2-B | 5 | 93.26 | 125.38 | 50.85 |
| 3 | CONTROL | IR65482-7-216-1-2-B | 6 | 86.78 | 118.08 | 48.20 |
| 3 | CONTROL | IR65482-7-216-1-2-B | 7 | 101.57 | 131.06 | 51.70 |
| 3 | CONTROL | IR65482-7-216-1-2-B | 8 | 116.35 | 144.03 | 55.19 |
| 3 | CONTROL | IR65482-7-216-1-2-B | 9 | 95.56 | 127.91 | 49.02 |
| 3 | CONTROL | IR65482-7-216-1-2-B | 10 | 116.36 | 152.79 | 69.60 |
| 3 | WBPH | IR65482-7-216-1-2-B | 1 | 90.09 | 116.98 | 56.47 |
| 3 | WBPH | IR65482-7-216-1-2-B | 2 | 87.74 | 117.49 | 58.83 |
| 3 | WBPH | IR65482-7-216-1-2-B | 3 | 85.39 | 118.00 | 61.18 |
| 3 | WBPH | IR65482-7-216-1-2-B | 4 | 84.40 | 115.09 | 60.77 |
| 3 | WBPH | IR65482-7-216-1-2-B | 5 | 83.40 | 112.17 | 60.35 |
| 3 | WBPH | IR65482-7-216-1-2-B | 6 | 79.43 | 105.40 | 57.68 |
| 3 | WBPH | IR65482-7-216-1-2-B | 7 | 87.77 | 113.72 | 59.95 |
| 3 | WBPH | IR65482-7-216-1-2-B | 8 | 96.11 | 122.03 | 62.22 |
| 3 | WBPH | IR65482-7-216-1-2-B | 9 | 98.24 | 120.24 | 69.95 |
| 3 | WBPH | IR65482-7-216-1-2-B | 10 | 119.14 | 136.56 | 74.87 |
| 1 | BPH | IR66 | 1 | 82.57 | 116.65 | 62.41 |
| 1 | BPH | IR66 | 2 | 80.86 | 115.26 | 68.42 |
| 1 | BPH | IR66 | 3 | 87.82 | 119.69 | 64.01 |
| 1 | BPH | IR66 | 4 | 80.17 | 111.29 | 63.61 |
| 1 | BPH | IR66 | 5 | 92.79 | 112.24 | 50.06 |
| 1 | BPH | IR66 | 6 | 97.40 | 116.40 | 63.32 |
| 1 | BPH | IR66 | 7 | 112.35 | 139.72 | 70.04 |
| 1 | BPH | IR66 | 8 | 114.34 | 129.52 | 82.64 |
| 1 | BPH | IR66 | 9 | 122.68 | 141.96 | 90.15 |
| 1 | BPH | IR66 | 10 | 104.71 | 115.37 | 76.10 |
| 1 | CONTROL | IR66 | 1 | 94.42 | 132.17 | 68.15 |
| 1 | CONTROL | IR66 | 2 | 85.95 | 118.98 | 70.84 |
| 1 | CONTROL | IR66 | 3 | 99.24 | 126.35 | 65.66 |
| 1 | CONTROL | IR66 | 4 | 79.96 | 112.75 | 62.72 |
| 1 | CONTROL | IR66 | 5 | 83.35 | 113.70 | 59.27 |
| 1 | CONTROL | IR66 | 6 | 80.90 | 106.71 | 59.06 |
| 1 | CONTROL | IR66 | 7 | 86.15 | 108.63 | 48.47 |
| 1 | CONTROL | IR66 | 8 | 94.81 | 118.78 | 59.18 |
| 1 | CONTROL | IR66 | 9 | 88.54 | 110.59 | 58.73 |
| 1 | CONTROL | IR66 | 10 | 89.78 | 120.97 | 67.57 |
| 1 | WBPH | IR66 | 1 | 112.58 | 149.78 | 80.57 |
| 1 | WBPH | IR66 | 2 | 88.98 | 113.99 | 56.73 |
| 1 | WBPH | IR66 | 3 | 111.80 | 143.87 | 71.46 |
| 1 | WBPH | IR66 | 4 | 108.03 | 134.15 | 64.18 |
| 1 | WBPH | IR66 | 5 | 101.87 | 124.19 | 57.77 |
| 1 | WBPH | IR66 | 6 | 94.99 | 121.75 | 59.81 |
| 1 | WBPH | IR66 | 7 | 96.23 | 117.31 | 50.82 |
| 1 | WBPH | IR66 | 8 | 101.50 | 123.77 | 65.81 |
| 1 | WBPH | IR66 | 9 | 96.48 | 114.99 | 63.91 |
| 1 | WBPH | IR66 | 10 | 103.70 | 120.98 | 83.14 |
| 2 | BPH | IR66 | 1 | 65.67 | 95.65 | 57.65 |
| 2 | BPH | IR66 | 2 | 65.46 | 95.41 | 56.79 |
| 2 | BPH | IR66 | 3 | 65.25 | 95.17 | 55.93 |
| 2 | BPH | IR66 | 4 | 63.15 | 89.66 | 57.82 |
| 2 | BPH | IR66 | 5 | 61.04 | 84.14 | 59.70 |
| 2 | BPH | IR66 | 6 | 85.85 | 95.48 | 76.84 |
| 2 | BPH | IR66 | 7 | 96.95 | 104.68 | 89.69 |
| 2 | BPH | IR66 | 8 | 108.05 | 113.87 | 102.53 |
| 2 | BPH | IR66 | 9 | 108.16 | 111.68 | 103.71 |
| 2 | BPH | IR66 | 10 | 108.27 | 109.49 | 104.88 |
| 2 | CONTROL | IR66 | 1 | 73.65 | 109.24 | 56.93 |
| 2 | CONTROL | IR66 | 2 | 67.82 | 106.95 | 68.30 |
| 2 | CONTROL | IR66 | 3 | 61.99 | 104.65 | 79.66 |
| 2 | CONTROL | IR66 | 4 | 68.30 | 107.35 | 66.13 |
| 2 | CONTROL | IR66 | 5 | 74.60 | 110.05 | 52.60 |
| 2 | CONTROL | IR66 | 6 | 80.99 | 116.70 | 57.23 |
| 2 | CONTROL | IR66 | 7 | 85.21 | 120.77 | 56.78 |
| 2 | CONTROL | IR66 | 8 | 89.42 | 124.83 | 56.33 |
| 2 | CONTROL | IR66 | 9 | 86.96 | 123.38 | 60.24 |
| 2 | CONTROL | IR66 | 10 | 84.50 | 121.93 | 64.15 |
| 2 | WBPH | IR66 | 1 | 72.30 | 106.55 | 51.80 |
| 2 | WBPH | IR66 | 2 | 74.93 | 108.74 | 54.92 |
| 2 | WBPH | IR66 | 3 | 77.56 | 110.93 | 58.03 |
| 2 | WBPH | IR66 | 4 | 79.25 | 106.71 | 49.73 |
| 2 | WBPH | IR66 | 5 | 72.33 | 100.73 | 54.58 |
| 2 | WBPH | IR66 | 6 | 58.01 | 83.38 | 47.29 |
| 2 | WBPH | IR66 | 7 | 87.67 | 102.60 | 70.17 |
| 2 | WBPH | IR66 | 8 | 89.39 | 105.30 | 88.02 |
| 2 | WBPH | IR66 | 9 | 90.00 | 103.53 | 88.52 |
| 2 | WBPH | IR66 | 10 | 90.60 | 101.76 | 89.01 |
| 3 | BPH | IR66 | 1 | 91.91 | 124.81 | 58.79 |
| 3 | BPH | IR66 | 2 | 89.57 | 122.07 | 62.74 |
| 3 | BPH | IR66 | 3 | 87.22 | 119.32 | 66.68 |
| 3 | BPH | IR66 | 4 | 89.40 | 115.12 | 74.67 |
| 3 | BPH | IR66 | 5 | 91.57 | 110.91 | 82.65 |
| 3 | BPH | IR66 | 6 | 98.00 | 102.39 | 84.95 |
| 3 | BPH | IR66 | 7 | 111.25 | 115.27 | 100.24 |
| 3 | BPH | IR66 | 8 | 124.49 | 128.15 | 115.53 |
| 3 | BPH | IR66 | 9 | 125.17 | 126.75 | 112.94 |
| 3 | BPH | IR66 | 10 | 140.16 | 140.26 | 132.28 |
| 3 | CONTROL | IR66 | 1 | 78.87 | 113.01 | 65.72 |
| 3 | CONTROL | IR66 | 2 | 93.92 | 130.90 | 72.56 |
| 3 | CONTROL | IR66 | 3 | 108.97 | 148.79 | 79.39 |
| 3 | CONTROL | IR66 | 4 | 100.78 | 138.33 | 68.76 |
| 3 | CONTROL | IR66 | 5 | 92.59 | 127.86 | 58.13 |
| 3 | CONTROL | IR66 | 6 | 80.89 | 115.49 | 59.21 |
| 3 | CONTROL | IR66 | 7 | 90.51 | 126.25 | 62.18 |
| 3 | CONTROL | IR66 | 8 | 100.13 | 137.01 | 65.15 |
| 3 | CONTROL | IR66 | 9 | 77.73 | 110.28 | 54.27 |
| 3 | CONTROL | IR66 | 10 | 92.80 | 128.88 | 71.14 |
| 3 | WBPH | IR66 | 1 | 79.66 | 113.21 | 58.00 |
| 3 | WBPH | IR66 | 2 | 81.68 | 112.93 | 57.00 |
| 3 | WBPH | IR66 | 3 | 83.70 | 112.65 | 55.99 |
| 3 | WBPH | IR66 | 4 | 80.10 | 110.11 | 55.24 |
| 3 | WBPH | IR66 | 5 | 76.49 | 107.56 | 54.49 |
| 3 | WBPH | IR66 | 6 | 77.44 | 109.47 | 54.54 |
| 3 | WBPH | IR66 | 7 | 82.87 | 114.95 | 56.78 |
| 3 | WBPH | IR66 | 8 | 88.30 | 120.43 | 59.02 |
| 3 | WBPH | IR66 | 9 | 94.38 | 123.35 | 62.64 |
| 3 | WBPH | IR66 | 10 | 105.54 | 136.71 | 73.14 |
| 1 | BPH | IR70 | 1 | 77.45 | 110.54 | 58.86 |
| 1 | BPH | IR70 | 2 | 60.03 | 91.86 | 61.14 |
| 1 | BPH | IR70 | 3 | 69.20 | 96.77 | 57.94 |
| 1 | BPH | IR70 | 4 | 73.92 | 92.85 | 60.74 |
| 1 | BPH | IR70 | 5 | 77.58 | 95.75 | 63.14 |
| 1 | BPH | IR70 | 6 | 88.33 | 102.09 | 85.01 |
| 1 | BPH | IR70 | 7 | 119.12 | 116.27 | 110.67 |
| 1 | BPH | IR70 | 8 | 116.76 | 112.23 | 106.26 |
| 1 | BPH | IR70 | 9 | 113.42 | 105.48 | 98.79 |
| 1 | BPH | IR70 | 10 | 90.04 | 85.98 | 83.69 |
| 1 | CONTROL | IR70 | 1 | 56.33 | 88.13 | 46.30 |
| 1 | CONTROL | IR70 | 2 | 66.93 | 97.51 | 55.13 |
| 1 | CONTROL | IR70 | 3 | 65.96 | 99.22 | 52.00 |
| 1 | CONTROL | IR70 | 4 | 66.08 | 99.88 | 56.34 |
| 1 | CONTROL | IR70 | 5 | 65.04 | 96.98 | 53.58 |
| 1 | CONTROL | IR70 | 6 | 62.64 | 94.51 | 56.20 |
| 1 | CONTROL | IR70 | 7 | 62.01 | 92.84 | 51.25 |
| 1 | CONTROL | IR70 | 8 | 69.08 | 102.03 | 59.16 |
| 1 | CONTROL | IR70 | 9 | 93.16 | 126.18 | 77.04 |
| 1 | CONTROL | IR70 | 10 | 70.44 | 99.48 | 56.66 |
| 1 | WBPH | IR70 | 1 | 95.54 | 131.87 | 71.88 |
| 1 | WBPH | IR70 | 2 | 72.93 | 101.48 | 57.64 |
| 1 | WBPH | IR70 | 3 | 83.91 | 117.42 | 67.66 |
| 1 | WBPH | IR70 | 4 | 94.00 | 119.32 | 62.75 |
| 1 | WBPH | IR70 | 5 | 82.65 | 108.99 | 64.52 |
| 1 | WBPH | IR70 | 6 | 97.41 | 118.46 | 67.14 |
| 1 | WBPH | IR70 | 7 | 88.70 | 102.33 | 65.45 |
| 1 | WBPH | IR70 | 8 | 106.94 | 117.92 | 78.29 |
| 1 | WBPH | IR70 | 9 | 108.86 | 116.70 | 79.53 |
| 1 | WBPH | IR70 | 10 | 97.65 | 107.07 | 82.13 |
| 2 | BPH | IR70 | 1 | 59.89 | 90.98 | 54.74 |
| 2 | BPH | IR70 | 2 | 62.94 | 88.88 | 49.39 |
| 2 | BPH | IR70 | 3 | 65.99 | 86.77 | 44.04 |
| 2 | BPH | IR70 | 4 | 65.54 | 83.56 | 54.43 |
| 2 | BPH | IR70 | 5 | 65.08 | 80.35 | 64.82 |
| 2 | BPH | IR70 | 6 | 103.25 | 108.89 | 102.84 |
| 2 | BPH | IR70 | 7 | 119.79 | 119.90 | 114.11 |
| 2 | BPH | IR70 | 8 | 136.33 | 130.91 | 125.37 |
| 2 | BPH | IR70 | 9 | 134.67 | 129.31 | 123.89 |
| 2 | BPH | IR70 | 10 | 133.01 | 127.70 | 122.41 |
| 2 | CONTROL | IR70 | 1 | 55.60 | 88.75 | 47.34 |
| 2 | CONTROL | IR70 | 2 | 56.12 | 92.54 | 58.33 |
| 2 | CONTROL | IR70 | 3 | 56.64 | 96.33 | 69.32 |
| 2 | CONTROL | IR70 | 4 | 54.60 | 89.86 | 57.12 |
| 2 | CONTROL | IR70 | 5 | 52.55 | 83.38 | 44.91 |
| 2 | CONTROL | IR70 | 6 | 62.04 | 84.87 | 41.89 |
| 2 | CONTROL | IR70 | 7 | 67.81 | 92.82 | 45.31 |
| 2 | CONTROL | IR70 | 8 | 73.57 | 100.77 | 48.73 |
| 2 | CONTROL | IR70 | 9 | 69.36 | 94.23 | 43.30 |
| 2 | CONTROL | IR70 | 10 | 65.14 | 87.69 | 37.86 |
| 2 | WBPH | IR70 | 1 | 59.46 | 85.33 | 49.57 |
| 2 | WBPH | IR70 | 2 | 63.00 | 87.37 | 51.17 |
| 2 | WBPH | IR70 | 3 | 66.54 | 89.41 | 52.76 |
| 2 | WBPH | IR70 | 4 | 73.77 | 93.73 | 53.55 |
| 2 | WBPH | IR70 | 5 | 69.98 | 76.52 | 49.06 |
| 2 | WBPH | IR70 | 6 | 69.99 | 73.13 | 65.04 |
| 2 | WBPH | IR70 | 7 | 80.18 | 90.71 | 70.38 |
| 2 | WBPH | IR70 | 8 | 102.34 | 98.03 | 93.47 |
| 2 | WBPH | IR70 | 9 | 107.65 | 102.02 | 94.85 |
| 2 | WBPH | IR70 | 10 | 112.95 | 106.01 | 96.23 |
| 3 | BPH | IR70 | 1 | 75.33 | 104.68 | 56.31 |
| 3 | BPH | IR70 | 2 | 75.55 | 106.55 | 57.23 |
| 3 | BPH | IR70 | 3 | 75.76 | 108.42 | 58.14 |
| 3 | BPH | IR70 | 4 | 70.00 | 101.27 | 57.21 |
| 3 | BPH | IR70 | 5 | 64.23 | 94.11 | 56.27 |
| 3 | BPH | IR70 | 6 | 65.13 | 89.80 | 57.32 |
| 3 | BPH | IR70 | 7 | 74.27 | 97.21 | 64.66 |
| 3 | BPH | IR70 | 8 | 83.40 | 104.61 | 72.00 |
| 3 | BPH | IR70 | 9 | 105.05 | 116.42 | 79.30 |
| 3 | BPH | IR70 | 10 | 115.14 | 127.56 | 90.61 |
| 3 | CONTROL | IR70 | 1 | 77.28 | 109.59 | 55.38 |
| 3 | CONTROL | IR70 | 2 | 82.64 | 118.30 | 57.31 |
| 3 | CONTROL | IR70 | 3 | 88.00 | 127.00 | 59.24 |
| 3 | CONTROL | IR70 | 4 | 84.78 | 121.45 | 54.00 |
| 3 | CONTROL | IR70 | 5 | 81.55 | 115.90 | 48.76 |
| 3 | CONTROL | IR70 | 6 | 77.96 | 107.89 | 40.65 |
| 3 | CONTROL | IR70 | 7 | 82.31 | 114.59 | 45.69 |
| 3 | CONTROL | IR70 | 8 | 86.66 | 121.29 | 50.72 |
| 3 | CONTROL | IR70 | 9 | 101.35 | 133.68 | 52.82 |
| 3 | CONTROL | IR70 | 10 | 96.75 | 134.49 | 66.17 |
| 3 | WBPH | IR70 | 1 | 67.96 | 101.25 | 57.61 |
| 3 | WBPH | IR70 | 2 | 68.73 | 96.74 | 53.75 |
| 3 | WBPH | IR70 | 3 | 69.50 | 92.22 | 49.89 |
| 3 | WBPH | IR70 | 4 | 66.89 | 91.48 | 47.57 |
| 3 | WBPH | IR70 | 5 | 64.27 | 90.74 | 45.25 |
| 3 | WBPH | IR70 | 6 | 82.55 | 116.54 | 57.93 |
| 3 | WBPH | IR70 | 7 | 85.90 | 119.05 | 61.77 |
| 3 | WBPH | IR70 | 8 | 89.24 | 121.55 | 65.60 |
| 3 | WBPH | IR70 | 9 | 79.70 | 106.09 | 64.91 |
| 3 | WBPH | IR70 | 10 | 111.38 | 132.87 | 75.25 |
| 1 | BPH | IR71033 | 1 | 88.16 | 119.90 | 62.33 |
| 1 | BPH | IR71033 | 2 | 95.02 | 128.86 | 78.78 |
| 1 | BPH | IR71033 | 3 | 90.20 | 114.42 | 61.99 |
| 1 | BPH | IR71033 | 4 | 85.93 | 111.86 | 74.08 |
| 1 | BPH | IR71033 | 5 | 102.83 | 123.15 | 71.35 |
| 1 | BPH | IR71033 | 6 | 101.98 | 116.62 | 74.22 |
| 1 | BPH | IR71033 | 7 | 102.97 | 120.44 | 68.80 |
| 1 | BPH | IR71033 | 8 | 103.00 | 121.97 | 87.77 |
| 1 | BPH | IR71033 | 9 | 110.72 | 122.96 | 79.52 |
| 1 | BPH | IR71033 | 10 | 95.54 | 98.54 | 82.48 |
| 1 | CONTROL | IR71033 | 1 | 93.35 | 124.44 | 58.75 |
| 1 | CONTROL | IR71033 | 2 | 85.75 | 126.11 | 75.60 |
| 1 | CONTROL | IR71033 | 3 | 96.12 | 133.54 | 64.07 |
| 1 | CONTROL | IR71033 | 4 | 77.67 | 112.73 | 61.77 |
| 1 | CONTROL | IR71033 | 5 | 95.59 | 127.30 | 56.21 |
| 1 | CONTROL | IR71033 | 6 | 78.64 | 112.26 | 64.91 |
| 1 | CONTROL | IR71033 | 7 | 84.94 | 115.30 | 60.09 |
| 1 | CONTROL | IR71033 | 8 | 96.40 | 129.08 | 71.32 |
| 1 | CONTROL | IR71033 | 9 | 93.82 | 125.36 | 65.39 |
| 1 | CONTROL | IR71033 | 10 | 103.09 | 134.55 | 70.88 |
| 1 | WBPH | IR71033 | 1 | 86.25 | 116.32 | 62.99 |
| 1 | WBPH | IR71033 | 2 | 76.75 | 104.10 | 64.01 |
| 1 | WBPH | IR71033 | 3 | 77.76 | 103.40 | 61.60 |
| 1 | WBPH | IR71033 | 4 | 103.43 | 132.22 | 81.21 |
| 1 | WBPH | IR71033 | 5 | 100.02 | 123.19 | 80.02 |
| 1 | WBPH | IR71033 | 6 | 90.12 | 112.58 | 78.44 |
| 1 | WBPH | IR71033 | 7 | 81.33 | 99.50 | 69.67 |
| 1 | WBPH | IR71033 | 8 | 105.57 | 122.03 | 92.27 |
| 1 | WBPH | IR71033 | 9 | 96.64 | 110.12 | 85.71 |
| 1 | WBPH | IR71033 | 10 | 90.09 | 99.79 | 75.94 |
| 2 | BPH | IR71033 | 1 | 58.04 | 86.00 | 52.50 |
| 2 | BPH | IR71033 | 2 | 59.37 | 87.50 | 53.93 |
| 2 | BPH | IR71033 | 3 | 60.70 | 88.99 | 55.36 |
| 2 | BPH | IR71033 | 4 | 59.26 | 85.54 | 54.83 |
| 2 | BPH | IR71033 | 5 | 57.82 | 82.08 | 54.30 |
| 2 | BPH | IR71033 | 6 | 74.00 | 101.06 | 63.97 |
| 2 | BPH | IR71033 | 7 | 81.54 | 107.27 | 71.30 |
| 2 | BPH | IR71033 | 8 | 89.07 | 113.47 | 78.63 |
| 2 | BPH | IR71033 | 9 | 79.06 | 103.14 | 74.13 |
| 2 | BPH | IR71033 | 10 | 69.04 | 92.80 | 69.62 |
| 2 | CONTROL | IR71033 | 1 | 91.51 | 116.97 | 49.92 |
| 2 | CONTROL | IR71033 | 2 | 82.53 | 114.69 | 60.63 |
| 2 | CONTROL | IR71033 | 3 | 73.54 | 112.41 | 71.33 |
| 2 | CONTROL | IR71033 | 4 | 82.64 | 119.70 | 61.99 |
| 2 | CONTROL | IR71033 | 5 | 91.74 | 126.99 | 52.64 |
| 2 | CONTROL | IR71033 | 6 | 84.31 | 117.59 | 47.27 |
| 2 | CONTROL | IR71033 | 7 | 89.45 | 123.03 | 47.74 |
| 2 | CONTROL | IR71033 | 8 | 94.59 | 128.46 | 48.21 |
| 2 | CONTROL | IR71033 | 9 | 92.71 | 126.86 | 51.03 |
| 2 | CONTROL | IR71033 | 10 | 90.82 | 125.26 | 53.85 |
| 2 | WBPH | IR71033 | 1 | 70.50 | 103.27 | 63.83 |
| 2 | WBPH | IR71033 | 2 | 65.08 | 96.85 | 59.65 |
| 2 | WBPH | IR71033 | 3 | 59.65 | 90.43 | 55.46 |
| 2 | WBPH | IR71033 | 4 | 62.98 | 87.78 | 52.63 |
| 2 | WBPH | IR71033 | 5 | 59.41 | 86.15 | 52.49 |
| 2 | WBPH | IR71033 | 6 | 75.72 | 99.83 | 54.79 |
| 2 | WBPH | IR71033 | 7 | 78.41 | 91.49 | 65.44 |
| 2 | WBPH | IR71033 | 8 | 86.82 | 109.84 | 65.84 |
| 2 | WBPH | IR71033 | 9 | 80.92 | 101.61 | 58.86 |
| 2 | WBPH | IR71033 | 10 | 75.02 | 93.37 | 51.87 |
| 3 | BPH | IR71033 | 1 | 101.72 | 132.53 | 59.43 |
| 3 | BPH | IR71033 | 2 | 102.66 | 135.95 | 65.39 |
| 3 | BPH | IR71033 | 3 | 103.59 | 139.36 | 71.34 |
| 3 | BPH | IR71033 | 4 | 99.33 | 131.07 | 64.24 |
| 3 | BPH | IR71033 | 5 | 95.07 | 122.77 | 57.14 |
| 3 | BPH | IR71033 | 6 | 84.21 | 114.97 | 57.27 |
| 3 | BPH | IR71033 | 7 | 92.45 | 124.14 | 59.73 |
| 3 | BPH | IR71033 | 8 | 100.69 | 133.30 | 62.18 |
| 3 | BPH | IR71033 | 9 | 94.42 | 115.14 | 54.84 |
| 3 | BPH | IR71033 | 10 | 124.08 | 153.39 | 81.02 |
| 3 | CONTROL | IR71033 | 1 | 75.85 | 111.91 | 54.55 |
| 3 | CONTROL | IR71033 | 2 | 85.70 | 122.95 | 60.03 |
| 3 | CONTROL | IR71033 | 3 | 95.55 | 133.99 | 65.50 |
| 3 | CONTROL | IR71033 | 4 | 96.79 | 134.25 | 59.38 |
| 3 | CONTROL | IR71033 | 5 | 98.03 | 134.50 | 53.26 |
| 3 | CONTROL | IR71033 | 6 | 85.31 | 118.86 | 48.05 |
| 3 | CONTROL | IR71033 | 7 | 83.37 | 116.95 | 49.98 |
| 3 | CONTROL | IR71033 | 8 | 81.42 | 115.04 | 51.91 |
| 3 | CONTROL | IR71033 | 9 | 79.11 | 111.03 | 48.86 |
| 3 | CONTROL | IR71033 | 10 | 123.40 | 158.89 | 59.59 |
| 3 | WBPH | IR71033 | 1 | 84.94 | 119.48 | 65.65 |
| 3 | WBPH | IR71033 | 2 | 80.05 | 113.13 | 63.21 |
| 3 | WBPH | IR71033 | 3 | 75.15 | 106.78 | 60.77 |
| 3 | WBPH | IR71033 | 4 | 79.13 | 108.97 | 61.82 |
| 3 | WBPH | IR71033 | 5 | 83.10 | 111.16 | 62.86 |
| 3 | WBPH | IR71033 | 6 | 85.84 | 111.38 | 59.92 |
| 3 | WBPH | IR71033 | 7 | 90.49 | 114.46 | 59.51 |
| 3 | WBPH | IR71033 | 8 | 95.14 | 117.54 | 59.10 |
| 3 | WBPH | IR71033 | 9 | 91.52 | 109.15 | 69.51 |
| 3 | WBPH | IR71033 | 10 | 117.25 | 135.55 | 88.81 |
| 1 | BPH | IR72 | 1 | 96.72 | 133.71 | 75.04 |
| 1 | BPH | IR72 | 2 | 75.01 | 103.51 | 64.85 |
| 1 | BPH | IR72 | 3 | 89.18 | 123.74 | 72.56 |
| 1 | BPH | IR72 | 4 | 82.39 | 115.48 | 65.02 |
| 1 | BPH | IR72 | 5 | 83.76 | 110.68 | 66.69 |
| 1 | BPH | IR72 | 6 | 89.07 | 119.76 | 82.23 |
| 1 | BPH | IR72 | 7 | 92.07 | 120.64 | 86.71 |
| 1 | BPH | IR72 | 8 | 104.01 | 126.99 | 84.83 |
| 1 | BPH | IR72 | 9 | 91.19 | 107.89 | 72.49 |
| 1 | BPH | IR72 | 10 | 81.00 | 98.70 | 78.40 |
| 1 | CONTROL | IR72 | 1 | 84.21 | 121.93 | 57.90 |
| 1 | CONTROL | IR72 | 2 | 78.11 | 118.30 | 68.50 |
| 1 | CONTROL | IR72 | 3 | 75.52 | 109.43 | 52.19 |
| 1 | CONTROL | IR72 | 4 | 72.21 | 107.34 | 54.89 |
| 1 | CONTROL | IR72 | 5 | 93.24 | 123.07 | 45.10 |
| 1 | CONTROL | IR72 | 6 | 86.15 | 122.47 | 62.71 |
| 1 | CONTROL | IR72 | 7 | 102.47 | 125.58 | 54.94 |
| 1 | CONTROL | IR72 | 8 | 84.97 | 118.61 | 53.64 |
| 1 | CONTROL | IR72 | 9 | 95.42 | 127.96 | 56.25 |
| 1 | CONTROL | IR72 | 10 | 89.83 | 122.29 | 54.02 |
| 1 | WBPH | IR72 | 1 | 96.63 | 125.06 | 61.13 |
| 1 | WBPH | IR72 | 2 | 84.46 | 111.06 | 56.90 |
| 1 | WBPH | IR72 | 3 | 93.64 | 116.32 | 53.77 |
| 1 | WBPH | IR72 | 4 | 91.16 | 124.32 | 66.10 |
| 1 | WBPH | IR72 | 5 | 97.93 | 118.38 | 55.40 |
| 1 | WBPH | IR72 | 6 | 89.67 | 116.37 | 70.52 |
| 1 | WBPH | IR72 | 7 | 99.31 | 116.09 | 64.95 |
| 1 | WBPH | IR72 | 8 | 97.84 | 118.74 | 71.84 |
| 1 | WBPH | IR72 | 9 | 81.84 | 101.68 | 68.97 |
| 1 | WBPH | IR72 | 10 | 88.05 | 104.59 | 72.22 |
| 2 | BPH | IR72 | 1 | 84.17 | 114.02 | 51.51 |
| 2 | BPH | IR72 | 2 | 79.38 | 107.10 | 47.90 |
| 2 | BPH | IR72 | 3 | 74.59 | 100.18 | 44.29 |
| 2 | BPH | IR72 | 4 | 67.81 | 91.39 | 48.67 |
| 2 | BPH | IR72 | 5 | 61.02 | 82.59 | 53.05 |
| 2 | BPH | IR72 | 6 | 83.14 | 93.66 | 78.02 |
| 2 | BPH | IR72 | 7 | 100.83 | 106.16 | 94.10 |
| 2 | BPH | IR72 | 8 | 118.52 | 118.66 | 110.18 |
| 2 | BPH | IR72 | 9 | 119.16 | 117.83 | 110.95 |
| 2 | BPH | IR72 | 10 | 119.80 | 116.99 | 111.71 |
| 2 | CONTROL | IR72 | 1 | 69.97 | 92.99 | 45.28 |
| 2 | CONTROL | IR72 | 2 | 70.75 | 95.32 | 47.90 |
| 2 | CONTROL | IR72 | 3 | 71.52 | 97.65 | 50.51 |
| 2 | CONTROL | IR72 | 4 | 91.02 | 121.40 | 53.52 |
| 2 | CONTROL | IR72 | 5 | 110.52 | 145.14 | 56.52 |
| 2 | CONTROL | IR72 | 6 | 94.28 | 129.99 | 53.91 |
| 2 | CONTROL | IR72 | 7 | 102.16 | 135.70 | 58.53 |
| 2 | CONTROL | IR72 | 8 | 110.04 | 141.41 | 63.15 |
| 2 | CONTROL | IR72 | 9 | 108.96 | 137.37 | 56.49 |
| 2 | CONTROL | IR72 | 10 | 107.88 | 133.33 | 49.82 |
| 2 | WBPH | IR72 | 1 | 69.56 | 100.96 | 52.70 |
| 2 | WBPH | IR72 | 2 | 70.29 | 101.92 | 55.84 |
| 2 | WBPH | IR72 | 3 | 71.01 | 102.87 | 58.98 |
| 2 | WBPH | IR72 | 4 | 74.99 | 102.05 | 54.33 |
| 2 | WBPH | IR72 | 5 | 90.33 | 113.66 | 53.19 |
| 2 | WBPH | IR72 | 6 | 87.85 | 108.43 | 49.94 |
| 2 | WBPH | IR72 | 7 | 85.70 | 107.95 | 60.95 |
| 2 | WBPH | IR72 | 8 | 95.68 | 116.06 | 67.11 |
| 2 | WBPH | IR72 | 9 | 95.26 | 112.53 | 70.90 |
| 2 | WBPH | IR72 | 10 | 94.84 | 109.00 | 74.69 |
| 3 | BPH | IR72 | 1 | 93.71 | 129.86 | 58.64 |
| 3 | BPH | IR72 | 2 | 92.32 | 125.89 | 54.51 |
| 3 | BPH | IR72 | 3 | 90.93 | 121.92 | 50.38 |
| 3 | BPH | IR72 | 4 | 89.76 | 121.91 | 54.98 |
| 3 | BPH | IR72 | 5 | 88.59 | 121.90 | 59.58 |
| 3 | BPH | IR72 | 6 | 80.76 | 111.48 | 57.85 |
| 3 | BPH | IR72 | 7 | 82.32 | 112.02 | 59.51 |
| 3 | BPH | IR72 | 8 | 83.87 | 112.55 | 61.16 |
| 3 | BPH | IR72 | 9 | 91.09 | 118.14 | 59.86 |
| 3 | BPH | IR72 | 10 | 115.25 | 139.09 | 75.66 |
| 3 | CONTROL | IR72 | 1 | 87.78 | 119.48 | 50.43 |
| 3 | CONTROL | IR72 | 2 | 83.75 | 116.75 | 51.69 |
| 3 | CONTROL | IR72 | 3 | 79.71 | 114.01 | 52.95 |
| 3 | CONTROL | IR72 | 4 | 77.49 | 111.73 | 50.77 |
| 3 | CONTROL | IR72 | 5 | 75.26 | 109.45 | 48.58 |
| 3 | CONTROL | IR72 | 6 | 85.96 | 113.14 | 40.79 |
| 3 | CONTROL | IR72 | 7 | 91.43 | 123.10 | 47.74 |
| 3 | CONTROL | IR72 | 8 | 96.89 | 133.06 | 54.68 |
| 3 | CONTROL | IR72 | 9 | 99.34 | 134.16 | 53.77 |
| 3 | CONTROL | IR72 | 10 | 94.45 | 129.77 | 61.99 |
| 3 | WBPH | IR72 | 1 | 87.87 | 122.99 | 62.19 |
| 3 | WBPH | IR72 | 2 | 86.18 | 120.36 | 57.84 |
| 3 | WBPH | IR72 | 3 | 84.49 | 117.72 | 53.49 |
| 3 | WBPH | IR72 | 4 | 87.06 | 117.68 | 55.53 |
| 3 | WBPH | IR72 | 5 | 89.62 | 117.63 | 57.57 |
| 3 | WBPH | IR72 | 6 | 92.39 | 118.48 | 53.35 |
| 3 | WBPH | IR72 | 7 | 98.16 | 123.94 | 59.75 |
| 3 | WBPH | IR72 | 8 | 103.93 | 129.39 | 66.15 |
| 3 | WBPH | IR72 | 9 | 117.25 | 137.86 | 81.15 |
| 3 | WBPH | IR72 | 10 | 140.09 | 154.21 | 89.83 |
| 1 | BPH | IR74 | 1 | 96.63 | 134.86 | 69.93 |
| 1 | BPH | IR74 | 2 | 65.65 | 98.67 | 57.29 |
| 1 | BPH | IR74 | 3 | 90.97 | 125.61 | 66.84 |
| 1 | BPH | IR74 | 4 | 86.02 | 107.63 | 51.59 |
| 1 | BPH | IR74 | 5 | 101.76 | 121.32 | 56.86 |
| 1 | BPH | IR74 | 6 | 103.77 | 123.82 | 69.50 |
| 1 | BPH | IR74 | 7 | 119.27 | 139.88 | 83.79 |
| 1 | BPH | IR74 | 8 | 112.18 | 122.66 | 81.99 |
| 1 | BPH | IR74 | 9 | 90.96 | 99.68 | 67.86 |
| 1 | BPH | IR74 | 10 | 98.53 | 108.58 | 76.75 |
| 1 | CONTROL | IR74 | 1 | 78.88 | 116.10 | 58.83 |
| 1 | CONTROL | IR74 | 2 | 70.67 | 109.18 | 68.51 |
| 1 | CONTROL | IR74 | 3 | 79.51 | 114.17 | 59.57 |
| 1 | CONTROL | IR74 | 4 | 66.97 | 98.96 | 53.04 |
| 1 | CONTROL | IR74 | 5 | 70.15 | 102.82 | 54.26 |
| 1 | CONTROL | IR74 | 6 | 67.64 | 98.37 | 54.80 |
| 1 | CONTROL | IR74 | 7 | 62.99 | 93.86 | 54.32 |
| 1 | CONTROL | IR74 | 8 | 93.21 | 116.36 | 64.64 |
| 1 | CONTROL | IR74 | 9 | 89.08 | 118.69 | 67.00 |
| 1 | CONTROL | IR74 | 10 | 89.21 | 107.28 | 60.46 |
| 1 | WBPH | IR74 | 1 | 93.18 | 124.70 | 61.02 |
| 1 | WBPH | IR74 | 2 | 76.88 | 100.89 | 49.95 |
| 1 | WBPH | IR74 | 3 | 88.66 | 113.43 | 58.09 |
| 1 | WBPH | IR74 | 4 | 99.89 | 120.76 | 59.05 |
| 1 | WBPH | IR74 | 5 | 92.67 | 106.30 | 56.88 |
| 1 | WBPH | IR74 | 6 | 121.39 | 131.34 | 76.01 |
| 1 | WBPH | IR74 | 7 | 108.32 | 110.47 | 64.11 |
| 1 | WBPH | IR74 | 8 | 119.32 | 125.60 | 90.71 |
| 1 | WBPH | IR74 | 9 | 95.21 | 101.30 | 79.57 |
| 1 | WBPH | IR74 | 10 | 100.66 | 107.55 | 83.01 |
| 2 | BPH | IR74 | 1 | 57.29 | 86.36 | 49.58 |
| 2 | BPH | IR74 | 2 | 55.32 | 82.68 | 46.13 |
| 2 | BPH | IR74 | 3 | 53.34 | 78.99 | 42.68 |
| 2 | BPH | IR74 | 4 | 59.96 | 86.06 | 45.24 |
| 2 | BPH | IR74 | 5 | 66.58 | 93.12 | 47.80 |
| 2 | BPH | IR74 | 6 | 73.04 | 97.08 | 52.28 |
| 2 | BPH | IR74 | 7 | 86.82 | 110.70 | 63.61 |
| 2 | BPH | IR74 | 8 | 100.59 | 124.31 | 74.93 |
| 2 | BPH | IR74 | 9 | 93.55 | 115.17 | 71.83 |
| 2 | BPH | IR74 | 10 | 86.50 | 106.02 | 68.72 |
| 2 | CONTROL | IR74 | 1 | 73.07 | 98.41 | 41.69 |
| 2 | CONTROL | IR74 | 2 | 66.69 | 98.90 | 54.56 |
| 2 | CONTROL | IR74 | 3 | 60.30 | 99.39 | 67.43 |
| 2 | CONTROL | IR74 | 4 | 66.35 | 101.99 | 55.32 |
| 2 | CONTROL | IR74 | 5 | 72.39 | 104.58 | 43.21 |
| 2 | CONTROL | IR74 | 6 | 79.18 | 114.19 | 49.04 |
| 2 | CONTROL | IR74 | 7 | 91.04 | 127.39 | 57.76 |
| 2 | CONTROL | IR74 | 8 | 102.89 | 140.58 | 66.47 |
| 2 | CONTROL | IR74 | 9 | 92.17 | 127.91 | 57.67 |
| 2 | CONTROL | IR74 | 10 | 81.44 | 115.24 | 48.87 |
| 2 | WBPH | IR74 | 1 | 75.81 | 104.33 | 51.38 |
| 2 | WBPH | IR74 | 2 | 69.43 | 98.57 | 50.20 |
| 2 | WBPH | IR74 | 3 | 63.04 | 92.80 | 49.01 |
| 2 | WBPH | IR74 | 4 | 70.63 | 101.09 | 55.62 |
| 2 | WBPH | IR74 | 5 | 70.25 | 99.30 | 52.26 |
| 2 | WBPH | IR74 | 6 | 79.55 | 101.74 | 60.51 |
| 2 | WBPH | IR74 | 7 | 91.07 | 109.57 | 62.89 |
| 2 | WBPH | IR74 | 8 | 94.29 | 110.71 | 75.83 |
| 2 | WBPH | IR74 | 9 | 97.25 | 110.49 | 79.07 |
| 2 | WBPH | IR74 | 10 | 100.21 | 110.26 | 82.31 |
| 3 | BPH | IR74 | 1 | 88.32 | 114.88 | 52.10 |
| 3 | BPH | IR74 | 2 | 88.16 | 119.03 | 56.93 |
| 3 | BPH | IR74 | 3 | 88.00 | 123.17 | 61.75 |
| 3 | BPH | IR74 | 4 | 86.24 | 118.66 | 54.53 |
| 3 | BPH | IR74 | 5 | 84.47 | 114.15 | 47.31 |
| 3 | BPH | IR74 | 6 | 91.46 | 115.36 | 48.06 |
| 3 | BPH | IR74 | 7 | 95.81 | 122.30 | 51.25 |
| 3 | BPH | IR74 | 8 | 100.15 | 129.23 | 54.44 |
| 3 | BPH | IR74 | 9 | 110.91 | 128.12 | 59.65 |
| 3 | BPH | IR74 | 10 | 125.00 | 138.08 | 74.61 |
| 3 | CONTROL | IR74 | 1 | 85.39 | 114.71 | 50.58 |
| 3 | CONTROL | IR74 | 2 | 86.45 | 120.32 | 55.24 |
| 3 | CONTROL | IR74 | 3 | 87.51 | 125.93 | 59.89 |
| 3 | CONTROL | IR74 | 4 | 77.93 | 112.60 | 50.98 |
| 3 | CONTROL | IR74 | 5 | 68.35 | 99.27 | 42.07 |
| 3 | CONTROL | IR74 | 6 | 74.76 | 105.96 | 44.03 |
| 3 | CONTROL | IR74 | 7 | 79.86 | 111.89 | 45.92 |
| 3 | CONTROL | IR74 | 8 | 84.95 | 117.81 | 47.80 |
| 3 | CONTROL | IR74 | 9 | 82.24 | 115.38 | 49.51 |
| 3 | CONTROL | IR74 | 10 | 97.89 | 134.11 | 65.23 |
| 3 | WBPH | IR74 | 1 | 79.31 | 111.65 | 56.73 |
| 3 | WBPH | IR74 | 2 | 81.01 | 112.82 | 56.65 |
| 3 | WBPH | IR74 | 3 | 82.70 | 113.98 | 56.56 |
| 3 | WBPH | IR74 | 4 | 82.32 | 110.72 | 55.13 |
| 3 | WBPH | IR74 | 5 | 81.93 | 107.45 | 53.70 |
| 3 | WBPH | IR74 | 6 | 96.92 | 111.26 | 48.24 |
| 3 | WBPH | IR74 | 7 | 103.70 | 118.12 | 53.65 |
| 3 | WBPH | IR74 | 8 | 110.47 | 124.98 | 59.05 |
| 3 | WBPH | IR74 | 9 | 105.28 | 116.76 | 63.87 |
| 3 | WBPH | IR74 | 10 | 127.72 | 136.22 | 87.56 |
| 1 | BPH | JiaNong66 | 1 | 55.60 | 96.88 | 77.38 |
| 1 | BPH | JiaNong66 | 2 | 51.99 | 98.37 | 90.74 |
| 1 | BPH | JiaNong66 | 3 | 58.08 | 106.46 | 92.66 |
| 1 | BPH | JiaNong66 | 4 | 67.31 | 99.21 | 70.71 |
| 1 | BPH | JiaNong66 | 5 | 62.62 | 107.18 | 90.69 |
| 1 | BPH | JiaNong66 | 6 | 80.49 | 108.98 | 81.34 |
| 1 | BPH | JiaNong66 | 7 | 65.93 | 108.98 | 92.71 |
| 1 | BPH | JiaNong66 | 8 | 80.45 | 108.80 | 81.70 |
| 1 | BPH | JiaNong66 | 9 | 114.13 | 124.82 | 124.89 |
| 1 | BPH | JiaNong66 | 10 | 89.70 | 95.99 | 93.55 |
| 1 | CONTROL | JiaNong66 | 1 | 70.20 | 107.28 | 64.65 |
| 1 | CONTROL | JiaNong66 | 2 | 84.81 | 121.12 | 84.32 |
| 1 | CONTROL | JiaNong66 | 3 | 67.34 | 110.09 | 78.99 |
| 1 | CONTROL | JiaNong66 | 4 | 66.65 | 105.96 | 71.24 |
| 1 | CONTROL | JiaNong66 | 5 | 80.33 | 123.16 | 76.60 |
| 1 | CONTROL | JiaNong66 | 6 | 73.45 | 99.82 | 55.06 |
| 1 | CONTROL | JiaNong66 | 7 | 77.65 | 109.88 | 58.66 |
| 1 | CONTROL | JiaNong66 | 8 | 85.39 | 126.61 | 78.65 |
| 1 | CONTROL | JiaNong66 | 9 | 86.26 | 126.65 | 79.51 |
| 1 | CONTROL | JiaNong66 | 10 | 75.22 | 114.17 | 76.75 |
| 3 | BPH | JiaNong66 | 1 | 60.54 | 92.53 | 67.47 |
| 3 | BPH | JiaNong66 | 2 | 69.58 | 104.49 | 73.39 |
| 3 | BPH | JiaNong66 | 3 | 78.61 | 116.45 | 79.31 |
| 3 | BPH | JiaNong66 | 4 | 74.59 | 109.22 | 72.79 |
| 3 | BPH | JiaNong66 | 5 | 70.56 | 101.98 | 66.27 |
| 3 | BPH | JiaNong66 | 6 | 79.05 | 110.92 | 89.49 |
| 3 | BPH | JiaNong66 | 7 | 97.72 | 120.60 | 105.17 |
| 3 | BPH | JiaNong66 | 8 | 116.39 | 130.28 | 120.85 |
| 3 | BPH | JiaNong66 | 9 | 151.17 | 148.51 | 144.42 |
| 3 | BPH | JiaNong66 | 10 | 157.00 | 152.24 | 147.53 |
| 1 | BPH | MOI | 1 | 118.08 | 151.45 | 79.93 |
| 1 | BPH | MOI | 2 | 90.53 | 126.06 | 80.14 |
| 1 | BPH | MOI | 3 | 97.75 | 130.20 | 79.96 |
| 1 | BPH | MOI | 4 | 101.24 | 134.88 | 86.52 |
| 1 | BPH | MOI | 5 | 102.10 | 127.80 | 68.76 |
| 1 | BPH | MOI | 6 | 96.97 | 128.38 | 87.36 |
| 1 | BPH | MOI | 7 | 109.48 | 134.30 | 85.03 |
| 1 | BPH | MOI | 8 | 113.44 | 143.59 | 108.93 |
| 1 | BPH | MOI | 9 | 103.15 | 121.60 | 93.38 |
| 1 | BPH | MOI | 10 | 114.26 | 130.23 | 83.51 |
| 1 | CONTROL | MOI | 1 | 109.80 | 148.10 | 77.35 |
| 1 | CONTROL | MOI | 2 | 100.37 | 138.27 | 82.86 |
| 1 | CONTROL | MOI | 3 | 104.79 | 140.87 | 78.68 |
| 1 | CONTROL | MOI | 4 | 92.66 | 119.94 | 68.95 |
| 1 | CONTROL | MOI | 5 | 97.87 | 128.60 | 66.88 |
| 1 | CONTROL | MOI | 6 | 85.68 | 118.89 | 72.77 |
| 1 | CONTROL | MOI | 7 | 100.27 | 131.85 | 72.91 |
| 1 | CONTROL | MOI | 8 | 114.29 | 147.15 | 88.94 |
| 1 | CONTROL | MOI | 9 | 119.62 | 142.80 | 81.03 |
| 1 | CONTROL | MOI | 10 | 100.22 | 128.51 | 75.72 |
| 1 | WBPH | MOI | 1 | 118.88 | 155.42 | 76.75 |
| 1 | WBPH | MOI | 2 | 87.32 | 120.43 | 65.86 |
| 1 | WBPH | MOI | 3 | 102.17 | 137.31 | 78.66 |
| 1 | WBPH | MOI | 4 | 100.26 | 125.24 | 65.12 |
| 1 | WBPH | MOI | 5 | 102.73 | 132.42 | 71.28 |
| 1 | WBPH | MOI | 6 | 105.54 | 132.93 | 77.15 |
| 1 | WBPH | MOI | 7 | 100.63 | 123.66 | 72.62 |
| 1 | WBPH | MOI | 8 | 130.29 | 151.84 | 90.12 |
| 1 | WBPH | MOI | 9 | 130.60 | 150.51 | 83.19 |
| 1 | WBPH | MOI | 10 | 124.15 | 147.28 | 96.22 |
| 2 | BPH | MOI | 1 | 78.68 | 108.33 | 56.99 |
| 2 | BPH | MOI | 2 | 79.35 | 108.43 | 55.17 |
| 2 | BPH | MOI | 3 | 80.02 | 108.53 | 53.34 |
| 2 | BPH | MOI | 4 | 80.44 | 108.72 | 53.97 |
| 2 | BPH | MOI | 5 | 80.86 | 108.91 | 54.60 |
| 2 | BPH | MOI | 6 | 84.29 | 111.86 | 53.49 |
| 2 | BPH | MOI | 7 | 96.68 | 124.61 | 59.70 |
| 2 | BPH | MOI | 8 | 109.07 | 137.36 | 65.90 |
| 2 | BPH | MOI | 9 | 101.94 | 127.96 | 66.71 |
| 2 | BPH | MOI | 10 | 94.80 | 118.55 | 67.51 |
| 2 | CONTROL | MOI | 1 | 75.64 | 109.02 | 61.16 |
| 2 | CONTROL | MOI | 2 | 72.39 | 109.59 | 74.03 |
| 2 | CONTROL | MOI | 3 | 69.13 | 110.16 | 86.89 |
| 2 | CONTROL | MOI | 4 | 83.27 | 123.72 | 77.71 |
| 2 | CONTROL | MOI | 5 | 97.40 | 137.27 | 68.53 |
| 2 | CONTROL | MOI | 6 | 81.57 | 116.68 | 57.28 |
| 2 | CONTROL | MOI | 7 | 93.18 | 128.68 | 59.13 |
| 2 | CONTROL | MOI | 8 | 104.78 | 140.68 | 60.98 |
| 2 | CONTROL | MOI | 9 | 105.23 | 140.15 | 61.08 |
| 2 | CONTROL | MOI | 10 | 105.67 | 139.62 | 61.18 |
| 2 | WBPH | MOI | 1 | 82.73 | 107.05 | 48.93 |
| 2 | WBPH | MOI | 2 | 83.00 | 110.94 | 56.21 |
| 2 | WBPH | MOI | 3 | 83.26 | 114.82 | 63.49 |
| 2 | WBPH | MOI | 4 | 78.48 | 111.22 | 63.52 |
| 2 | WBPH | MOI | 5 | 78.48 | 111.22 | 63.52 |
| 2 | WBPH | MOI | 6 | 72.86 | 94.24 | 45.68 |
| 2 | WBPH | MOI | 7 | 93.09 | 120.94 | 74.54 |
| 2 | WBPH | MOI | 8 | 93.09 | 120.94 | 74.54 |
| 2 | WBPH | MOI | 9 | 102.00 | 123.60 | 75.30 |
| 2 | WBPH | MOI | 10 | 110.91 | 126.26 | 76.05 |
| 3 | BPH | MOI | 1 | 88.06 | 122.97 | 65.91 |
| 3 | BPH | MOI | 2 | 87.05 | 121.63 | 61.73 |
| 3 | BPH | MOI | 3 | 86.04 | 120.28 | 57.55 |
| 3 | BPH | MOI | 4 | 87.55 | 120.60 | 60.82 |
| 3 | BPH | MOI | 5 | 89.06 | 120.92 | 64.08 |
| 3 | BPH | MOI | 6 | 98.94 | 124.75 | 53.71 |
| 3 | BPH | MOI | 7 | 98.61 | 122.99 | 57.46 |
| 3 | BPH | MOI | 8 | 98.28 | 121.22 | 61.21 |
| 3 | BPH | MOI | 9 | 119.77 | 137.11 | 67.46 |
| 3 | BPH | MOI | 10 | 120.38 | 141.10 | 81.45 |
| 3 | CONTROL | MOI | 1 | 85.49 | 120.96 | 59.78 |
| 3 | CONTROL | MOI | 2 | 103.80 | 140.46 | 61.49 |
| 3 | CONTROL | MOI | 3 | 122.10 | 159.95 | 63.19 |
| 3 | CONTROL | MOI | 4 | 102.46 | 136.80 | 56.59 |
| 3 | CONTROL | MOI | 5 | 82.81 | 113.64 | 49.99 |
| 3 | CONTROL | MOI | 6 | 93.60 | 118.40 | 47.01 |
| 3 | CONTROL | MOI | 7 | 102.90 | 128.12 | 50.87 |
| 3 | CONTROL | MOI | 8 | 112.19 | 137.84 | 54.73 |
| 3 | CONTROL | MOI | 9 | 112.46 | 135.49 | 52.24 |
| 3 | CONTROL | MOI | 10 | 116.35 | 151.47 | 67.66 |
| 3 | WBPH | MOI | 1 | 92.60 | 128.05 | 62.41 |
| 3 | WBPH | MOI | 2 | 96.52 | 129.59 | 62.75 |
| 3 | WBPH | MOI | 3 | 100.43 | 131.13 | 63.08 |
| 3 | WBPH | MOI | 4 | 94.01 | 120.94 | 57.72 |
| 3 | WBPH | MOI | 5 | 87.58 | 110.75 | 52.36 |
| 3 | WBPH | MOI | 6 | 102.40 | 117.26 | 57.46 |
| 3 | WBPH | MOI | 7 | 104.96 | 121.31 | 60.46 |
| 3 | WBPH | MOI | 8 | 107.51 | 125.35 | 63.45 |
| 3 | WBPH | MOI | 9 | 116.58 | 137.85 | 74.57 |
| 3 | WBPH | MOI | 10 | 122.31 | 145.32 | 81.39 |
| 1 | BPH | Mudgo | 1 | 84.85 | 120.96 | 56.07 |
| 1 | BPH | Mudgo | 2 | 72.36 | 105.50 | 59.37 |
| 1 | BPH | Mudgo | 3 | 104.24 | 140.86 | 67.51 |
| 1 | BPH | Mudgo | 4 | 89.06 | 120.37 | 57.22 |
| 1 | BPH | Mudgo | 5 | 106.70 | 127.06 | 49.67 |
| 1 | BPH | Mudgo | 6 | 93.88 | 112.22 | 67.74 |
| 1 | BPH | Mudgo | 7 | 109.61 | 125.37 | 107.51 |
| 1 | BPH | Mudgo | 8 | 132.27 | 128.27 | 120.59 |
| 1 | BPH | Mudgo | 9 | 132.57 | 129.62 | 122.44 |
| 1 | BPH | Mudgo | 10 | 113.14 | 108.10 | 99.61 |
| 1 | CONTROL | Mudgo | 1 | 97.77 | 132.44 | 58.76 |
| 1 | CONTROL | Mudgo | 2 | 104.81 | 136.38 | 66.10 |
| 1 | CONTROL | Mudgo | 3 | 111.54 | 146.74 | 63.50 |
| 1 | CONTROL | Mudgo | 4 | 79.64 | 108.98 | 43.88 |
| 1 | CONTROL | Mudgo | 5 | 120.41 | 153.87 | 52.20 |
| 1 | CONTROL | Mudgo | 6 | 86.44 | 117.22 | 55.14 |
| 1 | CONTROL | Mudgo | 7 | 90.25 | 119.44 | 51.06 |
| 1 | CONTROL | Mudgo | 8 | 105.52 | 137.29 | 65.58 |
| 1 | CONTROL | Mudgo | 9 | 115.85 | 147.87 | 57.76 |
| 1 | CONTROL | Mudgo | 10 | 100.09 | 129.31 | 59.86 |
| 1 | WBPH | Mudgo | 1 | 109.75 | 144.66 | 66.36 |
| 1 | WBPH | Mudgo | 2 | 87.63 | 116.39 | 53.43 |
| 1 | WBPH | Mudgo | 3 | 114.89 | 148.71 | 64.76 |
| 1 | WBPH | Mudgo | 4 | 103.70 | 132.60 | 70.68 |
| 1 | WBPH | Mudgo | 5 | 105.05 | 125.71 | 55.60 |
| 1 | WBPH | Mudgo | 6 | 103.75 | 127.86 | 68.61 |
| 1 | WBPH | Mudgo | 7 | 109.19 | 125.05 | 59.82 |
| 1 | WBPH | Mudgo | 8 | 105.19 | 127.76 | 77.33 |
| 1 | WBPH | Mudgo | 9 | 104.15 | 128.35 | 73.74 |
| 1 | WBPH | Mudgo | 10 | 106.93 | 131.33 | 83.40 |
| 2 | BPH | Mudgo | 1 | 80.39 | 112.97 | 58.69 |
| 2 | BPH | Mudgo | 2 | 77.08 | 108.59 | 59.60 |
| 2 | BPH | Mudgo | 3 | 73.76 | 104.20 | 60.50 |
| 2 | BPH | Mudgo | 4 | 73.01 | 100.61 | 63.37 |
| 2 | BPH | Mudgo | 5 | 72.26 | 97.02 | 66.23 |
| 2 | BPH | Mudgo | 6 | 96.05 | 105.66 | 88.48 |
| 2 | BPH | Mudgo | 7 | 114.91 | 120.47 | 108.92 |
| 2 | BPH | Mudgo | 8 | 133.76 | 135.28 | 129.35 |
| 2 | BPH | Mudgo | 9 | 125.76 | 124.94 | 119.20 |
| 2 | BPH | Mudgo | 10 | 117.76 | 114.60 | 109.05 |
| 2 | CONTROL | Mudgo | 1 | 75.55 | 103.21 | 39.98 |
| 2 | CONTROL | Mudgo | 2 | 79.75 | 107.59 | 44.19 |
| 2 | CONTROL | Mudgo | 3 | 83.95 | 111.96 | 48.40 |
| 2 | CONTROL | Mudgo | 4 | 92.98 | 122.65 | 46.89 |
| 2 | CONTROL | Mudgo | 5 | 102.01 | 133.34 | 45.37 |
| 2 | CONTROL | Mudgo | 6 | 104.97 | 132.20 | 44.78 |
| 2 | CONTROL | Mudgo | 7 | 111.50 | 137.54 | 47.95 |
| 2 | CONTROL | Mudgo | 8 | 118.03 | 142.87 | 51.11 |
| 2 | CONTROL | Mudgo | 9 | 120.17 | 146.88 | 52.63 |
| 2 | CONTROL | Mudgo | 10 | 122.31 | 150.89 | 54.15 |
| 2 | WBPH | Mudgo | 1 | 81.91 | 114.98 | 60.00 |
| 2 | WBPH | Mudgo | 2 | 82.07 | 113.35 | 61.94 |
| 2 | WBPH | Mudgo | 3 | 82.23 | 111.72 | 63.88 |
| 2 | WBPH | Mudgo | 4 | 84.52 | 114.96 | 65.89 |
| 2 | WBPH | Mudgo | 5 | 85.77 | 115.10 | 68.29 |
| 2 | WBPH | Mudgo | 6 | 76.29 | 94.18 | 66.04 |
| 2 | WBPH | Mudgo | 7 | 90.69 | 102.69 | 72.71 |
| 2 | WBPH | Mudgo | 8 | 108.51 | 111.14 | 99.73 |
| 2 | WBPH | Mudgo | 9 | 111.54 | 111.74 | 101.39 |
| 2 | WBPH | Mudgo | 10 | 114.56 | 112.33 | 103.04 |
| 3 | BPH | Mudgo | 1 | 86.44 | 111.67 | 51.85 |
| 3 | BPH | Mudgo | 2 | 91.96 | 116.97 | 55.08 |
| 3 | BPH | Mudgo | 3 | 97.48 | 122.26 | 58.30 |
| 3 | BPH | Mudgo | 4 | 92.38 | 112.02 | 61.18 |
| 3 | BPH | Mudgo | 5 | 87.28 | 101.77 | 64.05 |
| 3 | BPH | Mudgo | 6 | 88.61 | 103.96 | 75.06 |
| 3 | BPH | Mudgo | 7 | 99.08 | 109.54 | 91.34 |
| 3 | BPH | Mudgo | 8 | 109.55 | 115.11 | 107.61 |
| 3 | BPH | Mudgo | 9 | 114.93 | 110.73 | 106.36 |
| 3 | BPH | Mudgo | 10 | 124.78 | 115.85 | 104.50 |
| 3 | CONTROL | Mudgo | 1 | 97.63 | 131.82 | 59.76 |
| 3 | CONTROL | Mudgo | 2 | 100.37 | 134.89 | 58.25 |
| 3 | CONTROL | Mudgo | 3 | 103.11 | 137.95 | 56.73 |
| 3 | CONTROL | Mudgo | 4 | 102.70 | 136.38 | 54.76 |
| 3 | CONTROL | Mudgo | 5 | 102.28 | 134.80 | 52.78 |
| 3 | CONTROL | Mudgo | 6 | 114.93 | 141.34 | 49.32 |
| 3 | CONTROL | Mudgo | 7 | 122.92 | 152.86 | 56.38 |
| 3 | CONTROL | Mudgo | 8 | 130.91 | 164.37 | 63.43 |
| 3 | CONTROL | Mudgo | 9 | 125.42 | 156.77 | 54.93 |
| 3 | CONTROL | Mudgo | 10 | 141.52 | 176.04 | 72.29 |
| 3 | WBPH | Mudgo | 1 | 93.74 | 128.58 | 60.88 |
| 3 | WBPH | Mudgo | 2 | 97.73 | 132.55 | 58.50 |
| 3 | WBPH | Mudgo | 3 | 101.71 | 136.51 | 56.11 |
| 3 | WBPH | Mudgo | 4 | 104.27 | 135.00 | 55.87 |
| 3 | WBPH | Mudgo | 5 | 106.82 | 133.48 | 55.63 |
| 3 | WBPH | Mudgo | 6 | 101.52 | 126.47 | 41.73 |
| 3 | WBPH | Mudgo | 7 | 111.14 | 135.15 | 49.75 |
| 3 | WBPH | Mudgo | 8 | 120.75 | 143.82 | 57.77 |
| 3 | WBPH | Mudgo | 9 | 121.72 | 145.24 | 56.69 |
| 3 | WBPH | Mudgo | 10 | 138.48 | 164.37 | 82.02 |
| 1 | BPH | N22 | 1 | 90.29 | 125.16 | 66.07 |
| 1 | BPH | N22 | 2 | 76.64 | 107.33 | 68.81 |
| 1 | BPH | N22 | 3 | 97.96 | 134.20 | 79.23 |
| 1 | BPH | N22 | 4 | 93.93 | 119.25 | 71.97 |
| 1 | BPH | N22 | 5 | 94.68 | 113.74 | 60.33 |
| 1 | BPH | N22 | 6 | 91.79 | 108.71 | 75.10 |
| 1 | BPH | N22 | 7 | 102.25 | 120.90 | 87.45 |
| 1 | BPH | N22 | 8 | 113.33 | 125.62 | 99.39 |
| 1 | BPH | N22 | 9 | 109.74 | 108.15 | 78.53 |
| 1 | BPH | N22 | 10 | 90.10 | 91.79 | 82.17 |
| 1 | CONTROL | N22 | 1 | 103.63 | 143.10 | 71.92 |
| 1 | CONTROL | N22 | 2 | 101.53 | 135.92 | 64.59 |
| 1 | CONTROL | N22 | 3 | 90.51 | 117.09 | 51.22 |
| 1 | CONTROL | N22 | 4 | 92.70 | 125.58 | 62.70 |
| 1 | CONTROL | N22 | 5 | 99.66 | 132.96 | 61.77 |
| 1 | CONTROL | N22 | 6 | 101.33 | 132.04 | 72.72 |
| 1 | CONTROL | N22 | 7 | 109.11 | 132.49 | 48.38 |
| 1 | CONTROL | N22 | 8 | 123.63 | 140.44 | 60.95 |
| 1 | CONTROL | N22 | 9 | 126.85 | 145.55 | 66.71 |
| 1 | CONTROL | N22 | 10 | 106.79 | 134.16 | 60.35 |
| 1 | WBPH | N22 | 1 | 114.18 | 151.96 | 81.59 |
| 1 | WBPH | N22 | 2 | 91.37 | 121.93 | 71.74 |
| 1 | WBPH | N22 | 3 | 107.73 | 132.18 | 70.30 |
| 1 | WBPH | N22 | 4 | 112.95 | 140.75 | 82.88 |
| 1 | WBPH | N22 | 5 | 111.78 | 136.93 | 80.38 |
| 1 | WBPH | N22 | 6 | 100.37 | 127.69 | 81.85 |
| 1 | WBPH | N22 | 7 | 98.87 | 118.39 | 76.19 |
| 1 | WBPH | N22 | 8 | 106.03 | 132.35 | 90.36 |
| 1 | WBPH | N22 | 9 | 95.53 | 119.61 | 77.66 |
| 1 | WBPH | N22 | 10 | 93.73 | 119.53 | 87.65 |
| 2 | BPH | N22 | 1 | 62.55 | 73.96 | 50.68 |
| 2 | BPH | N22 | 2 | 67.72 | 73.86 | 58.84 |
| 2 | BPH | N22 | 3 | 72.88 | 73.76 | 67.00 |
| 2 | BPH | N22 | 4 | 83.69 | 80.21 | 74.72 |
| 2 | BPH | N22 | 5 | 94.49 | 86.65 | 82.44 |
| 2 | BPH | N22 | 6 | 112.54 | 101.60 | 91.82 |
| 2 | BPH | N22 | 7 | 112.68 | 102.81 | 94.17 |
| 2 | BPH | N22 | 8 | 112.81 | 104.02 | 96.52 |
| 2 | BPH | N22 | 9 | 121.10 | 112.18 | 105.65 |
| 2 | BPH | N22 | 10 | 129.39 | 120.34 | 114.77 |
| 2 | CONTROL | N22 | 1 | 86.89 | 121.82 | 59.59 |
| 2 | CONTROL | N22 | 2 | 86.60 | 123.85 | 68.43 |
| 2 | CONTROL | N22 | 3 | 86.30 | 125.87 | 77.26 |
| 2 | CONTROL | N22 | 4 | 86.76 | 123.68 | 65.66 |
| 2 | CONTROL | N22 | 5 | 87.21 | 121.49 | 54.05 |
| 2 | CONTROL | N22 | 6 | 92.29 | 126.76 | 53.16 |
| 2 | CONTROL | N22 | 7 | 88.17 | 121.58 | 48.57 |
| 2 | CONTROL | N22 | 8 | 84.04 | 116.39 | 43.98 |
| 2 | CONTROL | N22 | 9 | 89.85 | 122.75 | 46.06 |
| 2 | CONTROL | N22 | 10 | 95.65 | 129.10 | 48.13 |
| 2 | WBPH | N22 | 1 | 56.92 | 86.93 | 48.64 |
| 2 | WBPH | N22 | 2 | 61.77 | 89.51 | 50.42 |
| 2 | WBPH | N22 | 3 | 66.62 | 92.08 | 52.19 |
| 2 | WBPH | N22 | 4 | 75.23 | 102.44 | 61.53 |
| 2 | WBPH | N22 | 5 | 68.22 | 93.15 | 59.17 |
| 2 | WBPH | N22 | 6 | 76.55 | 97.30 | 60.31 |
| 2 | WBPH | N22 | 7 | 93.20 | 107.24 | 78.12 |
| 2 | WBPH | N22 | 8 | 110.10 | 120.30 | 90.19 |
| 2 | WBPH | N22 | 9 | 104.86 | 113.49 | 86.24 |
| 2 | WBPH | N22 | 10 | 99.62 | 106.67 | 82.28 |
| 3 | BPH | N22 | 1 | 92.12 | 114.38 | 65.14 |
| 3 | BPH | N22 | 2 | 93.72 | 109.19 | 75.86 |
| 3 | BPH | N22 | 3 | 95.32 | 103.99 | 86.57 |
| 3 | BPH | N22 | 4 | 114.19 | 113.73 | 99.50 |
| 3 | BPH | N22 | 5 | 133.05 | 123.46 | 112.42 |
| 3 | BPH | N22 | 6 | 116.49 | 106.40 | 96.53 |
| 3 | BPH | N22 | 7 | 121.57 | 112.04 | 102.67 |
| 3 | BPH | N22 | 8 | 126.65 | 117.67 | 108.81 |
| 3 | BPH | N22 | 9 | 109.80 | 101.97 | 93.73 |
| 3 | BPH | N22 | 10 | 126.04 | 119.79 | 113.54 |
| 3 | CONTROL | N22 | 1 | 85.87 | 121.46 | 64.49 |
| 3 | CONTROL | N22 | 2 | 103.32 | 141.06 | 71.12 |
| 3 | CONTROL | N22 | 3 | 120.76 | 160.65 | 77.75 |
| 3 | CONTROL | N22 | 4 | 107.01 | 143.54 | 68.19 |
| 3 | CONTROL | N22 | 5 | 93.26 | 126.43 | 58.63 |
| 3 | CONTROL | N22 | 6 | 92.11 | 125.17 | 48.32 |
| 3 | CONTROL | N22 | 7 | 103.04 | 137.13 | 57.34 |
| 3 | CONTROL | N22 | 8 | 113.97 | 149.08 | 66.35 |
| 3 | CONTROL | N22 | 9 | 123.96 | 158.31 | 65.87 |
| 3 | CONTROL | N22 | 10 | 120.53 | 155.95 | 64.65 |
| 3 | WBPH | N22 | 1 | 81.77 | 109.31 | 54.22 |
| 3 | WBPH | N22 | 2 | 79.93 | 110.88 | 53.36 |
| 3 | WBPH | N22 | 3 | 78.09 | 112.44 | 52.50 |
| 3 | WBPH | N22 | 4 | 79.83 | 111.66 | 51.50 |
| 3 | WBPH | N22 | 5 | 81.57 | 110.87 | 50.50 |
| 3 | WBPH | N22 | 6 | 87.88 | 117.16 | 53.62 |
| 3 | WBPH | N22 | 7 | 92.55 | 119.55 | 55.58 |
| 3 | WBPH | N22 | 8 | 97.22 | 121.93 | 57.53 |
| 3 | WBPH | N22 | 9 | 96.02 | 123.52 | 67.07 |
| 3 | WBPH | N22 | 10 | 114.72 | 145.30 | 77.16 |
| 1 | BPH | N'DiangMarie | 1 | 122.51 | 159.97 | 87.88 |
| 1 | BPH | N'DiangMarie | 2 | 91.48 | 124.50 | 73.82 |
| 1 | BPH | N'DiangMarie | 3 | 99.85 | 131.17 | 70.65 |
| 1 | BPH | N'DiangMarie | 4 | 101.28 | 127.94 | 63.51 |
| 1 | BPH | N'DiangMarie | 5 | 113.23 | 137.99 | 71.48 |
| 1 | BPH | N'DiangMarie | 6 | 92.56 | 123.04 | 80.83 |
| 1 | BPH | N'DiangMarie | 7 | 103.94 | 119.71 | 77.56 |
| 1 | BPH | N'DiangMarie | 8 | 113.77 | 119.31 | 95.87 |
| 1 | BPH | N'DiangMarie | 9 | 135.63 | 139.78 | 115.97 |
| 1 | BPH | N'DiangMarie | 10 | 104.88 | 103.14 | 100.47 |
| 1 | CONTROL | N'DiangMarie | 1 | 88.04 | 122.52 | 55.66 |
| 1 | CONTROL | N'DiangMarie | 2 | 99.02 | 131.45 | 70.24 |
| 1 | CONTROL | N'DiangMarie | 3 | 108.46 | 142.02 | 60.95 |
| 1 | CONTROL | N'DiangMarie | 4 | 82.84 | 113.56 | 52.90 |
| 1 | CONTROL | N'DiangMarie | 5 | 102.68 | 130.50 | 48.75 |
| 1 | CONTROL | N'DiangMarie | 6 | 82.63 | 111.30 | 43.05 |
| 1 | CONTROL | N'DiangMarie | 7 | 96.02 | 125.40 | 42.91 |
| 1 | CONTROL | N'DiangMarie | 8 | 100.75 | 132.98 | 60.82 |
| 1 | CONTROL | N'DiangMarie | 9 | 110.54 | 139.56 | 60.70 |
| 1 | CONTROL | N'DiangMarie | 10 | 107.62 | 130.32 | 41.96 |
| 1 | WBPH | N'DiangMarie | 1 | 113.97 | 150.97 | 81.29 |
| 1 | WBPH | N'DiangMarie | 2 | 72.44 | 106.18 | 61.36 |
| 1 | WBPH | N'DiangMarie | 3 | 110.86 | 145.71 | 79.98 |
| 1 | WBPH | N'DiangMarie | 4 | 91.37 | 123.92 | 71.47 |
| 1 | WBPH | N'DiangMarie | 5 | 87.28 | 117.56 | 64.35 |
| 1 | WBPH | N'DiangMarie | 6 | 100.97 | 125.81 | 73.42 |
| 1 | WBPH | N'DiangMarie | 7 | 102.15 | 123.55 | 62.17 |
| 1 | WBPH | N'DiangMarie | 8 | 112.93 | 132.49 | 79.38 |
| 1 | WBPH | N'DiangMarie | 9 | 122.76 | 147.11 | 89.11 |
| 1 | WBPH | N'DiangMarie | 10 | 102.05 | 129.24 | 89.23 |
| 2 | BPH | N'DiangMarie | 1 | 72.12 | 94.22 | 48.71 |
| 2 | BPH | N'DiangMarie | 2 | 70.27 | 95.99 | 56.57 |
| 2 | BPH | N'DiangMarie | 3 | 68.41 | 97.75 | 64.42 |
| 2 | BPH | N'DiangMarie | 4 | 72.99 | 94.71 | 69.85 |
| 2 | BPH | N'DiangMarie | 5 | 77.56 | 91.67 | 75.28 |
| 2 | BPH | N'DiangMarie | 6 | 92.75 | 98.00 | 93.06 |
| 2 | BPH | N'DiangMarie | 7 | 116.90 | 115.81 | 108.77 |
| 2 | BPH | N'DiangMarie | 8 | 141.05 | 133.61 | 124.48 |
| 2 | BPH | N'DiangMarie | 9 | 130.88 | 124.90 | 118.41 |
| 2 | BPH | N'DiangMarie | 10 | 120.70 | 116.18 | 112.34 |
| 2 | CONTROL | N'DiangMarie | 1 | 87.59 | 114.75 | 53.40 |
| 2 | CONTROL | N'DiangMarie | 2 | 86.33 | 119.38 | 61.74 |
| 2 | CONTROL | N'DiangMarie | 3 | 85.06 | 124.00 | 70.07 |
| 2 | CONTROL | N'DiangMarie | 4 | 85.41 | 121.43 | 58.21 |
| 2 | CONTROL | N'DiangMarie | 5 | 85.76 | 118.86 | 46.34 |
| 2 | CONTROL | N'DiangMarie | 6 | 91.37 | 121.79 | 43.76 |
| 2 | CONTROL | N'DiangMarie | 7 | 99.89 | 126.58 | 43.24 |
| 2 | CONTROL | N'DiangMarie | 8 | 108.41 | 131.37 | 42.72 |
| 2 | CONTROL | N'DiangMarie | 9 | 103.69 | 129.85 | 45.36 |
| 2 | CONTROL | N'DiangMarie | 10 | 98.96 | 128.33 | 47.99 |
| 2 | WBPH | N'DiangMarie | 1 | 86.86 | 108.66 | 46.66 |
| 2 | WBPH | N'DiangMarie | 2 | 82.01 | 107.34 | 48.45 |
| 2 | WBPH | N'DiangMarie | 3 | 77.15 | 106.02 | 50.24 |
| 2 | WBPH | N'DiangMarie | 4 | 77.54 | 105.90 | 59.05 |
| 2 | WBPH | N'DiangMarie | 5 | 88.46 | 119.71 | 65.91 |
| 2 | WBPH | N'DiangMarie | 6 | 86.00 | 108.03 | 55.95 |
| 2 | WBPH | N'DiangMarie | 7 | 95.10 | 118.47 | 67.61 |
| 2 | WBPH | N'DiangMarie | 8 | 113.65 | 139.64 | 74.90 |
| 2 | WBPH | N'DiangMarie | 9 | 106.55 | 133.25 | 75.05 |
| 2 | WBPH | N'DiangMarie | 10 | 99.45 | 126.86 | 75.20 |
| 3 | BPH | N'DiangMarie | 1 | 93.90 | 128.43 | 67.18 |
| 3 | BPH | N'DiangMarie | 2 | 87.05 | 119.58 | 68.51 |
| 3 | BPH | N'DiangMarie | 3 | 80.19 | 110.73 | 69.83 |
| 3 | BPH | N'DiangMarie | 4 | 83.53 | 105.29 | 78.07 |
| 3 | BPH | N'DiangMarie | 5 | 86.86 | 99.85 | 86.31 |
| 3 | BPH | N'DiangMarie | 6 | 109.16 | 113.48 | 103.67 |
| 3 | BPH | N'DiangMarie | 7 | 115.04 | 115.35 | 106.30 |
| 3 | BPH | N'DiangMarie | 8 | 120.92 | 117.22 | 108.93 |
| 3 | BPH | N'DiangMarie | 9 | 135.31 | 127.74 | 119.98 |
| 3 | BPH | N'DiangMarie | 10 | 127.39 | 123.32 | 117.80 |
| 3 | CONTROL | N'DiangMarie | 1 | 92.77 | 126.36 | 66.33 |
| 3 | CONTROL | N'DiangMarie | 2 | 100.57 | 135.47 | 66.55 |
| 3 | CONTROL | N'DiangMarie | 3 | 108.37 | 144.58 | 66.76 |
| 3 | CONTROL | N'DiangMarie | 4 | 102.11 | 136.21 | 60.66 |
| 3 | CONTROL | N'DiangMarie | 5 | 95.84 | 127.83 | 54.56 |
| 3 | CONTROL | N'DiangMarie | 6 | 77.68 | 108.32 | 52.74 |
| 3 | CONTROL | N'DiangMarie | 7 | 106.07 | 138.23 | 61.18 |
| 3 | CONTROL | N'DiangMarie | 8 | 134.45 | 168.13 | 69.61 |
| 3 | CONTROL | N'DiangMarie | 9 | 120.52 | 152.48 | 64.50 |
| 3 | CONTROL | N'DiangMarie | 10 | 141.29 | 169.49 | 73.05 |
| 3 | WBPH | N'DiangMarie | 1 | 84.51 | 115.43 | 63.63 |
| 3 | WBPH | N'DiangMarie | 2 | 82.94 | 113.49 | 61.89 |
| 3 | WBPH | N'DiangMarie | 3 | 81.36 | 111.55 | 60.14 |
| 3 | WBPH | N'DiangMarie | 4 | 78.59 | 106.93 | 60.01 |
| 3 | WBPH | N'DiangMarie | 5 | 75.82 | 102.31 | 59.88 |
| 3 | WBPH | N'DiangMarie | 6 | 95.18 | 119.41 | 62.65 |
| 3 | WBPH | N'DiangMarie | 7 | 100.38 | 122.94 | 68.65 |
| 3 | WBPH | N'DiangMarie | 8 | 105.58 | 126.47 | 74.64 |
| 3 | WBPH | N'DiangMarie | 9 | 85.93 | 103.40 | 67.61 |
| 3 | WBPH | N'DiangMarie | 10 | 120.28 | 139.05 | 91.65 |
| 1 | BPH | Pokkali | 1 | 99.00 | 138.55 | 77.83 |
| 1 | BPH | Pokkali | 2 | 88.61 | 120.41 | 69.71 |
| 1 | BPH | Pokkali | 3 | 100.46 | 135.77 | 65.59 |
| 1 | BPH | Pokkali | 4 | 87.68 | 121.07 | 65.89 |
| 1 | BPH | Pokkali | 5 | 99.12 | 131.21 | 71.30 |
| 1 | BPH | Pokkali | 6 | 103.77 | 129.46 | 75.58 |
| 1 | BPH | Pokkali | 7 | 111.40 | 142.59 | 83.56 |
| 1 | BPH | Pokkali | 8 | 111.42 | 139.04 | 91.69 |
| 1 | BPH | Pokkali | 9 | 113.16 | 134.98 | 90.63 |
| 1 | BPH | Pokkali | 10 | 89.03 | 107.71 | 96.19 |
| 1 | CONTROL | Pokkali | 1 | 81.50 | 115.67 | 55.14 |
| 1 | CONTROL | Pokkali | 2 | 94.21 | 131.42 | 62.81 |
| 1 | CONTROL | Pokkali | 3 | 107.99 | 144.44 | 64.95 |
| 1 | CONTROL | Pokkali | 4 | 87.43 | 112.57 | 52.13 |
| 1 | CONTROL | Pokkali | 5 | 81.33 | 112.87 | 47.64 |
| 1 | CONTROL | Pokkali | 6 | 82.79 | 113.42 | 50.60 |
| 1 | CONTROL | Pokkali | 7 | 82.19 | 111.46 | 55.46 |
| 1 | CONTROL | Pokkali | 8 | 110.30 | 143.06 | 59.59 |
| 1 | CONTROL | Pokkali | 9 | 95.65 | 125.50 | 58.57 |
| 1 | CONTROL | Pokkali | 10 | 97.27 | 118.07 | 52.16 |
| 1 | WBPH | Pokkali | 1 | 114.21 | 143.69 | 70.90 |
| 1 | WBPH | Pokkali | 2 | 93.73 | 118.24 | 63.23 |
| 1 | WBPH | Pokkali | 3 | 105.07 | 137.89 | 72.61 |
| 1 | WBPH | Pokkali | 4 | 102.45 | 127.23 | 58.10 |
| 1 | WBPH | Pokkali | 5 | 98.79 | 125.02 | 68.11 |
| 1 | WBPH | Pokkali | 6 | 108.84 | 134.48 | 79.55 |
| 1 | WBPH | Pokkali | 7 | 116.14 | 132.21 | 64.52 |
| 1 | WBPH | Pokkali | 8 | 137.22 | 155.64 | 88.33 |
| 1 | WBPH | Pokkali | 9 | 137.13 | 151.75 | 77.72 |
| 1 | WBPH | Pokkali | 10 | 125.17 | 140.77 | 88.04 |
| 2 | BPH | Pokkali | 1 | 78.66 | 113.30 | 64.03 |
| 2 | BPH | Pokkali | 2 | 77.31 | 112.39 | 65.39 |
| 2 | BPH | Pokkali | 3 | 75.96 | 111.47 | 66.74 |
| 2 | BPH | Pokkali | 4 | 73.81 | 107.54 | 65.07 |
| 2 | BPH | Pokkali | 5 | 71.65 | 103.61 | 63.39 |
| 2 | BPH | Pokkali | 6 | 77.78 | 101.54 | 77.25 |
| 2 | BPH | Pokkali | 7 | 96.83 | 114.16 | 98.80 |
| 2 | BPH | Pokkali | 8 | 115.87 | 126.78 | 120.34 |
| 2 | BPH | Pokkali | 9 | 113.29 | 119.79 | 115.24 |
| 2 | BPH | Pokkali | 10 | 110.70 | 112.79 | 110.13 |
| 2 | CONTROL | Pokkali | 1 | 85.30 | 111.68 | 50.74 |
| 2 | CONTROL | Pokkali | 2 | 80.31 | 112.51 | 60.46 |
| 2 | CONTROL | Pokkali | 3 | 75.32 | 113.34 | 70.17 |
| 2 | CONTROL | Pokkali | 4 | 82.28 | 118.15 | 61.94 |
| 2 | CONTROL | Pokkali | 5 | 89.23 | 122.95 | 53.70 |
| 2 | CONTROL | Pokkali | 6 | 105.22 | 127.14 | 49.11 |
| 2 | CONTROL | Pokkali | 7 | 114.83 | 142.54 | 60.80 |
| 2 | CONTROL | Pokkali | 8 | 124.43 | 157.93 | 72.49 |
| 2 | CONTROL | Pokkali | 9 | 125.48 | 153.51 | 70.81 |
| 2 | CONTROL | Pokkali | 10 | 126.53 | 149.08 | 69.13 |
| 2 | WBPH | Pokkali | 1 | 77.44 | 109.18 | 58.29 |
| 2 | WBPH | Pokkali | 2 | 75.26 | 107.83 | 57.13 |
| 2 | WBPH | Pokkali | 3 | 73.07 | 106.48 | 55.97 |
| 2 | WBPH | Pokkali | 4 | 85.29 | 111.07 | 50.69 |
| 2 | WBPH | Pokkali | 5 | 93.43 | 116.11 | 51.13 |
| 2 | WBPH | Pokkali | 6 | 89.54 | 116.80 | 63.94 |
| 2 | WBPH | Pokkali | 7 | 89.27 | 115.24 | 63.86 |
| 2 | WBPH | Pokkali | 8 | 92.53 | 122.44 | 71.76 |
| 2 | WBPH | Pokkali | 9 | 90.44 | 116.84 | 72.71 |
| 2 | WBPH | Pokkali | 10 | 88.35 | 111.24 | 73.65 |
| 3 | BPH | Pokkali | 1 | 85.12 | 119.89 | 70.06 |
| 3 | BPH | Pokkali | 2 | 88.27 | 123.94 | 72.62 |
| 3 | BPH | Pokkali | 3 | 91.41 | 127.99 | 75.18 |
| 3 | BPH | Pokkali | 4 | 97.40 | 132.62 | 73.65 |
| 3 | BPH | Pokkali | 5 | 103.39 | 137.25 | 72.12 |
| 3 | BPH | Pokkali | 6 | 94.29 | 120.30 | 58.90 |
| 3 | BPH | Pokkali | 7 | 91.34 | 118.70 | 62.22 |
| 3 | BPH | Pokkali | 8 | 88.39 | 117.09 | 65.53 |
| 3 | BPH | Pokkali | 9 | 89.38 | 115.78 | 77.05 |
| 3 | BPH | Pokkali | 10 | 112.44 | 140.37 | 96.89 |
| 3 | CONTROL | Pokkali | 1 | 95.32 | 129.64 | 63.74 |
| 3 | CONTROL | Pokkali | 2 | 96.97 | 131.32 | 60.83 |
| 3 | CONTROL | Pokkali | 3 | 98.62 | 132.99 | 57.91 |
| 3 | CONTROL | Pokkali | 4 | 97.45 | 127.76 | 50.19 |
| 3 | CONTROL | Pokkali | 5 | 96.28 | 122.52 | 42.46 |
| 3 | CONTROL | Pokkali | 6 | 99.05 | 130.54 | 51.35 |
| 3 | CONTROL | Pokkali | 7 | 110.33 | 137.74 | 55.86 |
| 3 | CONTROL | Pokkali | 8 | 121.61 | 144.94 | 60.37 |
| 3 | CONTROL | Pokkali | 9 | 120.84 | 148.40 | 58.33 |
| 3 | CONTROL | Pokkali | 10 | 124.38 | 154.01 | 69.25 |
| 3 | WBPH | Pokkali | 1 | 97.82 | 134.09 | 72.49 |
| 3 | WBPH | Pokkali | 2 | 98.67 | 132.28 | 67.44 |
| 3 | WBPH | Pokkali | 3 | 99.51 | 130.46 | 62.39 |
| 3 | WBPH | Pokkali | 4 | 99.56 | 126.40 | 61.05 |
| 3 | WBPH | Pokkali | 5 | 99.61 | 122.34 | 59.70 |
| 3 | WBPH | Pokkali | 6 | 109.54 | 126.31 | 64.32 |
| 3 | WBPH | Pokkali | 7 | 119.03 | 136.95 | 72.80 |
| 3 | WBPH | Pokkali | 8 | 128.51 | 147.59 | 81.28 |
| 3 | WBPH | Pokkali | 9 | 109.41 | 130.21 | 68.02 |
| 3 | WBPH | Pokkali | 10 | 137.13 | 162.45 | 91.01 |
| 1 | BPH | PTB33 | 1 | 110.46 | 141.64 | 69.51 |
| 1 | BPH | PTB33 | 2 | 70.61 | 104.74 | 56.08 |
| 1 | BPH | PTB33 | 3 | 96.39 | 129.48 | 60.96 |
| 1 | BPH | PTB33 | 4 | 89.96 | 118.36 | 52.49 |
| 1 | BPH | PTB33 | 5 | 81.02 | 109.11 | 59.05 |
| 1 | BPH | PTB33 | 6 | 108.72 | 138.66 | 81.32 |
| 1 | BPH | PTB33 | 7 | 105.00 | 123.71 | 63.84 |
| 1 | BPH | PTB33 | 8 | 111.99 | 131.22 | 75.32 |
| 1 | BPH | PTB33 | 9 | 101.79 | 122.81 | 69.27 |
| 1 | BPH | PTB33 | 10 | 113.87 | 130.25 | 72.32 |
| 1 | CONTROL | PTB33 | 1 | 106.64 | 140.66 | 63.98 |
| 1 | CONTROL | PTB33 | 2 | 93.05 | 124.93 | 65.24 |
| 1 | CONTROL | PTB33 | 3 | 99.12 | 126.50 | 59.59 |
| 1 | CONTROL | PTB33 | 4 | 101.41 | 128.15 | 59.07 |
| 1 | CONTROL | PTB33 | 5 | 93.58 | 125.07 | 63.54 |
| 1 | CONTROL | PTB33 | 6 | 107.47 | 131.90 | 65.65 |
| 1 | CONTROL | PTB33 | 7 | 118.13 | 143.70 | 63.14 |
| 1 | CONTROL | PTB33 | 8 | 114.63 | 139.29 | 67.13 |
| 1 | CONTROL | PTB33 | 9 | 117.91 | 144.23 | 66.79 |
| 1 | CONTROL | PTB33 | 10 | 110.80 | 127.51 | 63.01 |
| 1 | WBPH | PTB33 | 1 | 106.26 | 135.16 | 68.25 |
| 1 | WBPH | PTB33 | 2 | 77.06 | 105.77 | 52.84 |
| 1 | WBPH | PTB33 | 3 | 114.67 | 127.63 | 65.13 |
| 1 | WBPH | PTB33 | 4 | 143.86 | 148.62 | 93.60 |
| 1 | WBPH | PTB33 | 5 | 117.48 | 120.49 | 80.06 |
| 1 | WBPH | PTB33 | 6 | 115.95 | 119.02 | 78.39 |
| 1 | WBPH | PTB33 | 7 | 113.76 | 117.22 | 79.99 |
| 1 | WBPH | PTB33 | 8 | 123.28 | 126.56 | 91.81 |
| 1 | WBPH | PTB33 | 9 | 118.66 | 122.24 | 81.44 |
| 1 | WBPH | PTB33 | 10 | 97.51 | 107.45 | 88.07 |
| 2 | BPH | PTB33 | 1 | 76.07 | 105.07 | 61.76 |
| 2 | BPH | PTB33 | 2 | 74.39 | 102.88 | 60.01 |
| 2 | BPH | PTB33 | 3 | 72.71 | 100.69 | 58.26 |
| 2 | BPH | PTB33 | 4 | 72.05 | 100.24 | 58.74 |
| 2 | BPH | PTB33 | 5 | 71.38 | 99.79 | 59.21 |
| 2 | BPH | PTB33 | 6 | 70.11 | 97.42 | 59.79 |
| 2 | BPH | PTB33 | 7 | 84.98 | 111.92 | 68.04 |
| 2 | BPH | PTB33 | 8 | 99.85 | 126.42 | 76.28 |
| 2 | BPH | PTB33 | 9 | 90.76 | 117.92 | 77.38 |
| 2 | BPH | PTB33 | 10 | 81.66 | 109.42 | 78.47 |
| 2 | CONTROL | PTB33 | 1 | 62.00 | 95.31 | 55.67 |
| 2 | CONTROL | PTB33 | 2 | 61.97 | 98.84 | 68.58 |
| 2 | CONTROL | PTB33 | 3 | 61.94 | 102.37 | 81.48 |
| 2 | CONTROL | PTB33 | 4 | 63.45 | 101.36 | 68.73 |
| 2 | CONTROL | PTB33 | 5 | 64.96 | 100.34 | 55.97 |
| 2 | CONTROL | PTB33 | 6 | 76.89 | 104.67 | 54.31 |
| 2 | CONTROL | PTB33 | 7 | 75.90 | 108.26 | 58.33 |
| 2 | CONTROL | PTB33 | 8 | 74.90 | 111.84 | 62.35 |
| 2 | CONTROL | PTB33 | 9 | 73.34 | 106.47 | 57.97 |
| 2 | CONTROL | PTB33 | 10 | 71.78 | 101.10 | 53.58 |
| 2 | WBPH | PTB33 | 1 | 81.63 | 112.98 | 51.57 |
| 2 | WBPH | PTB33 | 2 | 79.68 | 109.70 | 50.50 |
| 2 | WBPH | PTB33 | 3 | 77.73 | 106.41 | 49.42 |
| 2 | WBPH | PTB33 | 4 | 73.56 | 100.72 | 52.81 |
| 2 | WBPH | PTB33 | 5 | 74.05 | 94.96 | 49.64 |
| 2 | WBPH | PTB33 | 6 | 71.39 | 78.95 | 56.45 |
| 2 | WBPH | PTB33 | 7 | 106.27 | 116.76 | 83.98 |
| 2 | WBPH | PTB33 | 8 | 123.00 | 116.72 | 104.01 |
| 2 | WBPH | PTB33 | 9 | 115.82 | 108.42 | 96.99 |
| 2 | WBPH | PTB33 | 10 | 108.64 | 100.12 | 89.96 |
| 3 | BPH | PTB33 | 1 | 102.34 | 132.87 | 63.47 |
| 3 | BPH | PTB33 | 2 | 94.96 | 127.36 | 60.15 |
| 3 | BPH | PTB33 | 3 | 87.58 | 121.85 | 56.82 |
| 3 | BPH | PTB33 | 4 | 90.81 | 122.00 | 56.52 |
| 3 | BPH | PTB33 | 5 | 94.03 | 122.15 | 56.21 |
| 3 | BPH | PTB33 | 6 | 82.32 | 109.57 | 52.17 |
| 3 | BPH | PTB33 | 7 | 96.54 | 122.38 | 60.86 |
| 3 | BPH | PTB33 | 8 | 110.75 | 135.19 | 69.55 |
| 3 | BPH | PTB33 | 9 | 98.00 | 118.19 | 71.43 |
| 3 | BPH | PTB33 | 10 | 116.55 | 140.38 | 82.51 |
| 3 | CONTROL | PTB33 | 1 | 92.81 | 128.97 | 64.87 |
| 3 | CONTROL | PTB33 | 2 | 90.00 | 125.60 | 60.12 |
| 3 | CONTROL | PTB33 | 3 | 87.18 | 122.23 | 55.36 |
| 3 | CONTROL | PTB33 | 4 | 95.96 | 131.08 | 58.54 |
| 3 | CONTROL | PTB33 | 5 | 104.73 | 139.92 | 61.72 |
| 3 | CONTROL | PTB33 | 6 | 91.32 | 124.23 | 52.84 |
| 3 | CONTROL | PTB33 | 7 | 102.54 | 136.45 | 60.06 |
| 3 | CONTROL | PTB33 | 8 | 113.75 | 148.66 | 67.28 |
| 3 | CONTROL | PTB33 | 9 | 114.23 | 148.81 | 70.06 |
| 3 | CONTROL | PTB33 | 10 | 119.14 | 154.84 | 73.49 |
| 3 | WBPH | PTB33 | 1 | 86.39 | 118.72 | 59.15 |
| 3 | WBPH | PTB33 | 2 | 84.37 | 115.76 | 57.23 |
| 3 | WBPH | PTB33 | 3 | 82.34 | 112.79 | 55.30 |
| 3 | WBPH | PTB33 | 4 | 87.08 | 111.38 | 55.94 |
| 3 | WBPH | PTB33 | 5 | 91.82 | 109.96 | 56.58 |
| 3 | WBPH | PTB33 | 6 | 106.09 | 117.22 | 59.34 |
| 3 | WBPH | PTB33 | 7 | 112.87 | 122.40 | 66.18 |
| 3 | WBPH | PTB33 | 8 | 119.65 | 127.57 | 73.02 |
| 3 | WBPH | PTB33 | 9 | 114.54 | 121.50 | 73.16 |
| 3 | WBPH | PTB33 | 10 | 132.45 | 143.45 | 94.45 |
| 1 | BPH | RathuHeenati | 1 | 106.02 | 141.75 | 76.03 |
| 1 | BPH | RathuHeenati | 2 | 89.31 | 124.89 | 80.29 |
| 1 | BPH | RathuHeenati | 3 | 97.71 | 131.27 | 71.60 |
| 1 | BPH | RathuHeenati | 4 | 91.66 | 122.52 | 65.76 |
| 1 | BPH | RathuHeenati | 5 | 97.35 | 123.90 | 55.79 |
| 1 | BPH | RathuHeenati | 6 | 102.52 | 127.97 | 62.21 |
| 1 | BPH | RathuHeenati | 7 | 99.44 | 130.96 | 78.81 |
| 1 | BPH | RathuHeenati | 8 | 102.60 | 134.16 | 84.17 |
| 1 | BPH | RathuHeenati | 9 | 92.69 | 119.86 | 77.23 |
| 1 | BPH | RathuHeenati | 10 | 89.84 | 117.09 | 71.72 |
| 1 | CONTROL | RathuHeenati | 1 | 110.09 | 139.07 | 64.86 |
| 1 | CONTROL | RathuHeenati | 2 | 93.66 | 131.42 | 72.22 |
| 1 | CONTROL | RathuHeenati | 3 | 110.32 | 146.68 | 71.81 |
| 1 | CONTROL | RathuHeenati | 4 | 86.86 | 119.89 | 62.74 |
| 1 | CONTROL | RathuHeenati | 5 | 99.03 | 132.37 | 66.26 |
| 1 | CONTROL | RathuHeenati | 6 | 92.13 | 124.94 | 67.67 |
| 1 | CONTROL | RathuHeenati | 7 | 100.06 | 130.63 | 59.64 |
| 1 | CONTROL | RathuHeenati | 8 | 95.29 | 126.20 | 65.30 |
| 1 | CONTROL | RathuHeenati | 9 | 116.29 | 148.13 | 80.32 |
| 1 | CONTROL | RathuHeenati | 10 | 94.80 | 123.21 | 64.94 |
| 1 | WBPH | RathuHeenati | 1 | 96.06 | 126.97 | 62.85 |
| 1 | WBPH | RathuHeenati | 2 | 82.15 | 115.17 | 65.44 |
| 1 | WBPH | RathuHeenati | 3 | 97.80 | 129.65 | 66.90 |
| 1 | WBPH | RathuHeenati | 4 | 98.74 | 130.25 | 78.21 |
| 1 | WBPH | RathuHeenati | 5 | 97.59 | 119.31 | 57.86 |
| 1 | WBPH | RathuHeenati | 6 | 101.77 | 121.56 | 64.62 |
| 1 | WBPH | RathuHeenati | 7 | 100.85 | 119.03 | 64.23 |
| 1 | WBPH | RathuHeenati | 8 | 110.72 | 130.20 | 82.97 |
| 1 | WBPH | RathuHeenati | 9 | 107.46 | 125.57 | 74.74 |
| 1 | WBPH | RathuHeenati | 10 | 97.07 | 112.15 | 75.73 |
| 2 | BPH | RathuHeenati | 1 | 72.69 | 101.34 | 53.63 |
| 2 | BPH | RathuHeenati | 2 | 72.37 | 98.91 | 48.75 |
| 2 | BPH | RathuHeenati | 3 | 72.05 | 96.48 | 43.87 |
| 2 | BPH | RathuHeenati | 4 | 75.97 | 100.03 | 47.28 |
| 2 | BPH | RathuHeenati | 5 | 79.88 | 103.58 | 50.69 |
| 2 | BPH | RathuHeenati | 6 | 95.16 | 122.00 | 56.91 |
| 2 | BPH | RathuHeenati | 7 | 97.76 | 124.67 | 64.21 |
| 2 | BPH | RathuHeenati | 8 | 100.35 | 127.34 | 71.51 |
| 2 | BPH | RathuHeenati | 9 | 98.91 | 122.26 | 70.16 |
| 2 | BPH | RathuHeenati | 10 | 97.47 | 117.17 | 68.81 |
| 2 | CONTROL | RathuHeenati | 1 | 93.07 | 121.25 | 54.27 |
| 2 | CONTROL | RathuHeenati | 2 | 90.32 | 120.41 | 59.42 |
| 2 | CONTROL | RathuHeenati | 3 | 87.57 | 119.56 | 64.57 |
| 2 | CONTROL | RathuHeenati | 4 | 83.49 | 115.68 | 57.63 |
| 2 | CONTROL | RathuHeenati | 5 | 79.41 | 111.80 | 50.69 |
| 2 | CONTROL | RathuHeenati | 6 | 88.02 | 121.57 | 52.92 |
| 2 | CONTROL | RathuHeenati | 7 | 103.51 | 132.43 | 53.15 |
| 2 | CONTROL | RathuHeenati | 8 | 119.00 | 143.29 | 53.37 |
| 2 | CONTROL | RathuHeenati | 9 | 113.08 | 135.38 | 48.76 |
| 2 | CONTROL | RathuHeenati | 10 | 107.16 | 127.47 | 44.15 |
| 2 | WBPH | RathuHeenati | 1 | 68.60 | 100.25 | 52.73 |
| 2 | WBPH | RathuHeenati | 2 | 69.02 | 99.48 | 52.07 |
| 2 | WBPH | RathuHeenati | 3 | 69.43 | 98.70 | 51.41 |
| 2 | WBPH | RathuHeenati | 4 | 80.64 | 110.31 | 55.88 |
| 2 | WBPH | RathuHeenati | 5 | 83.55 | 114.20 | 62.33 |
| 2 | WBPH | RathuHeenati | 6 | 82.56 | 100.92 | 46.32 |
| 2 | WBPH | RathuHeenati | 7 | 86.70 | 102.76 | 66.06 |
| 2 | WBPH | RathuHeenati | 8 | 102.00 | 126.56 | 75.67 |
| 2 | WBPH | RathuHeenati | 9 | 99.89 | 121.55 | 75.13 |
| 2 | WBPH | RathuHeenati | 10 | 97.78 | 116.54 | 74.59 |
| 3 | BPH | RathuHeenati | 1 | 103.81 | 138.00 | 65.20 |
| 3 | BPH | RathuHeenati | 2 | 105.34 | 138.65 | 65.86 |
| 3 | BPH | RathuHeenati | 3 | 106.87 | 139.29 | 66.51 |
| 3 | BPH | RathuHeenati | 4 | 103.89 | 134.36 | 63.26 |
| 3 | BPH | RathuHeenati | 5 | 100.91 | 129.43 | 60.00 |
| 3 | BPH | RathuHeenati | 6 | 84.90 | 116.52 | 54.44 |
| 3 | BPH | RathuHeenati | 7 | 91.30 | 118.98 | 56.72 |
| 3 | BPH | RathuHeenati | 8 | 97.69 | 121.43 | 58.99 |
| 3 | BPH | RathuHeenati | 9 | 91.63 | 123.06 | 60.67 |
| 3 | BPH | RathuHeenati | 10 | 110.38 | 141.02 | 76.58 |
| 3 | CONTROL | RathuHeenati | 1 | 92.08 | 124.49 | 55.81 |
| 3 | CONTROL | RathuHeenati | 2 | 110.16 | 146.50 | 72.10 |
| 3 | CONTROL | RathuHeenati | 3 | 128.23 | 168.51 | 88.38 |
| 3 | CONTROL | RathuHeenati | 4 | 107.09 | 143.81 | 71.57 |
| 3 | CONTROL | RathuHeenati | 5 | 85.95 | 119.10 | 54.76 |
| 3 | CONTROL | RathuHeenati | 6 | 88.08 | 120.14 | 50.50 |
| 3 | CONTROL | RathuHeenati | 7 | 105.40 | 137.35 | 55.20 |
| 3 | CONTROL | RathuHeenati | 8 | 122.71 | 154.56 | 59.89 |
| 3 | CONTROL | RathuHeenati | 9 | 119.71 | 145.50 | 59.90 |
| 3 | CONTROL | RathuHeenati | 10 | 142.71 | 169.90 | 83.86 |
| 3 | WBPH | RathuHeenati | 1 | 111.06 | 140.30 | 57.67 |
| 3 | WBPH | RathuHeenati | 2 | 104.27 | 133.08 | 57.21 |
| 3 | WBPH | RathuHeenati | 3 | 97.48 | 125.86 | 56.74 |
| 3 | WBPH | RathuHeenati | 4 | 91.88 | 119.51 | 54.47 |
| 3 | WBPH | RathuHeenati | 5 | 86.27 | 113.16 | 52.19 |
| 3 | WBPH | RathuHeenati | 6 | 94.35 | 109.73 | 49.20 |
| 3 | WBPH | RathuHeenati | 7 | 105.97 | 120.19 | 58.07 |
| 3 | WBPH | RathuHeenati | 8 | 117.59 | 130.65 | 66.93 |
| 3 | WBPH | RathuHeenati | 9 | 114.16 | 124.95 | 75.70 |
| 3 | WBPH | RathuHeenati | 10 | 128.32 | 138.42 | 86.71 |
| 1 | BPH | Swarnalata | 1 | 103.05 | 141.83 | 77.66 |
| 1 | BPH | Swarnalata | 2 | 73.83 | 109.54 | 65.62 |
| 1 | BPH | Swarnalata | 3 | 95.29 | 131.43 | 74.64 |
| 1 | BPH | Swarnalata | 4 | 85.24 | 119.24 | 66.12 |
| 1 | BPH | Swarnalata | 5 | 96.67 | 125.36 | 76.68 |
| 1 | BPH | Swarnalata | 6 | 104.43 | 132.95 | 87.19 |
| 1 | BPH | Swarnalata | 7 | 106.53 | 127.01 | 77.27 |
| 1 | BPH | Swarnalata | 8 | 110.78 | 127.82 | 103.76 |
| 1 | BPH | Swarnalata | 9 | 89.07 | 103.88 | 86.94 |
| 1 | BPH | Swarnalata | 10 | 108.74 | 119.68 | 97.00 |
| 1 | CONTROL | Swarnalata | 1 | 78.64 | 115.59 | 57.64 |
| 1 | CONTROL | Swarnalata | 2 | 86.65 | 126.91 | 74.49 |
| 1 | CONTROL | Swarnalata | 3 | 95.06 | 132.21 | 63.81 |
| 1 | CONTROL | Swarnalata | 4 | 80.88 | 115.73 | 61.98 |
| 1 | CONTROL | Swarnalata | 5 | 117.62 | 139.11 | 61.44 |
| 1 | CONTROL | Swarnalata | 6 | 86.66 | 120.42 | 70.13 |
| 1 | CONTROL | Swarnalata | 7 | 107.23 | 130.13 | 58.75 |
| 1 | CONTROL | Swarnalata | 8 | 105.96 | 132.16 | 61.04 |
| 1 | CONTROL | Swarnalata | 9 | 119.07 | 139.97 | 65.29 |
| 1 | CONTROL | Swarnalata | 10 | 99.64 | 128.74 | 63.48 |
| 1 | WBPH | Swarnalata | 1 | 101.12 | 140.73 | 80.77 |
| 1 | WBPH | Swarnalata | 2 | 85.63 | 113.33 | 61.56 |
| 1 | WBPH | Swarnalata | 3 | 96.08 | 120.56 | 63.86 |
| 1 | WBPH | Swarnalata | 4 | 103.50 | 133.00 | 80.83 |
| 1 | WBPH | Swarnalata | 5 | 105.26 | 125.23 | 77.42 |
| 1 | WBPH | Swarnalata | 6 | 105.97 | 122.83 | 87.43 |
| 1 | WBPH | Swarnalata | 7 | 96.19 | 105.98 | 85.69 |
| 1 | WBPH | Swarnalata | 8 | 108.11 | 119.94 | 102.09 |
| 1 | WBPH | Swarnalata | 9 | 95.00 | 103.00 | 90.81 |
| 1 | WBPH | Swarnalata | 10 | 81.65 | 92.34 | 87.19 |
| 2 | BPH | Swarnalata | 1 | 64.38 | 93.73 | 54.62 |
| 2 | BPH | Swarnalata | 2 | 64.08 | 94.08 | 57.13 |
| 2 | BPH | Swarnalata | 3 | 63.77 | 94.42 | 59.64 |
| 2 | BPH | Swarnalata | 4 | 66.30 | 96.32 | 59.53 |
| 2 | BPH | Swarnalata | 5 | 68.82 | 98.22 | 59.42 |
| 2 | BPH | Swarnalata | 6 | 68.02 | 93.81 | 57.39 |
| 2 | BPH | Swarnalata | 7 | 71.79 | 96.05 | 63.47 |
| 2 | BPH | Swarnalata | 8 | 75.56 | 98.28 | 69.54 |
| 2 | BPH | Swarnalata | 9 | 77.49 | 96.33 | 69.96 |
| 2 | BPH | Swarnalata | 10 | 79.42 | 94.37 | 70.37 |
| 2 | CONTROL | Swarnalata | 1 | 80.26 | 107.16 | 45.61 |
| 2 | CONTROL | Swarnalata | 2 | 75.04 | 105.64 | 55.33 |
| 2 | CONTROL | Swarnalata | 3 | 69.81 | 104.11 | 65.04 |
| 2 | CONTROL | Swarnalata | 4 | 75.98 | 105.16 | 56.31 |
| 2 | CONTROL | Swarnalata | 5 | 82.15 | 106.21 | 47.58 |
| 2 | CONTROL | Swarnalata | 6 | 78.79 | 112.61 | 49.51 |
| 2 | CONTROL | Swarnalata | 7 | 83.87 | 117.77 | 52.72 |
| 2 | CONTROL | Swarnalata | 8 | 88.94 | 122.93 | 55.92 |
| 2 | CONTROL | Swarnalata | 9 | 87.67 | 115.68 | 48.92 |
| 2 | CONTROL | Swarnalata | 10 | 86.39 | 108.42 | 41.92 |
| 2 | WBPH | Swarnalata | 1 | 58.05 | 88.45 | 60.25 |
| 2 | WBPH | Swarnalata | 2 | 57.83 | 86.24 | 58.83 |
| 2 | WBPH | Swarnalata | 3 | 57.60 | 84.02 | 57.41 |
| 2 | WBPH | Swarnalata | 4 | 69.53 | 95.98 | 57.22 |
| 2 | WBPH | Swarnalata | 5 | 69.62 | 93.26 | 63.03 |
| 2 | WBPH | Swarnalata | 6 | 76.15 | 86.89 | 72.12 |
| 2 | WBPH | Swarnalata | 7 | 98.09 | 108.06 | 74.54 |
| 2 | WBPH | Swarnalata | 8 | 113.62 | 115.20 | 102.75 |
| 2 | WBPH | Swarnalata | 9 | 103.05 | 103.26 | 95.09 |
| 2 | WBPH | Swarnalata | 10 | 92.48 | 91.32 | 87.42 |
| 3 | BPH | Swarnalata | 1 | 97.28 | 127.71 | 67.83 |
| 3 | BPH | Swarnalata | 2 | 89.22 | 121.97 | 64.29 |
| 3 | BPH | Swarnalata | 3 | 81.16 | 116.22 | 60.74 |
| 3 | BPH | Swarnalata | 4 | 78.86 | 112.86 | 60.38 |
| 3 | BPH | Swarnalata | 5 | 76.55 | 109.49 | 60.02 |
| 3 | BPH | Swarnalata | 6 | 84.84 | 116.51 | 58.62 |
| 3 | BPH | Swarnalata | 7 | 85.52 | 116.84 | 60.41 |
| 3 | BPH | Swarnalata | 8 | 86.20 | 117.16 | 62.20 |
| 3 | BPH | Swarnalata | 9 | 90.07 | 113.64 | 57.24 |
| 3 | BPH | Swarnalata | 10 | 110.34 | 137.84 | 88.08 |
| 3 | CONTROL | Swarnalata | 1 | 87.22 | 122.48 | 59.98 |
| 3 | CONTROL | Swarnalata | 2 | 83.99 | 119.97 | 60.89 |
| 3 | CONTROL | Swarnalata | 3 | 80.75 | 117.46 | 61.79 |
| 3 | CONTROL | Swarnalata | 4 | 84.34 | 120.20 | 59.13 |
| 3 | CONTROL | Swarnalata | 5 | 87.92 | 122.94 | 56.46 |
| 3 | CONTROL | Swarnalata | 6 | 77.24 | 107.59 | 49.38 |
| 3 | CONTROL | Swarnalata | 7 | 90.48 | 122.79 | 54.65 |
| 3 | CONTROL | Swarnalata | 8 | 103.71 | 137.98 | 59.91 |
| 3 | CONTROL | Swarnalata | 9 | 97.98 | 131.00 | 63.49 |
| 3 | CONTROL | Swarnalata | 10 | 110.44 | 146.70 | 73.26 |
| 3 | WBPH | Swarnalata | 1 | 77.01 | 105.67 | 54.06 |
| 3 | WBPH | Swarnalata | 2 | 72.91 | 103.82 | 54.88 |
| 3 | WBPH | Swarnalata | 3 | 68.80 | 101.97 | 55.70 |
| 3 | WBPH | Swarnalata | 4 | 67.94 | 100.06 | 56.40 |
| 3 | WBPH | Swarnalata | 5 | 67.07 | 98.15 | 57.10 |
| 3 | WBPH | Swarnalata | 6 | 75.67 | 106.68 | 56.11 |
| 3 | WBPH | Swarnalata | 7 | 90.03 | 119.66 | 58.82 |
| 3 | WBPH | Swarnalata | 8 | 104.38 | 132.64 | 61.52 |
| 3 | WBPH | Swarnalata | 9 | 114.28 | 139.08 | 76.84 |
| 3 | WBPH | Swarnalata | 10 | 118.62 | 141.08 | 81.88 |
| 1 | BPH | T65 | 1 | 88.83 | 126.97 | 79.84 |
| 1 | BPH | T65 | 2 | 60.41 | 92.41 | 65.82 |
| 1 | BPH | T65 | 3 | 71.05 | 106.21 | 64.31 |
| 1 | BPH | T65 | 4 | 63.70 | 91.49 | 63.10 |
| 1 | BPH | T65 | 5 | 77.75 | 109.61 | 82.46 |
| 1 | BPH | T65 | 6 | 80.92 | 108.82 | 70.71 |
| 1 | BPH | T65 | 7 | 101.89 | 114.73 | 94.23 |
| 1 | BPH | T65 | 8 | 102.97 | 107.30 | 106.91 |
| 1 | BPH | T65 | 9 | 122.60 | 117.24 | 105.74 |
| 1 | BPH | T65 | 10 | 100.93 | 99.51 | 98.08 |
| 1 | CONTROL | T65 | 1 | 87.66 | 125.65 | 64.09 |
| 1 | CONTROL | T65 | 2 | 78.01 | 117.75 | 72.52 |
| 1 | CONTROL | T65 | 3 | 84.55 | 122.02 | 65.70 |
| 1 | CONTROL | T65 | 4 | 83.34 | 108.66 | 52.57 |
| 1 | CONTROL | T65 | 5 | 79.38 | 113.05 | 56.41 |
| 1 | CONTROL | T65 | 6 | 77.09 | 110.81 | 60.85 |
| 1 | CONTROL | T65 | 7 | 87.03 | 119.54 | 57.42 |
| 1 | CONTROL | T65 | 8 | 87.53 | 120.70 | 63.42 |
| 1 | CONTROL | T65 | 9 | 82.10 | 113.62 | 66.65 |
| 1 | CONTROL | T65 | 10 | 87.15 | 117.68 | 60.34 |
| 1 | WBPH | T65 | 1 | 96.18 | 133.35 | 75.36 |
| 1 | WBPH | T65 | 2 | 72.90 | 102.67 | 55.93 |
| 1 | WBPH | T65 | 3 | 76.48 | 106.77 | 59.58 |
| 1 | WBPH | T65 | 4 | 98.44 | 125.16 | 62.68 |
| 1 | WBPH | T65 | 5 | 99.38 | 127.26 | 62.88 |
| 1 | WBPH | T65 | 6 | 92.23 | 119.16 | 70.17 |
| 1 | WBPH | T65 | 7 | 91.24 | 117.54 | 64.17 |
| 1 | WBPH | T65 | 8 | 101.43 | 121.97 | 78.16 |
| 1 | WBPH | T65 | 9 | 101.14 | 113.18 | 76.09 |
| 1 | WBPH | T65 | 10 | 91.43 | 106.59 | 78.55 |
| 2 | BPH | T65 | 1 | 66.59 | 94.28 | 59.20 |
| 2 | BPH | T65 | 2 | 66.08 | 86.98 | 60.23 |
| 2 | BPH | T65 | 3 | 65.56 | 79.67 | 61.26 |
| 2 | BPH | T65 | 4 | 76.99 | 86.39 | 74.04 |
| 2 | BPH | T65 | 5 | 88.41 | 93.10 | 86.81 |
| 2 | BPH | T65 | 6 | 105.14 | 104.42 | 99.28 |
| 2 | BPH | T65 | 7 | 111.32 | 110.97 | 107.22 |
| 2 | BPH | T65 | 8 | 117.50 | 117.51 | 115.15 |
| 2 | BPH | T65 | 9 | 120.36 | 119.09 | 116.14 |
| 2 | BPH | T65 | 10 | 123.22 | 120.67 | 117.13 |
| 2 | CONTROL | T65 | 1 | 63.47 | 95.76 | 44.24 |
| 2 | CONTROL | T65 | 2 | 70.91 | 101.25 | 47.72 |
| 2 | CONTROL | T65 | 3 | 78.35 | 106.74 | 51.19 |
| 2 | CONTROL | T65 | 4 | 86.31 | 115.61 | 51.80 |
| 2 | CONTROL | T65 | 5 | 94.27 | 124.48 | 52.40 |
| 2 | CONTROL | T65 | 6 | 89.77 | 125.19 | 53.80 |
| 2 | CONTROL | T65 | 7 | 97.85 | 131.15 | 56.86 |
| 2 | CONTROL | T65 | 8 | 105.92 | 137.11 | 59.92 |
| 2 | CONTROL | T65 | 9 | 102.28 | 131.06 | 54.08 |
| 2 | CONTROL | T65 | 10 | 98.64 | 125.00 | 48.23 |
| 2 | WBPH | T65 | 1 | 57.20 | 77.25 | 58.96 |
| 2 | WBPH | T65 | 2 | 57.55 | 74.14 | 57.93 |
| 2 | WBPH | T65 | 3 | 57.90 | 71.02 | 56.90 |
| 2 | WBPH | T65 | 4 | 71.75 | 85.17 | 65.86 |
| 2 | WBPH | T65 | 5 | 85.90 | 86.31 | 74.31 |
| 2 | WBPH | T65 | 6 | 94.56 | 95.85 | 90.94 |
| 2 | WBPH | T65 | 7 | 102.01 | 107.15 | 99.17 |
| 2 | WBPH | T65 | 8 | 127.86 | 127.40 | 126.21 |
| 2 | WBPH | T65 | 9 | 118.88 | 117.56 | 115.81 |
| 2 | WBPH | T65 | 10 | 109.90 | 107.71 | 105.41 |
| 3 | BPH | T65 | 1 | 78.91 | 112.16 | 63.21 |
| 3 | BPH | T65 | 2 | 83.13 | 117.77 | 66.49 |
| 3 | BPH | T65 | 3 | 87.35 | 123.38 | 69.77 |
| 3 | BPH | T65 | 4 | 84.71 | 115.71 | 69.75 |
| 3 | BPH | T65 | 5 | 82.07 | 108.03 | 69.72 |
| 3 | BPH | T65 | 6 | 79.04 | 99.97 | 78.26 |
| 3 | BPH | T65 | 7 | 93.16 | 105.86 | 91.73 |
| 3 | BPH | T65 | 8 | 107.27 | 111.74 | 105.20 |
| 3 | BPH | T65 | 9 | 124.10 | 126.60 | 127.23 |
| 3 | BPH | T65 | 10 | 132.16 | 136.43 | 140.08 |
| 3 | CONTROL | T65 | 1 | 69.71 | 103.74 | 54.67 |
| 3 | CONTROL | T65 | 2 | 63.92 | 97.77 | 54.24 |
| 3 | CONTROL | T65 | 3 | 58.12 | 91.79 | 53.80 |
| 3 | CONTROL | T65 | 4 | 67.20 | 100.59 | 52.44 |
| 3 | CONTROL | T65 | 5 | 76.27 | 109.39 | 51.07 |
| 3 | CONTROL | T65 | 6 | 77.64 | 110.24 | 52.29 |
| 3 | CONTROL | T65 | 7 | 76.36 | 109.33 | 55.37 |
| 3 | CONTROL | T65 | 8 | 75.08 | 108.42 | 58.44 |
| 3 | CONTROL | T65 | 9 | 80.62 | 112.44 | 51.09 |
| 3 | CONTROL | T65 | 10 | 90.75 | 127.09 | 68.15 |
| 3 | WBPH | T65 | 1 | 68.45 | 100.19 | 66.43 |
| 3 | WBPH | T65 | 2 | 72.47 | 103.54 | 67.70 |
| 3 | WBPH | T65 | 3 | 76.48 | 106.88 | 68.96 |
| 3 | WBPH | T65 | 4 | 73.89 | 102.29 | 70.27 |
| 3 | WBPH | T65 | 5 | 71.29 | 97.69 | 71.58 |
| 3 | WBPH | T65 | 6 | 87.21 | 107.19 | 74.39 |
| 3 | WBPH | T65 | 7 | 91.84 | 108.71 | 80.75 |
| 3 | WBPH | T65 | 8 | 96.47 | 110.22 | 87.11 |
| 3 | WBPH | T65 | 9 | 95.48 | 112.17 | 95.34 |
| 3 | WBPH | T65 | 10 | 128.37 | 143.27 | 120.50 |
| 1 | BPH | TN1 | 1 | 98.59 | 132.78 | 68.76 |
| 1 | BPH | TN1 | 2 | 80.77 | 112.40 | 68.02 |
| 1 | BPH | TN1 | 3 | 81.31 | 110.58 | 67.55 |
| 1 | BPH | TN1 | 4 | 85.04 | 103.64 | 74.09 |
| 1 | BPH | TN1 | 5 | 96.85 | 106.17 | 76.67 |
| 1 | BPH | TN1 | 6 | 98.00 | 108.40 | 87.70 |
| 1 | BPH | TN1 | 7 | 109.55 | 110.94 | 93.32 |
| 1 | BPH | TN1 | 8 | 107.61 | 108.56 | 99.25 |
| 1 | BPH | TN1 | 9 | 115.62 | 111.28 | 102.75 |
| 1 | BPH | TN1 | 10 | 101.04 | 96.71 | 89.32 |
| 1 | CONTROL | TN1 | 1 | 87.16 | 122.58 | 56.72 |
| 1 | CONTROL | TN1 | 2 | 81.49 | 119.61 | 67.62 |
| 1 | CONTROL | TN1 | 4 | 77.74 | 109.19 | 53.36 |
| 1 | CONTROL | TN1 | 5 | 86.16 | 118.01 | 55.53 |
| 1 | CONTROL | TN1 | 6 | 78.67 | 106.84 | 52.40 |
| 1 | CONTROL | TN1 | 7 | 85.28 | 110.86 | 47.33 |
| 1 | CONTROL | TN1 | 8 | 94.02 | 124.88 | 60.10 |
| 1 | CONTROL | TN1 | 9 | 106.59 | 132.50 | 63.03 |
| 1 | CONTROL | TN1 | 10 | 86.67 | 113.57 | 56.24 |
| 1 | WBPH | TN1 | 1 | 101.28 | 135.24 | 67.82 |
| 1 | WBPH | TN1 | 2 | 79.13 | 108.24 | 58.17 |
| 1 | WBPH | TN1 | 3 | 95.02 | 123.17 | 65.55 |
| 1 | WBPH | TN1 | 4 | 98.04 | 126.06 | 71.58 |
| 1 | WBPH | TN1 | 5 | 98.46 | 120.30 | 67.58 |
| 1 | WBPH | TN1 | 6 | 97.92 | 118.82 | 75.92 |
| 1 | WBPH | TN1 | 7 | 95.18 | 109.68 | 74.71 |
| 1 | WBPH | TN1 | 8 | 108.34 | 119.89 | 91.65 |
| 1 | WBPH | TN1 | 9 | 105.09 | 115.46 | 85.79 |
| 1 | WBPH | TN1 | 10 | 104.18 | 111.60 | 91.22 |
| 2 | BPH | TN1 | 1 | 71.86 | 101.13 | 53.56 |
| 2 | BPH | TN1 | 2 | 72.67 | 95.09 | 54.83 |
| 2 | BPH | TN1 | 3 | 73.49 | 89.05 | 56.11 |
| 2 | BPH | TN1 | 4 | 84.25 | 95.87 | 65.84 |
| 2 | BPH | TN1 | 5 | 95.00 | 102.70 | 75.57 |
| 2 | BPH | TN1 | 6 | 103.43 | 102.83 | 87.11 |
| 2 | BPH | TN1 | 7 | 110.91 | 107.97 | 94.17 |
| 2 | BPH | TN1 | 8 | 118.39 | 113.10 | 101.24 |
| 2 | BPH | TN1 | 9 | 118.55 | 112.70 | 102.66 |
| 2 | BPH | TN1 | 10 | 118.71 | 112.31 | 104.09 |
| 2 | CONTROL | TN1 | 1 | 78.83 | 106.63 | 44.36 |
| 2 | CONTROL | TN1 | 3 | 65.71 | 101.53 | 61.81 |
| 2 | CONTROL | TN1 | 5 | 79.91 | 110.66 | 43.63 |
| 2 | CONTROL | TN1 | 6 | 87.25 | 116.03 | 41.23 |
| 2 | CONTROL | TN1 | 8 | 98.72 | 131.24 | 52.85 |
| 2 | CONTROL | TN1 | 10 | 96.39 | 126.06 | 52.30 |
| 2 | WBPH | TN1 | 1 | 71.46 | 95.43 | 52.90 |
| 2 | WBPH | TN1 | 3 | 76.75 | 96.10 | 60.37 |
| 2 | WBPH | TN1 | 4 | 79.86 | 92.24 | 65.09 |
| 2 | WBPH | TN1 | 5 | 85.62 | 93.01 | 69.99 |
| 2 | WBPH | TN1 | 6 | 89.95 | 94.63 | 73.59 |
| 2 | WBPH | TN1 | 7 | 104.69 | 104.62 | 87.36 |
| 2 | WBPH | TN1 | 8 | 117.05 | 112.46 | 101.02 |
| 2 | WBPH | TN1 | 9 | 113.68 | 108.74 | 99.31 |
| 2 | WBPH | TN1 | 10 | 110.31 | 105.02 | 97.60 |
| 3 | BPH | TN1 | 1 | 88.12 | 115.89 | 67.44 |
| 3 | BPH | TN1 | 2 | 88.16 | 113.12 | 69.43 |
| 3 | BPH | TN1 | 3 | 88.19 | 110.34 | 71.42 |
| 3 | BPH | TN1 | 4 | 92.64 | 111.67 | 75.70 |
| 3 | BPH | TN1 | 5 | 97.09 | 112.99 | 79.97 |
| 3 | BPH | TN1 | 6 | 96.06 | 107.39 | 81.63 |
| 3 | BPH | TN1 | 7 | 105.17 | 110.99 | 89.38 |
| 3 | BPH | TN1 | 8 | 114.27 | 114.60 | 97.12 |
| 3 | BPH | TN1 | 9 | 115.84 | 112.01 | 102.70 |
| 3 | BPH | TN1 | 10 | 123.57 | 118.33 | 108.32 |
| 3 | CONTROL | TN1 | 1 | 78.48 | 111.98 | 56.82 |
| 3 | CONTROL | TN1 | 2 | 77.83 | 111.48 | 56.20 |
| 3 | CONTROL | TN1 | 3 | 77.18 | 110.97 | 55.59 |
| 3 | CONTROL | TN1 | 4 | 73.72 | 106.83 | 50.62 |
| 3 | CONTROL | TN1 | 5 | 70.26 | 102.68 | 45.65 |
| 3 | CONTROL | TN1 | 6 | 76.39 | 109.85 | 48.88 |
| 3 | CONTROL | TN1 | 7 | 78.12 | 111.43 | 49.29 |
| 3 | CONTROL | TN1 | 8 | 79.84 | 113.01 | 49.69 |
| 3 | CONTROL | TN1 | 9 | 85.49 | 119.95 | 55.34 |
| 3 | CONTROL | TN1 | 10 | 96.33 | 132.76 | 60.96 |
| 3 | WBPH | TN1 | 1 | 83.61 | 105.67 | 67.43 |
| 3 | WBPH | TN1 | 2 | 85.91 | 107.41 | 71.12 |
| 3 | WBPH | TN1 | 3 | 88.21 | 109.15 | 74.80 |
| 3 | WBPH | TN1 | 4 | 90.15 | 106.61 | 74.62 |
| 3 | WBPH | TN1 | 5 | 92.09 | 104.07 | 74.43 |
| 3 | WBPH | TN1 | 6 | 97.24 | 104.35 | 79.94 |
| 3 | WBPH | TN1 | 7 | 101.70 | 107.29 | 83.27 |
| 3 | WBPH | TN1 | 8 | 106.15 | 110.22 | 86.59 |
| 3 | WBPH | TN1 | 9 | 108.17 | 108.82 | 94.07 |
| 3 | WBPH | TN1 | 10 | 124.65 | 125.84 | 109.39 |
| 1 | BPH | Triveni | 1 | 101.41 | 141.49 | 71.54 |
| 1 | BPH | Triveni | 2 | 74.61 | 109.77 | 59.16 |
| 1 | BPH | Triveni | 3 | 81.55 | 114.75 | 55.27 |
| 1 | BPH | Triveni | 4 | 77.66 | 110.13 | 52.37 |
| 1 | BPH | Triveni | 5 | 79.12 | 102.99 | 51.00 |
| 1 | BPH | Triveni | 6 | 84.95 | 109.96 | 58.55 |
| 1 | BPH | Triveni | 7 | 102.70 | 129.27 | 60.82 |
| 1 | BPH | Triveni | 8 | 91.16 | 117.64 | 72.01 |
| 1 | BPH | Triveni | 9 | 100.30 | 123.20 | 81.55 |
| 1 | BPH | Triveni | 10 | 84.29 | 93.11 | 72.55 |
| 1 | CONTROL | Triveni | 1 | 92.87 | 130.70 | 55.82 |
| 1 | CONTROL | Triveni | 2 | 86.24 | 125.19 | 61.39 |
| 1 | CONTROL | Triveni | 3 | 90.63 | 128.18 | 57.99 |
| 1 | CONTROL | Triveni | 4 | 71.90 | 106.03 | 46.53 |
| 1 | CONTROL | Triveni | 5 | 88.52 | 124.68 | 49.79 |
| 1 | CONTROL | Triveni | 6 | 60.15 | 91.13 | 44.08 |
| 1 | CONTROL | Triveni | 7 | 81.83 | 115.22 | 47.33 |
| 1 | CONTROL | Triveni | 8 | 90.62 | 121.25 | 55.13 |
| 1 | CONTROL | Triveni | 9 | 86.59 | 121.58 | 53.50 |
| 1 | CONTROL | Triveni | 10 | 77.02 | 109.19 | 49.44 |
| 1 | WBPH | Triveni | 1 | 91.80 | 127.36 | 70.67 |
| 1 | WBPH | Triveni | 2 | 76.56 | 102.14 | 53.60 |
| 1 | WBPH | Triveni | 3 | 85.22 | 115.68 | 68.56 |
| 1 | WBPH | Triveni | 4 | 97.76 | 120.04 | 67.44 |
| 1 | WBPH | Triveni | 5 | 85.11 | 100.59 | 60.99 |
| 1 | WBPH | Triveni | 6 | 85.49 | 105.40 | 79.86 |
| 1 | WBPH | Triveni | 7 | 75.59 | 89.70 | 69.21 |
| 1 | WBPH | Triveni | 8 | 104.26 | 116.01 | 91.50 |
| 1 | WBPH | Triveni | 9 | 96.77 | 104.21 | 82.58 |
| 1 | WBPH | Triveni | 10 | 103.45 | 112.13 | 95.79 |
| 2 | BPH | Triveni | 1 | 51.62 | 72.07 | 51.55 |
| 2 | BPH | Triveni | 2 | 51.31 | 70.53 | 52.08 |
| 2 | BPH | Triveni | 3 | 51.00 | 68.98 | 52.61 |
| 2 | BPH | Triveni | 4 | 62.16 | 77.52 | 63.62 |
| 2 | BPH | Triveni | 5 | 73.32 | 86.06 | 74.63 |
| 2 | BPH | Triveni | 6 | 100.67 | 100.85 | 93.39 |
| 2 | BPH | Triveni | 7 | 108.67 | 104.96 | 97.08 |
| 2 | BPH | Triveni | 8 | 116.66 | 109.07 | 100.76 |
| 2 | BPH | Triveni | 9 | 119.44 | 111.56 | 104.56 |
| 2 | BPH | Triveni | 10 | 122.21 | 114.05 | 108.36 |
| 2 | CONTROL | Triveni | 1 | 90.78 | 118.98 | 38.12 |
| 2 | CONTROL | Triveni | 2 | 77.77 | 110.57 | 49.36 |
| 2 | CONTROL | Triveni | 3 | 64.75 | 102.16 | 60.59 |
| 2 | CONTROL | Triveni | 4 | 77.77 | 113.14 | 52.44 |
| 2 | CONTROL | Triveni | 5 | 90.79 | 124.12 | 44.28 |
| 2 | CONTROL | Triveni | 6 | 101.20 | 122.01 | 33.32 |
| 2 | CONTROL | Triveni | 7 | 95.58 | 122.99 | 34.65 |
| 2 | CONTROL | Triveni | 8 | 89.95 | 123.97 | 35.98 |
| 2 | CONTROL | Triveni | 9 | 91.09 | 125.30 | 39.99 |
| 2 | CONTROL | Triveni | 10 | 92.22 | 126.62 | 44.00 |
| 2 | WBPH | Triveni | 1 | 80.19 | 99.01 | 47.27 |
| 2 | WBPH | Triveni | 2 | 77.01 | 91.33 | 53.46 |
| 2 | WBPH | Triveni | 3 | 73.82 | 83.64 | 59.64 |
| 2 | WBPH | Triveni | 4 | 78.89 | 90.96 | 67.04 |
| 2 | WBPH | Triveni | 5 | 91.21 | 88.60 | 73.36 |
| 2 | WBPH | Triveni | 6 | 91.25 | 89.45 | 78.91 |
| 2 | WBPH | Triveni | 7 | 103.77 | 101.11 | 91.76 |
| 2 | WBPH | Triveni | 8 | 119.45 | 112.17 | 100.31 |
| 2 | WBPH | Triveni | 9 | 114.74 | 107.88 | 96.93 |
| 2 | WBPH | Triveni | 10 | 110.03 | 103.58 | 93.55 |
| 3 | BPH | Triveni | 1 | 90.31 | 123.15 | 48.68 |
| 3 | BPH | Triveni | 2 | 87.36 | 120.41 | 56.06 |
| 3 | BPH | Triveni | 3 | 84.40 | 117.66 | 63.44 |
| 3 | BPH | Triveni | 4 | 85.31 | 118.09 | 63.33 |
| 3 | BPH | Triveni | 5 | 86.22 | 118.52 | 63.21 |
| 3 | BPH | Triveni | 6 | 72.82 | 97.24 | 53.91 |
| 3 | BPH | Triveni | 7 | 79.90 | 103.14 | 57.33 |
| 3 | BPH | Triveni | 8 | 86.98 | 109.04 | 60.75 |
| 3 | BPH | Triveni | 9 | 88.34 | 111.32 | 66.18 |
| 3 | BPH | Triveni | 10 | 109.72 | 135.72 | 79.66 |
| 3 | CONTROL | Triveni | 1 | 75.93 | 106.55 | 45.58 |
| 3 | CONTROL | Triveni | 2 | 84.14 | 118.59 | 53.19 |
| 3 | CONTROL | Triveni | 3 | 92.34 | 130.62 | 60.79 |
| 3 | CONTROL | Triveni | 4 | 84.14 | 119.73 | 52.30 |
| 3 | CONTROL | Triveni | 5 | 75.93 | 108.84 | 43.81 |
| 3 | CONTROL | Triveni | 6 | 76.62 | 108.74 | 42.78 |
| 3 | CONTROL | Triveni | 7 | 71.20 | 103.31 | 44.33 |
| 3 | CONTROL | Triveni | 8 | 65.77 | 97.87 | 45.88 |
| 3 | CONTROL | Triveni | 9 | 80.85 | 113.43 | 48.22 |
| 3 | CONTROL | Triveni | 10 | 98.88 | 133.14 | 51.02 |
| 3 | WBPH | Triveni | 1 | 79.20 | 105.48 | 50.07 |
| 3 | WBPH | Triveni | 2 | 78.52 | 105.03 | 47.52 |
| 3 | WBPH | Triveni | 3 | 77.84 | 104.58 | 44.96 |
| 3 | WBPH | Triveni | 4 | 72.53 | 100.65 | 46.04 |
| 3 | WBPH | Triveni | 5 | 67.22 | 96.71 | 47.12 |
| 3 | WBPH | Triveni | 6 | 74.10 | 103.18 | 58.76 |
| 3 | WBPH | Triveni | 7 | 81.60 | 108.87 | 62.63 |
| 3 | WBPH | Triveni | 8 | 89.10 | 114.55 | 66.49 |
| 3 | WBPH | Triveni | 9 | 92.10 | 117.46 | 66.52 |
| 3 | WBPH | Triveni | 10 | 103.62 | 126.08 | 70.70 |
| 1 | BPH | UtriRajapan | 1 | 107.28 | 143.43 | 71.98 |
| 1 | BPH | UtriRajapan | 2 | 80.53 | 111.51 | 65.55 |
| 1 | BPH | UtriRajapan | 3 | 97.88 | 129.52 | 73.50 |
| 1 | BPH | UtriRajapan | 4 | 94.04 | 113.75 | 64.64 |
| 1 | BPH | UtriRajapan | 5 | 115.97 | 132.60 | 72.20 |
| 1 | BPH | UtriRajapan | 6 | 112.91 | 123.52 | 94.18 |
| 1 | BPH | UtriRajapan | 7 | 115.66 | 121.02 | 93.39 |
| 1 | BPH | UtriRajapan | 8 | 101.16 | 106.51 | 94.48 |
| 1 | BPH | UtriRajapan | 9 | 110.10 | 104.92 | 88.79 |
| 1 | BPH | UtriRajapan | 10 | 99.85 | 96.32 | 86.40 |
| 1 | CONTROL | UtriRajapan | 1 | 107.28 | 140.89 | 55.07 |
| 1 | CONTROL | UtriRajapan | 2 | 106.81 | 141.76 | 65.85 |
| 1 | CONTROL | UtriRajapan | 3 | 111.50 | 139.36 | 44.99 |
| 1 | CONTROL | UtriRajapan | 4 | 93.11 | 117.24 | 39.75 |
| 1 | CONTROL | UtriRajapan | 5 | 125.35 | 149.27 | 45.16 |
| 1 | CONTROL | UtriRajapan | 6 | 96.93 | 120.54 | 46.74 |
| 1 | CONTROL | UtriRajapan | 7 | 106.36 | 127.90 | 40.47 |
| 1 | CONTROL | UtriRajapan | 8 | 122.03 | 145.86 | 44.30 |
| 1 | CONTROL | UtriRajapan | 9 | 127.09 | 161.15 | 55.62 |
| 1 | CONTROL | UtriRajapan | 10 | 113.42 | 136.15 | 42.93 |
| 1 | WBPH | UtriRajapan | 1 | 117.44 | 144.30 | 62.68 |
| 1 | WBPH | UtriRajapan | 2 | 95.14 | 118.65 | 57.32 |
| 1 | WBPH | UtriRajapan | 3 | 117.11 | 137.29 | 64.75 |
| 1 | WBPH | UtriRajapan | 4 | 123.98 | 150.97 | 79.52 |
| 1 | WBPH | UtriRajapan | 5 | 112.82 | 130.52 | 75.62 |
| 1 | WBPH | UtriRajapan | 6 | 127.50 | 148.18 | 105.31 |
| 1 | WBPH | UtriRajapan | 7 | 131.28 | 144.14 | 100.52 |
| 1 | WBPH | UtriRajapan | 8 | 126.83 | 144.62 | 108.39 |
| 1 | WBPH | UtriRajapan | 9 | 122.48 | 137.84 | 96.93 |
| 1 | WBPH | UtriRajapan | 10 | 122.44 | 133.28 | 86.69 |
| 2 | BPH | UtriRajapan | 1 | 83.50 | 115.67 | 54.53 |
| 2 | BPH | UtriRajapan | 2 | 75.45 | 104.57 | 56.34 |
| 2 | BPH | UtriRajapan | 3 | 67.39 | 93.47 | 58.15 |
| 2 | BPH | UtriRajapan | 4 | 71.73 | 94.43 | 63.97 |
| 2 | BPH | UtriRajapan | 5 | 76.07 | 95.39 | 69.78 |
| 2 | BPH | UtriRajapan | 6 | 85.94 | 90.86 | 79.66 |
| 2 | BPH | UtriRajapan | 7 | 102.58 | 104.35 | 95.19 |
| 2 | BPH | UtriRajapan | 8 | 119.21 | 117.84 | 110.72 |
| 2 | BPH | UtriRajapan | 9 | 117.94 | 114.62 | 106.79 |
| 2 | BPH | UtriRajapan | 10 | 116.67 | 111.39 | 102.85 |
| 2 | CONTROL | UtriRajapan | 1 | 97.75 | 125.21 | 51.62 |
| 2 | CONTROL | UtriRajapan | 2 | 91.40 | 120.30 | 53.77 |
| 2 | CONTROL | UtriRajapan | 3 | 85.05 | 115.38 | 55.92 |
| 2 | CONTROL | UtriRajapan | 4 | 85.68 | 117.56 | 50.36 |
| 2 | CONTROL | UtriRajapan | 5 | 86.31 | 119.73 | 44.79 |
| 2 | CONTROL | UtriRajapan | 6 | 82.09 | 103.39 | 28.59 |
| 2 | CONTROL | UtriRajapan | 7 | 91.80 | 118.55 | 34.48 |
| 2 | CONTROL | UtriRajapan | 8 | 101.51 | 133.70 | 40.37 |
| 2 | CONTROL | UtriRajapan | 9 | 93.53 | 123.86 | 38.88 |
| 2 | CONTROL | UtriRajapan | 10 | 85.54 | 114.02 | 37.38 |
| 2 | WBPH | UtriRajapan | 1 | 94.06 | 129.32 | 55.90 |
| 2 | WBPH | UtriRajapan | 2 | 86.70 | 119.18 | 53.26 |
| 2 | WBPH | UtriRajapan | 3 | 79.34 | 109.03 | 50.62 |
| 2 | WBPH | UtriRajapan | 4 | 74.49 | 99.71 | 48.62 |
| 2 | WBPH | UtriRajapan | 5 | 81.49 | 105.37 | 42.44 |
| 2 | WBPH | UtriRajapan | 6 | 88.61 | 108.93 | 73.48 |
| 2 | WBPH | UtriRajapan | 7 | 104.91 | 109.48 | 93.87 |
| 2 | WBPH | UtriRajapan | 8 | 123.38 | 124.04 | 111.22 |
| 2 | WBPH | UtriRajapan | 9 | 118.16 | 117.90 | 109.62 |
| 2 | WBPH | UtriRajapan | 10 | 112.94 | 111.75 | 108.01 |
| 3 | BPH | UtriRajapan | 1 | 103.68 | 139.99 | 65.37 |
| 3 | BPH | UtriRajapan | 2 | 93.06 | 124.73 | 63.75 |
| 3 | BPH | UtriRajapan | 3 | 82.44 | 109.47 | 62.13 |
| 3 | BPH | UtriRajapan | 4 | 90.06 | 113.74 | 72.94 |
| 3 | BPH | UtriRajapan | 5 | 97.68 | 118.01 | 83.74 |
| 3 | BPH | UtriRajapan | 6 | 110.35 | 117.31 | 89.39 |
| 3 | BPH | UtriRajapan | 7 | 115.93 | 118.46 | 101.42 |
| 3 | BPH | UtriRajapan | 8 | 121.51 | 119.61 | 113.44 |
| 3 | BPH | UtriRajapan | 9 | 120.95 | 112.50 | 106.31 |
| 3 | BPH | UtriRajapan | 10 | 144.11 | 136.57 | 129.65 |
| 3 | CONTROL | UtriRajapan | 1 | 107.26 | 144.42 | 53.41 |
| 3 | CONTROL | UtriRajapan | 2 | 105.08 | 141.59 | 52.46 |
| 3 | CONTROL | UtriRajapan | 3 | 102.90 | 138.75 | 51.51 |
| 3 | CONTROL | UtriRajapan | 4 | 95.04 | 128.58 | 48.53 |
| 3 | CONTROL | UtriRajapan | 5 | 87.17 | 118.41 | 45.55 |
| 3 | CONTROL | UtriRajapan | 6 | 102.30 | 135.24 | 50.46 |
| 3 | CONTROL | UtriRajapan | 7 | 112.05 | 145.84 | 56.13 |
| 3 | CONTROL | UtriRajapan | 8 | 121.80 | 156.44 | 61.79 |
| 3 | CONTROL | UtriRajapan | 9 | 99.22 | 130.49 | 51.57 |
| 3 | CONTROL | UtriRajapan | 10 | 120.18 | 154.29 | 59.53 |
| 3 | WBPH | UtriRajapan | 1 | 94.47 | 127.30 | 52.66 |
| 3 | WBPH | UtriRajapan | 2 | 98.37 | 129.28 | 50.93 |
| 3 | WBPH | UtriRajapan | 3 | 102.26 | 131.25 | 49.20 |
| 3 | WBPH | UtriRajapan | 4 | 94.74 | 122.78 | 49.89 |
| 3 | WBPH | UtriRajapan | 5 | 87.22 | 114.30 | 50.58 |
| 3 | WBPH | UtriRajapan | 6 | 105.71 | 122.95 | 49.27 |
| 3 | WBPH | UtriRajapan | 7 | 109.36 | 128.11 | 54.21 |
| 3 | WBPH | UtriRajapan | 8 | 113.01 | 133.27 | 59.15 |
| 3 | WBPH | UtriRajapan | 9 | 113.34 | 137.37 | 71.02 |
| 3 | WBPH | UtriRajapan | 10 | 132.97 | 153.89 | 78.68 |
| 1 | BPH | Yagyaw | 1 | 83.20 | 112.23 | 66.77 |
| 1 | BPH | Yagyaw | 2 | 102.12 | 115.40 | 83.36 |
| 1 | BPH | Yagyaw | 3 | 127.77 | 125.12 | 109.89 |
| 1 | BPH | Yagyaw | 4 | 123.39 | 118.45 | 114.86 |
| 1 | BPH | Yagyaw | 5 | 120.87 | 110.42 | 99.35 |
| 1 | BPH | Yagyaw | 6 | 106.01 | 98.96 | 88.06 |
| 1 | BPH | Yagyaw | 7 | 90.96 | 84.91 | 78.82 |
| 1 | BPH | Yagyaw | 8 | 100.59 | 96.13 | 91.67 |
| 1 | BPH | Yagyaw | 9 | 92.50 | 87.73 | 82.88 |
| 1 | BPH | Yagyaw | 10 | 90.52 | 85.50 | 79.48 |
| 1 | CONTROL | Yagyaw | 1 | 88.02 | 124.50 | 59.04 |
| 1 | CONTROL | Yagyaw | 2 | 77.39 | 114.20 | 56.59 |
| 1 | CONTROL | Yagyaw | 3 | 84.92 | 118.89 | 51.73 |
| 1 | CONTROL | Yagyaw | 4 | 81.39 | 114.97 | 53.23 |
| 1 | CONTROL | Yagyaw | 5 | 85.22 | 106.27 | 35.06 |
| 1 | CONTROL | Yagyaw | 6 | 82.00 | 107.24 | 48.27 |
| 1 | CONTROL | Yagyaw | 7 | 81.25 | 111.39 | 51.60 |
| 1 | CONTROL | Yagyaw | 8 | 105.25 | 130.89 | 48.59 |
| 1 | CONTROL | Yagyaw | 9 | 103.98 | 136.48 | 61.85 |
| 1 | CONTROL | Yagyaw | 10 | 84.73 | 113.50 | 52.25 |
| 1 | WBPH | Yagyaw | 1 | 101.77 | 134.00 | 64.80 |
| 1 | WBPH | Yagyaw | 2 | 75.71 | 102.58 | 54.29 |
| 1 | WBPH | Yagyaw | 3 | 103.65 | 133.08 | 67.14 |
| 1 | WBPH | Yagyaw | 4 | 114.66 | 136.01 | 74.87 |
| 1 | WBPH | Yagyaw | 5 | 116.64 | 123.15 | 66.25 |
| 1 | WBPH | Yagyaw | 6 | 113.26 | 120.74 | 68.85 |
| 1 | WBPH | Yagyaw | 7 | 106.78 | 112.02 | 69.14 |
| 1 | WBPH | Yagyaw | 8 | 117.92 | 123.34 | 89.50 |
| 1 | WBPH | Yagyaw | 9 | 118.98 | 126.53 | 97.54 |
| 1 | WBPH | Yagyaw | 10 | 109.46 | 118.42 | 91.43 |
| 2 | BPH | Yagyaw | 1 | 57.56 | 87.71 | 57.23 |
| 2 | BPH | Yagyaw | 2 | 64.35 | 87.08 | 57.92 |
| 2 | BPH | Yagyaw | 3 | 71.13 | 86.45 | 58.60 |
| 2 | BPH | Yagyaw | 4 | 81.43 | 90.22 | 71.62 |
| 2 | BPH | Yagyaw | 5 | 91.73 | 93.98 | 84.64 |
| 2 | BPH | Yagyaw | 6 | 111.82 | 105.89 | 99.45 |
| 2 | BPH | Yagyaw | 7 | 122.97 | 115.66 | 107.82 |
| 2 | BPH | Yagyaw | 8 | 134.12 | 125.42 | 116.19 |
| 2 | BPH | Yagyaw | 9 | 129.26 | 120.71 | 112.17 |
| 2 | BPH | Yagyaw | 10 | 124.39 | 116.00 | 108.14 |
| 2 | CONTROL | Yagyaw | 1 | 75.24 | 102.76 | 53.11 |
| 2 | CONTROL | Yagyaw | 2 | 63.81 | 97.93 | 63.18 |
| 2 | CONTROL | Yagyaw | 3 | 52.38 | 93.10 | 73.25 |
| 2 | CONTROL | Yagyaw | 4 | 64.91 | 103.85 | 67.78 |
| 2 | CONTROL | Yagyaw | 5 | 77.43 | 114.59 | 62.30 |
| 2 | CONTROL | Yagyaw | 6 | 64.93 | 98.35 | 53.14 |
| 2 | CONTROL | Yagyaw | 7 | 83.79 | 116.42 | 61.23 |
| 2 | CONTROL | Yagyaw | 8 | 102.65 | 134.49 | 69.32 |
| 2 | CONTROL | Yagyaw | 9 | 86.06 | 117.65 | 57.92 |
| 2 | CONTROL | Yagyaw | 10 | 69.46 | 100.81 | 46.51 |
| 2 | WBPH | Yagyaw | 1 | 65.26 | 90.54 | 43.66 |
| 2 | WBPH | Yagyaw | 2 | 59.13 | 85.88 | 45.09 |
| 2 | WBPH | Yagyaw | 3 | 52.99 | 81.22 | 46.51 |
| 2 | WBPH | Yagyaw | 4 | 65.15 | 84.35 | 53.38 |
| 2 | WBPH | Yagyaw | 5 | 56.47 | 85.06 | 47.12 |
| 2 | WBPH | Yagyaw | 6 | 79.01 | 100.52 | 46.78 |
| 2 | WBPH | Yagyaw | 7 | 91.31 | 100.67 | 70.28 |
| 2 | WBPH | Yagyaw | 8 | 91.37 | 111.89 | 61.64 |
| 2 | WBPH | Yagyaw | 9 | 91.84 | 112.71 | 68.10 |
| 2 | WBPH | Yagyaw | 10 | 92.31 | 113.52 | 74.56 |
| 3 | BPH | Yagyaw | 1 | 91.79 | 123.73 | 52.00 |
| 3 | BPH | Yagyaw | 2 | 87.51 | 117.01 | 52.36 |
| 3 | BPH | Yagyaw | 3 | 83.23 | 110.28 | 52.71 |
| 3 | BPH | Yagyaw | 4 | 76.61 | 105.00 | 53.69 |
| 3 | BPH | Yagyaw | 5 | 69.99 | 99.71 | 54.67 |
| 3 | BPH | Yagyaw | 6 | 82.36 | 104.40 | 60.87 |
| 3 | BPH | Yagyaw | 7 | 90.02 | 106.29 | 75.71 |
| 3 | BPH | Yagyaw | 8 | 97.67 | 108.17 | 90.55 |
| 3 | BPH | Yagyaw | 9 | 129.50 | 119.28 | 109.13 |
| 3 | BPH | Yagyaw | 10 | 155.53 | 144.00 | 131.62 |
| 3 | CONTROL | Yagyaw | 1 | 65.61 | 99.03 | 46.11 |
| 3 | CONTROL | Yagyaw | 2 | 67.48 | 101.84 | 48.50 |
| 3 | CONTROL | Yagyaw | 3 | 69.34 | 104.64 | 50.88 |
| 3 | CONTROL | Yagyaw | 4 | 73.52 | 107.58 | 47.63 |
| 3 | CONTROL | Yagyaw | 5 | 77.70 | 110.51 | 44.38 |
| 3 | CONTROL | Yagyaw | 6 | 68.93 | 100.86 | 45.20 |
| 3 | CONTROL | Yagyaw | 7 | 77.04 | 110.13 | 45.98 |
| 3 | CONTROL | Yagyaw | 8 | 85.15 | 119.39 | 46.76 |
| 3 | CONTROL | Yagyaw | 9 | 92.33 | 127.51 | 52.34 |
| 3 | CONTROL | Yagyaw | 10 | 96.41 | 132.24 | 57.27 |
| 3 | WBPH | Yagyaw | 1 | 76.10 | 107.52 | 57.76 |
| 3 | WBPH | Yagyaw | 2 | 80.21 | 107.29 | 53.29 |
| 3 | WBPH | Yagyaw | 3 | 84.32 | 107.06 | 48.82 |
| 3 | WBPH | Yagyaw | 4 | 81.78 | 105.78 | 52.68 |
| 3 | WBPH | Yagyaw | 5 | 79.23 | 104.49 | 56.54 |
| 3 | WBPH | Yagyaw | 6 | 96.96 | 116.12 | 61.08 |
| 3 | WBPH | Yagyaw | 7 | 107.76 | 124.61 | 69.35 |
| 3 | WBPH | Yagyaw | 8 | 118.56 | 133.10 | 77.62 |
| 3 | WBPH | Yagyaw | 9 | 113.00 | 125.80 | 83.66 |
| 3 | WBPH | Yagyaw | 10 | 131.94 | 142.02 | 93.85 |
