# Supplementary material for: Changes in reflectance of rice seedlings during planthopper feeding as detected by digital camera: Potential applications for high-throughput phenotyping
Source: PLoS One. 2020 Aug 27;15(8):e0238173. doi: 10.1371/journal.pone.0238173 (PMC7451558; doi:10.1371/journal.pone.0238173)
Supplement: S10 Table — (DOCX) [file pone.0238173.s018.docx]

**Table S10. Damage scores and plant weights from Standard Seedling Seed-box Tests** (SSSTs) (plt_wgt = plant weight g; plt_no. = number of plants alive; BPH = brown planthopper; WBPH = whitebacked planthopper)

| Rep | Variety | Control plt_wgt | Control plt_no. | BPH damage | BPH plt_wgt | BPH plt_no. | WBPH damage | WBPH plt_wgt | WBPH plt_no. |
| --- | --- | --- | --- | --- | --- | --- | --- | --- | --- |
| 1 | ADR52 | 1.35 | 25.50 | 5.00 | 0.54 | 21.00 | 7.00 | 0.69 | 25.00 |
| 1 | ARC10239 | 1.36 | 24.00 | 9.00 | 0.41 | 13.00 | 7.00 | 0.54 | 25.00 |
| 1 | ARC10550 | 2.15 | 24.50 | 7.00 | 0.68 | 24.00 | 7.00 | 0.65 | 23.00 |
| 1 | ARC6650 | 1.15 | 16.50 | 7.00 | 0.62 | 20.00 | 7.00 | 0.49 | 23.00 |
| 1 | ASD7 | 2.16 | 25.00 | 7.00 | 0.69 | 24.00 | 7.00 | 0.37 | 23.00 |
| 1 | Asiminori | 1.07 | 25.00 | 9.00 | 0.34 | 20.00 | 7.00 | 0.22 | 22.00 |
| 1 | Babawee | 1.32 | 23.50 | 5.00 | 0.54 | 23.00 | 7.00 | 0.40 | 24.00 |
| 1 | Balamawee | 1.33 | 26.50 | 5.00 | 0.44 | 23.00 | 3.00 | 0.94 | 25.00 |
| 1 | Chinsaba | 1.08 | 25.50 | 9.00 | 0.27 | 19.00 | 7.00 | 0.45 | 26.00 |
| 1 | Da Hua Gu | 0.70 | 10.50 | 9.00 | 0.11 | 9.00 | 7.00 | 0.15 | 14.00 |
| 1 | IR22 | 0.90 | 24.00 | 9.00 | 0.15 | 12.00 | 7.00 | 0.34 | 24.00 |
| 1 | IR24 | 0.97 | 25.50 | 7.00 | 0.47 | 25.00 | 5.00 | 0.46 | 24.00 |
| 1 | IR40 | 0.82 | 23.50 | 9.00 | 0.23 | 17.00 | 7.00 | 0.25 | 20.00 |
| 1 | IR56 | 1.24 | 20.00 | 5.00 | 0.50 | 22.00 | 7.00 | 0.51 | 25.00 |
| 1 | IR60 | 1.36 | 24.00 | 7.00 | 0.26 | 24.00 | 7.00 | 0.33 | 25.00 |
| 1 | IR62 | 0.82 | 24.50 | 5.00 | 0.55 | 24.00 | 5.00 | 0.31 | 24.00 |
| 1 | IR64 | 1.22 | 24.00 | 5.00 | 0.52 | 21.00 | 5.00 | 0.53 | 25.00 |
| 1 | IR65482-4-136-2-2 | 1.57 | 24.50 | 7.00 | 0.43 | 20.00 | 5.00 | 0.69 | 24.00 |
| 1 | IR65482-7-216-1-2-B | 1.51 | 25.00 | 7.00 | 0.39 | 22.00 | 7.00 | 0.37 | 25.00 |
| 1 | IR66 | 0.83 | 24.50 | 7.00 | 0.26 | 23.00 | 5.00 | 0.79 | 25.00 |
| 1 | IR70 | 1.09 | 25.50 | 9.00 | 0.23 | 13.00 | 7.00 | 0.33 | 25.00 |
| 1 | IR71033 | 0.85 | 17.50 | 7.00 | 0.17 | 17.00 | 5.00 | 0.19 | 15.00 |
| 1 | IR72 | 1.55 | 27.00 | 7.00 | 0.29 | 21.00 | 5.00 | 0.53 | 21.00 |
| 1 | IR74 | 0.90 | 25.50 | 7.00 | 0.38 | 22.00 | 5.00 | 0.35 | 25.00 |
| 1 | MO1 | 0.72 | 13.50 | 5.00 | 0.32 | 15.00 | 3.00 | 1.12 | 17.00 |
| 1 | Mudgo | 1.34 | 24.00 | 9.00 | 0.79 | 17.00 | 5.00 | 0.69 | 25.00 |
| 1 | N22 | 0.97 | 25.00 | 9.00 | 0.10 | 9.00 | 5.00 | 0.63 | 25.00 |
| 1 | N'Diang Marie | 2.12 | 23.00 | 9.00 | 0.30 | 18.00 | 3.00 | 0.47 | 24.00 |
| 1 | Pokkali | 2.41 | 26.50 | 7.00 | 0.73 | 25.00 | 3.00 | 1.52 | 26.00 |
| 1 | Ptb33 | 1.59 | 24.50 | 3.00 | 1.03 | 23.00 | 7.00 | 0.45 | 25.00 |
| 1 | Rathu Heenati | 1.63 | 24.50 | 3.00 | 0.86 | 24.00 | 3.00 | 0.53 | 25.00 |
| 1 | Swarnalata | 1.23 | 23.50 | 7.00 | 0.30 | 21.00 | 7.00 | 0.34 | 26.00 |
| 1 | T65 | 1.07 | 22.50 | 9.00 | 0.16 | 15.00 | 7.00 | 0.32 | 26.00 |
| 1 | TN1 | 1.15 | 18.36 | 8.71 | 0.27 | 15.57 | 7.57 | 0.28 | 17.71 |
| 1 | Triveni | 1.09 | 20.00 | 7.00 | 0.42 | 18.00 | 7.00 | 0.22 | 19.00 |
| 1 | Utri Rajapan | 1.92 | 25.50 | 9.00 | 0.29 | 12.00 | 7.00 | 0.56 | 25.00 |
| 1 | Yagyaw | 1.02 | 21.50 | 9.00 | 0.23 | 19.00 | 7.00 | 0.43 | 25.00 |
| 2 | ADR52 | 0.75 | 20.50 | 9.00 | 0.28 | 22.00 | 5.00 | 0.61 | 25.00 |
| 2 | ARC10239 | 0.76 | 23.50 | 9.00 | 0.13 | 24.00 | 9.00 | 0.24 | 20.00 |
| 2 | ARC10550 | 1.05 | 24.50 | 9.00 | 0.24 | 26.00 | 9.00 | 0.38 | 21.00 |
| 2 | ARC6650 | 0.66 | 15.00 | 9.00 | 0.15 | 16.00 | 7.00 | 0.34 | 18.00 |
| 2 | ASD7 | 0.74 | 17.50 | 9.00 | 0.05 | 14.00 | 9.00 | 0.25 | 21.00 |
| 2 | Asiminori | 0.52 | 18.50 | 9.00 | 0.12 | 23.00 | 9.00 | 0.11 | 19.00 |
| 2 | Babawee | 0.69 | 23.00 | 7.00 | 0.23 | 23.00 | 7.00 | 0.10 | 12.00 |
| 2 | Balamawee | 0.78 | 26.00 | 7.00 | 0.29 | 25.00 | 3.00 | 0.38 | 24.00 |
| 2 | Chinsaba | 0.90 | 26.00 | 9.00 | 0.21 | 22.00 | 7.00 | 0.35 | 25.00 |
| 2 | Da Hua Gu | 0.25 | 10.50 | 9.00 | 0.04 | 13.00 | 9.00 | 0.09 | 11.00 |
| 2 | IR22 | 0.70 | 22.50 | 9.00 | 0.16 | 24.00 | 9.00 | 0.19 | 24.00 |
| 2 | IR24 | 0.82 | 25.50 | 9.00 | 0.22 | 25.00 | 5.00 | 0.38 | 24.00 |
| 2 | IR40 | 0.65 | 24.00 | 9.00 | 0.14 | 25.00 | 9.00 | 0.19 | 24.00 |
| 2 | IR56 | 0.66 | 24.50 | 7.00 | 0.16 | 23.00 | 7.00 | 0.21 | 22.00 |
| 2 | IR60 | 0.85 | 24.50 | 7.00 | 0.11 | 25.00 | 7.00 | 0.22 | 24.00 |
| 2 | IR62 | 0.54 | 21.00 | 7.00 | 0.27 | 23.00 | 7.00 | 0.13 | 17.00 |
| 2 | IR64 | 0.74 | 25.00 | 7.00 | 0.14 | 25.00 | 7.00 | 0.41 | 24.00 |
| 2 | IR65482-4-136-2-2 | 0.94 | 25.00 | 5.00 | 0.37 | 26.00 | 7.00 | 0.26 | 24.00 |
| 2 | IR65482-7-216-1-2-B | 0.87 | 25.00 | 9.00 | 0.25 | 25.00 | 7.00 | 0.31 | 25.00 |
| 2 | IR66 | 0.51 | 22.00 | 9.00 | 0.15 | 25.00 | 7.00 | 0.26 | 25.00 |
| 2 | IR70 | 0.71 | 25.00 | 7.00 | 0.16 | 22.00 | 9.00 | 0.17 | 23.00 |
| 2 | IR71033 | 0.53 | 14.50 | 5.00 | 0.12 | 15.00 | 7.00 | 0.15 | 15.00 |
| 2 | IR72 | 0.68 | 25.00 | 9.00 | 0.20 | 24.00 | 7.00 | 0.25 | 26.00 |
| 2 | IR74 | 0.78 | 26.50 | 5.00 | 0.27 | 28.00 | 7.00 | 0.30 | 25.00 |
| 2 | MO1 | 0.72 | 16.50 | 5.00 | 0.37 | 17.00 | 7.00 | 0.19 | 14.00 |
| 2 | Mudgo | 1.37 | 26.00 | 9.00 | 0.33 | 26.00 | 9.00 | 0.29 | 17.00 |
| 2 | N22 | 0.51 | 20.00 | 9.00 | 0.11 | 21.00 | 9.00 | 0.21 | 22.00 |
| 2 | N'Diang Marie | 0.84 | 21.50 | 9.00 | 0.18 | 24.00 | 7.00 | 0.27 | 19.00 |
| 2 | Pokkali | 1.33 | 26.50 | 9.00 | 0.36 | 24.00 | 5.00 | 0.51 | 24.00 |
| 2 | Ptb33 | 0.15 | 6.00 | 7.00 | 0.04 | 4.00 | 9.00 | 0.25 | 20.00 |
| 2 | Rathu Heenati | 0.97 | 24.50 | 5.00 | 0.39 | 24.00 | 7.00 | 0.32 | 22.00 |
| 2 | Swarnalata | 0.36 | 10.50 | 7.00 | 0.15 | 20.00 | 9.00 | 0.09 | 11.00 |
| 2 | T65 | 0.63 | 24.00 | 9.00 | 0.14 | 22.00 | 9.00 | 0.10 | 18.00 |
| 2 | TN1 | 0.74 | 23.21 | 9.00 | 0.19 | 21.57 | 9.00 | 0.19 | 22.71 |
| 2 | Triveni | 0.70 | 24.50 | 9.00 | 0.11 | 19.00 | 9.00 | 0.14 | 20.00 |
| 2 | Utri Rajapan | 0.96 | 25.00 | 9.00 | 0.19 | 24.00 | 9.00 | 0.36 | 25.00 |
| 2 | Yagyaw | 0.58 | 21.50 | 9.00 | 0.21 | 25.00 | 7.00 | 0.30 | 22.00 |
| 3 | ADR52 | 1.31 | 25.00 | 3.00 | 0.56 | 25.00 | 7.00 | 0.41 | 25.00 |
| 3 | ARC10239 | 0.96 | 23.00 | 9.00 | 0.26 | 25.00 | 7.00 | 0.44 | 25.00 |
| 3 | ARC10550 | 1.29 | 24.50 | 9.00 | 0.49 | 24.00 | 9.00 | 0.38 | 25.00 |
| 3 | ARC6650 | 0.82 | 13.50 | 7.00 | 0.54 | 25.00 | 7.00 | 0.50 | 24.00 |
| 3 | ASD7 | 0.86 | 25.00 | 9.00 | 0.38 | 25.00 | 9.00 | 0.43 | 25.00 |
| 3 | Asiminori | 0.71 | 25.00 | 9.00 | 0.14 | 22.00 | 7.00 | 0.30 | 26.00 |
| 3 | Babawee | 0.88 | 22.50 | 7.00 | 0.43 | 24.00 | 5.00 | 0.44 | 24.00 |
| 3 | Balamawee | 0.88 | 25.50 | 7.00 | 0.33 | 25.00 | 5.00 | 0.66 | 27.00 |
| 3 | Chinsaba | 0.91 | 25.50 | 9.00 | 0.31 | 24.00 | 7.00 | 0.43 | 25.00 |
| 3 | Da Hua Gu | 0.30 | 10.50 | 7.00 | 0.11 | 16.00 | 7.00 | 0.06 | 7.00 |
| 3 | IR22 | 0.91 | 24.00 | 9.00 | 0.14 | 24.00 | 7.00 | 0.22 | 25.00 |
| 3 | IR24 | 0.88 | 25.50 | 5.00 | 0.38 | 25.00 | 5.00 | 0.34 | 25.00 |
| 3 | IR40 | 0.83 | 24.50 | 9.00 | 0.22 | 24.00 | 7.00 | 0.23 | 24.00 |
| 3 | IR56 | 0.83 | 25.00 | 5.00 | 0.47 | 26.00 | 7.00 | 0.43 | 25.00 |
| 3 | IR60 | 0.78 | 25.50 | 9.00 | 0.21 | 25.00 | 9.00 | 0.26 | 26.00 |
| 3 | IR62 | 0.73 | 23.50 | 5.00 | 0.45 | 25.00 | 5.00 | 0.42 | 25.00 |
| 3 | IR64 | 0.99 | 25.00 | 7.00 | 0.32 | 25.00 | 7.00 | 0.35 | 24.00 |
| 3 | IR65482-4-136-2-2 | 1.07 | 24.50 | 5.00 | 0.65 | 26.00 | 7.00 | 0.30 | 24.00 |
| 3 | IR65482-7-216-1-2-B | 0.94 | 25.00 | 9.00 | 0.25 | 24.00 | 7.00 | 0.34 | 25.00 |
| 3 | IR66 | 0.57 | 26.00 | 9.00 | 0.21 | 25.00 | 5.00 | 0.29 | 23.00 |
| 3 | IR70 | 0.73 | 26.00 | 7.00 | 0.23 | 25.00 | 5.00 | 0.35 | 25.00 |
| 3 | IR71033 | 0.77 | 19.00 | 5.00 | 0.39 | 22.00 | 5.00 | 0.26 | 22.00 |
| 3 | IR72 | 0.86 | 25.50 | 7.00 | 0.31 | 26.00 | 7.00 | 0.30 | 25.00 |
| 3 | IR74 | 0.77 | 25.50 | 5.00 | 0.36 | 25.00 | 7.00 | 0.31 | 23.00 |
| 3 | MO1 | 0.92 | 18.50 | 5.00 | 0.42 | 18.00 | 3.00 | 0.60 | 17.00 |
| 3 | Mudgo | 1.20 | 24.50 | 9.00 | 0.32 | 23.00 | 5.00 | 0.52 | 21.00 |
| 3 | N22 | 0.80 | 22.50 | 9.00 | 0.16 | 25.00 | 3.00 | 0.60 | 27.00 |
| 3 | N'Diang Marie | 0.85 | 23.00 | 9.00 | 0.25 | 22.00 | 7.00 | 0.23 | 21.00 |
| 3 | Pokkali | 1.53 | 25.00 | 5.00 | 0.46 | 25.00 | 5.00 | 0.79 | 25.00 |
| 3 | Ptb33 | 1.12 | 25.50 | 5.00 | 0.51 | 25.00 | 7.00 | 0.42 | 26.00 |
| 3 | Rathu Heenati | 1.01 | 23.00 | 3.00 | 0.66 | 25.00 | 5.00 | 0.55 | 26.00 |
| 3 | Swarnalata | 0.67 | 19.50 | 7.00 | 0.34 | 23.00 | 5.00 | 0.37 | 25.00 |
| 3 | T65 | 0.71 | 22.00 | 9.00 | 0.20 | 24.00 | 9.00 | 0.24 | 24.00 |
| 3 | TN1 | 0.52 | 15.86 | 9.00 | 0.15 | 17.14 | 8.43 | 0.17 | 17.57 |
| 3 | Triveni | 0.61 | 18.00 | 5.00 | 0.16 | 17.00 | 5.00 | 0.24 | 19.00 |
| 3 | Utri Rajapan | 0.97 | 26.00 | 9.00 | 0.31 | 24.00 | 7.00 | 0.35 | 25.00 |
| 3 | Yagyaw | 0.81 | 24.50 | 9.00 | 0.33 | 24.00 | 7.00 | 0.27 | 22.00 |
