# Supplementary material for: Changes in reflectance of rice seedlings during planthopper feeding as detected by digital camera: Potential applications for high-throughput phenotyping
Source: PLoS One. 2020 Aug 27;15(8):e0238173. doi: 10.1371/journal.pone.0238173 (PMC7451558; doi:10.1371/journal.pone.0238173)
Supplement: S11 Table — (DOCX) [file pone.0238173.s019.docx]

**Table S11. Data from condition experiments** (Flash 0 = no, 1 = yes; Seed distribution B = broadcast, R = row; Age = seedling age; L = luminosity, R = red, G = green, B = blue; Dry_wt-g = total weight of seedling patch)

| Rep | Flash | Frame colour | Frame size (cm2) | Density (cm-2) | Seed distribution | Age (days) | mean L | mean R | mean G | mean B | Dry_wt-g |
| --- | --- | --- | --- | --- | --- | --- | --- | --- | --- | --- | --- |
| 1 | 0 | Black | 5 | 1 | B | 8 | 65.38 | 51.90 | 77.21 | 27.54 | 0.32 |
| 1 | 1 | Black | 5 | 1 | B | 8 | 96.60 | 84.42 | 116.68 | 44.61 | 0.32 |
| 1 | 0 | Blue | 5 | 1 | B | 8 | 75.41 | 54.83 | 86.53 | 38.36 | 0.32 |
| 1 | 1 | Blue | 5 | 1 | B | 8 | 92.48 | 70.31 | 109.64 | 53.13 | 0.12 |
| 1 | 0 | Black | 5 | 1 | R | 8 | 77.27 | 62.27 | 96.96 | 38.17 | 0.33 |
| 1 | 1 | Black | 5 | 1 | R | 8 | 91.00 | 84.98 | 118.68 | 39.93 | 0.33 |
| 1 | 0 | Blue | 5 | 1 | R | 8 | 85.30 | 69.45 | 103.83 | 43.96 | 0.33 |
| 1 | 1 | Blue | 5 | 1 | R | 8 | 85.85 | 74.37 | 107.23 | 42.28 | 0.32 |
| 1 | 0 | Black | 5 | 2 | B | 8 | 77.62 | 66.72 | 90.97 | 33.93 | 0.11 |
| 1 | 1 | Black | 5 | 2 | B | 8 | 100.59 | 85.95 | 116.25 | 52.59 | 0.11 |
| 1 | 0 | Blue | 5 | 2 | B | 8 | 100.83 | 74.20 | 117.34 | 79.59 | 0.11 |
| 1 | 1 | Blue | 5 | 2 | B | 8 | 85.73 | 58.10 | 96.66 | 56.33 | 0.33 |
| 1 | 0 | Black | 5 | 2 | R | 8 | 84.04 | 76.90 | 107.17 | 39.78 | 0.12 |
| 1 | 1 | Black | 5 | 2 | R | 8 | 103.09 | 92.26 | 125.42 | 53.39 | 0.12 |
| 1 | 0 | Blue | 5 | 2 | R | 8 | 104.41 | 87.04 | 118.22 | 70.09 | 0.11 |
| 1 | 1 | Blue | 5 | 2 | R | 8 | 83.17 | 64.46 | 100.80 | 52.08 | 0.12 |
| 1 | 0 | Black | 8 | 1 | B | 8 | 84.21 | 74.12 | 107.87 | 41.56 | 0.64 |
| 1 | 1 | Black | 8 | 1 | B | 8 | 97.14 | 94.50 | 132.91 | 54.11 | 0.64 |
| 1 | 0 | Blue | 8 | 1 | B | 8 | 98.75 | 80.73 | 122.61 | 56.86 | 0.64 |
| 1 | 1 | Blue | 8 | 1 | B | 8 | 84.13 | 75.55 | 111.83 | 48.53 | 0.23 |
| 1 | 0 | Black | 8 | 1 | R | 8 | 71.05 | 64.66 | 89.50 | 24.47 | 0.80 |
| 1 | 1 | Black | 8 | 1 | R | 8 | 94.34 | 86.91 | 122.11 | 42.16 | 0.80 |
| 1 | 0 | Blue | 8 | 1 | R | 8 | 83.40 | 79.01 | 99.44 | 32.96 | 0.80 |
| 1 | 1 | Blue | 8 | 1 | R | 8 | 92.63 | 81.69 | 119.19 | 45.93 | 0.64 |
| 1 | 0 | Black | 8 | 2 | B | 8 | 77.45 | 74.10 | 90.81 | 18.30 | 0.19 |
| 1 | 1 | Black | 8 | 2 | B | 8 | 106.20 | 102.81 | 140.62 | 53.61 | 0.19 |
| 1 | 0 | Blue | 8 | 2 | B | 8 | 67.55 | 43.57 | 77.89 | 40.87 | 0.19 |
| 1 | 1 | Blue | 8 | 2 | B | 8 | 88.57 | 78.11 | 112.00 | 41.77 | 0.80 |
| 1 | 0 | Black | 8 | 2 | R | 8 | 97.52 | 89.17 | 114.90 | 37.33 | 0.23 |
| 1 | 1 | Black | 8 | 2 | R | 8 | 105.08 | 103.00 | 141.51 | 56.52 | 0.23 |
| 1 | 0 | Blue | 8 | 2 | R | 8 | 104.42 | 73.12 | 124.79 | 81.47 | 0.19 |
| 1 | 1 | Blue | 8 | 2 | R | 8 | 91.16 | 72.99 | 110.90 | 52.73 | 0.23 |
| 1 | 0 | Black | 10 | 1 | B | 8 | 67.01 | 52.52 | 80.78 | 28.00 | 0.95 |
| 1 | 1 | Black | 10 | 1 | B | 8 | 89.12 | 78.19 | 111.12 | 45.66 | 0.95 |
| 1 | 0 | Blue | 10 | 1 | B | 8 | 72.91 | 60.37 | 89.18 | 28.92 | 0.95 |
| 1 | 1 | Blue | 10 | 1 | B | 8 | 88.72 | 77.23 | 112.33 | 48.70 | 0.36 |
| 1 | 0 | Black | 10 | 1 | R | 8 | 73.23 | 61.35 | 88.92 | 28.71 | 1.00 |
| 1 | 1 | Black | 10 | 1 | R | 8 | 88.51 | 84.49 | 119.85 | 50.61 | 1.00 |
| 1 | 0 | Blue | 10 | 1 | R | 8 | 86.47 | 70.27 | 108.29 | 42.80 | 1.00 |
| 1 | 1 | Blue | 10 | 1 | R | 8 | 85.93 | 75.66 | 111.80 | 46.74 | 0.95 |
| 1 | 0 | Black | 10 | 2 | B | 8 | 83.50 | 65.30 | 96.52 | 50.93 | 0.34 |
| 1 | 1 | Black | 10 | 2 | B | 8 | 102.72 | 85.32 | 120.15 | 53.73 | 0.34 |
| 1 | 0 | Blue | 10 | 2 | B | 8 | 92.46 | 72.51 | 111.18 | 52.66 | 0.34 |
| 1 | 1 | Blue | 10 | 2 | B | 8 | 94.95 | 74.41 | 111.84 | 54.67 | 1.00 |
| 1 | 0 | Black | 10 | 2 | R | 8 | 77.44 | 64.19 | 92.31 | 38.87 | 0.36 |
| 1 | 1 | Black | 10 | 2 | R | 8 | 96.50 | 88.80 | 123.08 | 53.53 | 0.36 |
| 1 | 0 | Blue | 10 | 2 | R | 8 | 89.94 | 71.53 | 101.75 | 58.24 | 0.34 |
| 1 | 1 | Blue | 10 | 2 | R | 8 | 85.41 | 66.15 | 102.19 | 50.52 | 0.36 |
| 1 | 0 | Black | 12 | 1 | B | 8 | 66.23 | 70.10 | 88.70 | 20.37 | 1.59 |
| 1 | 1 | Black | 12 | 1 | B | 8 | 93.89 | 77.83 | 111.57 | 44.93 | 1.59 |
| 1 | 0 | Blue | 12 | 1 | B | 8 | 79.33 | 67.76 | 97.04 | 29.98 | 1.59 |
| 1 | 1 | Blue | 12 | 1 | B | 8 | 88.35 | 73.09 | 105.98 | 44.29 | 0.50 |
| 1 | 0 | Black | 12 | 1 | R | 8 | 77.05 | 65.17 | 93.89 | 30.72 | 1.55 |
| 1 | 1 | Black | 12 | 1 | R | 8 | 94.33 | 84.59 | 119.52 | 52.19 | 1.55 |
| 1 | 0 | Blue | 12 | 1 | R | 8 | 93.01 | 86.14 | 110.18 | 35.09 | 1.55 |
| 1 | 1 | Blue | 12 | 1 | R | 8 | 91.50 | 73.87 | 110.44 | 49.73 | 1.59 |
| 1 | 0 | Black | 12 | 2 | B | 8 | 74.43 | 59.23 | 84.57 | 45.13 | 0.45 |
| 1 | 1 | Black | 12 | 2 | B | 8 | 100.29 | 98.98 | 136.83 | 54.16 | 0.45 |
| 1 | 0 | Blue | 12 | 2 | B | 8 | 99.50 | 93.49 | 121.59 | 47.16 | 0.45 |
| 1 | 1 | Blue | 12 | 2 | B | 8 | 98.24 | 86.67 | 125.15 | 56.83 | 1.55 |
| 1 | 0 | Black | 12 | 2 | R | 8 | 69.23 | 52.32 | 80.00 | 42.35 | 0.50 |
| 1 | 1 | Black | 12 | 2 | R | 8 | 97.36 | 96.44 | 133.68 | 50.66 | 0.50 |
| 1 | 0 | Blue | 12 | 2 | R | 8 | 96.71 | 78.21 | 119.76 | 72.86 | 0.45 |
| 1 | 1 | Blue | 12 | 2 | R | 8 | 91.59 | 73.93 | 110.67 | 52.92 | 0.50 |
| 1 | 0 | Black | 15 | 1 | B | 8 | 88.09 | 81.42 | 108.36 | 31.86 | 2.07 |
| 1 | 1 | Black | 15 | 1 | B | 8 | 88.33 | 79.78 | 112.20 | 44.56 | 2.07 |
| 1 | 0 | Blue | 15 | 1 | B | 8 | 97.65 | 81.94 | 120.58 | 51.05 | 2.07 |
| 1 | 1 | Blue | 15 | 1 | B | 8 | 89.16 | 77.86 | 111.89 | 49.44 | 0.71 |
| 1 | 0 | Black | 15 | 1 | R | 8 | 96.44 | 96.40 | 115.25 | 30.84 | 2.62 |
| 1 | 1 | Black | 15 | 1 | R | 8 | 87.56 | 80.28 | 114.24 | 40.99 | 2.62 |
| 1 | 0 | Blue | 15 | 1 | R | 8 | 98.98 | 91.20 | 123.32 | 48.89 | 2.62 |
| 1 | 1 | Blue | 15 | 1 | R | 8 | 90.70 | 84.28 | 116.55 | 42.94 | 2.07 |
| 1 | 0 | Black | 15 | 2 | B | 8 | 90.82 | 80.07 | 109.08 | 48.02 | 0.73 |
| 1 | 1 | Black | 15 | 2 | B | 8 | 92.23 | 83.94 | 117.32 | 48.33 | 0.73 |
| 1 | 0 | Blue | 15 | 2 | B | 8 | 102.23 | 86.88 | 128.06 | 67.78 | 0.73 |
| 1 | 1 | Blue | 15 | 2 | B | 8 | 94.96 | 84.40 | 120.83 | 53.88 | 2.62 |
| 1 | 0 | Black | 15 | 2 | R | 8 | 82.02 | 66.74 | 93.96 | 50.36 | 0.71 |
| 1 | 1 | Black | 15 | 2 | R | 8 | 92.61 | 91.60 | 127.95 | 49.77 | 0.71 |
| 1 | 0 | Blue | 15 | 2 | R | 8 | 93.68 | 70.66 | 114.31 | 67.32 | 0.73 |
| 1 | 1 | Blue | 15 | 2 | R | 8 | 88.99 | 75.21 | 109.76 | 53.99 | 0.71 |
| 2 | 0 | Black | 5 | 1 | B | 8 | 71.12 | 58.13 | 83.89 | 30.74 | 0.19 |
| 2 | 1 | Black | 5 | 1 | B | 8 | 92.43 | 80.25 | 109.86 | 52.98 | 0.19 |
| 2 | 0 | Blue | 5 | 1 | B | 8 | 88.08 | 60.62 | 108.03 | 61.81 | 0.19 |
| 2 | 1 | Blue | 5 | 1 | B | 8 | 77.51 | 53.60 | 87.26 | 52.52 | 0.19 |
| 2 | 0 | Black | 5 | 1 | R | 8 | 78.77 | 62.98 | 99.15 | 50.34 | 0.31 |
| 2 | 1 | Black | 5 | 1 | R | 8 | 91.94 | 81.04 | 114.24 | 55.52 | 0.31 |
| 2 | 0 | Blue | 5 | 1 | R | 8 | 94.35 | 80.52 | 112.15 | 57.93 | 0.31 |
| 2 | 1 | Blue | 5 | 1 | R | 8 | 71.13 | 49.31 | 81.94 | 43.93 | 0.31 |
| 2 | 0 | Black | 5 | 2 | B | 8 | 61.30 | 51.17 | 72.78 | 30.21 | 0.10 |
| 2 | 1 | Black | 5 | 2 | B | 8 | 91.65 | 79.05 | 107.22 | 54.84 | 0.10 |
| 2 | 0 | Blue | 5 | 2 | B | 8 | 76.94 | 65.56 | 81.44 | 52.72 | 0.10 |
| 2 | 1 | Blue | 5 | 2 | B | 8 | 73.05 | 48.82 | 81.13 | 49.72 | 0.10 |
| 2 | 0 | Black | 5 | 2 | R | 8 | 86.08 | 67.48 | 98.08 | 52.94 | 0.11 |
| 2 | 1 | Black | 5 | 2 | R | 8 | 107.21 | 96.70 | 130.48 | 51.15 | 0.11 |
| 2 | 0 | Blue | 5 | 2 | R | 8 | 102.97 | 73.58 | 122.40 | 72.69 | 0.11 |
| 2 | 1 | Blue | 5 | 2 | R | 8 | 98.95 | 81.51 | 119.07 | 54.01 | 0.11 |
| 2 | 0 | Black | 8 | 1 | B | 8 | 93.28 | 77.58 | 114.72 | 50.86 | 0.45 |
| 2 | 1 | Black | 8 | 1 | B | 8 | 90.13 | 79.65 | 109.80 | 50.10 | 0.45 |
| 2 | 0 | Blue | 8 | 1 | B | 8 | 84.32 | 59.81 | 99.92 | 56.66 | 0.45 |
| 2 | 1 | Blue | 8 | 1 | B | 8 | 82.36 | 69.64 | 102.28 | 47.47 | 0.45 |
| 2 | 0 | Black | 8 | 1 | R | 8 | 81.42 | 68.05 | 102.38 | 46.67 | 0.57 |
| 2 | 1 | Black | 8 | 1 | R | 8 | 93.51 | 84.16 | 116.78 | 55.30 | 0.57 |
| 2 | 0 | Blue | 8 | 1 | R | 8 | 98.79 | 81.62 | 119.47 | 61.26 | 0.57 |
| 2 | 1 | Blue | 8 | 1 | R | 8 | 77.24 | 66.09 | 99.46 | 45.43 | 0.57 |
| 2 | 0 | Black | 8 | 2 | B | 8 | 73.62 | 54.04 | 84.08 | 46.89 | 0.17 |
| 2 | 1 | Black | 8 | 2 | B | 8 | 97.29 | 81.01 | 109.78 | 54.66 | 0.17 |
| 2 | 0 | Blue | 8 | 2 | B | 8 | 99.79 | 77.93 | 114.91 | 67.16 | 0.17 |
| 2 | 1 | Blue | 8 | 2 | B | 8 | 78.14 | 56.70 | 90.96 | 52.88 | 0.17 |
| 2 | 0 | Black | 8 | 2 | R | 8 | 77.96 | 69.08 | 95.53 | 38.62 | 0.20 |
| 2 | 1 | Black | 8 | 2 | R | 8 | 98.69 | 90.62 | 122.61 | 51.42 | 0.20 |
| 2 | 0 | Blue | 8 | 2 | R | 8 | 99.53 | 92.10 | 117.93 | 50.75 | 0.20 |
| 2 | 1 | Blue | 8 | 2 | R | 8 | 87.39 | 71.20 | 107.99 | 48.81 | 0.20 |
| 2 | 0 | Black | 10 | 1 | B | 8 | 69.36 | 59.50 | 84.07 | 27.23 | 0.81 |
| 2 | 1 | Black | 10 | 1 | B | 8 | 89.81 | 84.86 | 119.34 | 49.19 | 0.81 |
| 2 | 0 | Blue | 10 | 1 | B | 8 | 68.79 | 51.82 | 79.14 | 30.28 | 0.81 |
| 2 | 1 | Blue | 10 | 1 | B | 8 | 77.85 | 66.06 | 96.67 | 43.05 | 0.81 |
| 2 | 0 | Black | 10 | 1 | R | 8 | 73.20 | 56.94 | 90.23 | 32.32 | 0.89 |
| 2 | 1 | Black | 10 | 1 | R | 8 | 94.69 | 90.59 | 125.43 | 47.68 | 0.89 |
| 2 | 0 | Blue | 10 | 1 | R | 8 | 83.22 | 62.40 | 102.55 | 50.62 | 0.89 |
| 2 | 1 | Blue | 10 | 1 | R | 8 | 82.16 | 69.71 | 103.36 | 40.22 | 0.89 |
| 2 | 0 | Black | 10 | 2 | B | 8 | 77.87 | 60.78 | 91.42 | 44.54 | 0.28 |
| 2 | 1 | Black | 10 | 2 | B | 8 | 103.80 | 98.29 | 135.12 | 54.03 | 0.28 |
| 2 | 0 | Blue | 10 | 2 | B | 8 | 92.57 | 67.24 | 109.12 | 70.68 | 0.28 |
| 2 | 1 | Blue | 10 | 2 | B | 8 | 82.50 | 66.07 | 99.01 | 47.61 | 0.28 |
| 2 | 0 | Black | 10 | 2 | R | 8 | 84.00 | 73.87 | 103.42 | 53.32 | 0.28 |
| 2 | 1 | Black | 10 | 2 | R | 8 | 102.71 | 101.50 | 137.50 | 57.54 | 0.28 |
| 2 | 0 | Blue | 10 | 2 | R | 8 | 101.47 | 82.59 | 123.92 | 72.95 | 0.28 |
| 2 | 1 | Blue | 10 | 2 | R | 8 | 87.77 | 76.16 | 112.02 | 49.33 | 0.28 |
| 2 | 0 | Black | 12 | 1 | B | 8 | 71.70 | 57.01 | 87.18 | 29.77 | 1.26 |
| 2 | 1 | Black | 12 | 1 | B | 8 | 88.21 | 75.58 | 105.30 | 47.27 | 1.26 |
| 2 | 0 | Blue | 12 | 1 | B | 8 | 92.44 | 72.46 | 113.41 | 56.57 | 1.26 |
| 2 | 1 | Blue | 12 | 1 | B | 8 | 92.14 | 78.16 | 112.20 | 53.31 | 1.26 |
| 2 | 0 | Black | 12 | 1 | R | 8 | 87.41 | 73.78 | 102.01 | 48.81 | 1.01 |
| 2 | 1 | Black | 12 | 1 | R | 8 | 93.21 | 83.75 | 116.30 | 48.49 | 1.01 |
| 2 | 0 | Blue | 12 | 1 | R | 8 | 86.60 | 76.64 | 102.62 | 38.73 | 1.01 |
| 2 | 1 | Blue | 12 | 1 | R | 8 | 90.49 | 80.15 | 112.98 | 47.54 | 1.01 |
| 2 | 0 | Black | 12 | 2 | B | 8 | 87.76 | 73.93 | 104.22 | 51.55 | 0.39 |
| 2 | 1 | Black | 12 | 2 | B | 8 | 92.21 | 85.31 | 119.15 | 51.02 | 0.39 |
| 2 | 0 | Blue | 12 | 2 | B | 8 | 90.18 | 65.68 | 109.98 | 65.08 | 0.39 |
| 2 | 1 | Blue | 12 | 2 | B | 8 | 82.61 | 68.99 | 102.91 | 49.49 | 0.39 |
| 2 | 0 | Black | 12 | 2 | R | 8 | 75.06 | 65.24 | 89.63 | 33.73 | 0.40 |
| 2 | 1 | Black | 12 | 2 | R | 8 | 103.09 | 96.51 | 131.41 | 54.61 | 0.40 |
| 2 | 0 | Blue | 12 | 2 | R | 8 | 95.87 | 81.49 | 117.13 | 54.73 | 0.40 |
| 2 | 1 | Blue | 12 | 2 | R | 8 | 92.13 | 80.75 | 116.40 | 54.41 | 0.40 |
| 2 | 0 | Black | 15 | 1 | B | 8 | 78.23 | 61.52 | 92.00 | 39.80 | 1.56 |
| 2 | 1 | Black | 15 | 1 | B | 8 | 89.89 | 73.47 | 103.03 | 45.16 | 1.56 |
| 2 | 0 | Blue | 15 | 1 | B | 8 | 76.46 | 52.91 | 88.63 | 47.52 | 1.56 |
| 2 | 1 | Blue | 15 | 1 | B | 8 | 87.41 | 70.44 | 101.84 | 47.87 | 1.56 |
| 2 | 0 | Black | 15 | 1 | R | 8 | 79.49 | 62.65 | 94.72 | 35.03 | 1.71 |
| 2 | 1 | Black | 15 | 1 | R | 8 | 88.47 | 79.00 | 110.69 | 47.51 | 1.71 |
| 2 | 0 | Blue | 15 | 1 | R | 8 | 89.34 | 65.77 | 108.01 | 52.32 | 1.71 |
| 2 | 1 | Blue | 15 | 1 | R | 8 | 89.23 | 79.42 | 107.03 | 41.26 | 1.71 |
| 2 | 0 | Black | 15 | 2 | B | 8 | 85.82 | 69.93 | 101.27 | 55.18 | 0.68 |
| 2 | 1 | Black | 15 | 2 | B | 8 | 92.05 | 89.48 | 125.42 | 53.27 | 0.68 |
| 2 | 0 | Blue | 15 | 2 | B | 8 | 96.33 | 71.14 | 115.47 | 69.05 | 0.68 |
| 2 | 1 | Blue | 15 | 2 | B | 8 | 82.06 | 63.46 | 95.81 | 44.84 | 0.68 |
| 2 | 0 | Black | 15 | 2 | R | 8 | 80.31 | 65.84 | 86.99 | 48.75 | 0.82 |
| 2 | 1 | Black | 15 | 2 | R | 8 | 101.79 | 99.51 | 135.03 | 55.98 | 0.82 |
| 2 | 0 | Blue | 15 | 2 | R | 8 | 100.00 | 82.16 | 120.96 | 67.90 | 0.82 |
| 2 | 1 | Blue | 15 | 2 | R | 8 | 96.21 | 90.82 | 128.27 | 58.08 | 0.82 |
| 3 | 0 | Black | 5 | 1 | B | 8 | 67.01 | 54.99 | 78.54 | 33.43 | 0.21 |
| 3 | 1 | Black | 5 | 1 | B | 8 | 86.65 | 81.87 | 114.46 | 45.00 | 0.21 |
| 3 | 0 | Blue | 5 | 1 | B | 8 | 79.17 | 61.76 | 95.34 | 49.14 | 0.21 |
| 3 | 1 | Blue | 5 | 1 | B | 8 | 75.47 | 62.38 | 94.62 | 42.68 | 0.21 |
| 3 | 0 | Black | 5 | 1 | R | 8 | 75.57 | 57.76 | 82.32 | 42.66 | 0.25 |
| 3 | 1 | Black | 5 | 1 | R | 8 | 99.87 | 85.69 | 117.56 | 51.51 | 0.25 |
| 3 | 0 | Blue | 5 | 1 | R | 8 | 76.90 | 55.50 | 89.93 | 46.50 | 0.25 |
| 3 | 1 | Blue | 5 | 1 | R | 8 | 84.48 | 70.83 | 100.96 | 44.92 | 0.25 |
| 3 | 0 | Black | 5 | 2 | B | 8 | 69.55 | 54.62 | 83.34 | 34.74 | 0.10 |
| 3 | 1 | Black | 5 | 2 | B | 8 | 94.57 | 87.78 | 119.39 | 51.18 | 0.10 |
| 3 | 0 | Blue | 5 | 2 | B | 8 | 75.14 | 45.92 | 91.54 | 59.85 | 0.10 |
| 3 | 1 | Blue | 5 | 2 | B | 8 | 76.67 | 55.95 | 90.58 | 47.87 | 0.10 |
| 3 | 0 | Black | 5 | 2 | R | 8 | 71.92 | 58.91 | 85.27 | 37.48 | 0.11 |
| 3 | 1 | Black | 5 | 2 | R | 8 | 112.53 | 113.68 | 154.45 | 59.79 | 0.11 |
| 3 | 0 | Blue | 5 | 2 | R | 8 | 84.70 | 65.04 | 102.46 | 52.88 | 0.11 |
| 3 | 1 | Blue | 5 | 2 | R | 8 | 90.99 | 78.24 | 116.66 | 43.99 | 0.11 |
| 3 | 0 | Black | 8 | 1 | B | 8 | 84.26 | 66.82 | 101.16 | 46.17 | 0.52 |
| 3 | 1 | Black | 8 | 1 | B | 8 | 96.94 | 91.14 | 127.13 | 54.52 | 0.52 |
| 3 | 0 | Blue | 8 | 1 | B | 8 | 85.80 | 63.99 | 104.62 | 52.44 | 0.52 |
| 3 | 1 | Blue | 8 | 1 | B | 8 | 79.36 | 64.21 | 97.64 | 46.01 | 0.52 |
| 3 | 0 | Black | 8 | 1 | R | 8 | 76.51 | 72.67 | 89.26 | 20.03 | 0.52 |
| 3 | 1 | Black | 8 | 1 | R | 8 | 98.64 | 93.51 | 128.33 | 54.12 | 0.52 |
| 3 | 0 | Blue | 8 | 1 | R | 8 | 73.74 | 57.41 | 90.04 | 40.24 | 0.52 |
| 3 | 1 | Blue | 8 | 1 | R | 8 | 78.82 | 67.68 | 100.80 | 42.53 | 0.52 |
| 3 | 0 | Black | 8 | 2 | B | 8 | 82.86 | 67.47 | 100.27 | 56.14 | 0.15 |
| 3 | 1 | Black | 8 | 2 | B | 8 | 107.71 | 95.39 | 129.75 | 57.57 | 0.15 |
| 3 | 0 | Blue | 8 | 2 | B | 8 | 93.23 | 77.05 | 110.30 | 55.29 | 0.15 |
| 3 | 1 | Blue | 8 | 2 | B | 8 | 84.97 | 70.60 | 105.80 | 49.80 | 0.15 |
| 3 | 0 | Black | 8 | 2 | R | 8 | 78.62 | 63.23 | 93.81 | 46.47 | 0.15 |
| 3 | 1 | Black | 8 | 2 | R | 8 | 102.99 | 94.79 | 128.00 | 52.23 | 0.15 |
| 3 | 0 | Blue | 8 | 2 | R | 8 | 100.90 | 92.82 | 119.99 | 49.81 | 0.15 |
| 3 | 1 | Blue | 8 | 2 | R | 8 | 84.28 | 71.73 | 106.63 | 48.37 | 0.15 |
| 3 | 0 | Black | 10 | 1 | B | 8 | 64.18 | 45.21 | 71.42 | 35.52 | 0.60 |
| 3 | 1 | Black | 10 | 1 | B | 8 | 93.89 | 82.88 | 115.80 | 57.89 | 0.60 |
| 3 | 0 | Blue | 10 | 1 | B | 8 | 69.19 | 49.68 | 80.98 | 47.97 | 0.60 |
| 3 | 1 | Blue | 10 | 1 | B | 8 | 74.52 | 58.76 | 87.45 | 45.24 | 0.60 |
| 3 | 0 | Black | 10 | 1 | R | 8 | 64.92 | 54.06 | 78.03 | 24.26 | 0.71 |
| 3 | 1 | Black | 10 | 1 | R | 8 | 91.77 | 84.85 | 118.14 | 51.72 | 0.71 |
| 3 | 0 | Blue | 10 | 1 | R | 8 | 86.34 | 72.75 | 101.98 | 37.05 | 0.71 |
| 3 | 1 | Blue | 10 | 1 | R | 8 | 87.68 | 71.24 | 104.66 | 47.75 | 0.71 |
| 3 | 0 | Black | 10 | 2 | B | 8 | 69.99 | 54.03 | 81.76 | 42.39 | 0.22 |
| 3 | 1 | Black | 10 | 2 | B | 8 | 99.18 | 89.83 | 122.07 | 54.34 | 0.22 |
| 3 | 0 | Blue | 10 | 2 | B | 8 | 75.77 | 48.72 | 88.80 | 58.08 | 0.22 |
| 3 | 1 | Blue | 10 | 2 | B | 8 | 83.18 | 62.68 | 95.84 | 53.80 | 0.22 |
| 3 | 0 | Black | 10 | 2 | R | 8 | 80.02 | 62.81 | 93.12 | 49.61 | 0.24 |
| 3 | 1 | Black | 10 | 2 | R | 8 | 100.38 | 96.98 | 134.69 | 52.28 | 0.24 |
| 3 | 0 | Blue | 10 | 2 | R | 8 | 92.17 | 72.83 | 110.20 | 58.94 | 0.24 |
| 3 | 1 | Blue | 10 | 2 | R | 8 | 85.89 | 69.55 | 103.91 | 50.88 | 0.24 |
| 3 | 0 | Black | 12 | 1 | B | 8 | 73.36 | 59.28 | 81.42 | 41.08 | 1.02 |
| 3 | 1 | Black | 12 | 1 | B | 8 | 90.01 | 80.33 | 111.75 | 51.01 | 1.02 |
| 3 | 0 | Blue | 12 | 1 | B | 8 | 78.54 | 56.41 | 95.42 | 50.09 | 1.02 |
| 3 | 1 | Blue | 12 | 1 | B | 8 | 79.74 | 65.55 | 96.78 | 48.34 | 1.02 |
| 3 | 0 | Black | 12 | 1 | R | 8 | 74.90 | 64.88 | 90.70 | 32.48 | 1.14 |
| 3 | 1 | Black | 12 | 1 | R | 8 | 93.38 | 85.19 | 118.86 | 53.97 | 1.14 |
| 3 | 0 | Blue | 12 | 1 | R | 8 | 87.93 | 65.24 | 108.22 | 59.97 | 1.14 |
| 3 | 1 | Blue | 12 | 1 | R | 8 | 88.27 | 72.00 | 105.41 | 49.40 | 1.14 |
| 3 | 0 | Black | 12 | 2 | B | 8 | 73.19 | 60.63 | 83.11 | 44.65 | 0.39 |
| 3 | 1 | Black | 12 | 2 | B | 8 | 99.55 | 91.40 | 125.49 | 56.67 | 0.39 |
| 3 | 0 | Blue | 12 | 2 | B | 8 | 85.27 | 66.30 | 98.92 | 57.95 | 0.39 |
| 3 | 1 | Blue | 12 | 2 | B | 8 | 82.83 | 69.32 | 104.20 | 47.47 | 0.39 |
| 3 | 0 | Black | 12 | 2 | R | 8 | 70.52 | 56.56 | 78.32 | 44.00 | 0.38 |
| 3 | 1 | Black | 12 | 2 | R | 8 | 101.82 | 98.40 | 136.01 | 57.00 | 0.38 |
| 3 | 0 | Blue | 12 | 2 | R | 8 | 73.64 | 57.29 | 90.30 | 41.73 | 0.38 |
| 3 | 1 | Blue | 12 | 2 | R | 8 | 82.60 | 63.56 | 97.12 | 46.40 | 0.38 |
| 3 | 0 | Black | 15 | 1 | B | 8 | 80.85 | 71.38 | 96.53 | 31.66 | 1.51 |
| 3 | 1 | Black | 15 | 1 | B | 8 | 88.35 | 75.92 | 107.04 | 45.93 | 1.51 |
| 3 | 0 | Blue | 15 | 1 | B | 8 | 87.52 | 68.09 | 104.63 | 50.08 | 1.51 |
| 3 | 1 | Blue | 15 | 1 | B | 8 | 88.14 | 75.83 | 108.71 | 47.65 | 1.51 |
| 3 | 0 | Black | 15 | 1 | R | 8 | 61.56 | 51.06 | 73.17 | 18.78 | 1.60 |
| 3 | 1 | Black | 15 | 1 | R | 8 | 87.48 | 71.90 | 101.46 | 42.22 | 1.60 |
| 3 | 0 | Blue | 15 | 1 | R | 8 | 74.82 | 56.52 | 88.89 | 35.78 | 1.60 |
| 3 | 1 | Blue | 15 | 1 | R | 8 | 96.87 | 80.39 | 115.28 | 51.85 | 1.60 |
| 3 | 0 | Black | 15 | 2 | B | 8 | 74.55 | 60.22 | 88.65 | 39.99 | 0.67 |
| 3 | 1 | Black | 15 | 2 | B | 8 | 94.55 | 84.19 | 116.91 | 49.75 | 0.67 |
| 3 | 0 | Blue | 15 | 2 | B | 8 | 89.10 | 64.13 | 107.72 | 62.71 | 0.67 |
| 3 | 1 | Blue | 15 | 2 | B | 8 | 92.83 | 82.05 | 117.84 | 54.76 | 0.67 |
| 3 | 0 | Black | 15 | 2 | R | 8 | 68.53 | 53.59 | 77.54 | 42.92 | 0.50 |
| 3 | 1 | Black | 15 | 2 | R | 8 | 95.95 | 90.37 | 123.12 | 53.43 | 0.50 |
| 3 | 0 | Blue | 15 | 2 | R | 8 | 90.16 | 72.01 | 109.12 | 68.51 | 0.50 |
| 3 | 1 | Blue | 15 | 2 | R | 8 | 87.55 | 75.09 | 109.95 | 54.38 | 0.50 |
| 4 | 0 | Black | 5 | 1 | B | 8 | 62.38 | 50.08 | 69.14 | 24.56 | 0.26 |
| 4 | 1 | Black | 5 | 1 | B | 8 | 94.93 | 86.94 | 118.99 | 49.77 | 0.26 |
| 4 | 0 | Blue | 5 | 1 | B | 8 | 69.71 | 53.17 | 83.21 | 39.21 | 0.26 |
| 4 | 1 | Blue | 5 | 1 | B | 8 | 76.67 | 58.22 | 90.98 | 42.96 | 0.26 |
| 4 | 0 | Black | 5 | 1 | R | 8 | 59.93 | 50.22 | 70.18 | 22.35 | 0.25 |
| 4 | 1 | Black | 5 | 1 | R | 8 | 95.58 | 91.74 | 124.55 | 51.76 | 0.25 |
| 4 | 0 | Blue | 5 | 1 | R | 8 | 77.96 | 58.57 | 92.91 | 45.37 | 0.25 |
| 4 | 1 | Blue | 5 | 1 | R | 8 | 79.53 | 58.25 | 92.00 | 46.15 | 0.25 |
| 4 | 0 | Black | 5 | 2 | B | 8 | 76.21 | 60.68 | 92.52 | 44.00 | 0.09 |
| 4 | 1 | Black | 5 | 2 | B | 8 | 111.92 | 110.33 | 148.49 | 58.04 | 0.09 |
| 4 | 0 | Blue | 5 | 2 | B | 8 | 76.72 | 52.28 | 88.13 | 55.45 | 0.09 |
| 4 | 1 | Blue | 5 | 2 | B | 8 | 88.79 | 72.87 | 108.13 | 50.25 | 0.09 |
| 4 | 0 | Black | 5 | 2 | R | 8 | 64.15 | 45.85 | 68.23 | 36.00 | 0.10 |
| 4 | 1 | Black | 5 | 2 | R | 8 | 103.45 | 98.99 | 135.35 | 54.44 | 0.10 |
| 4 | 0 | Blue | 5 | 2 | R | 8 | 74.06 | 53.18 | 85.01 | 46.55 | 0.10 |
| 4 | 1 | Blue | 5 | 2 | R | 8 | 84.98 | 71.91 | 99.25 | 48.95 | 0.10 |
| 4 | 0 | Black | 8 | 1 | B | 8 | 71.88 | 54.92 | 81.94 | 37.40 | 0.54 |
| 4 | 1 | Black | 8 | 1 | B | 8 | 97.51 | 85.65 | 117.01 | 51.69 | 0.54 |
| 4 | 0 | Blue | 8 | 1 | B | 8 | 74.75 | 62.76 | 85.10 | 33.13 | 0.54 |
| 4 | 1 | Blue | 8 | 1 | B | 8 | 80.20 | 60.84 | 93.28 | 42.78 | 0.54 |
| 4 | 0 | Black | 8 | 1 | R | 8 | 69.48 | 58.28 | 81.91 | 26.97 | 0.57 |
| 4 | 1 | Black | 8 | 1 | R | 8 | 95.47 | 86.63 | 119.68 | 47.07 | 0.57 |
| 4 | 0 | Blue | 8 | 1 | R | 8 | 80.67 | 56.07 | 92.83 | 51.73 | 0.57 |
| 4 | 1 | Blue | 8 | 1 | R | 8 | 90.55 | 74.55 | 107.77 | 47.07 | 0.57 |
| 4 | 0 | Black | 8 | 2 | B | 8 | 73.76 | 60.70 | 87.92 | 39.60 | 0.16 |
| 4 | 1 | Black | 8 | 2 | B | 8 | 108.99 | 100.01 | 134.84 | 54.96 | 0.16 |
| 4 | 0 | Blue | 8 | 2 | B | 8 | 73.13 | 49.00 | 85.93 | 51.12 | 0.16 |
| 4 | 1 | Blue | 8 | 2 | B | 8 | 87.90 | 76.29 | 111.60 | 50.70 | 0.16 |
| 4 | 0 | Black | 8 | 2 | R | 8 | 80.72 | 65.79 | 97.16 | 41.20 | 0.14 |
| 4 | 1 | Black | 8 | 2 | R | 8 | 95.51 | 88.29 | 120.78 | 50.83 | 0.14 |
| 4 | 0 | Blue | 8 | 2 | R | 8 | 83.71 | 76.80 | 100.38 | 37.28 | 0.14 |
| 4 | 1 | Blue | 8 | 2 | R | 8 | 81.63 | 72.86 | 103.37 | 42.52 | 0.14 |
| 4 | 0 | Black | 10 | 1 | B | 8 | 61.06 | 51.28 | 72.79 | 20.60 | 0.84 |
| 4 | 1 | Black | 10 | 1 | B | 8 | 94.04 | 92.53 | 127.44 | 46.73 | 0.84 |
| 4 | 0 | Blue | 10 | 1 | B | 8 | 79.32 | 71.71 | 96.48 | 32.27 | 0.84 |
| 4 | 1 | Blue | 10 | 1 | B | 8 | 91.93 | 86.09 | 120.91 | 45.17 | 0.84 |
| 4 | 0 | Black | 10 | 1 | R | 8 | 64.55 | 54.41 | 75.89 | 25.87 | 0.87 |
| 4 | 1 | Black | 10 | 1 | R | 8 | 96.60 | 87.05 | 115.17 | 40.70 | 0.87 |
| 4 | 0 | Blue | 10 | 1 | R | 8 | 80.64 | 66.69 | 96.14 | 36.37 | 0.87 |
| 4 | 1 | Blue | 10 | 1 | R | 8 | 87.23 | 73.93 | 107.26 | 43.65 | 0.87 |
| 4 | 0 | Black | 10 | 2 | B | 8 | 68.93 | 54.01 | 79.32 | 32.91 | 0.29 |
| 4 | 1 | Black | 10 | 2 | B | 8 | 101.09 | 95.50 | 128.26 | 49.94 | 0.29 |
| 4 | 0 | Blue | 10 | 2 | B | 8 | 77.31 | 56.85 | 92.28 | 53.12 | 0.29 |
| 4 | 1 | Blue | 10 | 2 | B | 8 | 89.12 | 75.78 | 109.94 | 53.51 | 0.29 |
| 4 | 0 | Black | 10 | 2 | R | 8 | 75.86 | 62.51 | 89.24 | 42.24 | 0.25 |
| 4 | 1 | Black | 10 | 2 | R | 8 | 112.70 | 117.76 | 156.64 | 64.40 | 0.25 |
| 4 | 0 | Blue | 10 | 2 | R | 8 | 90.22 | 81.09 | 104.74 | 45.17 | 0.25 |
| 4 | 1 | Blue | 10 | 2 | R | 8 | 96.28 | 86.85 | 122.22 | 56.28 | 0.25 |
| 4 | 0 | Black | 12 | 1 | B | 8 | 67.00 | 57.42 | 79.00 | 28.85 | 1.18 |
| 4 | 1 | Black | 12 | 1 | B | 8 | 95.94 | 92.56 | 127.73 | 49.97 | 1.18 |
| 4 | 0 | Blue | 12 | 1 | B | 8 | 85.46 | 73.76 | 101.07 | 42.50 | 1.18 |
| 4 | 1 | Blue | 12 | 1 | B | 8 | 96.74 | 80.37 | 114.24 | 49.07 | 1.18 |
| 4 | 0 | Black | 12 | 1 | R | 8 | 57.80 | 46.14 | 66.37 | 17.05 | 1.09 |
| 4 | 1 | Black | 12 | 1 | R | 8 | 91.41 | 76.07 | 106.63 | 42.45 | 1.09 |
| 4 | 0 | Blue | 12 | 1 | R | 8 | 81.63 | 74.75 | 92.36 | 24.41 | 1.09 |
| 4 | 1 | Blue | 12 | 1 | R | 8 | 87.65 | 71.84 | 104.19 | 41.01 | 1.09 |
| 4 | 0 | Black | 12 | 2 | B | 8 | 70.30 | 50.44 | 75.55 | 35.60 | 0.33 |
| 4 | 1 | Black | 12 | 2 | B | 8 | 98.47 | 85.71 | 117.06 | 48.23 | 0.33 |
| 4 | 0 | Blue | 12 | 2 | B | 8 | 82.84 | 61.26 | 97.88 | 58.24 | 0.33 |
| 4 | 1 | Blue | 12 | 2 | B | 8 | 93.40 | 73.52 | 107.26 | 53.12 | 0.33 |
| 4 | 0 | Black | 12 | 2 | R | 8 | 75.06 | 64.91 | 87.75 | 36.15 | 0.36 |
| 4 | 1 | Black | 12 | 2 | R | 8 | 100.20 | 95.03 | 128.99 | 57.36 | 0.36 |
| 4 | 0 | Blue | 12 | 2 | R | 8 | 82.14 | 66.99 | 97.74 | 46.03 | 0.36 |
| 4 | 1 | Blue | 12 | 2 | R | 8 | 89.76 | 82.79 | 116.53 | 54.56 | 0.36 |
| 4 | 0 | Black | 15 | 1 | B | 8 | 74.11 | 65.00 | 88.83 | 23.98 | 1.59 |
| 4 | 1 | Black | 15 | 1 | B | 8 | 93.82 | 83.86 | 114.67 | 45.68 | 1.59 |
| 4 | 0 | Blue | 15 | 1 | B | 8 | 80.36 | 64.21 | 94.99 | 38.99 | 1.59 |
| 4 | 1 | Blue | 15 | 1 | B | 8 | 90.96 | 76.48 | 108.06 | 48.46 | 1.59 |
| 4 | 0 | Black | 15 | 1 | R | 8 | 57.25 | 47.71 | 68.57 | 17.72 | 1.64 |
| 4 | 1 | Black | 15 | 1 | R | 8 | 97.79 | 95.97 | 130.96 | 51.09 | 1.64 |
| 4 | 0 | Blue | 15 | 1 | R | 8 | 85.23 | 76.88 | 105.77 | 39.60 | 1.64 |
| 4 | 1 | Blue | 15 | 1 | R | 8 | 95.67 | 90.86 | 125.43 | 49.72 | 1.64 |
| 4 | 0 | Black | 15 | 2 | B | 8 | 75.03 | 62.68 | 86.95 | 37.65 | 0.62 |
| 4 | 1 | Black | 15 | 2 | B | 8 | 94.34 | 91.74 | 127.91 | 49.32 | 0.62 |
| 4 | 0 | Blue | 15 | 2 | B | 8 | 84.18 | 67.16 | 95.71 | 50.65 | 0.62 |
| 4 | 1 | Blue | 15 | 2 | B | 8 | 95.91 | 84.65 | 119.45 | 52.90 | 0.62 |
| 4 | 0 | Black | 15 | 2 | R | 8 | 64.45 | 54.96 | 75.59 | 20.19 | 0.62 |
| 4 | 1 | Black | 15 | 2 | R | 8 | 100.25 | 98.07 | 133.27 | 50.31 | 0.62 |
| 4 | 0 | Blue | 15 | 2 | R | 8 | 88.02 | 68.16 | 103.54 | 61.36 | 0.62 |
| 4 | 1 | Blue | 15 | 2 | R | 8 | 92.97 | 84.37 | 119.18 | 49.92 | 0.62 |
| 5 | 0 | Black | 5 | 1 | B | 8 | 62.94 | 51.75 | 73.33 | 29.55 | 0.24 |
| 5 | 1 | Black | 5 | 1 | B | 8 | 95.13 | 81.32 | 113.08 | 51.22 | 0.24 |
| 5 | 0 | Blue | 5 | 1 | B | 8 | 68.47 | 51.29 | 80.44 | 39.65 | 0.24 |
| 5 | 1 | Blue | 5 | 1 | B | 8 | 80.61 | 63.66 | 92.88 | 41.82 | 0.24 |
| 5 | 0 | Black | 5 | 1 | R | 8 | 57.98 | 44.87 | 62.58 | 21.03 | 0.25 |
| 5 | 1 | Black | 5 | 1 | R | 8 | 108.75 | 104.90 | 142.99 | 59.39 | 0.25 |
| 5 | 0 | Blue | 5 | 1 | R | 8 | 72.09 | 56.67 | 85.87 | 37.92 | 0.25 |
| 5 | 1 | Blue | 5 | 1 | R | 8 | 81.73 | 72.71 | 102.85 | 34.46 | 0.25 |
| 5 | 0 | Black | 5 | 2 | B | 8 | 62.58 | 48.74 | 70.79 | 30.07 | 0.10 |
| 5 | 1 | Black | 5 | 2 | B | 8 | 120.42 | 114.90 | 154.03 | 71.80 | 0.10 |
| 5 | 0 | Blue | 5 | 2 | B | 8 | 67.88 | 51.02 | 80.12 | 38.48 | 0.10 |
| 5 | 1 | Blue | 5 | 2 | B | 8 | 91.57 | 71.40 | 109.47 | 55.53 | 0.10 |
| 5 | 0 | Black | 5 | 2 | R | 8 | 65.85 | 54.66 | 77.16 | 33.46 | 0.11 |
| 5 | 1 | Black | 5 | 2 | R | 8 | 109.72 | 95.75 | 131.01 | 63.91 | 0.11 |
| 5 | 0 | Blue | 5 | 2 | R | 8 | 79.46 | 57.18 | 92.15 | 55.20 | 0.11 |
| 5 | 1 | Blue | 5 | 2 | R | 8 | 83.07 | 61.96 | 97.78 | 54.77 | 0.11 |
| 5 | 0 | Black | 8 | 1 | B | 8 | 70.37 | 59.31 | 82.58 | 31.18 | 0.57 |
| 5 | 1 | Black | 8 | 1 | B | 8 | 95.58 | 81.71 | 114.70 | 54.05 | 0.57 |
| 5 | 0 | Blue | 8 | 1 | B | 8 | 68.54 | 52.09 | 81.14 | 37.67 | 0.57 |
| 5 | 1 | Blue | 8 | 1 | B | 8 | 78.04 | 63.27 | 92.77 | 38.29 | 0.57 |
| 5 | 0 | Black | 8 | 1 | R | 8 | 52.59 | 41.89 | 60.77 | 20.09 | 0.48 |
| 5 | 1 | Black | 8 | 1 | R | 8 | 102.12 | 86.11 | 118.77 | 57.34 | 0.48 |
| 5 | 0 | Blue | 8 | 1 | R | 8 | 68.62 | 52.28 | 81.24 | 35.62 | 0.48 |
| 5 | 1 | Blue | 8 | 1 | R | 8 | 79.73 | 59.51 | 91.02 | 44.29 | 0.48 |
| 5 | 0 | Black | 8 | 2 | B | 8 | 71.29 | 55.74 | 79.28 | 42.97 | 0.19 |
| 5 | 1 | Black | 8 | 2 | B | 8 | 111.60 | 112.24 | 150.13 | 66.09 | 0.19 |
| 5 | 0 | Blue | 8 | 2 | B | 8 | 82.69 | 55.50 | 97.19 | 62.49 | 0.19 |
| 5 | 1 | Blue | 8 | 2 | B | 8 | 84.38 | 70.56 | 104.92 | 50.78 | 0.19 |
| 5 | 0 | Black | 8 | 2 | R | 8 | 70.92 | 63.13 | 84.04 | 28.64 | 0.13 |
| 5 | 1 | Black | 8 | 2 | R | 8 | 102.32 | 102.76 | 140.72 | 61.43 | 0.13 |
| 5 | 0 | Blue | 8 | 2 | R | 8 | 75.09 | 53.90 | 88.60 | 50.27 | 0.13 |
| 5 | 1 | Blue | 8 | 2 | R | 8 | 83.28 | 65.95 | 97.65 | 47.74 | 0.13 |
| 5 | 0 | Black | 10 | 1 | B | 8 | 61.99 | 52.09 | 69.86 | 22.74 | 0.84 |
| 5 | 1 | Black | 10 | 1 | B | 8 | 102.44 | 96.82 | 134.03 | 58.89 | 0.84 |
| 5 | 0 | Blue | 10 | 1 | B | 8 | 76.82 | 72.26 | 89.74 | 31.95 | 0.84 |
| 5 | 1 | Blue | 10 | 1 | B | 8 | 81.42 | 69.93 | 102.13 | 42.98 | 0.84 |
| 5 | 0 | Black | 10 | 1 | R | 8 | 66.48 | 51.70 | 80.85 | 41.88 | 0.69 |
| 5 | 1 | Black | 10 | 1 | R | 8 | 94.65 | 88.98 | 125.01 | 57.77 | 0.69 |
| 5 | 0 | Blue | 10 | 1 | R | 8 | 70.07 | 63.84 | 79.28 | 23.31 | 0.69 |
| 5 | 1 | Blue | 10 | 1 | R | 8 | 80.70 | 64.89 | 95.62 | 49.58 | 0.69 |
| 5 | 0 | Black | 10 | 2 | B | 8 | 67.24 | 50.80 | 76.51 | 38.76 | 0.26 |
| 5 | 1 | Black | 10 | 2 | B | 8 | 107.90 | 98.78 | 134.19 | 64.08 | 0.26 |
| 5 | 0 | Blue | 10 | 2 | B | 8 | 73.18 | 48.50 | 86.53 | 52.60 | 0.26 |
| 5 | 1 | Blue | 10 | 2 | B | 8 | 87.30 | 76.19 | 111.31 | 54.58 | 0.26 |
| 5 | 0 | Black | 10 | 2 | R | 8 | 80.98 | 64.77 | 94.17 | 51.58 | 0.25 |
| 5 | 1 | Black | 10 | 2 | R | 8 | 110.06 | 98.64 | 132.94 | 68.17 | 0.25 |
| 5 | 0 | Blue | 10 | 2 | R | 8 | 76.12 | 49.89 | 89.39 | 59.95 | 0.25 |
| 5 | 1 | Blue | 10 | 2 | R | 8 | 80.58 | 61.39 | 94.44 | 50.15 | 0.25 |
| 5 | 0 | Black | 12 | 1 | B | 8 | 57.49 | 43.76 | 64.20 | 28.24 | 0.90 |
| 5 | 1 | Black | 12 | 1 | B | 8 | 98.45 | 90.66 | 125.25 | 56.09 | 0.90 |
| 5 | 0 | Blue | 12 | 1 | B | 8 | 86.61 | 73.95 | 102.95 | 43.52 | 0.90 |
| 5 | 1 | Blue | 12 | 1 | B | 8 | 83.82 | 68.36 | 99.62 | 49.48 | 0.90 |
| 5 | 0 | Black | 12 | 1 | R | 8 | 65.49 | 47.74 | 74.62 | 37.10 | 1.07 |
| 5 | 1 | Black | 12 | 1 | R | 8 | 94.99 | 82.84 | 116.29 | 58.26 | 1.07 |
| 5 | 0 | Blue | 12 | 1 | R | 8 | 79.33 | 70.19 | 94.67 | 40.02 | 1.07 |
| 5 | 1 | Blue | 12 | 1 | R | 8 | 86.71 | 72.17 | 104.19 | 51.11 | 1.07 |
| 5 | 0 | Black | 12 | 2 | B | 8 | 71.78 | 60.74 | 82.09 | 39.40 | 0.37 |
| 5 | 1 | Black | 12 | 2 | B | 8 | 101.37 | 93.92 | 130.11 | 58.02 | 0.37 |
| 5 | 0 | Blue | 12 | 2 | B | 8 | 79.53 | 55.64 | 95.30 | 57.61 | 0.37 |
| 5 | 1 | Blue | 12 | 2 | B | 8 | 90.02 | 75.02 | 108.61 | 55.66 | 0.37 |
| 5 | 0 | Black | 12 | 2 | R | 8 | 69.43 | 57.96 | 76.46 | 31.96 | 0.34 |
| 5 | 1 | Black | 12 | 2 | R | 8 | 102.69 | 93.42 | 126.11 | 64.08 | 0.34 |
| 5 | 0 | Blue | 12 | 2 | R | 8 | 85.29 | 78.31 | 98.21 | 41.61 | 0.34 |
| 5 | 1 | Blue | 12 | 2 | R | 8 | 86.57 | 74.18 | 107.00 | 57.08 | 0.34 |
| 5 | 0 | Black | 15 | 1 | B | 8 | 59.16 | 49.07 | 67.69 | 17.45 | 1.48 |
| 5 | 1 | Black | 15 | 1 | B | 8 | 98.37 | 84.21 | 116.35 | 49.47 | 1.48 |
| 5 | 0 | Blue | 15 | 1 | B | 8 | 87.24 | 70.53 | 106.34 | 46.51 | 1.48 |
| 5 | 1 | Blue | 15 | 1 | B | 8 | 92.50 | 80.63 | 113.75 | 49.62 | 1.48 |
| 5 | 0 | Black | 15 | 1 | R | 8 | 71.76 | 67.56 | 83.80 | 23.28 | 1.61 |
| 5 | 1 | Black | 15 | 1 | R | 8 | 96.17 | 77.23 | 108.05 | 44.93 | 1.61 |
| 5 | 0 | Blue | 15 | 1 | R | 8 | 80.15 | 74.19 | 89.78 | 33.66 | 1.61 |
| 5 | 1 | Blue | 15 | 1 | R | 8 | 95.57 | 88.32 | 124.04 | 54.87 | 1.61 |
| 5 | 0 | Black | 15 | 2 | B | 8 | 67.90 | 49.76 | 75.78 | 38.56 | 0.67 |
| 5 | 1 | Black | 15 | 2 | B | 8 | 100.63 | 92.25 | 126.52 | 63.00 | 0.67 |
| 5 | 0 | Blue | 15 | 2 | B | 8 | 79.93 | 56.14 | 93.73 | 59.44 | 0.67 |
| 5 | 1 | Blue | 15 | 2 | B | 8 | 93.37 | 82.58 | 117.85 | 60.18 | 0.67 |
| 5 | 0 | Black | 15 | 2 | R | 8 | 81.50 | 67.74 | 94.35 | 50.00 | 0.61 |
| 5 | 1 | Black | 15 | 2 | R | 8 | 104.30 | 99.90 | 134.65 | 61.25 | 0.61 |
| 5 | 0 | Blue | 15 | 2 | R | 8 | 83.11 | 59.62 | 95.10 | 61.20 | 0.61 |
| 5 | 1 | Blue | 15 | 2 | R | 8 | 89.51 | 76.32 | 109.07 | 54.88 | 0.61 |
| 1 | 0 | Black | 5 | 1 | B | 14 | 83.72 | 76.21 | 108.81 | 19.06 | 0.94 |
| 1 | 1 | Black | 5 | 1 | B | 14 | 98.71 | 85.94 | 116.16 | 38.25 | 0.94 |
| 1 | 0 | Blue | 5 | 1 | B | 14 | 93.09 | 84.75 | 125.03 | 31.28 | 2.10 |
| 1 | 1 | Blue | 5 | 1 | B | 14 | 95.36 | 75.51 | 109.33 | 44.38 | 2.10 |
| 1 | 0 | Black | 5 | 1 | R | 14 | 117.05 | 109.32 | 140.26 | 22.03 | 0.77 |
| 1 | 1 | Black | 5 | 1 | R | 14 | 97.81 | 85.61 | 118.32 | 42.06 | 0.77 |
| 1 | 0 | Blue | 5 | 1 | R | 14 | 140.01 | 126.68 | 167.94 | 41.85 | 0.94 |
| 1 | 1 | Blue | 5 | 1 | R | 14 | 91.92 | 84.37 | 109.94 | 34.51 | 0.94 |
| 1 | 0 | Black | 5 | 2 | B | 14 | 80.29 | 73.43 | 105.06 | 26.58 | 0.39 |
| 1 | 1 | Black | 5 | 2 | B | 14 | 86.56 | 72.71 | 101.28 | 45.75 | 0.39 |
| 1 | 0 | Blue | 5 | 2 | B | 14 | 101.74 | 80.37 | 123.41 | 53.01 | 2.60 |
| 1 | 1 | Blue | 5 | 2 | B | 14 | 88.34 | 74.56 | 106.22 | 50.70 | 2.60 |
| 1 | 0 | Black | 5 | 2 | R | 14 | 79.84 | 84.20 | 101.07 | 15.65 | 0.41 |
| 1 | 1 | Black | 5 | 2 | R | 14 | 104.29 | 98.01 | 133.20 | 51.59 | 0.41 |
| 1 | 0 | Blue | 5 | 2 | R | 14 | 83.42 | 73.06 | 105.11 | 27.45 | 3.56 |
| 1 | 1 | Blue | 5 | 2 | R | 14 | 99.09 | 89.29 | 120.81 | 42.65 | 3.56 |
| 1 | 0 | Black | 8 | 1 | B | 14 | 86.36 | 77.80 | 110.87 | 13.10 | 2.10 |
| 1 | 1 | Black | 8 | 1 | B | 14 | 94.60 | 89.32 | 125.58 | 41.19 | 2.10 |
| 1 | 0 | Blue | 8 | 1 | B | 14 | 94.28 | 85.85 | 117.16 | 27.38 | 2.21 |
| 1 | 1 | Blue | 8 | 1 | B | 14 | 86.91 | 78.76 | 111.84 | 34.80 | 2.21 |
| 1 | 0 | Black | 8 | 1 | R | 14 | 111.36 | 108.01 | 130.71 | 25.91 | 2.21 |
| 1 | 1 | Black | 8 | 1 | R | 14 | 85.44 | 75.93 | 103.67 | 31.79 | 2.21 |
| 1 | 0 | Blue | 8 | 1 | R | 14 | 105.36 | 101.78 | 133.36 | 36.64 | 0.77 |
| 1 | 1 | Blue | 8 | 1 | R | 14 | 91.20 | 78.76 | 111.90 | 42.96 | 0.77 |
| 1 | 0 | Black | 8 | 2 | B | 14 | 120.52 | 133.40 | 160.99 | 31.88 | 0.86 |
| 1 | 1 | Black | 8 | 2 | B | 14 | 107.49 | 96.27 | 133.97 | 50.57 | 0.86 |
| 1 | 0 | Blue | 8 | 2 | B | 14 | 139.98 | 126.76 | 162.58 | 41.41 | 5.84 |
| 1 | 1 | Blue | 8 | 2 | B | 14 | 98.79 | 85.28 | 113.85 | 40.73 | 5.84 |
| 1 | 0 | Black | 8 | 2 | R | 14 | 90.27 | 93.77 | 124.55 | 26.10 | 1.05 |
| 1 | 1 | Black | 8 | 2 | R | 14 | 105.76 | 89.00 | 122.86 | 52.45 | 1.05 |
| 1 | 0 | Blue | 8 | 2 | R | 14 | 103.04 | 100.10 | 133.07 | 32.64 | 2.90 |
| 1 | 1 | Blue | 8 | 2 | R | 14 | 102.35 | 93.65 | 130.99 | 57.13 | 2.90 |
| 1 | 0 | Black | 10 | 1 | B | 14 | 80.78 | 80.37 | 114.33 | 9.07 | 2.60 |
| 1 | 1 | Black | 10 | 1 | B | 14 | 98.34 | 98.93 | 123.74 | 33.18 | 2.60 |
| 1 | 0 | Blue | 10 | 1 | B | 14 | 96.39 | 99.98 | 134.42 | 17.14 | 0.86 |
| 1 | 1 | Blue | 10 | 1 | B | 14 | 81.03 | 74.04 | 98.22 | 31.27 | 0.86 |
| 1 | 0 | Black | 10 | 1 | R | 14 | 120.81 | 113.42 | 151.20 | 22.66 | 2.90 |
| 1 | 1 | Black | 10 | 1 | R | 14 | 83.05 | 79.67 | 106.78 | 30.71 | 2.90 |
| 1 | 0 | Blue | 10 | 1 | R | 14 | 125.60 | 108.29 | 150.24 | 32.47 | 3.41 |
| 1 | 1 | Blue | 10 | 1 | R | 14 | 76.81 | 67.88 | 97.14 | 32.81 | 3.41 |
| 1 | 0 | Black | 10 | 2 | B | 14 | 127.82 | 126.03 | 156.05 | 38.19 | 0.99 |
| 1 | 1 | Black | 10 | 2 | B | 14 | 91.16 | 74.21 | 102.81 | 46.15 | 0.99 |
| 1 | 0 | Blue | 10 | 2 | B | 14 | 130.94 | 126.46 | 156.13 | 48.34 | 5.00 |
| 1 | 1 | Blue | 10 | 2 | B | 14 | 89.27 | 88.91 | 122.75 | 54.43 | 5.00 |
| 1 | 0 | Black | 10 | 2 | R | 14 | 85.08 | 84.73 | 107.13 | 22.96 | 1.34 |
| 1 | 1 | Black | 10 | 2 | R | 14 | 88.26 | 74.26 | 106.01 | 36.88 | 1.34 |
| 1 | 0 | Blue | 10 | 2 | R | 14 | 91.04 | 85.34 | 113.27 | 35.47 | 0.39 |
| 1 | 1 | Blue | 10 | 2 | R | 14 | 95.60 | 88.29 | 123.74 | 46.53 | 0.39 |
| 1 | 0 | Black | 12 | 1 | B | 14 | 88.51 | 91.82 | 118.17 | 9.21 | 3.56 |
| 1 | 1 | Black | 12 | 1 | B | 14 | 79.54 | 71.51 | 95.91 | 29.40 | 3.56 |
| 1 | 0 | Blue | 12 | 1 | B | 14 | 93.94 | 96.36 | 128.87 | 16.21 | 0.41 |
| 1 | 1 | Blue | 12 | 1 | B | 14 | 85.76 | 73.96 | 105.05 | 40.40 | 0.41 |
| 1 | 0 | Black | 12 | 1 | R | 14 | 80.98 | 81.84 | 112.51 | 19.92 | 3.41 |
| 1 | 1 | Black | 12 | 1 | R | 14 | 81.33 | 79.80 | 106.43 | 34.92 | 3.41 |
| 1 | 0 | Blue | 12 | 1 | R | 14 | 101.28 | 97.44 | 132.25 | 25.20 | 2.63 |
| 1 | 1 | Blue | 12 | 1 | R | 14 | 79.43 | 76.17 | 104.83 | 42.28 | 2.63 |
| 1 | 0 | Black | 12 | 2 | B | 14 | 91.61 | 87.68 | 119.00 | 26.11 | 1.64 |
| 1 | 1 | Black | 12 | 2 | B | 14 | 100.33 | 94.72 | 131.25 | 46.37 | 1.64 |
| 1 | 0 | Blue | 12 | 2 | B | 14 | 93.66 | 82.11 | 117.50 | 35.28 | 1.64 |
| 1 | 1 | Blue | 12 | 2 | B | 14 | 97.35 | 92.97 | 127.16 | 44.46 | 1.64 |
| 1 | 0 | Black | 12 | 2 | R | 14 | 136.80 | 134.47 | 166.35 | 20.92 | 2.05 |
| 1 | 1 | Black | 12 | 2 | R | 14 | 100.34 | 95.14 | 127.33 | 38.06 | 2.05 |
| 1 | 0 | Blue | 12 | 2 | R | 14 | 131.43 | 125.24 | 160.76 | 28.19 | 0.99 |
| 1 | 1 | Blue | 12 | 2 | R | 14 | 96.54 | 80.00 | 112.83 | 40.82 | 0.99 |
| 1 | 0 | Black | 15 | 1 | B | 14 | 132.76 | 131.91 | 179.02 | 19.64 | 5.84 |
| 1 | 1 | Black | 15 | 1 | B | 14 | 69.42 | 61.93 | 84.74 | 21.53 | 5.84 |
| 1 | 0 | Blue | 15 | 1 | B | 14 | 133.34 | 124.57 | 172.69 | 25.80 | 1.05 |
| 1 | 1 | Blue | 15 | 1 | B | 14 | 66.94 | 60.18 | 84.86 | 22.94 | 1.05 |
| 1 | 0 | Black | 15 | 1 | R | 14 | 92.50 | 96.26 | 124.85 | 14.98 | 5.00 |
| 1 | 1 | Black | 15 | 1 | R | 14 | 78.59 | 80.98 | 108.54 | 31.04 | 5.00 |
| 1 | 0 | Blue | 15 | 1 | R | 14 | 94.62 | 88.50 | 122.05 | 23.56 | 3.05 |
| 1 | 1 | Blue | 15 | 1 | R | 14 | 91.20 | 82.98 | 114.59 | 43.90 | 3.05 |
| 1 | 0 | Black | 15 | 2 | B | 14 | 84.28 | 79.90 | 106.73 | 18.75 | 2.63 |
| 1 | 1 | Black | 15 | 2 | B | 14 | 88.67 | 87.37 | 122.42 | 44.19 | 2.63 |
| 1 | 0 | Blue | 15 | 2 | B | 14 | 85.11 | 74.54 | 105.32 | 23.56 | 2.05 |
| 1 | 1 | Blue | 15 | 2 | B | 14 | 82.25 | 79.12 | 111.58 | 36.65 | 2.05 |
| 1 | 0 | Black | 15 | 2 | R | 14 | 128.98 | 125.82 | 159.97 | 19.55 | 3.05 |
| 1 | 1 | Black | 15 | 2 | R | 14 | 89.61 | 77.64 | 108.56 | 31.17 | 3.05 |
| 1 | 0 | Blue | 15 | 2 | R | 14 | 134.64 | 128.12 | 167.38 | 26.24 | 1.34 |
| 1 | 1 | Blue | 15 | 2 | R | 14 | 83.03 | 82.50 | 111.34 | 28.13 | 1.34 |
| 2 | 0 | Black | 5 | 1 | B | 14 | 108.57 | 112.29 | 129.24 | 30.83 | 0.65 |
| 2 | 1 | Black | 5 | 1 | B | 14 | 111.72 | 99.30 | 131.39 | 59.69 | 0.65 |
| 2 | 0 | Blue | 5 | 1 | B | 14 | 114.36 | 108.92 | 135.85 | 35.21 | 0.65 |
| 2 | 1 | Blue | 5 | 1 | B | 14 | 97.62 | 69.25 | 99.49 | 50.63 | 0.65 |
| 2 | 0 | Black | 5 | 1 | R | 14 | 116.32 | 126.34 | 151.15 | 28.37 | 0.77 |
| 2 | 1 | Black | 5 | 1 | R | 14 | 112.17 | 86.69 | 115.85 | 46.74 | 0.77 |
| 2 | 0 | Blue | 5 | 1 | R | 14 | 125.70 | 120.16 | 150.39 | 51.49 | 0.77 |
| 2 | 1 | Blue | 5 | 1 | R | 14 | 120.24 | 101.54 | 131.20 | 53.67 | 0.77 |
| 2 | 0 | Black | 5 | 2 | B | 14 | 112.50 | 110.41 | 138.41 | 43.16 | 0.28 |
| 2 | 1 | Black | 5 | 2 | B | 14 | 129.80 | 127.59 | 162.89 | 72.24 | 0.28 |
| 2 | 0 | Blue | 5 | 2 | B | 14 | 128.05 | 109.08 | 146.91 | 77.56 | 0.28 |
| 2 | 1 | Blue | 5 | 2 | B | 14 | 114.77 | 91.42 | 128.00 | 65.92 | 0.28 |
| 2 | 0 | Black | 5 | 2 | R | 14 | 106.34 | 97.70 | 130.03 | 42.42 | 0.28 |
| 2 | 1 | Black | 5 | 2 | R | 14 | 113.40 | 101.94 | 133.06 | 55.19 | 0.28 |
| 2 | 0 | Blue | 5 | 2 | R | 14 | 129.68 | 106.68 | 157.55 | 78.08 | 0.28 |
| 2 | 1 | Blue | 5 | 2 | R | 14 | 110.61 | 85.96 | 123.59 | 67.33 | 0.28 |
| 2 | 0 | Black | 8 | 1 | B | 14 | 104.29 | 113.22 | 137.13 | 13.65 | 0.70 |
| 2 | 1 | Black | 8 | 1 | B | 14 | 104.37 | 93.38 | 124.85 | 57.20 | 0.70 |
| 2 | 0 | Blue | 8 | 1 | B | 14 | 109.48 | 111.94 | 145.62 | 27.81 | 0.70 |
| 2 | 1 | Blue | 8 | 1 | B | 14 | 101.43 | 81.57 | 113.63 | 50.55 | 0.70 |
| 2 | 0 | Black | 8 | 1 | R | 14 | 102.50 | 100.51 | 128.69 | 24.48 | 1.44 |
| 2 | 1 | Black | 8 | 1 | R | 14 | 103.36 | 89.06 | 118.81 | 53.13 | 1.44 |
| 2 | 0 | Blue | 8 | 1 | R | 14 | 117.57 | 116.24 | 153.68 | 40.10 | 1.44 |
| 2 | 1 | Blue | 8 | 1 | R | 14 | 95.84 | 74.70 | 105.23 | 46.50 | 1.44 |
| 2 | 0 | Black | 8 | 2 | B | 14 | 99.66 | 91.95 | 120.39 | 35.69 | 0.42 |
| 2 | 1 | Black | 8 | 2 | B | 14 | 108.57 | 107.20 | 138.19 | 57.24 | 0.42 |
| 2 | 0 | Blue | 8 | 2 | B | 14 | 115.61 | 105.89 | 140.75 | 63.33 | 0.42 |
| 2 | 1 | Blue | 8 | 2 | B | 14 | 107.73 | 92.71 | 124.75 | 60.87 | 0.42 |
| 2 | 0 | Black | 8 | 2 | R | 14 | 115.36 | 111.94 | 135.68 | 48.62 | 0.76 |
| 2 | 1 | Black | 8 | 2 | R | 14 | 102.33 | 84.09 | 114.87 | 49.41 | 0.76 |
| 2 | 0 | Blue | 8 | 2 | R | 14 | 127.28 | 126.88 | 162.53 | 40.41 | 0.76 |
| 2 | 1 | Blue | 8 | 2 | R | 14 | 112.68 | 95.02 | 131.05 | 60.21 | 0.76 |
| 2 | 0 | Black | 10 | 1 | B | 14 | 104.13 | 99.91 | 128.06 | 19.70 | 1.92 |
| 2 | 1 | Black | 10 | 1 | B | 14 | 106.65 | 102.67 | 135.14 | 57.21 | 1.92 |
| 2 | 0 | Blue | 10 | 1 | B | 14 | 110.59 | 107.41 | 145.54 | 41.37 | 1.92 |
| 2 | 1 | Blue | 10 | 1 | B | 14 | 101.39 | 78.76 | 109.33 | 54.06 | 1.92 |
| 2 | 0 | Black | 10 | 1 | R | 14 | 109.57 | 115.58 | 144.50 | 11.92 | 2.09 |
| 2 | 1 | Black | 10 | 1 | R | 14 | 93.38 | 81.53 | 111.62 | 49.37 | 2.09 |
| 2 | 0 | Blue | 10 | 1 | R | 14 | 131.30 | 123.35 | 160.70 | 28.64 | 2.09 |
| 2 | 1 | Blue | 10 | 1 | R | 14 | 91.63 | 81.65 | 109.91 | 44.22 | 2.09 |
| 2 | 0 | Black | 10 | 2 | B | 14 | 103.43 | 121.08 | 143.32 | 25.07 | 0.53 |
| 2 | 1 | Black | 10 | 2 | B | 14 | 106.28 | 103.15 | 134.83 | 58.39 | 0.53 |
| 2 | 0 | Blue | 10 | 2 | B | 14 | 114.33 | 106.38 | 149.94 | 60.35 | 0.53 |
| 2 | 1 | Blue | 10 | 2 | B | 14 | 89.58 | 75.65 | 105.06 | 50.04 | 0.53 |
| 2 | 0 | Black | 10 | 2 | R | 14 | 107.98 | 98.63 | 120.52 | 43.16 | 0.62 |
| 2 | 1 | Black | 10 | 2 | R | 14 | 113.42 | 99.61 | 128.62 | 60.27 | 0.62 |
| 2 | 0 | Blue | 10 | 2 | R | 14 | 121.40 | 105.34 | 143.28 | 66.65 | 0.62 |
| 2 | 1 | Blue | 10 | 2 | R | 14 | 94.75 | 76.09 | 106.04 | 51.60 | 0.62 |
| 2 | 0 | Black | 12 | 1 | B | 14 | 111.90 | 123.71 | 149.92 | 13.66 | 3.01 |
| 2 | 1 | Black | 12 | 1 | B | 14 | 96.38 | 89.00 | 119.52 | 49.54 | 3.01 |
| 2 | 0 | Blue | 12 | 1 | B | 14 | 114.47 | 115.90 | 149.66 | 30.33 | 3.01 |
| 2 | 1 | Blue | 12 | 1 | B | 14 | 87.77 | 64.97 | 92.67 | 37.42 | 3.01 |
| 2 | 0 | Black | 12 | 1 | R | 14 | 94.39 | 93.36 | 113.91 | 10.21 | 2.76 |
| 2 | 1 | Black | 12 | 1 | R | 14 | 102.29 | 99.99 | 132.52 | 57.87 | 2.76 |
| 2 | 0 | Blue | 12 | 1 | R | 14 | 99.50 | 92.45 | 121.23 | 26.60 | 2.76 |
| 2 | 1 | Blue | 12 | 1 | R | 14 | 93.66 | 73.94 | 101.55 | 48.97 | 2.76 |
| 2 | 0 | Black | 12 | 2 | B | 14 | 128.55 | 117.87 | 152.57 | 34.78 | 1.11 |
| 2 | 1 | Black | 12 | 2 | B | 14 | 89.94 | 79.34 | 107.39 | 40.39 | 1.11 |
| 2 | 0 | Blue | 12 | 2 | B | 14 | 134.79 | 121.62 | 168.56 | 69.23 | 1.11 |
| 2 | 1 | Blue | 12 | 2 | B | 14 | 101.80 | 90.72 | 123.29 | 53.98 | 1.11 |
| 2 | 0 | Black | 12 | 2 | R | 14 | 126.48 | 122.71 | 158.13 | 32.21 | 1.25 |
| 2 | 1 | Black | 12 | 2 | R | 14 | 97.39 | 79.32 | 105.66 | 47.46 | 1.25 |
| 2 | 0 | Blue | 12 | 2 | R | 14 | 112.53 | 96.24 | 138.40 | 58.41 | 1.25 |
| 2 | 1 | Blue | 12 | 2 | R | 14 | 88.38 | 66.70 | 96.10 | 50.52 | 1.25 |
| 2 | 0 | Black | 15 | 1 | B | 14 | 103.89 | 103.27 | 132.47 | 18.52 | 4.49 |
| 2 | 1 | Black | 15 | 1 | B | 14 | 106.80 | 95.37 | 124.11 | 47.81 | 4.49 |
| 2 | 0 | Blue | 15 | 1 | B | 14 | 110.94 | 101.10 | 138.44 | 35.11 | 4.49 |
| 2 | 1 | Blue | 15 | 1 | B | 14 | 96.64 | 83.16 | 112.35 | 47.00 | 4.49 |
| 2 | 0 | Black | 15 | 1 | R | 14 | 101.80 | 91.09 | 121.68 | 18.87 | 4.72 |
| 2 | 1 | Black | 15 | 1 | R | 14 | 93.75 | 82.69 | 108.36 | 38.39 | 4.72 |
| 2 | 0 | Blue | 15 | 1 | R | 14 | 108.21 | 98.26 | 136.51 | 32.52 | 4.72 |
| 2 | 1 | Blue | 15 | 1 | R | 14 | 83.73 | 64.60 | 90.99 | 34.54 | 4.72 |
| 2 | 0 | Black | 15 | 2 | B | 14 | 98.36 | 92.32 | 123.68 | 34.55 | 1.42 |
| 2 | 1 | Black | 15 | 2 | B | 14 | 101.22 | 94.60 | 122.83 | 55.24 | 1.42 |
| 2 | 0 | Blue | 15 | 2 | B | 14 | 113.68 | 97.09 | 135.68 | 58.37 | 1.42 |
| 2 | 1 | Blue | 15 | 2 | B | 14 | 97.93 | 79.40 | 108.33 | 54.93 | 1.42 |
| 2 | 0 | Black | 15 | 2 | R | 14 | 109.17 | 118.30 | 146.09 | 19.13 | 1.45 |
| 2 | 1 | Black | 15 | 2 | R | 14 | 99.93 | 93.48 | 121.36 | 46.80 | 1.45 |
| 2 | 0 | Blue | 15 | 2 | R | 14 | 125.82 | 119.60 | 158.60 | 58.41 | 1.45 |
| 2 | 1 | Blue | 15 | 2 | R | 14 | 105.11 | 95.67 | 127.04 | 58.08 | 1.45 |
| 3 | 0 | Black | 5 | 1 | B | 14 | 114.90 | 130.36 | 154.24 | 32.88 | 0.78 |
| 3 | 1 | Black | 5 | 1 | B | 14 | 121.55 | 111.23 | 147.58 | 62.18 | 0.78 |
| 3 | 0 | Blue | 5 | 1 | B | 14 | 131.64 | 112.09 | 144.07 | 54.96 | 0.78 |
| 3 | 1 | Blue | 5 | 1 | B | 14 | 75.92 | 71.25 | 100.54 | 43.56 | 0.78 |
| 3 | 0 | Black | 5 | 1 | R | 14 | 130.06 | 125.93 | 161.06 | 33.48 | 0.97 |
| 3 | 1 | Black | 5 | 1 | R | 14 | 81.20 | 71.71 | 95.33 | 30.38 | 0.97 |
| 3 | 0 | Blue | 5 | 1 | R | 14 | 144.57 | 131.57 | 174.33 | 48.33 | 0.97 |
| 3 | 1 | Blue | 5 | 1 | R | 14 | 81.47 | 66.15 | 95.39 | 39.82 | 0.97 |
| 3 | 0 | Black | 5 | 2 | B | 14 | 101.80 | 107.23 | 125.59 | 32.91 | 0.45 |
| 3 | 1 | Black | 5 | 2 | B | 14 | 102.53 | 79.31 | 108.75 | 53.72 | 0.45 |
| 3 | 0 | Blue | 5 | 2 | B | 14 | 118.29 | 113.19 | 135.36 | 46.08 | 0.45 |
| 3 | 1 | Blue | 5 | 2 | B | 14 | 98.11 | 75.47 | 109.19 | 57.61 | 0.45 |
| 3 | 0 | Black | 5 | 2 | R | 14 | 134.48 | 127.02 | 144.32 | 61.69 | 0.43 |
| 3 | 1 | Black | 5 | 2 | R | 14 | 111.37 | 92.87 | 126.55 | 59.04 | 0.43 |
| 3 | 0 | Blue | 5 | 2 | R | 14 | 122.58 | 112.68 | 153.23 | 59.74 | 0.43 |
| 3 | 1 | Blue | 5 | 2 | R | 14 | 103.23 | 87.68 | 123.58 | 58.39 | 0.43 |
| 3 | 0 | Black | 8 | 1 | B | 14 | 110.70 | 111.18 | 143.32 | 14.69 | 1.76 |
| 3 | 1 | Black | 8 | 1 | B | 14 | 92.56 | 78.52 | 105.25 | 38.81 | 1.76 |
| 3 | 0 | Blue | 8 | 1 | B | 14 | 124.25 | 117.72 | 157.99 | 22.25 | 1.76 |
| 3 | 1 | Blue | 8 | 1 | B | 14 | 85.33 | 63.51 | 92.73 | 43.49 | 1.76 |
| 3 | 0 | Black | 8 | 1 | R | 14 | 131.21 | 127.08 | 160.20 | 20.53 | 1.58 |
| 3 | 1 | Black | 8 | 1 | R | 14 | 88.21 | 81.24 | 112.12 | 52.48 | 1.58 |
| 3 | 0 | Blue | 8 | 1 | R | 14 | 125.42 | 124.63 | 166.62 | 38.00 | 1.58 |
| 3 | 1 | Blue | 8 | 1 | R | 14 | 85.57 | 72.76 | 103.44 | 49.68 | 1.58 |
| 3 | 0 | Black | 8 | 2 | B | 14 | 129.13 | 109.99 | 142.61 | 65.85 | 0.86 |
| 3 | 1 | Black | 8 | 2 | B | 14 | 99.27 | 85.97 | 118.03 | 53.90 | 0.86 |
| 3 | 0 | Blue | 8 | 2 | B | 14 | 128.88 | 128.91 | 167.09 | 78.01 | 0.86 |
| 3 | 1 | Blue | 8 | 2 | B | 14 | 98.25 | 74.68 | 107.97 | 50.53 | 0.86 |
| 3 | 0 | Black | 8 | 2 | R | 14 | 127.15 | 123.37 | 154.29 | 26.13 | 1.60 |
| 3 | 1 | Black | 8 | 2 | R | 14 | 99.22 | 87.20 | 117.72 | 46.06 | 1.60 |
| 3 | 0 | Blue | 8 | 2 | R | 14 | 133.23 | 132.26 | 173.57 | 42.19 | 1.60 |
| 3 | 1 | Blue | 8 | 2 | R | 14 | 98.92 | 83.26 | 116.46 | 46.56 | 1.60 |
| 3 | 0 | Black | 10 | 1 | B | 14 | 111.36 | 108.98 | 136.81 | 25.35 | 2.38 |
| 3 | 1 | Black | 10 | 1 | B | 14 | 89.25 | 78.80 | 110.65 | 47.05 | 2.38 |
| 3 | 0 | Blue | 10 | 1 | B | 14 | 128.87 | 110.06 | 146.74 | 33.78 | 2.38 |
| 3 | 1 | Blue | 10 | 1 | B | 14 | 88.62 | 77.74 | 108.90 | 48.41 | 2.38 |
| 3 | 0 | Black | 10 | 1 | R | 14 | 118.72 | 118.66 | 146.09 | 12.35 | 2.93 |
| 3 | 1 | Black | 10 | 1 | R | 14 | 78.81 | 64.88 | 92.24 | 36.90 | 2.93 |
| 3 | 0 | Blue | 10 | 1 | R | 14 | 123.43 | 109.27 | 140.40 | 27.59 | 2.93 |
| 3 | 1 | Blue | 10 | 1 | R | 14 | 85.12 | 74.40 | 105.97 | 44.91 | 2.93 |
| 3 | 0 | Black | 10 | 2 | B | 14 | 138.75 | 133.21 | 170.86 | 28.12 | 1.01 |
| 3 | 1 | Black | 10 | 2 | B | 14 | 83.20 | 74.47 | 97.63 | 33.79 | 1.01 |
| 3 | 0 | Blue | 10 | 2 | B | 14 | 152.91 | 128.18 | 176.39 | 67.40 | 1.01 |
| 3 | 1 | Blue | 10 | 2 | B | 14 | 97.28 | 85.26 | 117.41 | 55.73 | 1.01 |
| 3 | 0 | Black | 10 | 2 | R | 14 | 130.22 | 129.09 | 153.96 | 28.07 | 1.09 |
| 3 | 1 | Black | 10 | 2 | R | 14 | 79.26 | 92.63 | 128.16 | 52.55 | 1.09 |
| 3 | 0 | Blue | 10 | 2 | R | 14 | 126.33 | 114.06 | 144.98 | 42.81 | 1.09 |
| 3 | 1 | Blue | 10 | 2 | R | 14 | 91.59 | 76.62 | 109.11 | 53.87 | 1.09 |
| 3 | 0 | Black | 12 | 1 | B | 14 | 133.65 | 114.95 | 154.23 | 26.56 | 3.76 |
| 3 | 1 | Black | 12 | 1 | B | 14 | 76.23 | 59.92 | 82.33 | 24.83 | 3.76 |
| 3 | 0 | Blue | 12 | 1 | B | 14 | 134.62 | 109.00 | 151.97 | 41.93 | 3.76 |
| 3 | 1 | Blue | 12 | 1 | B | 14 | 77.95 | 64.36 | 92.22 | 38.70 | 3.76 |
| 3 | 0 | Black | 12 | 1 | R | 14 | 118.18 | 119.64 | 147.97 | 14.59 | 3.10 |
| 3 | 1 | Black | 12 | 1 | R | 14 | 98.60 | 85.51 | 115.93 | 51.43 | 3.10 |
| 3 | 0 | Blue | 12 | 1 | R | 14 | 117.69 | 116.31 | 152.24 | 28.05 | 3.10 |
| 3 | 1 | Blue | 12 | 1 | R | 14 | 65.13 | 56.02 | 82.29 | 34.55 | 3.10 |
| 3 | 0 | Black | 12 | 2 | B | 14 | 136.68 | 130.42 | 169.36 | 17.95 | 1.28 |
| 3 | 1 | Black | 12 | 2 | B | 14 | 100.43 | 88.14 | 117.85 | 47.14 | 1.28 |
| 3 | 0 | Blue | 12 | 2 | B | 14 | 152.52 | 125.58 | 174.00 | 69.67 | 1.28 |
| 3 | 1 | Blue | 12 | 2 | B | 14 | 100.82 | 94.78 | 128.76 | 55.05 | 1.28 |
| 3 | 0 | Black | 12 | 2 | R | 14 | 113.99 | 114.24 | 138.63 | 20.61 | 1.57 |
| 3 | 1 | Black | 12 | 2 | R | 14 | 103.07 | 96.60 | 131.14 | 59.49 | 1.57 |
| 3 | 0 | Blue | 12 | 2 | R | 14 | 106.08 | 94.43 | 121.03 | 48.70 | 1.57 |
| 3 | 1 | Blue | 12 | 2 | R | 14 | 95.61 | 76.36 | 107.84 | 55.54 | 1.57 |
| 3 | 0 | Black | 15 | 1 | B | 14 | 115.64 | 117.47 | 150.54 | 8.05 | 4.89 |
| 3 | 1 | Black | 15 | 1 | B | 14 | 91.72 | 84.57 | 112.13 | 39.46 | 4.89 |
| 3 | 0 | Blue | 15 | 1 | B | 14 | 121.86 | 121.55 | 161.73 | 15.91 | 4.89 |
| 3 | 1 | Blue | 15 | 1 | B | 14 | 78.58 | 66.19 | 94.25 | 36.07 | 4.89 |
| 3 | 0 | Black | 15 | 1 | R | 14 | 128.63 | 121.35 | 155.71 | 15.43 | 5.08 |
| 3 | 1 | Black | 15 | 1 | R | 14 | 94.65 | 79.07 | 106.06 | 38.26 | 5.08 |
| 3 | 0 | Blue | 15 | 1 | R | 14 | 143.08 | 121.69 | 163.54 | 33.68 | 5.08 |
| 3 | 1 | Blue | 15 | 1 | R | 14 | 96.22 | 82.70 | 113.10 | 41.65 | 5.08 |
| 3 | 0 | Black | 15 | 2 | B | 14 | 115.33 | 110.98 | 137.24 | 17.54 | 2.57 |
| 3 | 1 | Black | 15 | 2 | B | 14 | 84.89 | 83.52 | 111.94 | 35.43 | 2.57 |
| 3 | 0 | Blue | 15 | 2 | B | 14 | 114.31 | 109.77 | 142.67 | 24.89 | 2.57 |
| 3 | 1 | Blue | 15 | 2 | B | 14 | 84.74 | 81.33 | 107.77 | 30.48 | 2.57 |
| 3 | 0 | Black | 15 | 2 | R | 14 | 127.21 | 135.21 | 168.69 | 13.61 | 2.25 |
| 3 | 1 | Black | 15 | 2 | R | 14 | 97.31 | 79.68 | 106.97 | 37.43 | 2.25 |
| 3 | 0 | Blue | 15 | 2 | R | 14 | 158.95 | 146.12 | 191.41 | 59.97 | 2.25 |
| 3 | 1 | Blue | 15 | 2 | R | 14 | 97.59 | 77.37 | 107.57 | 44.69 | 2.25 |
| 4 | 0 | Black | 5 | 1 | B | 14 | 107.85 | 103.73 | 119.71 | 26.49 | 0.91 |
| 4 | 1 | Black | 5 | 1 | B | 14 | 108.66 | 96.74 | 128.98 | 55.16 | 0.91 |
| 4 | 0 | Blue | 5 | 1 | B | 14 | 106.14 | 101.23 | 125.37 | 35.29 | 0.91 |
| 4 | 1 | Blue | 5 | 1 | B | 14 | 104.70 | 99.57 | 135.67 | 55.71 | 0.91 |
| 4 | 0 | Black | 5 | 1 | R | 14 | 88.58 | 78.04 | 105.71 | 22.29 | 1.00 |
| 4 | 1 | Black | 5 | 1 | R | 14 | 112.25 | 99.48 | 133.52 | 48.67 | 1.00 |
| 4 | 0 | Blue | 5 | 1 | R | 14 | 88.96 | 71.29 | 109.66 | 32.77 | 1.00 |
| 4 | 1 | Blue | 5 | 1 | R | 14 | 106.89 | 89.94 | 125.02 | 46.96 | 1.00 |
| 4 | 0 | Black | 5 | 2 | B | 14 | 99.70 | 100.18 | 112.32 | 39.59 | 0.38 |
| 4 | 1 | Black | 5 | 2 | B | 14 | 122.21 | 109.05 | 142.81 | 61.59 | 0.38 |
| 4 | 0 | Blue | 5 | 2 | B | 14 | 101.49 | 92.41 | 121.83 | 38.45 | 0.38 |
| 4 | 1 | Blue | 5 | 2 | B | 14 | 111.53 | 83.43 | 118.12 | 60.05 | 0.38 |
| 4 | 0 | Black | 5 | 2 | R | 14 | 118.24 | 110.02 | 134.77 | 68.73 | 0.38 |
| 4 | 1 | Black | 5 | 2 | R | 14 | 113.63 | 95.24 | 127.02 | 58.07 | 0.38 |
| 4 | 0 | Blue | 5 | 2 | R | 14 | 119.73 | 114.01 | 140.25 | 49.87 | 0.38 |
| 4 | 1 | Blue | 5 | 2 | R | 14 | 107.17 | 86.78 | 118.62 | 52.97 | 0.38 |
| 4 | 0 | Black | 8 | 1 | B | 14 | 94.99 | 82.56 | 114.53 | 34.45 | 2.22 |
| 4 | 1 | Black | 8 | 1 | B | 14 | 101.66 | 92.16 | 125.47 | 40.42 | 2.22 |
| 4 | 0 | Blue | 8 | 1 | B | 14 | 105.00 | 90.34 | 130.14 | 44.02 | 2.22 |
| 4 | 1 | Blue | 8 | 1 | B | 14 | 100.87 | 77.81 | 109.85 | 40.13 | 2.22 |
| 4 | 0 | Black | 8 | 1 | R | 14 | 93.38 | 88.23 | 111.55 | 10.77 | 1.79 |
| 4 | 1 | Black | 8 | 1 | R | 14 | 104.73 | 92.41 | 121.36 | 34.73 | 1.79 |
| 4 | 0 | Blue | 8 | 1 | R | 14 | 110.58 | 102.40 | 135.07 | 22.25 | 1.79 |
| 4 | 1 | Blue | 8 | 1 | R | 14 | 105.12 | 81.16 | 112.87 | 43.45 | 1.79 |
| 4 | 0 | Black | 8 | 2 | B | 14 | 105.56 | 102.44 | 133.39 | 30.47 | 0.65 |
| 4 | 1 | Black | 8 | 2 | B | 14 | 112.02 | 104.89 | 138.98 | 55.51 | 0.65 |
| 4 | 0 | Blue | 8 | 2 | B | 14 | 116.40 | 109.21 | 145.22 | 42.32 | 0.65 |
| 4 | 1 | Blue | 8 | 2 | B | 14 | 109.35 | 101.32 | 134.77 | 57.53 | 0.65 |
| 4 | 0 | Black | 8 | 2 | R | 14 | 79.30 | 69.54 | 96.71 | 19.55 | 0.87 |
| 4 | 1 | Black | 8 | 2 | R | 14 | 112.07 | 102.92 | 138.74 | 51.66 | 0.87 |
| 4 | 0 | Blue | 8 | 2 | R | 14 | 88.83 | 72.56 | 108.05 | 31.54 | 0.87 |
| 4 | 1 | Blue | 8 | 2 | R | 14 | 103.80 | 92.60 | 126.21 | 49.41 | 0.87 |
| 4 | 0 | Black | 10 | 1 | B | 14 | 89.23 | 87.36 | 112.56 | 13.63 | 2.90 |
| 4 | 1 | Black | 10 | 1 | B | 14 | 101.36 | 91.17 | 122.64 | 38.90 | 2.90 |
| 4 | 0 | Blue | 10 | 1 | B | 14 | 102.77 | 100.78 | 132.41 | 19.40 | 2.90 |
| 4 | 1 | Blue | 10 | 1 | B | 14 | 107.44 | 111.58 | 141.32 | 39.11 | 2.90 |
| 4 | 0 | Black | 10 | 1 | R | 14 | 98.80 | 103.45 | 126.91 | 17.34 | 2.48 |
| 4 | 1 | Black | 10 | 1 | R | 14 | 107.22 | 97.90 | 130.70 | 53.24 | 2.48 |
| 4 | 0 | Blue | 10 | 1 | R | 14 | 119.45 | 116.33 | 150.10 | 36.48 | 2.48 |
| 4 | 1 | Blue | 10 | 1 | R | 14 | 103.84 | 89.77 | 123.51 | 49.90 | 2.48 |
| 4 | 0 | Black | 10 | 2 | B | 14 | 95.36 | 92.84 | 118.51 | 29.66 | 1.46 |
| 4 | 1 | Black | 10 | 2 | B | 14 | 111.48 | 101.60 | 132.26 | 51.82 | 1.46 |
| 4 | 0 | Blue | 10 | 2 | B | 14 | 114.46 | 100.67 | 134.03 | 50.13 | 1.46 |
| 4 | 1 | Blue | 10 | 2 | B | 14 | 105.40 | 94.31 | 127.79 | 52.95 | 1.46 |
| 4 | 0 | Black | 10 | 2 | R | 14 | 123.46 | 125.97 | 157.79 | 43.57 | 1.18 |
| 4 | 1 | Black | 10 | 2 | R | 14 | 109.22 | 101.58 | 134.90 | 52.11 | 1.18 |
| 4 | 0 | Blue | 10 | 2 | R | 14 | 120.01 | 111.70 | 148.14 | 50.72 | 1.18 |
| 4 | 1 | Blue | 10 | 2 | R | 14 | 108.82 | 101.02 | 134.83 | 54.40 | 1.18 |
| 4 | 0 | Black | 12 | 1 | B | 14 | 102.72 | 104.18 | 132.48 | 12.02 | 3.45 |
| 4 | 1 | Black | 12 | 1 | B | 14 | 98.11 | 90.28 | 117.56 | 32.80 | 3.45 |
| 4 | 0 | Blue | 12 | 1 | B | 14 | 105.19 | 105.88 | 134.28 | 18.46 | 3.45 |
| 4 | 1 | Blue | 12 | 1 | B | 14 | 94.21 | 76.88 | 105.74 | 41.90 | 3.45 |
| 4 | 0 | Black | 12 | 1 | R | 14 | 86.48 | 81.26 | 106.43 | 11.59 | 3.37 |
| 4 | 1 | Black | 12 | 1 | R | 14 | 103.04 | 82.96 | 109.23 | 43.07 | 3.37 |
| 4 | 0 | Blue | 12 | 1 | R | 14 | 95.18 | 88.24 | 120.34 | 20.16 | 3.37 |
| 4 | 1 | Blue | 12 | 1 | R | 14 | 106.68 | 95.31 | 129.09 | 51.69 | 3.37 |
| 4 | 0 | Black | 12 | 2 | B | 14 | 114.02 | 105.02 | 142.13 | 55.93 | 1.33 |
| 4 | 1 | Black | 12 | 2 | B | 14 | 102.93 | 88.36 | 117.96 | 52.23 | 1.33 |
| 4 | 0 | Blue | 12 | 2 | B | 14 | 99.11 | 76.98 | 116.86 | 55.98 | 1.33 |
| 4 | 1 | Blue | 12 | 2 | B | 14 | 97.81 | 86.17 | 118.31 | 52.56 | 1.33 |
| 4 | 0 | Black | 12 | 2 | R | 14 | 104.42 | 99.86 | 123.78 | 24.92 | 1.41 |
| 4 | 1 | Black | 12 | 2 | R | 14 | 111.59 | 95.62 | 129.60 | 50.13 | 1.41 |
| 4 | 0 | Blue | 12 | 2 | R | 14 | 113.06 | 104.13 | 135.83 | 36.87 | 1.41 |
| 4 | 1 | Blue | 12 | 2 | R | 14 | 109.97 | 96.19 | 129.93 | 52.07 | 1.41 |
| 4 | 0 | Black | 15 | 1 | B | 14 | 95.74 | 92.70 | 127.95 | 24.99 | 5.38 |
| 4 | 1 | Black | 15 | 1 | B | 14 | 94.48 | 90.29 | 121.62 | 36.78 | 5.38 |
| 4 | 0 | Blue | 15 | 1 | B | 14 | 99.47 | 85.82 | 125.01 | 37.90 | 5.38 |
| 4 | 1 | Blue | 15 | 1 | B | 14 | 93.41 | 87.12 | 117.70 | 36.15 | 5.38 |
| 4 | 0 | Black | 15 | 1 | R | 14 | 90.96 | 96.39 | 115.77 | 14.75 | 4.54 |
| 4 | 1 | Black | 15 | 1 | R | 14 | 97.08 | 93.16 | 124.68 | 47.69 | 4.54 |
| 4 | 0 | Blue | 15 | 1 | R | 14 | 99.53 | 95.48 | 118.22 | 21.68 | 4.54 |
| 4 | 1 | Blue | 15 | 1 | R | 14 | 98.10 | 93.66 | 123.90 | 48.83 | 4.54 |
| 4 | 0 | Black | 15 | 2 | B | 14 | 103.52 | 101.33 | 126.80 | 15.05 | 2.06 |
| 4 | 1 | Black | 15 | 2 | B | 14 | 107.50 | 99.13 | 127.24 | 40.39 | 2.06 |
| 4 | 0 | Blue | 15 | 2 | B | 14 | 112.86 | 106.66 | 139.19 | 25.61 | 2.06 |
| 4 | 1 | Blue | 15 | 2 | B | 14 | 107.95 | 90.47 | 122.59 | 45.39 | 2.06 |
| 4 | 0 | Black | 15 | 2 | R | 14 | 110.91 | 109.49 | 134.26 | 33.63 | 2.10 |
| 4 | 1 | Black | 15 | 2 | R | 14 | 109.96 | 104.79 | 139.93 | 46.95 | 2.10 |
| 4 | 0 | Blue | 15 | 2 | R | 14 | 130.21 | 119.15 | 156.18 | 60.94 | 2.10 |
| 4 | 1 | Blue | 15 | 2 | R | 14 | 113.88 | 107.93 | 143.53 | 58.72 | 2.10 |
| 5 | 0 | Black | 5 | 1 | B | 14 | 115.47 | 112.64 | 142.95 | 42.64 | 0.87 |
| 5 | 1 | Black | 5 | 1 | B | 14 | 116.08 | 106.56 | 141.85 | 49.25 | 0.87 |
| 5 | 0 | Blue | 5 | 1 | B | 14 | 123.91 | 117.64 | 152.61 | 35.35 | 0.87 |
| 5 | 1 | Blue | 5 | 1 | B | 14 | 102.63 | 94.51 | 127.36 | 46.90 | 0.87 |
| 5 | 0 | Black | 5 | 1 | R | 14 | 78.09 | 62.91 | 90.97 | 21.00 | 0.90 |
| 5 | 1 | Black | 5 | 1 | R | 14 | 109.32 | 85.63 | 117.10 | 49.70 | 0.90 |
| 5 | 0 | Blue | 5 | 1 | R | 14 | 93.69 | 77.81 | 111.89 | 36.30 | 0.90 |
| 5 | 1 | Blue | 5 | 1 | R | 14 | 105.21 | 90.41 | 120.45 | 44.75 | 0.90 |
| 5 | 0 | Black | 5 | 2 | B | 14 | 75.83 | 65.90 | 90.38 | 18.83 | 0.40 |
| 5 | 1 | Black | 5 | 2 | B | 14 | 109.69 | 94.49 | 127.29 | 46.88 | 0.40 |
| 5 | 0 | Blue | 5 | 2 | B | 14 | 90.10 | 74.07 | 104.29 | 29.78 | 0.40 |
| 5 | 1 | Blue | 5 | 2 | B | 14 | 102.74 | 80.86 | 113.63 | 48.42 | 0.40 |
| 5 | 0 | Black | 5 | 2 | R | 14 | 84.90 | 85.27 | 111.00 | 33.59 | 0.39 |
| 5 | 1 | Black | 5 | 2 | R | 14 | 126.72 | 109.28 | 145.07 | 55.71 | 0.39 |
| 5 | 0 | Blue | 5 | 2 | R | 14 | 99.78 | 86.26 | 116.72 | 43.74 | 0.39 |
| 5 | 1 | Blue | 5 | 2 | R | 14 | 109.09 | 85.68 | 118.60 | 52.55 | 0.39 |
| 5 | 0 | Black | 8 | 1 | B | 14 | 80.70 | 70.49 | 95.63 | 13.48 | 1.65 |
| 5 | 1 | Black | 8 | 1 | B | 14 | 92.06 | 82.14 | 109.31 | 35.57 | 1.65 |
| 5 | 0 | Blue | 8 | 1 | B | 14 | 87.20 | 76.36 | 107.43 | 19.80 | 1.65 |
| 5 | 1 | Blue | 8 | 1 | B | 14 | 95.84 | 80.36 | 113.02 | 45.79 | 1.65 |
| 5 | 0 | Black | 8 | 1 | R | 14 | 79.47 | 68.90 | 93.71 | 15.56 | 1.87 |
| 5 | 1 | Black | 8 | 1 | R | 14 | 108.81 | 87.47 | 118.30 | 48.70 | 1.87 |
| 5 | 0 | Blue | 8 | 1 | R | 14 | 85.13 | 66.98 | 96.68 | 28.16 | 1.87 |
| 5 | 1 | Blue | 8 | 1 | R | 14 | 100.53 | 78.33 | 111.12 | 45.28 | 1.87 |
| 5 | 0 | Black | 8 | 2 | B | 14 | 103.02 | 95.24 | 123.91 | 34.06 | 0.77 |
| 5 | 1 | Black | 8 | 2 | B | 14 | 111.56 | 99.38 | 133.85 | 50.96 | 0.77 |
| 5 | 0 | Blue | 8 | 2 | B | 14 | 115.10 | 94.34 | 140.24 | 54.16 | 0.77 |
| 5 | 1 | Blue | 8 | 2 | B | 14 | 104.04 | 80.99 | 110.95 | 39.99 | 0.77 |
| 5 | 0 | Black | 8 | 2 | R | 14 | 91.00 | 84.85 | 110.85 | 24.10 | 0.69 |
| 5 | 1 | Black | 8 | 2 | R | 14 | 119.81 | 104.37 | 139.92 | 56.15 | 0.69 |
| 5 | 0 | Blue | 8 | 2 | R | 14 | 100.83 | 80.69 | 121.47 | 52.72 | 0.69 |
| 5 | 1 | Blue | 8 | 2 | R | 14 | 113.48 | 105.14 | 134.45 | 54.65 | 0.69 |
| 5 | 0 | Black | 10 | 1 | B | 14 | 92.30 | 81.38 | 105.06 | 34.43 | 2.45 |
| 5 | 1 | Black | 10 | 1 | B | 14 | 91.53 | 78.38 | 107.47 | 41.76 | 2.45 |
| 5 | 0 | Blue | 10 | 1 | B | 14 | 92.26 | 84.01 | 116.92 | 47.97 | 2.45 |
| 5 | 1 | Blue | 10 | 1 | B | 14 | 91.93 | 79.45 | 109.10 | 46.83 | 2.45 |
| 5 | 0 | Black | 10 | 1 | R | 14 | 109.59 | 111.60 | 141.63 | 25.33 | 2.45 |
| 5 | 1 | Black | 10 | 1 | R | 14 | 99.96 | 86.02 | 113.04 | 35.48 | 2.45 |
| 5 | 0 | Blue | 10 | 1 | R | 14 | 112.62 | 110.75 | 143.69 | 33.60 | 2.45 |
| 5 | 1 | Blue | 10 | 1 | R | 14 | 101.96 | 96.99 | 131.31 | 45.86 | 2.45 |
| 5 | 0 | Black | 10 | 2 | B | 14 | 84.14 | 69.68 | 98.36 | 40.15 | 1.06 |
| 5 | 1 | Black | 10 | 2 | B | 14 | 96.42 | 85.53 | 118.40 | 41.25 | 1.06 |
| 5 | 0 | Blue | 10 | 2 | B | 14 | 90.05 | 70.13 | 109.79 | 43.29 | 1.06 |
| 5 | 1 | Blue | 10 | 2 | B | 14 | 102.20 | 88.04 | 121.27 | 48.75 | 1.06 |
| 5 | 0 | Black | 10 | 2 | R | 14 | 97.58 | 86.72 | 112.67 | 37.60 | 1.03 |
| 5 | 1 | Black | 10 | 2 | R | 14 | 107.39 | 95.12 | 127.90 | 45.44 | 1.03 |
| 5 | 0 | Blue | 10 | 2 | R | 14 | 100.96 | 91.76 | 118.54 | 43.28 | 1.03 |
| 5 | 1 | Blue | 10 | 2 | R | 14 | 92.19 | 75.79 | 105.83 | 41.59 | 1.03 |
| 5 | 0 | Black | 12 | 1 | B | 14 | 82.96 | 72.31 | 100.88 | 17.85 | 3.79 |
| 5 | 1 | Black | 12 | 1 | B | 14 | 100.81 | 94.00 | 124.77 | 36.32 | 3.79 |
| 5 | 0 | Blue | 12 | 1 | B | 14 | 86.85 | 69.27 | 102.29 | 25.01 | 3.79 |
| 5 | 1 | Blue | 12 | 1 | B | 14 | 92.42 | 66.88 | 95.33 | 33.00 | 3.79 |
| 5 | 0 | Black | 12 | 1 | R | 14 | 99.07 | 90.51 | 122.94 | 26.96 | 3.70 |
| 5 | 1 | Black | 12 | 1 | R | 14 | 100.27 | 92.31 | 120.61 | 39.25 | 3.70 |
| 5 | 0 | Blue | 12 | 1 | R | 14 | 99.74 | 90.44 | 128.58 | 31.96 | 3.70 |
| 5 | 1 | Blue | 12 | 1 | R | 14 | 98.69 | 92.16 | 124.54 | 44.48 | 3.70 |
| 5 | 0 | Black | 12 | 2 | B | 14 | 86.32 | 74.00 | 95.80 | 25.61 | 1.38 |
| 5 | 1 | Black | 12 | 2 | B | 14 | 107.95 | 100.30 | 135.18 | 46.65 | 1.38 |
| 5 | 0 | Blue | 12 | 2 | B | 14 | 92.00 | 71.71 | 104.93 | 40.94 | 1.38 |
| 5 | 1 | Blue | 12 | 2 | B | 14 | 108.12 | 97.25 | 131.08 | 53.71 | 1.38 |
| 5 | 0 | Black | 12 | 2 | R | 14 | 116.39 | 111.65 | 150.23 | 34.86 | 1.55 |
| 5 | 1 | Black | 12 | 2 | R | 14 | 115.77 | 109.23 | 140.24 | 45.37 | 1.55 |
| 5 | 0 | Blue | 12 | 2 | R | 14 | 129.75 | 119.99 | 162.14 | 56.53 | 1.55 |
| 5 | 1 | Blue | 12 | 2 | R | 14 | 108.22 | 94.75 | 128.95 | 48.09 | 1.55 |
| 5 | 0 | Black | 15 | 1 | B | 14 | 103.98 | 110.18 | 138.39 | 12.60 | 4.88 |
| 5 | 1 | Black | 15 | 1 | B | 14 | 97.13 | 93.43 | 122.78 | 34.14 | 4.88 |
| 5 | 0 | Blue | 15 | 1 | B | 14 | 104.87 | 100.98 | 129.44 | 18.35 | 4.88 |
| 5 | 1 | Blue | 15 | 1 | B | 14 | 99.00 | 88.96 | 120.13 | 44.60 | 4.88 |
| 5 | 0 | Black | 15 | 1 | R | 14 | 88.70 | 82.07 | 105.82 | 14.92 | 5.46 |
| 5 | 1 | Black | 15 | 1 | R | 14 | 109.17 | 97.31 | 127.83 | 44.63 | 5.46 |
| 5 | 0 | Blue | 15 | 1 | R | 14 | 88.23 | 80.80 | 107.58 | 16.54 | 5.46 |
| 5 | 1 | Blue | 15 | 1 | R | 14 | 109.30 | 96.05 | 128.84 | 47.16 | 5.46 |
| 5 | 0 | Black | 15 | 2 | B | 14 | 109.68 | 105.48 | 141.14 | 34.28 | 2.49 |
| 5 | 1 | Black | 15 | 2 | B | 14 | 96.06 | 76.10 | 103.12 | 37.49 | 2.49 |
| 5 | 0 | Blue | 15 | 2 | B | 14 | 100.47 | 85.81 | 122.82 | 43.10 | 2.49 |
| 5 | 1 | Blue | 15 | 2 | B | 14 | 99.42 | 84.54 | 114.35 | 47.69 | 2.49 |
| 5 | 0 | Black | 15 | 2 | R | 14 | 91.92 | 88.95 | 110.47 | 9.19 | 2.40 |
| 5 | 1 | Black | 15 | 2 | R | 14 | 103.39 | 85.63 | 115.55 | 48.37 | 2.40 |
| 5 | 0 | Blue | 15 | 2 | R | 14 | 86.56 | 73.87 | 101.23 | 17.81 | 2.40 |
| 5 | 1 | Blue | 15 | 2 | R | 14 | 110.96 | 93.47 | 129.07 | 54.82 | 2.40 |
